# Impact of Gestational Haloperidol Exposure on miR-137-3p and Nr3c1 mRNA Expression in Hippocampus of Offspring Mice

**DOI:** 10.1093/ijnp/pyac044

**Published:** 2022-07-21

**Authors:** Yuta Yoshino, Hiroshi Kumon, Tetsuya Shimokawa, Hajime Yano, Shinichiro Ochi, Yu Funahashi, Jun-ichi Iga, Seiji Matsuda, Junya Tanaka, Shu-ichi Ueno

**Affiliations:** Department of Neuropsychiatry, Molecules and Function, Ehime University Graduate School of Medicine, Shitsukawa, Toon, Ehime, Japan; Department of Neuropsychiatry, Molecules and Function, Ehime University Graduate School of Medicine, Shitsukawa, Toon, Ehime, Japan; Department of Anatomy and Embryology, Ehime University Graduate School of Medicine, Toon, Ehime, Japan; Department of Molecular and Cellular Physiology, Ehime University Graduate School of Medicine, Toon, Ehime, Japan; Department of Neuropsychiatry, Molecules and Function, Ehime University Graduate School of Medicine, Shitsukawa, Toon, Ehime, Japan; Department of Neuropsychiatry, Molecules and Function, Ehime University Graduate School of Medicine, Shitsukawa, Toon, Ehime, Japan; Department of Neuropsychiatry, Molecules and Function, Ehime University Graduate School of Medicine, Shitsukawa, Toon, Ehime, Japan; Department of Anatomy and Embryology, Ehime University Graduate School of Medicine, Toon, Ehime, Japan; Department of Molecular and Cellular Physiology, Ehime University Graduate School of Medicine, Toon, Ehime, Japan; Department of Neuropsychiatry, Molecules and Function, Ehime University Graduate School of Medicine, Shitsukawa, Toon, Ehime, Japan

**Keywords:** Gene expression, RNA-sequencing, schizophrenia, haloperidol, gene ontology, prenatal exposure

## Abstract

**Background:**

Schizophrenia is a mental disorder caused by both environmental and genetic factors. Prenatal exposure to antipsychotics, an environmental factor for the fetal brain, induces apoptotic neurodegeneration and cognitive impairment of offspring similar to schizophrenia. The aim was to investigate molecular biological changes in the fetal hippocampus exposed to haloperidol (HAL) by RNA expression as a model of the disorder.

**Methods:**

HAL (1 mg/kg/d) was administered to pregnant mice. Upregulated and downregulated gene expressions in the hippocampus of offspring were studied with RNA-sequencing and validated with the qPCR method, and micro-RNA (miR) regulating mRNA expressional changes was predicted by in silico analysis. An in vitro experiment was used to identify the miRNA using a dual-luciferase assay.

**Results:**

There were significant gene expressional changes (1370 upregulated and 1260 downregulated genes) in the HAL group compared with the control group on RNA-sequencing analysis (*P* < .05 and q < 0.05). Of them, the increase of *Nr3c1* mRNA expression was successfully validated, and in silico analysis predicted that microRNA-137-3p (miR-137-3p) possibly regulates that gene’s expression. The expression of miR-137-3p in the hippocampus of offspring was significantly decreased in the first generation, but it increased in the second generation. In vitro experiments with Neuro2a cells showed that miR-137-3p inversely regulated *Nr3c1* mRNA expression, which was upregulated in the HAL group.

**Conclusions:**

These findings will be key for understanding the impact of the molecular biological effects of antipsychotics on the fetal brain.

**Significance Statement**

Previous studies have shown that prenatal exposure to antipsychotics induces apoptotic neurodegeneration in the hippocampus and cognitive impairment of the offspring, which may then cause schizophrenia-like behavioral changes. Using pregnant mice exposed prenatally to haloperidol (HAL, 1 mg/kg/d), there were significant gene expressional changes, with 1370 upregulated and 1260 downregulated genes, compared with controls (*P *< .05 and q < 0.05) by RNA-seq of hippocampal RNA. GO analysis showed that synapse-related and neuron-related genes were prominent in both upregulated (563/1370 = 41.1%; >1.3-fold change) and downregulated (400/1260 = 31.7%; >1.3-fold change) genes. The expression of one microRNA, miR-137-3R, was increased in the HAL group, and it was confirmed that miR-137-3p regulates *Nr3c1* mRNA expression with an in vitro luciferase assay.

## Introduction

Schizophrenia (SCZ) is a chronic mental disorder, and lifetime antipsychotic medication for it is essential to prevent relapse (Japanese Society of Neuropsychopharmacology, 2021). It is also recommended that women with SCZ continue antipsychotics even during pregnancy, because stopping medication often leads to a relapse of the psychiatric condition and because antipsychotics have lower teratogenicity than other medications (Hasan et al., 2015). However, the exact impact of exposure to antipsychotics on the neural development of offspring has not been well studied. Numerous studies have investigated the pathogenesis of SCZ based on several hypotheses, including dopamine (Seeman and Lee, 1975) and glutamate hypotheses (Javitt and Zukin, 1991). However, no clear mechanism has been elucidated. Both environmental and genetic backgrounds are associated with the onset of SCZ (Gomes et al., 2019; Socrates et al., 2021).

MicroRNAs (miRNAs), which are noncoding RNAs approximately 20 nucleotides in length that are synthesized via several enzymatic processes, are an important component of the genetic background. A miRNA/miRNA* duplex forms, and 1 strand of the duplex loads into Argonaute homologue protein to form an RNA-induced silencing complex. Generally, such complexes work epigenetically as suppressors of mRNA expression (Yoshino and Dwivedi, 2020). MiR-137-3p was first identified in a genome-wide association study that reported the association between rs1625579 located in the miR-137 gene and SCZ onset (Schizophrenia Psychiatric Genome-Wide Association Study, 2011) and is a well-studied miRNA in SCZ research (Sakamoto and Crowley, 2018). Growing evidence has shown that miR-137-3p is associated with neurogenesis, synaptogenesis, and synaptic plasticity (Smrt et al., 2010; Szulwach et al., 2010; Siegert et al., 2015). It has also been consistently reported to regulate several genes relevant to neural functions (e.g., *BDNF*, *CACNA1C*, and *TCF4*) (Wright et al., 2013; Thomas et al., 2017). Prenatal stress, an environmental factor, is a risk factor for developing psychiatric disorders, including SCZ (Cattane et al., 2020; Krontira et al., 2020), and stress also regulates expression of several miRNAs in offspring (Liu et al., 2020a; Mazzelli et al., 2020; Labib, 2021). However, limited studies of miRNAs have been published.

Among environmental background factors, maternal conditions (e.g., immunological changes and drug exposure) possibly contribute to SCZ pathogenesis (Glass et al., 2019; Choudhury and Lennox, 2021). Wang et al. (H. Wang et al., 2019) reported that prenatal exposure to antipsychotics (haloperidol [HAL] and risperidone) disrupts the plasticity of dentate neurons and memory. Prenatal exposure to risperidone also induces fetal neurotoxicity in the hippocampal region and cognitive impairment (Singh and Singh, 2017). Moreover, prenatal exposure to quetiapine impacts apoptotic neurodegeneration in the fetal hippocampus and cognitive impairment (Singh and Tripathi, 2015).

The mechanism by which exposure of mothers to antipsychotics affects miRNA and mRNA expression in offspring is unknown. Thus, a prenatal HAL exposure mouse model was used to investigate (1) global expression changes by RNA-sequencing (RNA-seq) in the hippocampus of offspring; (2) the type of biological process based on differentially expressed genes (DEGs) using gene ontology (GO) analysis; (3) miRNAs relevant to global expressional changes according to in silico prediction; (4) the expression profiles of selected genes from RNA-seq, regulated by the miRNA; and (5) confirmation of the miRNA suppression of target genes using an in vitro system.

## Methods

**Figure 1.**
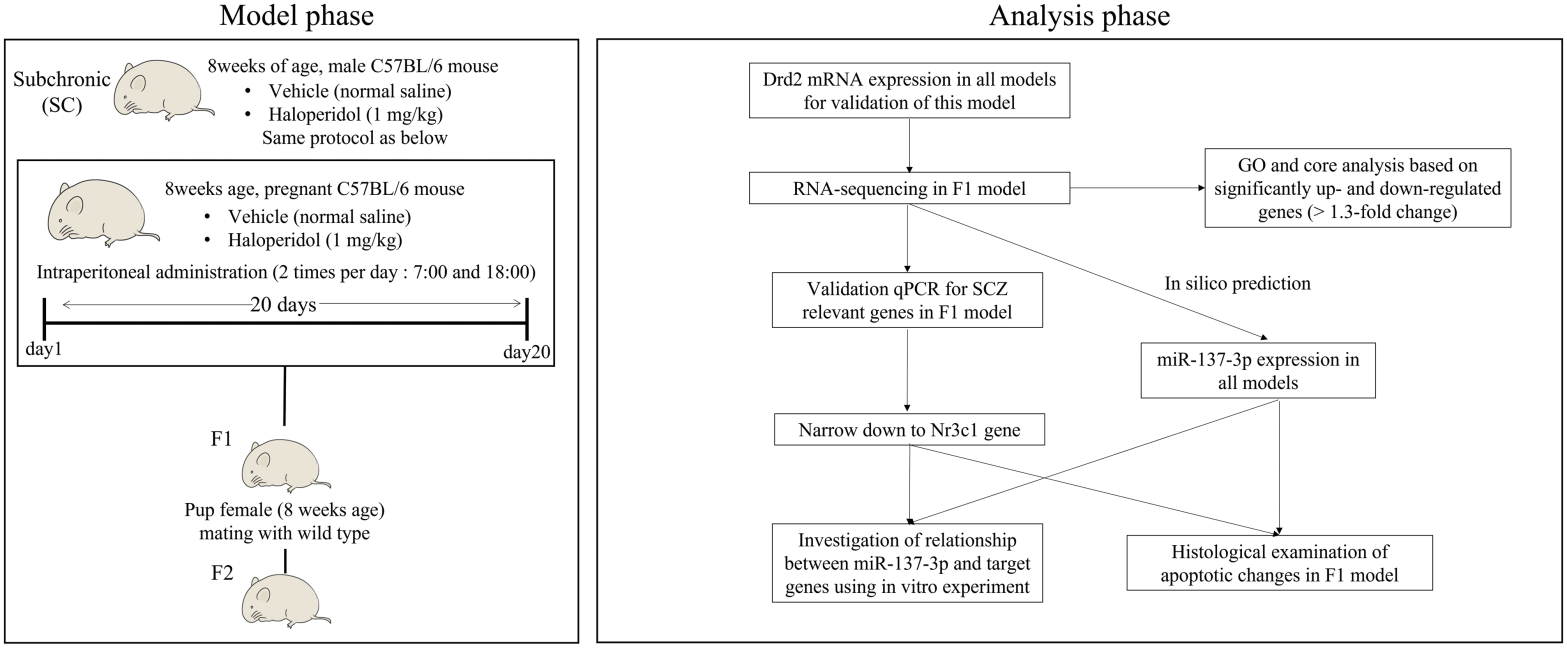
Schematic diagram of this study. The first generation of offspring was defined as the F1 model, and the second generation of offspring born from F1 females and wild-type male mice was defined as the second generation (F2). In addition, a subchronic model (SC) was created by haloperidol (HAL) administration to wild-type male mice (8 weeks old) for 20 days. With these samples, molecular biology and histological changes were investigated.

The outline of this study is shown in Figure 1.

### Animals

Day 0 pregnant C57BL/6 mice (8 weeks old) were purchased from CLEA Japan (Tokyo, Japan). Pregnancy was confirmed by checking for a vaginal plug. The first generation of offspring was defined as the F1 model, and the pups born from F1 females and male mice were defined as the F2 model. Male mice (8 weeks) for mating with F1 females and a subchronic model (SC) aged 8 weeks were also purchased from CLEA Japan (Tokyo, Japan). The pregnant mice were housed 1 per cage (temperature, 22°C ± 2°C) with free access to water and food with a 12-hour light/-dark cycle (lights on at 6:00 am). All experiments were conducted according to the Guidelines for Animal Experimentation of Ehime University Graduate School of Medicine (Ehime, Japan) and the ethics committee of Ehime University Graduate School of Medicine (No. 28-34).

### Drugs and Experimental Procedures

HAL and aripiprazole (ARP) were purchased from Tokyo Chemical Industry (Tokyo, Japan) and dissolved in 0.9% normal saline (NS) and 10% β-cyclodextrin (DEX) (Tokyo Chemical Industry), respectively. HAL and ARP were injected into pregnant females housed 1 per cage at 1 mg/kg/d (i.p., 2 times per day [7:00 am and 6:00 pm]), a dosing regimen capable of inducing side effects such as parkinsonism and catalepsy based on previous reports (Bilge and Erol, 2012; Nishchal et al., 2014; Sharma et al., 2018). Drug was administered from pregnancy day 1 to birth. Offspring (F1 and F2) were weaned at 4 postnatal weeks and housed 3 per cage after weaning. The SC mice were created by 1 mg/kg/d HAL or NS injection for 20 days from 8 weeks of age, and they were also housed 3 per cage. Only male mice were used for subsequent analyses. All mice were killed by decapitation, and tissue from the hippocampus was bilaterally dissected on an ice-cold stage, weighed, and stored at −80°C until analysis. This procedure was conducted according to the “Guidelines for the Care and Use of Mammals in Neuroscience and Behavioral Research (National Research Council (US) Committee on Guidelines for the Use of Animals in Neuroscience and Behavioral Research 2003).”

### RNA Isolation and Synthesis of Complementary DNA (cDNA)

RNA was isolated using TRIzol (Thermo Fisher Scientific, Carlsbad, CA, USA) according to the manufacturer’s protocol (Roy et al., 2017). RNA quality and concentration were tested using the NanoDrop 1000 system (Thermo Fisher Scientific, Yokohama, Japan). RNA samples with a 260/280 ratio between 1.8 and 2.0 were considered pure.

### **Synthesis of cDNA for mRNA-Specific Gene Expression and Quantitative PCR** (**qPCR)**

RNA (1.0 µg) was used in 40-µL reaction mixtures to synthesize cDNA using the High-Capacity cDNA Reverse Transcription Kit (Applied Biosystems, Foster City, CA, USA). Reverse transcription (RT)-qPCR was used to measure mRNA expression levels by the StepOnePlus Real-Time PCR System (Applied Biosystems). The Predesigned qPCR Assay used Mm.PT.58.7811767 for *Drd2*, Mm.PT.58.12183825 for *Htr2c*, Mm.PT.58.41280327 for *Gsk3b*, Mm.PT.58.33547773 for *Nrxn1*, Hs.PT.58.42952901 for *Nr3c1*, and Mm.PT.39a.1 for *Gapdh*. RT-qPCR was conducted by using the PrimeTime Gene Expression Master Mix (Integrated DNA Technologies, Inc., Coralville, IA, USA) in a final volume of 10 µL. The mRNA expression levels were tested in duplicate, and outliers were removed according to the Outlier Calculator (https://miniwebtool.com/outlier-calculator/). The relative expression value was calculated using Livak’s ΔΔCt method (Livak and Schmittgen, 2001).

### Synthesis of cDNA for miRNA-Specific Gene Expression and qPCR Method

RNA (0.25–8.0 μg; 3.75 μL) was used in a 5-μL reaction mixture to synthesize cDNA for miRNA using the Mir-X miRNA First-Strand Synthesis Kit (Takara Bio Inc., Tokyo, Japan). Subsequently, the cDNA for miRNA was diluted 10 times. The miRNA expression levels were measured in duplicate using the Mir-X miRNA qRT-PCR TB Green Kit (Takara Bio Inc.) and StepOnePlus Real-Time PCR System (Applied Biosystems) with miRNA-specific primers (miR-137-3p: 5’-TTATTGCTTAAGAATACGCGTAG-3’) according to the manufacturer’s protocol. Briefly, the thermal cycling conditions were initial denaturation for 10 seconds at 95℃ and 35 subsequent cycles of denaturation for 5 seconds at 95℃, annealing and elongation for 20 seconds at 60℃, and a dissociation curve step (60 seconds at 95℃, 30 seconds at 55℃, and 30 seconds at 95℃). U6 included in the Mir-X miRNA qRT-PCR TB Green Kit was used as an internal standard. Outliers were removed according to the Outlier Calculator (https://miniwebtool.com/outlier-calculator/). The relative expression value was calculated using Livak’s ΔΔCt method (Livak and Schmittgen, 2001).

### RNA Sequencing

RNA from F1 mice (NS vs HAL; n = 6 each) was subjected to RNA-seq. The RNA integrity number was calculated by an Agilent 2100 Bioanalyzer (Agilent Technologies Inc., Santa Clara, CA, USA) and an Agilent RNA 6000 Nano Kit (Agilent Technologies Inc.) (supplementary Table 1). Subsequently, 200 ng total RNA from each sample was used for RNA-seq library preparation. An RNA-seq library of each sample was created using the Illumina TruSeq Stranded mRNA Sample Prep Kit (Illumina, Indianapolis, IN, USA) according to the manufacturer’s protocol. The quality of the average size (340–380 bp) was validated using an Agilent DNA1000 kit and Agilent 2100 Bioanalyzer (Agilent Technologies Inc.). The amount was determined using qPCR with the Kapa Library Quantification Kit (Illumina). The MiSeq Reagent kit V3 on a MiSeq system was used for sequencing (Illumina) based on pair-end reads (75 bp) according to the manufacturer’s instructions. The running cycle was set to 150 cycles.

### Bioinformatic Analysis for RNA-Seq Data

Raw data files (FASTQ format) were extracted from the MiSeq system (Illumina). The reads were aligned with the reference genome (mm10) using TopHat software (Trapnell et al., 2009). Cufflinks was used for estimating expression levels (metric fragments per kilobase of transcript per million mapped reads [FPKM value]) of the known genes (Trapnell et al., 2010). The criterion for abundantly expressed genes per group was set as average FPKM values ≥1.0 in each group. Genes including an FPKM value of 0 in either the NS or F1 group were excluded. DEG analysis was performed using the edgeR package (Robinson et al., 2010). Significant DEGs were defined as *P* < .05 and q < 0.05. DEseq2 was used to create a volcano plot (Love et al., 2014). A heatmap based on significantly changed DEGs was generated using heatmap.2.

### Functional Annotation

ClueGO plugin (Bindea et al., 2009) in the Cytoscape program (ver. 3.8.0) was used to perform GO of biological process (BP), molecular function (MF), and cellular component (CC). Statistical significance was set at *P* < .05 with the post-hoc Benjamini–Hochberg method. Functional connectivities of significant GO terms were generated to a graphical network using GO term fusion based on the following criteria: visual style = groups; GO term/pathway network connectivity = medium (kappa score = 0.50).

### Core Analysis (Canonical Pathway, Diseases, and Functions)

A module for the functional enrichment of target genes and their functional roles were considered as canonical and disease pathways using Fisher’s exact test (*P* value threshold set at <.05) by Ingenuity Pathway Analysis software (Qiagen, Valencia, CA, USA).

### Prediction of miRNAs Associated With DEGs in RNA-seq and Target Genes of miRNA

MiRNAs relevant to significant DEGs on RNA-seq were predicted by Ingenuity Pathway Analysis software (Qiagen) with the miRNA Target Filter function with the criteria of “experimentally determined” or “high confidence” downstream targets of the miRNAs.

An in silico prediction algorithm for 8 prediction programs (miRWalk, Microt4, miRanda, miRDB, Pictar2, PITA, RNA22, and Targetscan under miRWalk version 2.0 software package) was used for target gene prediction. Next, genes that were present in >6 of them were defined as predicted target genes.

### In Vitro Cell Line-Based Studies

Neuro2a neuroblastoma cells were purchased from KAC (EC89121404-F0, Kyoto, Japan) and cultured in Dulbecco′s Modified Eagle′s Medium/Nutrient Mixture F-12 Ham (DMEM/F-12) containing 10% fetal bovine serum and penicillin and streptomycin (10 000 U/mL). The cells were incubated at 37°C in a 5% CO_2_ atmosphere, and the medium was replaced every 24 hours. Double-stranded RNA oligos (mmu-miR-137-3p mimic [C-310413-05-0002] and mmu-miR-137-3p hairpin inhibitor [IH-31043-07-0002]) were used (Horizon Discovery Group, Cambridge, UK). RNA oligos were transfected into Neuro2a cells using Lipofectamine RNAiMAX (Invitrogen, Grand Island, NY, USA) according to the manufacturer’s protocol and harvested 48 hours posttransfection for target gene expression analysis. This study was replicated in 2 independent batches.

### Dual Luciferase Reporter Assay

The seed and mutant sequence of Nr3c1 gene was predicted using TargetScanMouse (http://www.targetscan.org/mmu_80/) and cloned into a pmirGLO vector (Promega Corporation, Madison, WI, USA). The pmirGLO was digested by *Pme*I (New England Biolabs, Ipswich, MA, USA) and *Xba*I (New England Biolabs) following the manufacturer’s protocol. The sequences of seed and mutant oligos are shown in supplementary Table 2, and they were annealed by annealing buffer (10 mM Tris-HCl pH 7.5, 1 mM ethylenediaminetetraacetic acid, and 10 mM MgCl_2_) with the following conditions: 68°C for 2 minutes, 37°C for 10 minutes, and incubated at room temperature for 5 minutes. Digested pmirGLO vector and annealed seed or mutant oligos were ligated with Ligation-Convenience Kit:Nippon Gene (Tokyo, Japan). The plasmid was subjected to transformation of the *E. coli* DH5α, and the sequence of the inserted DNA in the recombinant plasmid obtained was confirmed by Sanger sequencing with sequence primer as shown in supplementary Table 2. For the luciferase reporter assay, luciferase reporter vectors and mmu-miR-137-3p mimic were cotransfected into Neuro2a cells using Lipofectamine 3000 (Invitrogen) according to the manufacturer’s protocol and harvested 48 hours posttransfection (n = 3 in each group).

### TUNEL Staining

Mice (8 weeks of age) were decapitated, and brains were removed and fixed in 4% paraformaldehyde in 0.1 M phosphate buffer (pH 7.4). Specimens were dehydrated in a graded series of ethanol, infiltrated in xylene, and embedded in paraffin wax. The brain was processed routinely for paraffin sectioning. Sections were cut at 7 μm, dewaxed in xylene, and rehydrated with a graded series of ethanol. The sections were assayed using the terminal deoxynucleotidyl transferase-mediated dUTP-biotin nick end labeling (TUNEL) method using an apoptosis in situ detection kit (FUJIFILM Wako Pure Chemical Industries, Osaka, Japan).

### Immunohistochemistry

Deparaffinized sections were incubated in a Phosphate-buffered saline (PBS) solution including 0.005% hydrogen peroxide for 20 minutes to suppress endogenous peroxidase activity. After a brief wash in PBS, the sections were exposed to 3% normal goat serum (Jackson ImmunoResearch Laboratories, West Grove, PA, USA) and 5% bovine serum albumin in PBS and then rinsed in PBS. The sections were processed for immunohistochemistry, mainly with the caspase 3 antibody (Bioss antibodies, Woburn, MA, USA) as the primary antibody at a dilution of 5 µg/mL in PBS with 5% bovine serum albumin. The antigen-antibody complexes were visualized using a VECTASTAIN ABC and substrate kit (Vector Laboratories, Burlingame, CA, USA).

### Statistical Analysis

SPSS 22.0 software (IBM Japan, Tokyo, Japan) was used for the statistical analysis. Assessment of normal distribution was performed using the Shapiro–Wilk test. Average differences in mRNA and miRNA levels between 2 groups were assessed using the Student’s *t* test or the Mann–Whitney *U* test, and the average differences in 3 or more groups were assessed by 1-way ANOVA with the post-hoc Tukey test or the Kruskal-Wallis test with the post-hoc Steel-Dwass test. Statistical significance was defined at the 95% level (*P* = .05).

## Results

### Investigation of Pharmacological Effect of HAL on Offspring Mice

Drd2 mRNA expression was measured to elucidate the direct effect of HAL, because the main pharmacological effect of HAL is as an antagonist of the Drd2 receptor. The number of mice in each model was as follows: NS vs HAL (SC, 8 vs 8: F1, 8 vs 6: F2, 8 vs 8) and NS vs DEX vs ARP (F1, 8 vs 8 vs 5). Drd2 expression was significantly increased in the HAL group compared with the NS group in F1 (NS vs HAL: 1.0 ± 0.37 vs 1.47 ± 0.30, *P* = .035) but not in SC (NS vs HAL: 1.0 ± 0.54 vs 0.81 ± 0.12, *P* = .959) or F2 (NS vs HAL: 1.0 ± 0.27 vs 0.85 ± 0.18, *P* = .206) mice, as shown in supplementary Figure 1. In terms of ARP treatment, there were no significant changes (NS vs DEX vs ARP: 1.0 ± 0.10 vs 0.97 ± 0.08 vs 0.99 ± 0.08, *P* = .752; supplementary Figure 2A).

### MiR-137-3p Expression

**Figure 2.**
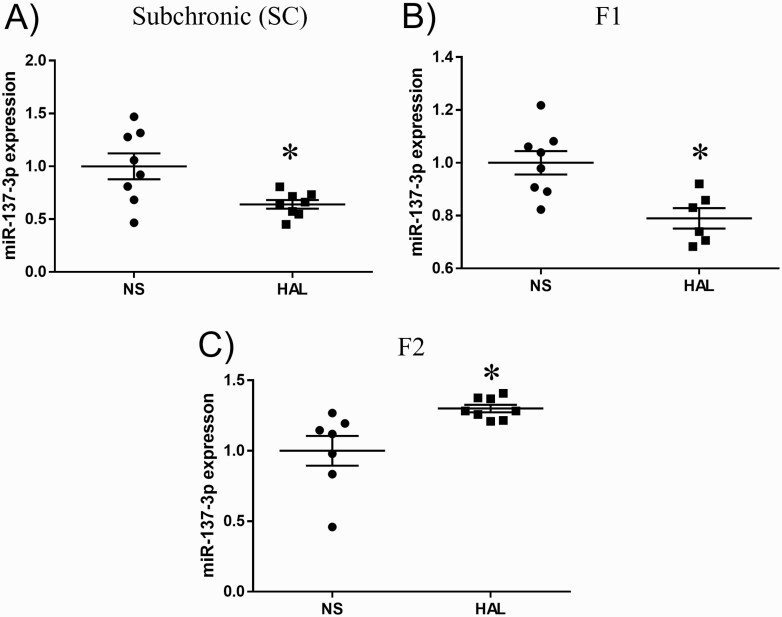
MicroRNA (MiR)-137-3p expressions in all models. The y-axis represents the ratio of relative expression values of the normal saline (NS) and haloperidol (HAL) groups. miR-137-3p expression was measured by the quantitative PCR (qPCR) method in (A) subchronic, (B) first-generation (F1), and (C) second-generation (F2) mice. *P < .05.

MiR-137-3p expression was significantly decreased in the HAL group compared with the NS group in SC (NS vs HAL: 1.0 ± 0.34 vs 0.64 ± 0.11, *P* = .022; Figure 2A) and F1 (NS vs HAL: 1.0 ± 0.13 vs 0.79 ± 0.09, *P* = .005; Figure 2B) mice. On the other hand, significantly increased miR-137-3p expression was found in the HAL group of F2 offspring (NS vs HAL: 1.0 ± 0.28 vs 1.30 ± 0.08, *P* = .029; Figure 2C).

### RNA-Seq

A total 12 941 genes were identified using the stringent criteria described in the Methods; these genes are depicted in the volcano plot in supplementary Figure 3A. Of those genes, 1370 upregulated and 1260 downregulated genes were detected in the HAL group compared with the NS group (*P* < .05 and q < 0.05). A heatmap of significant DEGs is shown in supplementary Figure 3B. All significant DEGs are shown in supplementary Table 3.

### Functional Annotation and Core Analysis of RNA-Seq Data

**Figure 3.**
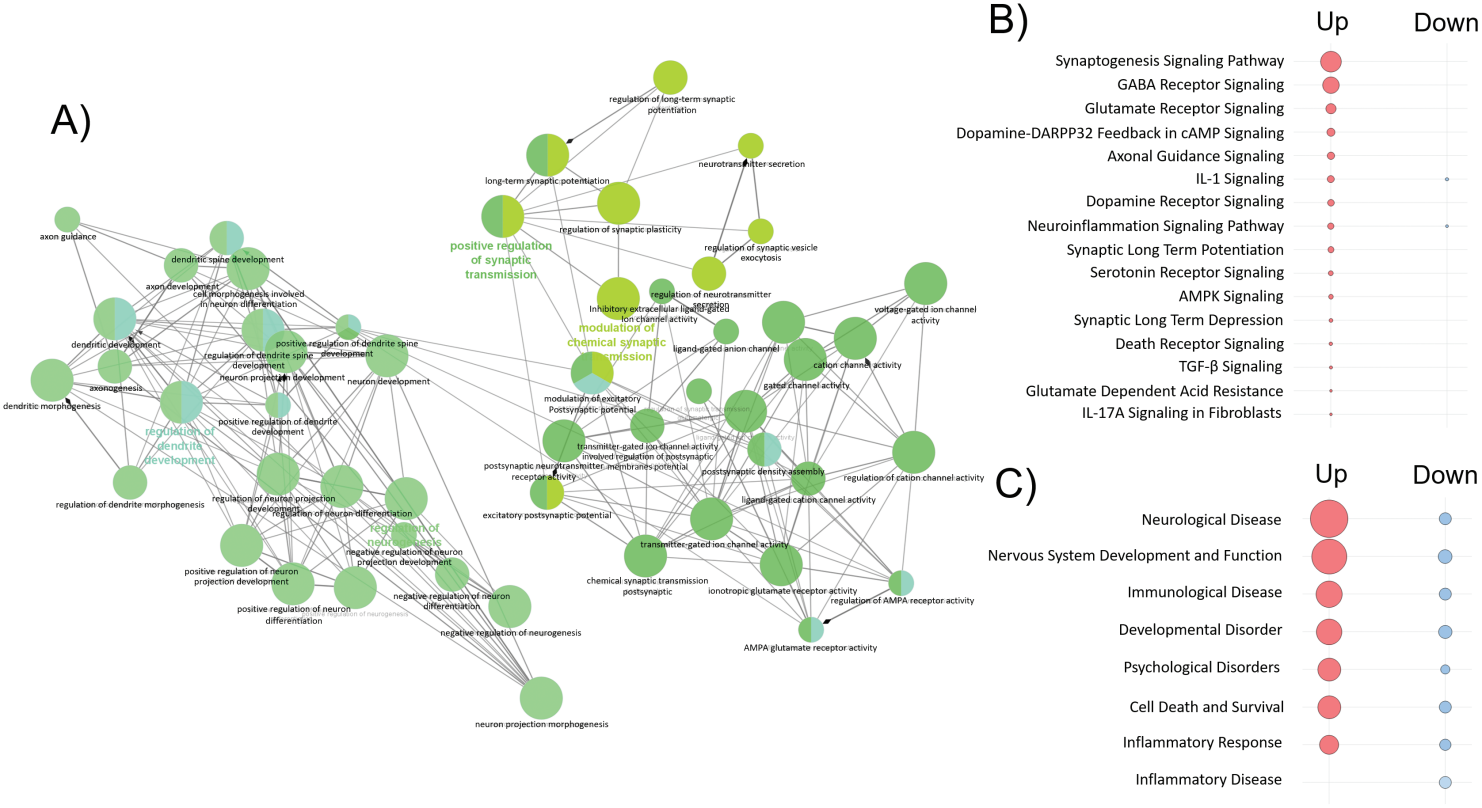
Synapse-related and neuron-related biological process (BP) results and core analysis based on RNA-sequencing data. (A) The network was constructed with neuron-related BP terms based on 563 upregulated genes (*P* < .05, q < 0.05, and >1.3-fold change), which were more abundant than 400 downregulated genes (*P* < .05, q < 0.05, and >1.3-fold change) using ClueGo in the Cytoscape program. Canonical pathways, disease, and function were analyzed using 563 upregulated and 400 downregulated genes (*P* < .05, q < 0.05, and >1.3-fold change). Significant results for the canonical pathway (B) and disease and function (C) are shown in red (up) and blue (down) circles. Spot/circle size is the function of –log(base = 10) of Fisher’s exact test enrichment *P* value.

A total 563 upregulated genes (>1.3-fold change) were subjected to analysis, and 87 BP terms reached significant levels (supplementary Table 4). Synapse and neuron-related BP terms were abundant and connected to each other (Figure 3A). Twelve CC and 14 MF terms reached significance. Interestingly, 4 synapse membrane-related CC terms were the most significant (e.g., postsynaptic density membrane, integral component of postsynaptic membrane, integral component of postsynaptic specialization membrane, integral component of postsynaptic density membrane). For the 400 downregulated genes (>1.3-fold change), 11 BP terms, 9 CC terms, and 1 MF term were significant (supplementary Table 5). When considering canonical pathways, the synaptogenesis signaling pathway was the most significant in terms of upregulated genes, but not in downregulated genes (Figure 3B). Several neurotransmitter receptor signaling genes (e.g., receptors for GABA, glutamine, dopamine, serotonin) were also significant among upregulated genes, but not downregulated genes. On the other hand, CNS-related terms such as neurological disease and psychological disease were found both among upregulated and downregulated genes (Figure 3C).

### In Silico Prediction of mIR-137-3p Target Genes and qPCR Validation in the in Vivo Study

**Figure 4.**
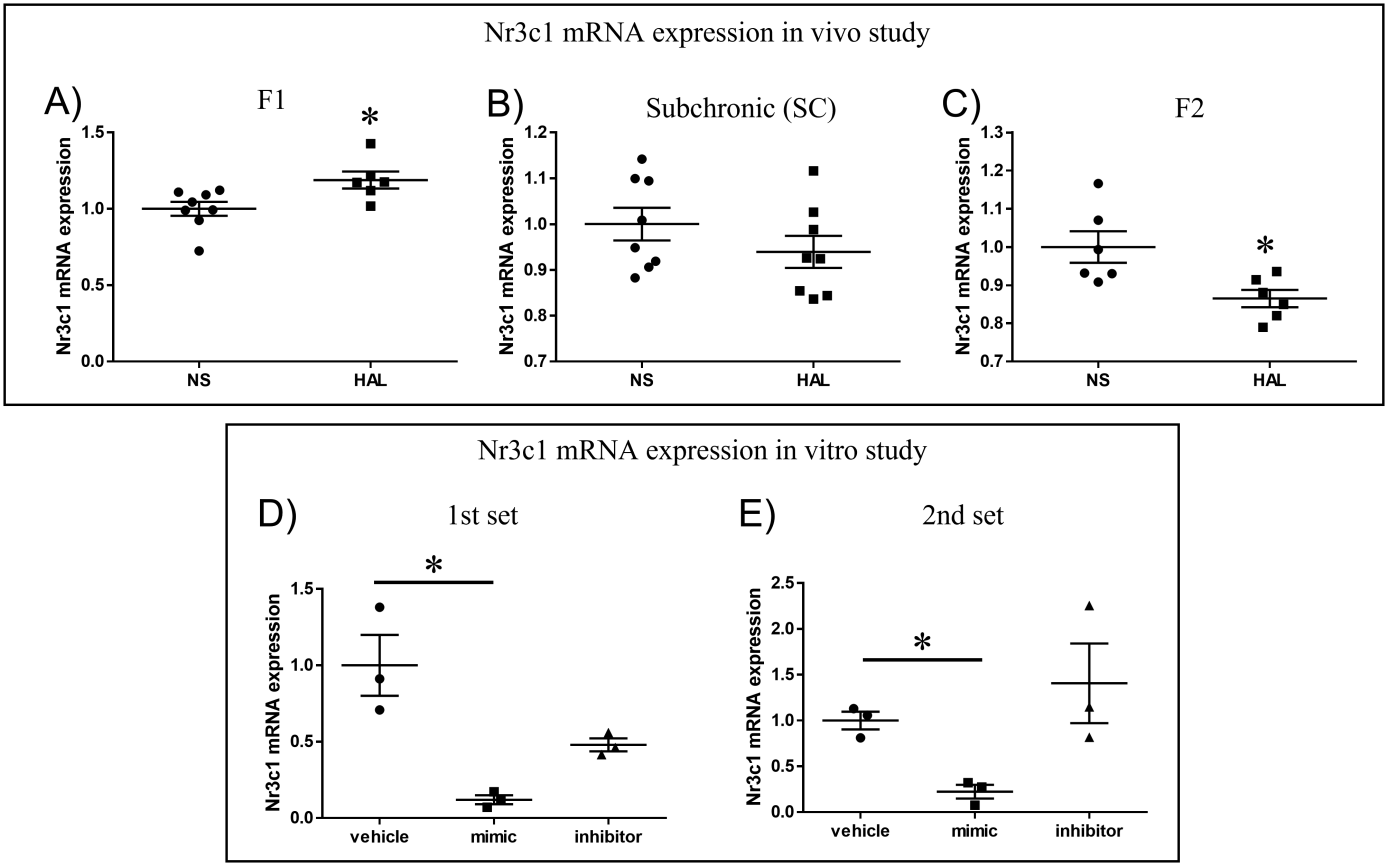
Nr3c1 mRNA expression from in vivo and in vitro studies. The y-axis represents the ratio of relative expression values in the normal saline (NS) and haloperidol (HAL) groups. The validation tests were conducted by the quantitative PCR (qPCR) method of samples from F1 offspring (A). The expressional changes in *Nr3c1* were also confirmed in subchronic model (SC) (B) and F2 offspring (C). *Nr3c1* expression was also confirmed with the qPCR method in both the first set (D) and second set (E). *P < .05.

Based on the results of functional annotation and core analysis in the RNA-seq data, we focused on the 1370 upregulated genes because they were more relevant to neuronal functions than the 1260 downregulated genes. Subsequently, we narrowed down miR-137-3p from the list of miRNAs relevant to upregulated DEGs (supplementary Table 6) because miR-137-3p is well-studied in the SCZ field. Decreased miR-137-3p expression was found in the HAL group of F1 offspring. As a result of in silico prediction of miR-137-3p, 445 genes were considered to be predicted target genes based on our criteria (supplementary Table 7). Subsequently, 4 genes (*Htr2c*, *Gsk3b*, *Nrxn1*, and *Nr3c1*) were selected for qPCR validation according to the following criteria: (1) upregulated genes on RNA-seq; (2) predicted target genes of miR-137-3p; and (3) relevant to neuronal function or psychiatric disorders. qPCR in samples from F1 offspring validated *Nr3c1* mRNA expression (NS vs HAL: 1.0 ± 0.13 vs 1.19 ± 0.14, *P* = .022; Figure 4A), but the other 3 genes were unchanged (*Htr2c*, *P* = .181; *Gsk3b*, *P* = .755; and *Nrxn1*, *P* = .182). Furthermore, decreased *Nr3c1* expression was found in the HAL group of F2 offspring (1.0 ± 0.10 vs 0.87 ± 0.06, *P* = .016; Figure 4C) but not in SC mice (1.0 ± 0.10 vs 0.94 ± 0.10, *P* = .244; Figure 4B). In terms of ARP treatment, there were significant changes between the NS and DEX groups but not the DEX and ARP groups (NS vs DEX vs ARP: 1.0 ± 0.06 vs 1.11 ± 0.08 vs 1.03 ± 0.13, *P* = .045; supplementary Figure 2B).

### In Vitro Cell Line-Based Studies

When Neuro2a cells were transfected with the 20-nM miR-137-3p oligo, 40 times and 20 times upregulation of miR-137-3p expression was seen in the mimic compared with the vehicle in the first and second set, respectively (supplementary Figure 4). Nr3c1 and Gsk3b mRNA expressions were investigated because *Htr2c* and *Nrxn1* mRNA expressions were not confirmed in the Neuro2a cell line. Significant downregulation was found for *Nr3c1* expression in the first set (12%, *P* = .006; Figure 4D) and second set (22%, *P* = .047; Figure 4E). No significant changes were seen for *Gsk3b* in either the first (*P* = .059) or second set (*P* = .708).

### Dual Luciferase Reporter Assay in the in Vitro Study

**Figure 5.**
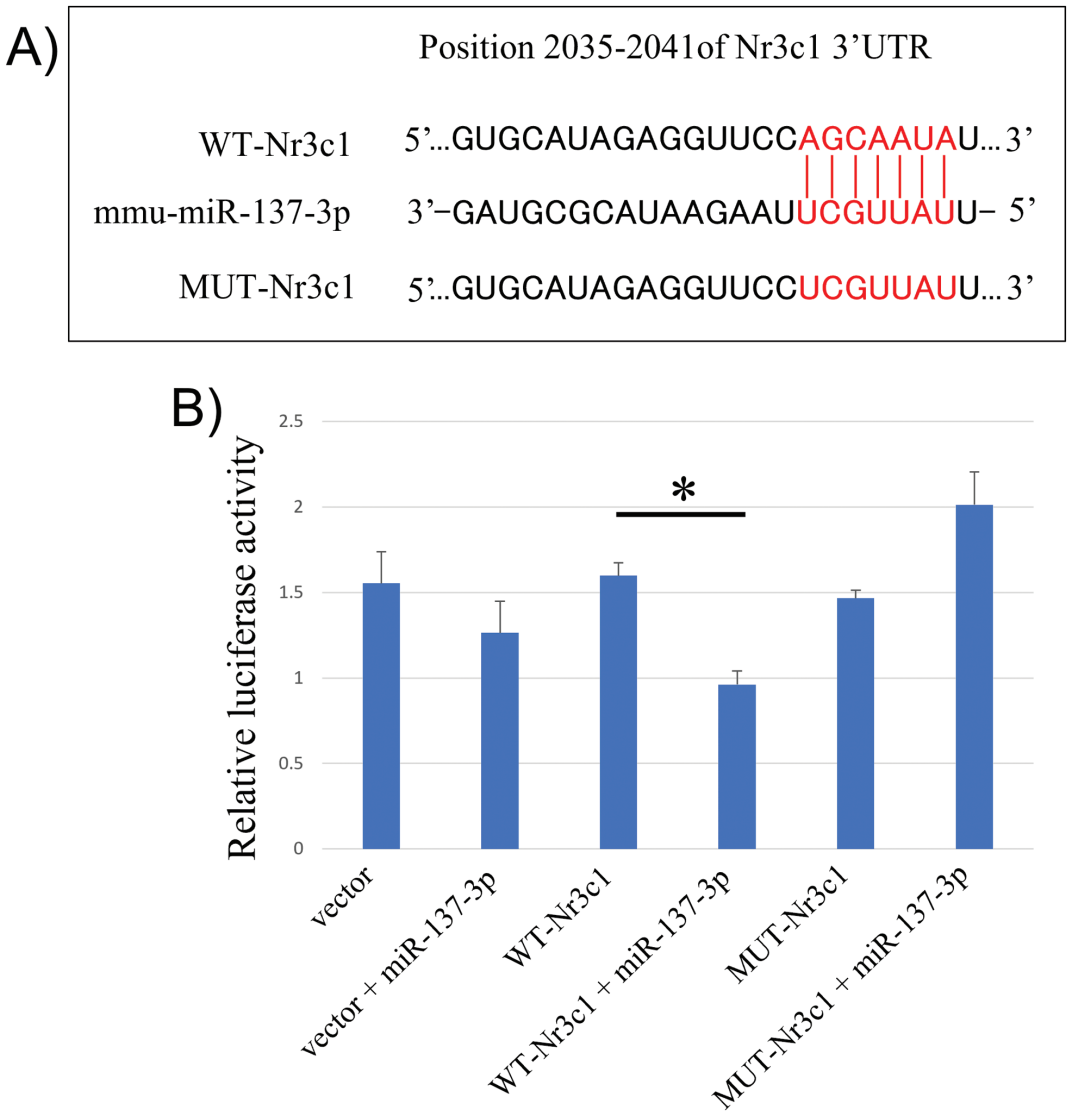
In silico prediction of seed sequence in the Nr3c1 gene and luciferase assay. (A) Bioinformatics predicated the interaction between seed sequences of the Nr3c1 gene in 3’-untranslated region (UTR) and mmu-miR-137-3p. (B) Relative luciferase activity was decreased in neuro2a cells co-transfected both wild-type-Nr3c1 of Nr3c1 cloned pmirGLO vector and mmu-miR-137-3p (n = 3 in each group). *P < .05. Abbreviations: miR, microRNA; MUT, mutant.

As a result of in silico prediction, there were 2 possible seed sequences of mmu-miR-137-3p (AGCAAUA and GCAAUAA). The AGCAAUA sequence was selected for the dual luciferase reporter assay because the sequence is preserved in humans, whereas the GCAAUAA sequence is not (Figure 5A). When measuring the relative luciferase assay in pmirGLO vectors and 20-nM miR-137-3p co-transfected Neuro2a cells, there was a significant change (F = 6.188, *P* = .005; Figure 5B).

### TUNEL Staining and Immunohistochemistry to Assess Apoptosis

Differences were not observed in the TUNEL and caspase 3-positive reactions between sections from HAL-treated and control mice (supplementary Figure 5).

## Discussion

To the best of our knowledge, this is the first study to investigate miR-137-3p and global gene expression in offspring from mothers exposed to HAL. Based on the RNA-seq data, miR-137-3p, which is well-studied in the SCZ field, was selected from the list of miRNAs relevant to upregulated DEGs; miR-137-3p expression was downregulated and upregulated in F1 and F2 offspring, respectively. From the GO and core analyses with upregulated DEGs in F1 offspring, DEGs were relevant to synapse and neuronal functions. Of the DEGs, *Nr3c1* mRNA expression was successfully validated in F1 offspring using the qPCR method, and downregulation of *Nr3c1* mRNA was found in F2 offspring. Using an in vitro experiment, miR-137-3p was found to inversely regulate *Nr3c1* mRNA expression by binding the 3’-UTR seed sequence of *Nr3c1*.

*Drd2* mRNA expression was upregulated in F1 offspring from mothers exposed to HAL. When considering the unchanged *Drd2* expression in SC and F2 offspring, HAL affected fetuses by crossing the placenta. Indeed, placental passage of HAL was reported in a human study (Newport et al., 2007). Furthermore, the side effects of antipsychotics such as increased birth weight, abnormal neuromotor performance, and glucose tolerance were found in human offspring (Boden et al., 2012; Johnson et al., 2012; Frayne et al., 2018) and rodent studies (Courty et al., 2018). Interestingly, the opposite results for miR-137-3p expression (upregulation in AC and F1 offspring, and downregulation in F2 offspring) were found. Previous studies have also reported that HAL treatment induces expression changes in several miRNAs (Santarelli et al., 2013; Swathy and Banerjee, 2017; P. Wang et al., 2019). The same downregulation of miR-137-3p expression in AC and F1 offspring was directly affected by HAL treatment. In addition, a mouse study showed that administration of olanzapine to mothers induces decreased body weight in F1 offspring and increased body weight in F2 offspring (Courty et al., 2018). Taken together, opposite results at the molecular biological level between F1 and F2 offspring are understandable.

From the RNA-seq results, upregulated DEGs were more relevant to synaptic and neural functions and several neurotransmitters than downregulated DEGs. Of those, we focused on *Nr3c1*, which encodes a glucocorticoid receptor, and its upregulated expression was successfully validated in F1 offspring. Elevated glucocorticoids in utero may induce neurodevelopmental alterations in offspring through the activity of the fetal hypothalamic–pituitary–adrenal axis (Krontira et al., 2020). Indeed, DNA methylation and mRNA expression changes of *Nr3c1* have been reported in SCZ patients (Sinclair et al., 2012a; Sinclair et al., 2012b; Liu et al., 2020b). Other than downregulated *Nr3c1*, upregulation was found in 3 genes (*Clock*, *Ncoa2*, *Ntrk2*) on RNA-seq, which are also relevant to the glucocorticoid receptor signaling pathway (GO:0042921). The dysregulation of the glucocorticoid receptor signaling pathway is reported as an interaction with miR-137 (Valles et al., 2014). Using an in vitro experiment, miR-137-3p was found to inversely regulate Nr3c1 mRNA expression. Moreover, the luciferase activity assay showed that miR-137-3p regulated Nr3c1 mRNA expression through binding the predicted seed sequence in 3’-UTR of the *Nr3c1* gene. Furthermore, inverse expression between miR-137-3p and *Nr3c1* was found in F1 and F2 offspring. When considering the function of miRNAs as suppressors of mRNA expression, the miR-137 downregulation contributes to upregulation of Nr3c1 (and other targets) in the HAL group.

Growing evidence has shown that miR-137-3p modulates neuronal apoptosis both in rats and in vitro (Wang et al., 2020) and apoptosis in hippocampal neural stem cells in mice (Schouten et al., 2015). In addition, apoptotic neurodegeneration was found in the hippocampus of rat offspring from mothers exposed to antipsychotics (Singh and Tripathi, 2015; Singh et al., 2016). Thus, we speculated that apoptotic changes occur in F1 offspring due to changes in the expression of miRNAs like miR-137-3p, but apoptotic changes were not found in our experiments. Apoptotic changes may need to be assessed in older mice because only 8-week-old offspring were used.

This study has several limitations. First, animal studies cannot be applied directly to humans. In addition, the recommendations of pharmacotherapy for mothers with SCZ should not be affected by the present results. However, the present results indicate that offspring exposed to antipsychotics during gestation should be followed-up carefully in terms of not only teratogenicity but also other neurodevelopmental abnormalities. Second, behavioral tests were not conducted even though behavioral abnormalities, including cognitive dysfunction, were found in previous reports (Singh and Tripathi, 2015; Singh and Singh, 2017; Wang et al., H. 2019). In the future, replication studies using the same protocol should be conducted and include behavioral tests.

In conclusion, it was confirmed that prenatal HAL exposure induces global gene expressional changes relevant to synaptic and neural functions. Of those, *Nr3c1* mRNA expression was upregulated, followed by the downregulation of miR-137-3p, and this inverse expression was clarified in an in vitro experiment. These findings will be key to understanding the molecular biological mechanism of the effect of antipsychotics in the fetal brain.

## Supplementary Material

pyac044_suppl_Supplementary_Material
