# Supplementary material for: Impact of Gestational Haloperidol Exposure on miR-137-3p and Nr3c1 mRNA Expression in Hippocampus of Offspring Mice
Source: Int J Neuropsychopharmacol. 2022 Jul 21;25(10):853–62. doi: 10.1093/ijnp/pyac044 (PMC9593222; doi:10.1093/ijnp/pyac044)
Supplement: pyac044_suppl_Supplementary_Material [file pyac044_suppl_supplementary_material.docx]

Impact of gestational haloperidol exposure on miR-137-3p and Nr3c1 mRNA expression in the hippocampus of offspring mice

Yuta Yoshino M.D., PhD^1^, Hiroshi Kumon M.D.^1^, Tetsuya Shimokawa D.V.M., PhD^2^, Hajime Yano Ph.D.^3^, Shinichiro Ochi M.D., PhD ^1^, Yu Funahashi M.D.^1^, Jun-ichi Iga M.D., PhD *^1^, Seiji Matsuda M.D., PhD ^2^, Junya Tanaka M.D., PhD ^3^, Shu-ichi Ueno M.D., PhD ^1^

1. Department of Neuropsychiatry, Molecules and Function, Ehime University Graduate School of Medicine, Shitsukawa, Toon, Ehime 791-0295, Japan

2. Department of Anatomy and Embryology, Ehime University Graduate School of Medicine, Toon, Ehime 791-0295, Japan

3. Department of Molecular and Cellular Physiology, Ehime University Graduate School of Medicine, Toon, Ehime 791-0295, Japan.

Running title: Impact of gestational haloperidol exposure on miR-137-3p

*Corresponding author: Jun-ichi Iga

Department of Neuropsychiatry, Molecules and Function

Ehime University Graduate School of Medicine

Shitsukawa, Toon, Ehime 791-0295, Japan

Tel: +81-89-960-5315, Fax: +81-89-960-5317

E-mail: igajunichi@hotmail.com

**Table S1. RIN values in NS and HAL samples for RNA-sequencing**

| **Samples** | **RIN** |
| --- | --- |
| NS1 | 7.9 |
| NS2 | 7.5 |
| NS3 | 7.8 |
| NS4 | 7.2 |
| NS5 | 7.3 |
| NS6 | 7.3 |
| HAL1 | 8.4 |
| HAL2 | 7.9 |
| HAL3 | 7.9 |
| HAL4 | 7.5 |
| HAL5 | 7.9 |
| HAL6 | 7.6 |

HAL, haloperidol; NS, normal saline; RIN, RNA Integrity Number

**Table S2. The sequence of seed and mutant oligos and pmirGLO sequence primer**

|  |  | Sequence |
| --- | --- | --- |
| Seed | sense | 5’-AAAATAGCGGCCGCTAGTAGAGGTACCAGCAATATGTAAATAT-3’ |
|  | antisense | 5’-CTAGATATTTACATATTGCTGGTACCTCTACTAGCGGCCGCTATTTT-3’ |
| Mutant | sense | 5’-AAAATAGCGGCCGCTAGTAGAGGTACCTCGTTATTGTAAATAT-3’ |
|  | antisense | 5’-CTAGATATTTACAATAACGAGGTACCTCTACTAGCGGCCGCTATTTT-3’ |
| Sequence primer | | 5’-TTACAACCGCCAAGAAGCTG-3’ |

**Table S3. All significantly up- and downregulated genes for RNA-sequencing of F1**

| **Gene symbol** | **Locus** | **NS FPKM average value** | **HAL FPKM average value** | **Fold change** | **p value** | **q value** |
| --- | --- | --- | --- | --- | --- | --- |
| **Upregulated genes** | | | | | | |
| Rxfp1 | chr3:79644715-79737794 | 1.15093 | 4.10447 | 3.56622 | 5.00E-05 | 0.000607 |
| Rims3 | chr4:120877868-120891560 | 1.96122 | 5.17217 | 2.637221 | 0.0009 | 0.006615 |
| Fmod | chr1:134037514-134048277 | 2.02337 | 5.1285 | 2.534633 | 5.00E-05 | 0.000607 |
| Hdac4 | chr1:91932748-92148393 | 1.5133 | 3.6886 | 2.437455 | 5.00E-05 | 0.000607 |
| Lgi2 | chr5:52537863-52566280 | 2.12049 | 4.89094 | 2.306514 | 5.00E-05 | 0.000607 |
| Nos1 | chr5:117867118-117953813 | 2.51219 | 5.78213 | 2.301629 | 5.00E-05 | 0.000607 |
| Uhmk1 | chr1:170199255-170215393 | 4.12927 | 9.1514 | 2.216227 | 5.00E-05 | 0.000607 |
| Irs2 | chr8:10986963-11054541 | 2.18741 | 4.82365 | 2.205188 | 5.00E-05 | 0.000607 |
| Fosb | chr7:19302695-19310045 | 1.85938 | 4.00795 | 2.15553 | 5.00E-05 | 0.000607 |
| Clvs1 | chr4:9269316-9669162 | 2.38282 | 5.10552 | 2.142638 | 0.0034 | 0.018393 |
| Hint3 | chr10:30608206-30618366 | 4.12659 | 8.73783 | 2.117446 | 0.0011 | 0.007621 |
| Nr4a2 | chr2:57107225-57124003 | 9.30033 | 19.679 | 2.115946 | 5.00E-05 | 0.000607 |
| Necab1 | chr4:14930640-15149131 | 8.75898 | 18.1855 | 2.076212 | 5.00E-05 | 0.000607 |
| Cdh13 | chr8:118283754-119323448 | 9.24132 | 19.0054 | 2.056568 | 5.00E-05 | 0.000607 |
| Gpr101 | chrX:57496667-57503757 | 1.83754 | 3.77428 | 2.053985 | 5.00E-05 | 0.000607 |
| Tshz2 | chr2:169633645-169888504 | 4.64008 | 9.45077 | 2.036769 | 5.00E-05 | 0.000607 |
| Ncam2 | chr16:81200696-81624287 | 3.81494 | 7.64669 | 2.004406 | 5.00E-05 | 0.000607 |
| Bmpr2 | chr1:59764636-59870859 | 2.27633 | 4.55972 | 2.003101 | 5.00E-05 | 0.000607 |
| Lmbrd2 | chr15:9140569-9197450 | 2.22174 | 4.37413 | 1.968786 | 5.00E-05 | 0.000607 |
| Pcsk1 | chr13:75089986-75132498 | 2.99772 | 5.88567 | 1.963382 | 5.00E-05 | 0.000607 |
| Kcnk9 | chr15:72512118-72546279 | 3.22399 | 6.29022 | 1.951067 | 0.0003 | 0.002726 |
| Hs6st3 | chr14:119138264-119869815 | 1.88251 | 3.67127 | 1.950199 | 0.00065 | 0.005126 |
| D130017N08Rik | chr5:143758353-143764942 | 2.14543 | 4.15647 | 1.93736 | 0.0002 | 0.001971 |
| Cbln1 | chr8:87468852-87472592 | 2.69409 | 5.18528 | 1.924687 | 5.00E-05 | 0.000607 |
| Ttr | chr18:20665249-20674326 | 401.19 | 769.441 | 1.917897 | 0.0043 | 0.021953 |
| Rims4 | chr2:163863880-163918683 | 3.97185 | 7.54836 | 1.900465 | 0.00205 | 0.012328 |
| Rtn4rl2 | chr2:84871945-84886692 | 9.5207 | 17.8451 | 1.874347 | 5.00E-05 | 0.000607 |
| Fgf11 | chr11:69798201-69801625 | 6.69789 | 12.4684 | 1.861541 | 0.00075 | 0.005715 |
| Gpr26 | chr7:131966459-131985633 | 2.62407 | 4.87796 | 1.858929 | 5.00E-05 | 0.000607 |
| AI504432 | chr3:107039503-107054322 | 2.6889 | 4.98943 | 1.855565 | 5.00E-05 | 0.000607 |
| Glg1 | chr8:111157557-111259202 | 9.1118 | 16.8773 | 1.852247 | 5.00E-05 | 0.000607 |
| Cobl | chr11:12236675-12464960 | 2.79986 | 5.17886 | 1.849685 | 5.00E-05 | 0.000607 |
| Hs6st2 | chrX:51387211-51681602 | 4.31285 | 7.97219 | 1.848474 | 5.00E-05 | 0.000607 |
| Camk2d | chr3:126596972-126845054 | 7.253 | 13.3933 | 1.846588 | 5.00E-05 | 0.000607 |
| Cbln4 | chr2:172036335-172043466 | 3.09846 | 5.6794 | 1.832975 | 5.00E-05 | 0.000607 |
| Soat1 | chr1:156428107-156474328 | 2.06193 | 3.74031 | 1.813985 | 5.00E-05 | 0.000607 |
| Slc5a3 | chr16:92058321-92112227 | 2.01282 | 3.64356 | 1.810177 | 5.00E-05 | 0.000607 |
| Chrna7 | chr7:63098691-63212526 | 4.09335 | 7.4096 | 1.810155 | 5.00E-05 | 0.000607 |
| D8Ertd82e | chr8:36094827-36147787 | 3.00043 | 5.42758 | 1.808934 | 5.00E-05 | 0.000607 |
| Nr4a3 | chr4:48051247-48083352 | 4.1438 | 7.4766 | 1.804286 | 5.00E-05 | 0.000607 |
| Zbtb16 | chr9:48654296-48835945 | 6.41466 | 11.518 | 1.795575 | 5.00E-05 | 0.000607 |
| Plcxd2 | chr16:45959260-46010413 | 2.92207 | 5.22373 | 1.787681 | 5.00E-05 | 0.000607 |
| Slc17a6 | chr7:51621829-51671126 | 2.43785 | 4.34278 | 1.781398 | 5.00E-05 | 0.000607 |
| 2410127L17Rik | chr19:18670779-18704792 | 2.26714 | 4.02916 | 1.777199 | 0.0008 | 0.006001 |
| Pcdh7 | chr5:57718020-58132240 | 2.42346 | 4.29294 | 1.771409 | 5.00E-05 | 0.000607 |
| Zdhhc23 | chr16:43969145-43979050 | 4.39921 | 7.72339 | 1.755631 | 5.00E-05 | 0.000607 |
| Vat1l | chr8:114205639-114374070 | 5.49885 | 9.64177 | 1.753416 | 5.00E-05 | 0.000607 |
| Cntn4 | chr6:105677744-106699305 | 2.06887 | 3.62562 | 1.752464 | 5.00E-05 | 0.000607 |
| Spp1 | chr5:104435110-104441053 | 3.8621 | 6.76259 | 1.751014 | 0.0002 | 0.001971 |
| Unc5d | chr8:28646716-29219636 | 2.21204 | 3.86585 | 1.74764 | 5.00E-05 | 0.000607 |
| Thbd | chr2:148404470-148408188 | 2.55573 | 4.46573 | 1.74734 | 5.00E-05 | 0.000607 |
| Ace | chr11:105967944-105989964 | 2.86847 | 5.00578 | 1.745105 | 5.00E-05 | 0.000607 |
| Fnbp1l | chr3:122538718-122619667 | 9.2429 | 16.11 | 1.742959 | 5.00E-05 | 0.000607 |
| Rbms1 | chr2:60751952-60963204 | 2.22179 | 3.84825 | 1.732049 | 0.00015 | 0.001558 |
| Egr3 | chr14:70077444-70080157 | 6.1075 | 10.5478 | 1.727024 | 5.00E-05 | 0.000607 |
| Rasgef1b | chr5:99217419-99252927 | 3.32301 | 5.73384 | 1.725496 | 5.00E-05 | 0.000607 |
| Parm1 | chr5:91517699-91623996 | 7.94263 | 13.7004 | 1.72492 | 5.00E-05 | 0.000607 |
| Gpr161 | chr1:165295765-165321851 | 4.67605 | 8.04867 | 1.721254 | 5.00E-05 | 0.000607 |
| Fut9 | chr4:25609332-25800003 | 2.72591 | 4.68309 | 1.717991 | 5.00E-05 | 0.000607 |
| D7Ertd715e | chr7:59969576-59974431 | 2.37313 | 4.04101 | 1.702819 | 0.00075 | 0.005715 |
| Dcn | chr10:97479499-97518162 | 32.4768 | 55.2645 | 1.701661 | 5.00E-05 | 0.000607 |
| Cpeb3 | chr19:37021290-37207293 | 3.42632 | 5.81747 | 1.697877 | 5.00E-05 | 0.000607 |
| Slc6a13 | chr6:121300295-121337718 | 2.66942 | 4.52152 | 1.693821 | 0.00025 | 0.002352 |
| Rspo2 | chr15:43020794-43170818 | 5.2393 | 8.86498 | 1.692016 | 5.00E-05 | 0.000607 |
| Tgm2 | chr2:158116404-158146392 | 4.14912 | 7.0045 | 1.688189 | 5.00E-05 | 0.000607 |
| Cux2 | chr5:121860215-122047825 | 2.85321 | 4.81216 | 1.686578 | 5.00E-05 | 0.000607 |
| Slc6a20a | chr9:123636906-123678832 | 3.20703 | 5.38372 | 1.678725 | 0.0006 | 0.004799 |
| Gatad2b | chr3:90341653-90358120 | 4.0768 | 6.81367 | 1.671328 | 0.0002 | 0.001971 |
| Arl10 | chr13:54575012-54581128 | 4.54781 | 7.58434 | 1.667691 | 0.0022 | 0.013067 |
| Pcdh9 | chr14:93013699-93890669 | 7.74153 | 12.9016 | 1.666544 | 5.00E-05 | 0.000607 |
| Lrfn1 | chr7:28451984-28482345 | 6.42965 | 10.7085 | 1.665487 | 5.00E-05 | 0.000607 |
| Efemp1 | chr11:28853204-28926743 | 3.02874 | 5.03501 | 1.662411 | 0.001 | 0.007133 |
| Gjb2 | chr14:57098601-57104702 | 2.29411 | 3.78886 | 1.65156 | 0.00125 | 0.008413 |
| Klhl11 | chr11:100462611-100472782 | 2.57678 | 4.2532 | 1.650587 | 0.00075 | 0.005715 |
| B630005N14Rik | chr6:13625674-13677966 | 3.82551 | 6.30591 | 1.648384 | 5.00E-05 | 0.000607 |
| Gatsl2 | chr5:134099747-134141758 | 8.04944 | 13.2659 | 1.648053 | 5.00E-05 | 0.000607 |
| Thrb | chr14:17660959-18038088 | 3.81659 | 6.2856 | 1.646915 | 5.00E-05 | 0.000607 |
| Pcdh1 | chr18:38196693-38209762 | 12.1167 | 19.9551 | 1.646909 | 5.00E-05 | 0.000607 |
| Grik3 | chr4:125490830-125714173 | 4.87806 | 8.0067 | 1.64137 | 5.00E-05 | 0.000607 |
| Spred1 | chr2:117121449-117179336 | 8.45848 | 13.8649 | 1.639172 | 5.00E-05 | 0.000607 |
| Gmps | chr3:63976142-64019078 | 4.07608 | 6.67821 | 1.63839 | 5.00E-05 | 0.000607 |
| Ctgf | chr10:24595441-24598682 | 5.00147 | 8.19011 | 1.637541 | 5.00E-05 | 0.000607 |
| Nxph3 | chr11:95509845-95514565 | 8.78384 | 14.3785 | 1.636926 | 5.00E-05 | 0.000607 |
| Ccdc141 | chr2:77009905-77170635 | 3.09223 | 5.06102 | 1.636689 | 5.00E-05 | 0.000607 |
| Trove2 | chr1:143750790-143777051 | 3.25307 | 5.31683 | 1.634404 | 5.00E-05 | 0.000607 |
| Sesn3 | chr9:14276300-14326134 | 3.59772 | 5.86424 | 1.629988 | 0.00025 | 0.002352 |
| B230209E15Rik | chr7:61529409-61615327 | 4.98017 | 8.09115 | 1.624673 | 5.00E-05 | 0.000607 |
| L3mbtl3 | chr10:26275451-26375185 | 2.88522 | 4.67722 | 1.621096 | 0.0001 | 0.001096 |
| Cpeb2 | chr5:43151685-43289724 | 4.84566 | 7.83358 | 1.616618 | 5.00E-05 | 0.000607 |
| 1700020I14Rik | chr2:119594295-119600744 | 9.14658 | 14.7791 | 1.615806 | 5.00E-05 | 0.000607 |
| Nxph1 | chr6:8950018-9248578 | 8.84086 | 14.2803 | 1.615261 | 5.00E-05 | 0.000607 |
| Lipe | chr7:25376818-25566417 | 2.69483 | 4.34867 | 1.613708 | 0.0009 | 0.006615 |
| Cntnap4 | chr8:112570042-112882707 | 3.73335 | 6.02168 | 1.612943 | 5.00E-05 | 0.000607 |
| Lrrc8b | chr5:105415774-105486189 | 4.51413 | 7.27794 | 1.612258 | 5.00E-05 | 0.000607 |
| Nr2c2 | chr6:92091417-92184023 | 3.06014 | 4.93017 | 1.611093 | 0.0043 | 0.021953 |
| Fosl2 | chr5:32136471-32157839 | 7.3539 | 11.8394 | 1.609948 | 5.00E-05 | 0.000607 |
| Elavl4 | chr4:110203736-110351911 | 9.48253 | 15.2422 | 1.607398 | 5.00E-05 | 0.000607 |
| Tmem74 | chr15:43866694-43870029 | 3.66487 | 5.88845 | 1.606728 | 0.0025 | 0.014398 |
| Arhgap5 | chr12:52516076-52567851 | 5.14762 | 8.26178 | 1.604971 | 5.00E-05 | 0.000607 |
| Cit | chr5:115845655-116006341 | 2.53923 | 4.0749 | 1.604778 | 5.00E-05 | 0.000607 |
| Atp2b4 | chr1:133702673-133753747 | 12.584 | 20.1825 | 1.603822 | 5.00E-05 | 0.000607 |
| Sstr1 | chr12:58211803-58216036 | 6.31459 | 10.096 | 1.598837 | 5.00E-05 | 0.000607 |
| Mef2a | chr7:67233826-67372858 | 10.3758 | 16.5798 | 1.59793 | 5.00E-05 | 0.000607 |
| Enpp2 | chr15:54838897-54920146 | 47.4936 | 75.7004 | 1.593907 | 5.00E-05 | 0.000607 |
| Cdr1 | chrX:61183245-61185558 | 6.64426 | 10.5851 | 1.593119 | 5.00E-05 | 0.000607 |
| Tmem255a | chrX:38197300-38252481 | 7.41228 | 11.7958 | 1.591386 | 5.00E-05 | 0.000607 |
| Tmem56 | chr3:121202009-121263316 | 3.72184 | 5.92125 | 1.590947 | 5.00E-05 | 0.000607 |
| Hapln1 | chr13:89540635-89611832 | 3.52533 | 5.60378 | 1.589576 | 5.00E-05 | 0.000607 |
| Csgalnact1 | chr8:68356780-68735146 | 2.92361 | 4.64681 | 1.589408 | 5.00E-05 | 0.000607 |
| Slc7a11 | chr3:50364935-50443613 | 2.28489 | 3.62599 | 1.586943 | 5.00E-05 | 0.000607 |
| Cpeb4 | chr11:31872210-31931577 | 6.78455 | 10.7338 | 1.582095 | 5.00E-05 | 0.000607 |
| Dpp10 | chr1:123332137-124045559 | 7.58393 | 11.9841 | 1.580197 | 5.00E-05 | 0.000607 |
| Rgs16 | chr1:153740352-153745468 | 2.62254 | 4.13495 | 1.576697 | 0.00125 | 0.008413 |
| St3gal1 | chr15:67102874-67176882 | 5.44647 | 8.58591 | 1.576417 | 5.00E-05 | 0.000607 |
| Igf2 | chr7:142650767-142670356 | 15.6519 | 24.6604 | 1.575553 | 5.00E-05 | 0.000607 |
| Amer2 | chr14:60378285-60381003 | 3.18817 | 5.01148 | 1.571899 | 0.0008 | 0.006001 |
| Ptprm | chr17:66666847-67354459 | 3.40354 | 5.34706 | 1.571029 | 5.00E-05 | 0.000607 |
| Ttbk2 | chr2:120732816-120850584 | 4.64913 | 7.29961 | 1.570102 | 5.00E-05 | 0.000607 |
| Fam5c | chr1:146495665-146902472 | 3.05341 | 4.79337 | 1.569842 | 0.00035 | 0.003088 |
| Osbpl8 | chr10:111164801-111297247 | 6.13268 | 9.62519 | 1.569492 | 5.00E-05 | 0.000607 |
| Crim1 | chr17:78200247-78376592 | 8.57398 | 13.4556 | 1.569353 | 5.00E-05 | 0.000607 |
| Stx1b | chr7:127806843-127824531 | 46.3368 | 72.7093 | 1.569148 | 5.00E-05 | 0.000607 |
| Dyrk2 | chr10:118859348-118868903 | 4.18643 | 6.56586 | 1.568367 | 0.0005 | 0.004159 |
| Scai | chr2:39066214-39190730 | 3.37086 | 5.28564 | 1.568039 | 5.00E-05 | 0.000607 |
| Lrrtm3 | chr10:63430097-65003667 | 3.79736 | 5.95289 | 1.567639 | 5.00E-05 | 0.000607 |
| A230070E04Rik | chr14:68119544-68131371 | 13.2209 | 20.6824 | 1.564372 | 0.00325 | 0.017763 |
| Fam126b | chr1:58522805-58586333 | 6.15844 | 9.62284 | 1.562545 | 5.00E-05 | 0.000607 |
| Dpysl3 | chr18:43324226-43393331 | 4.86388 | 7.59471 | 1.561451 | 0.0001 | 0.001096 |
| Slc13a3 | chr2:165405294-165473197 | 3.02044 | 4.71588 | 1.561322 | 5.00E-05 | 0.000607 |
| Ankrd34b | chr13:92425968-92441658 | 2.463 | 3.84032 | 1.559204 | 0.0002 | 0.001971 |
| Alg10b | chr15:90224310-90230554 | 3.55405 | 5.53291 | 1.55679 | 5.00E-05 | 0.000607 |
| Zfp369 | chr13:65278853-65297795 | 2.4969 | 3.8863 | 1.55645 | 0.00065 | 0.005126 |
| Gpr17 | chr18:31931506-31958619 | 10.4246 | 16.2224 | 1.556165 | 5.00E-05 | 0.000607 |
| Cav2 | chr6:17281340-17289011 | 5.83628 | 9.07817 | 1.555472 | 5.00E-05 | 0.000607 |
| Hecw1 | chr13:14226437-14523226 | 3.56567 | 5.52385 | 1.549176 | 5.00E-05 | 0.000607 |
| Lzts1 | chr8:69135502-69140953 | 4.82302 | 7.46709 | 1.548219 | 0.00055 | 0.004491 |
| Lamp2 | chrX:38405063-38456455 | 13.8987 | 21.5125 | 1.547807 | 5.00E-05 | 0.000607 |
| Cdkn1a | chr17:29090985-29100722 | 5.36128 | 8.29255 | 1.546748 | 0.00015 | 0.001558 |
| Hspb1 | chr5:135887918-135889563 | 10.0431 | 15.526 | 1.545937 | 0.0007 | 0.005435 |
| Ccnt1 | chr15:98543210-98570864 | 4.03953 | 6.2439 | 1.5457 | 0.0038 | 0.020026 |
| Mtmr9 | chr14:63523609-63543953 | 10.3902 | 16.0548 | 1.545187 | 5.00E-05 | 0.000607 |
| Tnr | chr1:159523768-159924922 | 5.90642 | 9.11635 | 1.543465 | 5.00E-05 | 0.000607 |
| C030023E24Rik | chrX:61191292-61194164 | 3.79612 | 5.85815 | 1.543194 | 0.00015 | 0.001558 |
| Fmr1 | chrX:68678554-68717961 | 8.62811 | 13.314 | 1.543096 | 5.00E-05 | 0.000607 |
| Rgs17 | chr10:5825663-5922400 | 4.74371 | 7.31197 | 1.541403 | 5.00E-05 | 0.000607 |
| Zbtb33 | chrX:38189792-38197046 | 3.06053 | 4.70545 | 1.537462 | 5.00E-05 | 0.000607 |
| Eif2c2 | chr15:73101624-73184947 | 3.59846 | 5.53167 | 1.537233 | 5.00E-05 | 0.000607 |
| Dab2 | chr15:6299788-6440709 | 2.27153 | 3.49176 | 1.537184 | 0.0004 | 0.00346 |
| Hcn1 | chr13:117602319-117981028 | 5.40315 | 8.30244 | 1.536593 | 5.00E-05 | 0.000607 |
| Akap12 | chr10:4266328-4359471 | 2.66186 | 4.0881 | 1.535806 | 5.00E-05 | 0.000607 |
| Klhl34 | chrX:157818434-157820369 | 12.9371 | 19.8672 | 1.535676 | 5.00E-05 | 0.000607 |
| Tspan9 | chr6:127961399-128143578 | 12.3392 | 18.9285 | 1.534014 | 5.00E-05 | 0.000607 |
| Raph1 | chr1:60483184-60566765 | 4.33308 | 6.64419 | 1.533364 | 5.00E-05 | 0.000607 |
| Hpgd | chr8:56294551-56321046 | 2.54863 | 3.90436 | 1.531945 | 0.0114 | 0.046754 |
| Sstr4 | chr2:148395376-148396764 | 5.96896 | 9.14305 | 1.531766 | 0.0013 | 0.008656 |
| Edil3 | chr13:88821471-89323225 | 9.70154 | 14.8497 | 1.530654 | 5.00E-05 | 0.000607 |
| Ube3a | chr7:59228749-59306727 | 3.3086 | 5.06204 | 1.529964 | 5.00E-05 | 0.000607 |
| Dnajb14 | chr3:137867674-137908931 | 2.74684 | 4.19685 | 1.527883 | 0.00715 | 0.032557 |
| Kcng1 | chr2:168261697-168269331 | 8.34526 | 12.7478 | 1.52755 | 5.00E-05 | 0.000607 |
| Peg10 | chr6:4747305-4760516 | 3.12087 | 4.75679 | 1.524187 | 5.00E-05 | 0.000607 |
| Pdpr | chr8:111094744-111145498 | 2.46288 | 3.75227 | 1.523529 | 0.01095 | 0.045258 |
| Dnajc3 | chr14:118937931-118981702 | 8.18091 | 12.4353 | 1.520039 | 5.00E-05 | 0.000607 |
| Tbl1x | chrX:77511226-77660265 | 5.17615 | 7.86647 | 1.519753 | 5.00E-05 | 0.000607 |
| Plagl1 | chr10:13090787-13131695 | 5.58306 | 8.48451 | 1.519688 | 5.00E-05 | 0.000607 |
| Npas2 | chr1:39194271-39363240 | 6.20003 | 9.41685 | 1.518839 | 5.00E-05 | 0.000607 |
| Prrg3 | chrX:71963020-71972728 | 4.24666 | 6.44984 | 1.518803 | 5.00E-05 | 0.000607 |
| Hdac9 | chr12:34047581-34917095 | 5.01453 | 7.60613 | 1.516818 | 5.00E-05 | 0.000607 |
| Itgbl1 | chr14:123660139-123974079 | 2.51005 | 3.80324 | 1.515205 | 0.00395 | 0.020612 |
| 7-Mar | chr2:60209935-60248385 | 8.21244 | 12.438 | 1.514532 | 5.00E-05 | 0.000607 |
| Etl4 | chr2:20289912-20810535 | 6.68293 | 10.1204 | 1.514366 | 5.00E-05 | 0.000607 |
| Pdia4 | chr6:47796140-47813512 | 16.6231 | 25.0559 | 1.507294 | 5.00E-05 | 0.000607 |
| Megf9 | chr4:70431926-70534928 | 8.83634 | 13.3121 | 1.506517 | 5.00E-05 | 0.000607 |
| Tnks | chr8:34829178-34965690 | 5.58754 | 8.4099 | 1.505117 | 5.00E-05 | 0.000607 |
| Hivep1 | chr13:42052020-42185026 | 2.60134 | 3.90988 | 1.503025 | 5.00E-05 | 0.000607 |
| Nfix | chr8:84707598-84800340 | 18.9679 | 28.437 | 1.499217 | 5.00E-05 | 0.000607 |
| Kl | chr5:150952606-150993817 | 3.25145 | 4.87164 | 1.498298 | 5.00E-05 | 0.000607 |
| Glra2 | chrX:165129016-165326981 | 2.9627 | 4.43832 | 1.498066 | 0.0016 | 0.010187 |
| C230091D08Rik | chr7:59307923-59324149 | 5.43209 | 8.13638 | 1.497836 | 5.00E-05 | 0.000607 |
| Ppm1l | chr3:69316917-69555396 | 3.95078 | 5.91294 | 1.496651 | 5.00E-05 | 0.000607 |
| Prkar2a | chr9:108692142-108749511 | 7.5799 | 11.3406 | 1.496141 | 5.00E-05 | 0.000607 |
| Cacng2 | chr15:77993622-78119280 | 5.75696 | 8.61028 | 1.49563 | 5.00E-05 | 0.000607 |
| Lypla1 | chr1:4807892-4846735 | 6.485 | 9.69915 | 1.495628 | 5.00E-05 | 0.000607 |
| Tnpo1 | chr13:98842080-98926384 | 3.38183 | 5.0564 | 1.495167 | 5.00E-05 | 0.000607 |
| Acta2 | chr19:34240335-34255373 | 6.71189 | 10.0326 | 1.49475 | 5.00E-05 | 0.000607 |
| Ldb2 | chr5:44472132-44799707 | 14.2704 | 21.3264 | 1.49445 | 5.00E-05 | 0.000607 |
| Nuak1 | chr10:84371318-84440471 | 9.42701 | 14.0862 | 1.494238 | 5.00E-05 | 0.000607 |
| 9330182L06Rik | chr5:9266192-9480717 | 3.14784 | 4.70114 | 1.493449 | 5.00E-05 | 0.000607 |
| Adcy9 | chr16:4287544-4419587 | 8.29286 | 12.3843 | 1.493369 | 5.00E-05 | 0.000607 |
| Pag1 | chr3:9687481-9833679 | 2.93849 | 4.38713 | 1.492988 | 5.00E-05 | 0.000607 |
| Pigl | chr11:62458459-62513900 | 4.52929 | 6.75845 | 1.492165 | 0.0025 | 0.014398 |
| Spock2 | chr10:60106256-60133913 | 88.6287 | 132.214 | 1.491774 | 5.00E-05 | 0.000607 |
| Hmbox1 | chr14:64822217-64949847 | 3.06406 | 4.57041 | 1.491619 | 0.0011 | 0.007621 |
| Dlk1 | chr12:109452822-109463336 | 3.43155 | 5.10881 | 1.488776 | 0.00015 | 0.001558 |
| Kcnma1 | chr14:23298693-24004205 | 7.21425 | 10.73 | 1.487334 | 5.00E-05 | 0.000607 |
| Abhd2 | chr7:79273265-79361601 | 4.80983 | 7.1502 | 1.486581 | 5.00E-05 | 0.000607 |
| Peli3 | chr19:4931854-4943092 | 2.67597 | 3.9744 | 1.485218 | 0.01025 | 0.043155 |
| Id4 | chr13:48261426-48264036 | 13.0007 | 19.3041 | 1.484851 | 5.00E-05 | 0.000607 |
| Sos2 | chr12:69583760-69681852 | 3.69827 | 5.48904 | 1.484218 | 5.00E-05 | 0.000607 |
| C78339 | chr13:46669521-46675773 | 7.12443 | 10.5706 | 1.483712 | 0.00105 | 0.007395 |
| Tnfaip8l3 | chr9:54025605-54068411 | 5.79129 | 8.59146 | 1.483514 | 0.00015 | 0.001558 |
| Setbp1 | chr18:78750377-79109391 | 3.22514 | 4.78079 | 1.482351 | 5.00E-05 | 0.000607 |
| Cckbr | chr7:105425819-105436338 | 4.11649 | 6.0992 | 1.481651 | 0.0006 | 0.004799 |
| Serpinh1 | chr7:99345374-99353239 | 8.62805 | 12.776 | 1.480752 | 5.00E-05 | 0.000607 |
| Plxdc1 | chr11:97923236-97986446 | 4.38774 | 6.48521 | 1.47803 | 0.0003 | 0.002726 |
| Frrs1l | chr4:56960135-56990391 | 19.2258 | 28.3782 | 1.476048 | 5.00E-05 | 0.000607 |
| Slc36a4 | chr9:15709768-15738789 | 4.28498 | 6.31977 | 1.474866 | 0.00215 | 0.012828 |
| Zhx3 | chr2:160731309-160872990 | 3.43763 | 5.06361 | 1.472994 | 0.00795 | 0.03541 |
| 1500004A13Rik | chr3:88777056-88832487 | 5.82201 | 8.57038 | 1.472065 | 5.00E-05 | 0.000607 |
| Ednrb | chr14:103814614-103844476 | 6.03755 | 8.8863 | 1.471839 | 5.00E-05 | 0.000607 |
| Trpm3 | chr19:22139116-22989884 | 4.62168 | 6.79645 | 1.470558 | 5.00E-05 | 0.000607 |
| Dgkb | chr12:37880704-38633410 | 14.4244 | 21.1906 | 1.46908 | 5.00E-05 | 0.000607 |
| Mib1 | chr18:10725624-10812217 | 4.47445 | 6.5732 | 1.469052 | 5.00E-05 | 0.000607 |
| Tenm3 | chr8:48225664-48674690 | 6.13254 | 9.00791 | 1.468871 | 5.00E-05 | 0.000607 |
| Qpct | chr17:79051905-79090243 | 4.15601 | 6.10174 | 1.468173 | 0.0028 | 0.0158 |
| Gm16532 | chr7:6415174-6431086 | 3.95511 | 5.80499 | 1.467719 | 0.0017 | 0.010645 |
| Kitl | chr10:100015823-100100412 | 4.094 | 6.00553 | 1.46691 | 5.00E-05 | 0.000607 |
| Rgag4 | chrX:101849384-102092055 | 3.09175 | 4.53242 | 1.465972 | 0.00985 | 0.041903 |
| Cfh | chr1:140085854-140183411 | 2.99961 | 4.39561 | 1.465394 | 0.0003 | 0.002726 |
| Impad1 | chr4:4764350-4793306 | 6.73241 | 9.85888 | 1.464391 | 5.00E-05 | 0.000607 |
| Myoc | chr1:162639149-162658219 | 7.99815 | 11.7085 | 1.463901 | 0.0002 | 0.001971 |
| Slc8a1 | chr17:81373104-81738377 | 9.18003 | 13.4385 | 1.463884 | 5.00E-05 | 0.000607 |
| Sall1 | chr8:89027242-89044162 | 2.75878 | 4.03852 | 1.463879 | 5.00E-05 | 0.000607 |
| Glce | chr9:62057248-62070606 | 4.37313 | 6.40101 | 1.463714 | 0.0001 | 0.001096 |
| Ptprz1 | chr6:22875501-23052916 | 11.0995 | 16.2428 | 1.463381 | 5.00E-05 | 0.000607 |
| Eps8 | chr6:137477244-137649285 | 6.53177 | 9.54089 | 1.46069 | 5.00E-05 | 0.000607 |
| Grin2a | chr16:9577709-9992533 | 3.29838 | 4.81354 | 1.459365 | 5.00E-05 | 0.000607 |
| Ttc9 | chr12:81631368-81664941 | 7.33068 | 10.6981 | 1.45936 | 0.00015 | 0.001558 |
| Aqp4 | chr18:15389393-15403684 | 13.9603 | 20.3405 | 1.457025 | 5.00E-05 | 0.000607 |
| Htr2c | chrX:146962512-147197277 | 6.10129 | 8.88788 | 1.456721 | 5.00E-05 | 0.000607 |
| Tox2 | chr2:163225453-163323102 | 4.12454 | 6.00151 | 1.455074 | 0.00705 | 0.032226 |
| Coro2a | chr4:46536936-46601929 | 5.96522 | 8.6797 | 1.455051 | 5.00E-05 | 0.000607 |
| Meis2 | chr2:115861263-116065058 | 7.61131 | 11.067 | 1.45402 | 5.00E-05 | 0.000607 |
| Tgfbr2 | chr9:116087694-116175363 | 2.70471 | 3.92996 | 1.453006 | 0.0002 | 0.001971 |
| 9430020K01Rik | chr18:4634928-4682869 | 9.86273 | 14.3261 | 1.452549 | 5.00E-05 | 0.000607 |
| Gpr137c | chr14:45219716-45280976 | 3.40484 | 4.937 | 1.449995 | 0.0034 | 0.018393 |
| Hook3 | chr8:26021420-26119224 | 5.31572 | 7.70722 | 1.449892 | 5.00E-05 | 0.000607 |
| Tinagl1 | chr4:130165599-130175122 | 3.92051 | 5.68301 | 1.449559 | 0.0042 | 0.021546 |
| Larp4b | chr13:9093904-9173090 | 7.20262 | 10.4328 | 1.448473 | 5.00E-05 | 0.000607 |
| Gsg1l | chr7:125878418-126082411 | 9.28309 | 13.4404 | 1.447837 | 5.00E-05 | 0.000607 |
| Gabbr2 | chr4:46663897-46991714 | 24.3588 | 35.2635 | 1.44767 | 5.00E-05 | 0.000607 |
| Fktn | chr4:53714181-53763271 | 4.64702 | 6.72625 | 1.447433 | 5.00E-05 | 0.000607 |
| Jhdm1d | chr6:39118472-39206773 | 6.33825 | 9.17055 | 1.446858 | 5.00E-05 | 0.000607 |
| L3mbtl1 | chr2:162943464-162974522 | 4.1629 | 6.02214 | 1.446621 | 0.0009 | 0.006615 |
| Lims1 | chr10:58323465-58424691 | 4.85072 | 7.01305 | 1.445775 | 5.00E-05 | 0.000607 |
| Adcy8 | chr15:64699034-64922296 | 3.41063 | 4.93049 | 1.445624 | 5.00E-05 | 0.000607 |
| Tmem123 | chr9:7764076-7794332 | 3.38847 | 4.89551 | 1.444755 | 0.00135 | 0.008911 |
| Slc7a2 | chr8:40862366-40922070 | 2.45302 | 3.54344 | 1.444521 | 5.00E-05 | 0.000607 |
| Sema4d | chr13:51701247-51793644 | 6.90945 | 9.97632 | 1.443866 | 5.00E-05 | 0.000607 |
| Gnaz | chr10:74957476-75032586 | 11.3037 | 16.3151 | 1.443342 | 5.00E-05 | 0.000607 |
| Fchsd2 | chr7:101108774-101284405 | 6.86021 | 9.90131 | 1.443295 | 5.00E-05 | 0.000607 |
| Cnot6 | chr11:49674705-49712710 | 7.21836 | 10.4117 | 1.442391 | 5.00E-05 | 0.000607 |
| Eif4ebp2 | chr10:61432496-61452669 | 4.74732 | 6.83693 | 1.440166 | 0.0041 | 0.021197 |
| Ptgds | chr2:25466711-25469749 | 643.572 | 925.042 | 1.437356 | 5.00E-05 | 0.000607 |
| Rgs5 | chr1:169655500-169693526 | 10.7713 | 15.4553 | 1.434859 | 5.00E-05 | 0.000607 |
| Cacnb4 | chr2:52428319-52676582 | 10.3992 | 14.9051 | 1.433293 | 5.00E-05 | 0.000607 |
| Cbln2 | chr18:86713047-86718283 | 3.09795 | 4.43792 | 1.432534 | 0.0083 | 0.0366 |
| Wasf3 | chr5:146385005-146471125 | 11.3937 | 16.3216 | 1.432511 | 5.00E-05 | 0.000607 |
| Lphn3 | chr5:81021592-81795730 | 4.5397 | 6.501 | 1.432033 | 5.00E-05 | 0.000607 |
| Arhgap31 | chr16:38598342-38713035 | 4.18301 | 5.98969 | 1.431909 | 5.00E-05 | 0.000607 |
| Rbm12 | chr2:156071840-156111965 | 4.08684 | 5.85162 | 1.43182 | 0.00685 | 0.031529 |
| Homer1 | chr13:93304494-93404962 | 12.3804 | 17.7252 | 1.431715 | 5.00E-05 | 0.000607 |
| Nav3 | chr10:109683438-110000219 | 3.44222 | 4.92765 | 1.431533 | 5.00E-05 | 0.000607 |
| Ank1 | chr8:22974881-23150496 | 3.67771 | 5.2624 | 1.43089 | 5.00E-05 | 0.000607 |
| Ankrd12 | chr17:65967500-66077046 | 4.36841 | 6.24458 | 1.429486 | 5.00E-05 | 0.000607 |
| Elavl2 | chr4:91250766-91399984 | 11.0938 | 15.852 | 1.428906 | 5.00E-05 | 0.000607 |
| Syt4 | chr18:31437807-31447415 | 26.9812 | 38.5456 | 1.42861 | 5.00E-05 | 0.000607 |
| Luzp2 | chr7:54835244-55268888 | 10.4534 | 14.927 | 1.427956 | 5.00E-05 | 0.000607 |
| Vcpip1 | chr1:9723195-9771256 | 5.72542 | 8.16603 | 1.426276 | 5.00E-05 | 0.000607 |
| Cnot6l | chr5:96075737-96161990 | 3.2205 | 4.59076 | 1.425481 | 0.00915 | 0.039575 |
| Fads2 | chr19:10064163-10101503 | 37.7167 | 53.6732 | 1.423062 | 5.00E-05 | 0.000607 |
| Itgav | chr2:83724396-83806916 | 3.30841 | 4.70477 | 1.422064 | 5.00E-05 | 0.000607 |
| Exoc4 | chr6:33249149-33972930 | 9.97072 | 14.1627 | 1.420429 | 5.00E-05 | 0.000607 |
| Enah | chr1:181904444-182019980 | 12.4274 | 17.6113 | 1.417135 | 5.00E-05 | 0.000607 |
| Kcnj3 | chr2:55437155-55595525 | 19.8153 | 28.0426 | 1.415199 | 5.00E-05 | 0.000607 |
| Lpgat1 | chr1:191718023-191784257 | 19.3581 | 27.3932 | 1.415077 | 5.00E-05 | 0.000607 |
| Phactr2 | chr10:13207716-13474396 | 2.72264 | 3.85212 | 1.414847 | 5.00E-05 | 0.000607 |
| Scrn1 | chr6:54508815-54566382 | 24.5705 | 34.7591 | 1.414668 | 5.00E-05 | 0.000607 |
| Ubxn7 | chr16:32332251-32393747 | 3.46683 | 4.90301 | 1.414263 | 5.00E-05 | 0.000607 |
| Btbd11 | chr10:85386813-85660292 | 3.52616 | 4.98668 | 1.414196 | 0.0008 | 0.006001 |
| Kcnq3 | chr15:65994914-66286224 | 3.94443 | 5.56697 | 1.41135 | 0.0016 | 0.010187 |
| Plxnd1 | chr6:115954810-115995005 | 4.35048 | 6.13981 | 1.411295 | 5.00E-05 | 0.000607 |
| C77370 | chrX:104077434-104201117 | 2.69496 | 3.80105 | 1.410429 | 5.00E-05 | 0.000607 |
| Mrs2 | chr13:24992482-25020379 | 5.8876 | 8.30106 | 1.409923 | 0.00195 | 0.011841 |
| Kcnf1 | chr12:17172099-17176888 | 15.5883 | 21.9773 | 1.409859 | 5.00E-05 | 0.000607 |
| Shank1 | chr7:44310263-44358353 | 27.4959 | 38.7641 | 1.409814 | 5.00E-05 | 0.000607 |
| Sema3c | chr5:17574815-17730267 | 3.27069 | 4.60973 | 1.409406 | 0.0003 | 0.002726 |
| Satb1 | chr17:51736186-51833290 | 9.40145 | 13.2498 | 1.409336 | 5.00E-05 | 0.000607 |
| Soga3 | chr10:29143995-29199628 | 8.16703 | 11.5007 | 1.408186 | 5.00E-05 | 0.000607 |
| Spty2d1 | chr7:46990395-47008414 | 3.26962 | 4.60384 | 1.408066 | 0.00015 | 0.001558 |
| Rnd1 | chr15:98669204-98677461 | 4.10605 | 5.78144 | 1.40803 | 0.00485 | 0.024168 |
| Igsf21 | chr4:140026851-140246811 | 13.4604 | 18.9468 | 1.407596 | 5.00E-05 | 0.000607 |
| Abca5 | chr11:110269368-110337716 | 4.10289 | 5.77494 | 1.40753 | 5.00E-05 | 0.000607 |
| Mamld1 | chrX:71050255-71154717 | 6.8807 | 9.68413 | 1.407434 | 5.00E-05 | 0.000607 |
| Gpam | chr19:55069733-55099447 | 5.70787 | 8.03193 | 1.407168 | 5.00E-05 | 0.000607 |
| Acvr1b | chr15:101174124-101212601 | 16.4681 | 23.1638 | 1.406586 | 5.00E-05 | 0.000607 |
| Prkacb | chr3:146729578-146812946 | 59.2544 | 83.3172 | 1.406093 | 5.00E-05 | 0.000607 |
| Sh3bgrl2 | chr9:83548337-83600291 | 3.64149 | 5.11608 | 1.404941 | 0.0007 | 0.005435 |
| Rab11fip4 | chr11:79591211-79694012 | 12.8285 | 18.02 | 1.404685 | 5.00E-05 | 0.000607 |
| Zfp462 | chr4:54947944-55083563 | 3.65412 | 5.12673 | 1.403 | 5.00E-05 | 0.000607 |
| Zfp36l1 | chr12:80107759-80113013 | 4.96087 | 6.95709 | 1.402393 | 0.0006 | 0.004799 |
| Fstl5 | chr3:76074560-76710005 | 3.55858 | 4.99021 | 1.402304 | 0.00025 | 0.002352 |
| Slc35a3 | chr3:116670797-116712280 | 2.98447 | 4.18308 | 1.401616 | 0.00135 | 0.008911 |
| Dnm3 | chr1:161987301-162478034 | 10.7461 | 15.0548 | 1.400955 | 5.00E-05 | 0.000607 |
| Pdik1l | chr4:134275004-134287846 | 2.61012 | 3.65549 | 1.400506 | 0.00125 | 0.008413 |
| Sox5 | chr6:143828424-144209568 | 2.74705 | 3.84564 | 1.399916 | 5.00E-05 | 0.000607 |
| Nol4 | chr18:22693154-23041653 | 12.0192 | 16.8201 | 1.399436 | 5.00E-05 | 0.000607 |
| Cplx3 | chr9:57599991-57606281 | 3.2544 | 4.54935 | 1.397907 | 0.00435 | 0.022102 |
| Tcof1 | chr18:60813755-60848964 | 3.89895 | 5.44052 | 1.395381 | 0.0001 | 0.001096 |
| Gpr123 | chr7:139834173-139878088 | 14.9843 | 20.9005 | 1.394827 | 5.00E-05 | 0.000607 |
| Grin3a | chr4:49661610-49845769 | 4.9905 | 6.94757 | 1.392159 | 5.00E-05 | 0.000607 |
| 2900056M20Rik | chrX:152294827-152327493 | 5.07458 | 7.06375 | 1.391987 | 5.00E-05 | 0.000607 |
| Zbtb7a | chr10:81136270-81151657 | 8.76207 | 12.184 | 1.390539 | 5.00E-05 | 0.000607 |
| Cnksr2 | chrX:157821572-158043110 | 19.1673 | 26.6494 | 1.390358 | 5.00E-05 | 0.000607 |
| Tjp1 | chr7:65296164-65371244 | 10.1816 | 14.1555 | 1.390302 | 5.00E-05 | 0.000607 |
| Cd164 | chr10:41519499-41531042 | 16.3435 | 22.7182 | 1.390045 | 5.00E-05 | 0.000607 |
| Ppp1r12b | chr1:134765942-134955940 | 4.22883 | 5.87712 | 1.389774 | 0.00105 | 0.007395 |
| Prelp | chr1:133910303-133921401 | 6.10448 | 8.48289 | 1.389617 | 5.00E-05 | 0.000607 |
| Grm3 | chr5:9485235-9725352 | 5.50603 | 7.6505 | 1.389477 | 0.0002 | 0.001971 |
| Med13 | chr11:86265714-86357525 | 3.85872 | 5.35889 | 1.388774 | 5.00E-05 | 0.000607 |
| Klf9 | chr19:23141225-23166911 | 26.2007 | 36.3767 | 1.388387 | 5.00E-05 | 0.000607 |
| Reln | chr5:21884453-22344705 | 3.89142 | 5.40129 | 1.388 | 5.00E-05 | 0.000607 |
| Gfod1 | chr13:43195518-43304172 | 11.2535 | 15.6157 | 1.387631 | 5.00E-05 | 0.000607 |
| Setd1b | chr5:123142192-123168630 | 3.53506 | 4.89777 | 1.385484 | 5.00E-05 | 0.000607 |
| Ankrd52 | chr10:128377123-128394006 | 8.20346 | 11.3652 | 1.385415 | 5.00E-05 | 0.000607 |
| Sp3 | chr2:72936431-72989249 | 8.44966 | 11.7061 | 1.385393 | 0.00025 | 0.002352 |
| Chpt1 | chr10:88459586-88503970 | 6.25623 | 8.66353 | 1.384784 | 0.00415 | 0.021362 |
| Cd47 | chr16:49855653-49911683 | 9.72646 | 13.4682 | 1.384697 | 0.00025 | 0.002352 |
| Mta3 | chr17:83706162-83814905 | 12.0383 | 16.6683 | 1.384606 | 5.00E-05 | 0.000607 |
| Fndc3a | chr14:72537952-72710003 | 6.85658 | 9.49194 | 1.384355 | 5.00E-05 | 0.000607 |
| Clstn1 | chr4:149586637-149647899 | 144.031 | 199.35 | 1.384077 | 5.00E-05 | 0.000607 |
| Cdk12 | chr11:98203304-98253540 | 2.52755 | 3.49635 | 1.383296 | 0.0003 | 0.002726 |
| Mef2d | chr3:88142394-88169167 | 20.3744 | 28.1752 | 1.382873 | 5.00E-05 | 0.000607 |
| Fsd1l | chr4:53631470-53707009 | 3.76537 | 5.20333 | 1.381891 | 5.00E-05 | 0.000607 |
| Ipcef1 | chr10:6788600-7038209 | 4.02685 | 5.56462 | 1.381879 | 0.00025 | 0.002352 |
| Papd5 | chr8:88199212-88259722 | 7.94197 | 10.9725 | 1.381584 | 5.00E-05 | 0.000607 |
| Slc38a1 | chr15:96571417-96642913 | 15.1887 | 20.9763 | 1.381046 | 5.00E-05 | 0.000607 |
| Twsg1 | chr17:65923064-65951187 | 6.85419 | 9.46131 | 1.380369 | 5.00E-05 | 0.000607 |
| Akap5 | chr12:76324890-76334151 | 8.85089 | 12.2073 | 1.379217 | 5.00E-05 | 0.000607 |
| Arhgap32 | chr9:32116135-32265511 | 11.4269 | 15.7593 | 1.37914 | 5.00E-05 | 0.000607 |
| Tead1 | chr7:112679319-112906805 | 3.8797 | 5.34871 | 1.37864 | 5.00E-05 | 0.000607 |
| Fn1 | chr1:71585472-71653234 | 9.72163 | 13.4021 | 1.378586 | 5.00E-05 | 0.000607 |
| Strn | chr17:78653963-78736560 | 5.0186 | 6.91812 | 1.378496 | 0.00025 | 0.002352 |
| 1-Mar | chr8:65618039-66471637 | 6.07029 | 8.36613 | 1.378209 | 5.00E-05 | 0.000607 |
| Eea1 | chr10:95940662-96045518 | 4.70779 | 6.48711 | 1.377952 | 5.00E-05 | 0.000607 |
| Opa3 | chr7:19228388-19246817 | 12.0729 | 16.6353 | 1.377904 | 5.00E-05 | 0.000607 |
| Rabgap1l | chr1:160219173-160792938 | 19.7173 | 27.1598 | 1.37746 | 5.00E-05 | 0.000607 |
| Usp31 | chr7:121642020-121707253 | 6.96447 | 9.58831 | 1.376747 | 5.00E-05 | 0.000607 |
| Gabrb2 | chr11:42419756-42632591 | 17.4912 | 24.0765 | 1.376492 | 5.00E-05 | 0.000607 |
| Fos | chr12:85473900-85477270 | 4.86026 | 6.69 | 1.37647 | 0.0051 | 0.025119 |
| Slc7a3 | chrX:101079220-101085352 | 5.03404 | 6.92888 | 1.376405 | 0.00265 | 0.01513 |
| Sgms1 | chr19:32122726-32388454 | 8.96779 | 12.3399 | 1.376025 | 5.00E-05 | 0.000607 |
| Ids | chrX:70343069-70365085 | 61.3888 | 84.4649 | 1.375901 | 5.00E-05 | 0.000607 |
| Ssh2 | chr11:77216424-77460219 | 4.87216 | 6.70359 | 1.375897 | 5.00E-05 | 0.000607 |
| Srebf2 | chr15:82147268-82204960 | 22.9354 | 31.5435 | 1.375319 | 5.00E-05 | 0.000607 |
| Mctp1 | chr13:76384960-77031810 | 8.72749 | 12.0015 | 1.375138 | 5.00E-05 | 0.000607 |
| F3 | chr3:121723536-121735052 | 16.8729 | 23.1971 | 1.374814 | 0.0001 | 0.001096 |
| Usp13 | chr3:32817625-32935257 | 3.04969 | 4.19246 | 1.374717 | 0.0011 | 0.007621 |
| Fndc3b | chr3:27416161-27710439 | 2.70198 | 3.71367 | 1.374425 | 0.00035 | 0.003088 |
| Neto1 | chr18:86394951-86501897 | 17.6171 | 24.206 | 1.374006 | 5.00E-05 | 0.000607 |
| Prkab2 | chr3:97658211-97673067 | 11.0163 | 15.1342 | 1.373801 | 5.00E-05 | 0.000607 |
| Olfm2 | chr9:20667985-20728214 | 24.9569 | 34.2683 | 1.373099 | 5.00E-05 | 0.000607 |
| Etv1 | chr12:38780257-38868215 | 15.0359 | 20.6321 | 1.372189 | 5.00E-05 | 0.000607 |
| Slc39a10 | chr1:46807543-46853509 | 20.277 | 27.8173 | 1.371865 | 5.00E-05 | 0.000607 |
| Dennd5b | chr6:148988068-149101680 | 4.72247 | 6.47804 | 1.371748 | 5.00E-05 | 0.000607 |
| Pank3 | chr11:35769494-35791285 | 5.49372 | 7.52677 | 1.370068 | 5.00E-05 | 0.000607 |
| Ehd2 | chr7:15948986-15967535 | 2.84886 | 3.90243 | 1.369822 | 0.00955 | 0.040972 |
| Serpinb9 | chr13:33004540-33017955 | 2.67916 | 3.66972 | 1.369728 | 0.00785 | 0.035053 |
| Osbpl10 | chr9:115067278-115232223 | 6.72372 | 9.20877 | 1.369595 | 0.0024 | 0.013943 |
| Synj2 | chr17:5941279-6079739 | 8.83142 | 12.0933 | 1.369349 | 0.0002 | 0.001971 |
| Lrrtm2 | chr18:35118911-35254775 | 6.80853 | 9.32 | 1.368871 | 0.0012 | 0.008175 |
| Utrn | chr10:12382187-12861735 | 2.62435 | 3.58949 | 1.367763 | 5.00E-05 | 0.000607 |
| Lrrc58 | chr16:37868399-37888857 | 6.38137 | 8.72801 | 1.367733 | 5.00E-05 | 0.000607 |
| Hspa4l | chr3:40745612-40790365 | 20.0697 | 27.4222 | 1.366348 | 5.00E-05 | 0.000607 |
| Cep170 | chr1:176733652-176807124 | 8.13286 | 11.1112 | 1.366211 | 5.00E-05 | 0.000607 |
| Sik2 | chr9:50856934-51009073 | 4.20313 | 5.74114 | 1.36592 | 0.0081 | 0.035882 |
| Garem | chr18:21127341-21300139 | 4.96083 | 6.77564 | 1.365828 | 0.0001 | 0.001096 |
| Nkap | chrX:37126762-37168842 | 2.95937 | 4.0397 | 1.365054 | 0.00285 | 0.016005 |
| Gabra1 | chr11:42131479-42182930 | 36.3555 | 49.6084 | 1.364536 | 5.00E-05 | 0.000607 |
| Kbtbd7 | chr14:79426510-79431038 | 6.45925 | 8.80066 | 1.362489 | 5.00E-05 | 0.000607 |
| Ppp1r12a | chr10:108162399-108277575 | 10.5475 | 14.3703 | 1.362437 | 5.00E-05 | 0.000607 |
| Nos1ap | chr1:170317495-170589849 | 20.3996 | 27.7748 | 1.361537 | 5.00E-05 | 0.000607 |
| Slit3 | chr11:35121455-35708507 | 7.55902 | 10.2898 | 1.361261 | 5.00E-05 | 0.000607 |
| Caln1 | chr5:130369457-130840645 | 9.67599 | 13.171 | 1.361204 | 0.0002 | 0.001971 |
| Stox2 | chr8:47180047-47352348 | 8.25114 | 11.2294 | 1.360951 | 5.00E-05 | 0.000607 |
| Cacna1b | chr2:24606374-24763152 | 9.18936 | 12.5058 | 1.3609 | 5.00E-05 | 0.000607 |
| Cpsf6 | chr10:117344667-117376973 | 13.4769 | 18.3326 | 1.360298 | 5.00E-05 | 0.000607 |
| Elfn2 | chr15:78670006-78718113 | 16.6065 | 22.5769 | 1.359522 | 5.00E-05 | 0.000607 |
| Vma21 | chrX:71816778-71824706 | 8.75492 | 11.8953 | 1.358699 | 5.00E-05 | 0.000607 |
| Mid2 | chrX:140678027-140767715 | 3.25249 | 4.41727 | 1.358119 | 0.00025 | 0.002352 |
| Atp6v0a2 | chr5:124712207-124722146 | 13.4031 | 18.198 | 1.357746 | 0.0002 | 0.001971 |
| Slc7a1 | chr5:148327409-148399904 | 5.63223 | 7.64707 | 1.357734 | 5.00E-05 | 0.000607 |
| Ctdspl2 | chr2:121956452-122013586 | 2.94172 | 3.99372 | 1.357614 | 0.00245 | 0.014171 |
| Mef2c | chr13:83504033-83667079 | 17.3803 | 23.5906 | 1.357318 | 5.00E-05 | 0.000607 |
| Flt1 | chr5:147562195-147725988 | 5.71847 | 7.76141 | 1.357253 | 5.00E-05 | 0.000607 |
| Nfya | chr17:48386884-48409820 | 5.89278 | 7.99557 | 1.356842 | 0.0001 | 0.001096 |
| Sgpp1 | chr12:75714247-75735729 | 7.19951 | 9.76843 | 1.356819 | 5.00E-05 | 0.000607 |
| Gabra3 | chrX:72432675-72656246 | 19.2084 | 26.0551 | 1.356443 | 5.00E-05 | 0.000607 |
| Crkl | chr16:17451986-17486255 | 6.84457 | 9.27449 | 1.355014 | 5.00E-05 | 0.000607 |
| Oxr1 | chr15:41447481-41861047 | 56.2348 | 76.1877 | 1.354814 | 5.00E-05 | 0.000607 |
| Nov | chr15:54745927-54753761 | 53.6475 | 72.6676 | 1.354538 | 5.00E-05 | 0.000607 |
| Amot | chrX:145446423-145487046 | 3.23901 | 4.3854 | 1.353932 | 0.0002 | 0.001971 |
| Timp2 | chr11:118301060-118355411 | 90.5189 | 122.51 | 1.353419 | 5.00E-05 | 0.000607 |
| Apba3 | chr10:81268171-81291267 | 9.11453 | 12.3353 | 1.353367 | 0.00115 | 0.007885 |
| Gad2 | chr2:22622326-22693877 | 16.5464 | 22.3934 | 1.35337 | 5.00E-05 | 0.000607 |
| Vps37a | chr8:40492537-40634792 | 6.48248 | 8.76884 | 1.352698 | 0.0062 | 0.029235 |
| Plxnc1 | chr10:94790865-94944578 | 6.62984 | 8.96744 | 1.352588 | 5.00E-05 | 0.000607 |
| Nap1l2 | chrX:103184058-103186664 | 26.8098 | 36.2578 | 1.352408 | 5.00E-05 | 0.000607 |
| Lipa | chr19:34492315-34527474 | 7.01371 | 9.48211 | 1.351939 | 0.0004 | 0.00346 |
| S1pr1 | chr3:115710432-115715055 | 35.0782 | 47.3914 | 1.351021 | 5.00E-05 | 0.000607 |
| Gdpd5 | chr7:99381548-99460984 | 5.69798 | 7.69248 | 1.350036 | 0.0002 | 0.001971 |
| Gucy1a3 | chr3:82092426-82145877 | 5.44826 | 7.355 | 1.349972 | 0.00015 | 0.001558 |
| Phip | chr9:82866158-82975489 | 4.15185 | 5.60326 | 1.349582 | 5.00E-05 | 0.000607 |
| Lrba | chr3:86224689-86782694 | 2.6796 | 3.61586 | 1.349403 | 5.00E-05 | 0.000607 |
| Bcl9l | chr9:44499135-44510412 | 3.97923 | 5.36945 | 1.349369 | 0.00035 | 0.003088 |
| Dlx6as1 | chr6:6820545-6869533 | 2.69846 | 3.64074 | 1.349192 | 0.0089 | 0.038747 |
| Pank1 | chr19:34810893-34879455 | 3.95769 | 5.33709 | 1.348537 | 0.00625 | 0.029392 |
| Ddx3y | chrY:1260714-1286613 | 6.45262 | 8.69981 | 1.34826 | 0.00015 | 0.001558 |
| Zbtb38 | chr9:96685422-96752831 | 3.34145 | 4.50347 | 1.347759 | 0.0025 | 0.014398 |
| Ktn1 | chr14:47663755-47736564 | 12.1272 | 16.3437 | 1.347689 | 5.00E-05 | 0.000607 |
| Arhgap26 | chr18:38993144-39376285 | 10.2855 | 13.8611 | 1.347635 | 5.00E-05 | 0.000607 |
| 1700066M21Rik | chr1:57377619-57385422 | 7.01242 | 9.44992 | 1.347598 | 0.00045 | 0.003815 |
| Prkx | chrX:77762029-77795960 | 2.94067 | 3.96209 | 1.347343 | 0.0106 | 0.044208 |
| Phf6 | chrX:52912265-52956943 | 5.28725 | 7.11956 | 1.346553 | 0.00045 | 0.003815 |
| Dzank1 | chr2:144470556-144527398 | 21.8968 | 29.4845 | 1.346521 | 5.00E-05 | 0.000607 |
| Grm5 | chr7:87584167-88135063 | 17.7416 | 23.8863 | 1.346344 | 5.00E-05 | 0.000607 |
| Agfg1 | chr1:82839482-82896275 | 9.27174 | 12.4829 | 1.346338 | 5.00E-05 | 0.000607 |
| Mtmr4 | chr11:87592216-87616296 | 17.1584 | 23.0909 | 1.345749 | 5.00E-05 | 0.000607 |
| Mark4 | chr7:19426074-19458494 | 12.4879 | 16.8005 | 1.345342 | 0.0001 | 0.001096 |
| Fam63b | chr9:70599013-70657174 | 6.31777 | 8.49875 | 1.345214 | 5.00E-05 | 0.000607 |
| Cdh20 | chr1:104768818-104995481 | 3.06299 | 4.11949 | 1.344924 | 0.0071 | 0.032371 |
| Ipo7 | chr7:110018424-110055114 | 16.4895 | 22.1743 | 1.344753 | 5.00E-05 | 0.000607 |
| Clock | chr5:76183879-76469519 | 7.08915 | 9.53187 | 1.344572 | 5.00E-05 | 0.000607 |
| Tmem181a | chr17:6270474-6305783 | 20.4601 | 27.5083 | 1.344485 | 5.00E-05 | 0.000607 |
| Adcy5 | chr16:35155635-35304549 | 14.0621 | 18.9043 | 1.344344 | 5.00E-05 | 0.000607 |
| Gramd1b | chr9:40297906-40455764 | 18.9105 | 25.4195 | 1.3442 | 5.00E-05 | 0.000607 |
| Igf1r | chr7:67952256-68233667 | 5.03368 | 6.76479 | 1.343905 | 5.00E-05 | 0.000607 |
| Pcdh17 | chr14:84443562-84537060 | 5.49811 | 7.38876 | 1.343873 | 5.00E-05 | 0.000607 |
| Cacna2d1 | chr5:15934690-16374511 | 20.8506 | 28.0112 | 1.343424 | 5.00E-05 | 0.000607 |
| Abcb7 | chrX:104280564-104413846 | 4.51292 | 6.06094 | 1.34302 | 5.00E-05 | 0.000607 |
| Tob1 | chr11:94211453-94215492 | 8.39367 | 11.2626 | 1.341797 | 0.001 | 0.007133 |
| Bmpr1a | chr14:34411067-34502546 | 6.19983 | 8.31614 | 1.34135 | 5.00E-05 | 0.000607 |
| Lmln | chr16:33062520-33125659 | 2.93496 | 3.93189 | 1.339674 | 0.0059 | 0.028247 |
| Rimklb | chr6:122453608-122486305 | 7.91758 | 10.5986 | 1.338616 | 0.0001 | 0.001096 |
| Mafg | chr11:120628350-120633547 | 12.0365 | 16.1096 | 1.338396 | 0.0008 | 0.006001 |
| Lonrf2 | chr1:38794508-38821215 | 19.445 | 26.0245 | 1.338365 | 5.00E-05 | 0.000607 |
| Adcy3 | chr12:4133396-4240123 | 8.28829 | 11.0922 | 1.338298 | 0.00225 | 0.013268 |
| Ppp1r26 | chr2:28447940-28455508 | 3.3548 | 4.4893 | 1.338172 | 0.00095 | 0.006869 |
| Adam10 | chr9:70679000-70780229 | 11.7038 | 15.661 | 1.338112 | 5.00E-05 | 0.000607 |
| Aqr | chr2:114101171-114175339 | 6.25681 | 8.36995 | 1.337734 | 5.00E-05 | 0.000607 |
| Ankrd17 | chr5:90227165-90366185 | 11.6563 | 15.5853 | 1.337071 | 5.00E-05 | 0.000607 |
| Crebl2 | chr6:134830198-134857883 | 8.86085 | 11.8431 | 1.336565 | 0.0006 | 0.004799 |
| Slc4a10 | chr2:62046514-62326743 | 28.5322 | 38.134 | 1.336525 | 5.00E-05 | 0.000607 |
| Slco3a1 | chr7:74275417-74554780 | 9.654 | 12.8956 | 1.335778 | 0.0001 | 0.001096 |
| Gng4 | chr13:13784538-13827895 | 10.2983 | 13.7468 | 1.334861 | 5.00E-05 | 0.000607 |
| Stk35 | chr2:129798368-129832285 | 4.4465 | 5.93137 | 1.333941 | 0.0008 | 0.006001 |
| 11-Sep | chr5:93093456-93174958 | 12.1298 | 16.1754 | 1.333526 | 5.00E-05 | 0.000607 |
| Zdhhc2 | chr8:40423814-40484842 | 8.82836 | 11.77 | 1.333203 | 0.00065 | 0.005126 |
| Sorl1 | chr9:41968488-42124289 | 13.6024 | 18.1291 | 1.332787 | 5.00E-05 | 0.000607 |
| Usp32 | chr11:84984487-85139955 | 12.5724 | 16.7539 | 1.332594 | 5.00E-05 | 0.000607 |
| Homer2 | chr7:81600480-81706925 | 4.93388 | 6.57426 | 1.332473 | 5.00E-05 | 0.000607 |
| Fam217b | chr2:178414533-178422161 | 5.42749 | 7.22962 | 1.332037 | 0.00685 | 0.031529 |
| Zwint | chr10:72654894-72669789 | 93.6822 | 124.778 | 1.331929 | 5.00E-05 | 0.000607 |
| D1Ertd622e | chr1:97643901-97662018 | 9.69375 | 12.9067 | 1.331446 | 0.0004 | 0.00346 |
| Adamts4 | chr1:171250421-171259922 | 3.09933 | 4.12357 | 1.330471 | 0.0083 | 0.0366 |
| Pja2 | chr17:64281005-64331883 | 104.899 | 139.555 | 1.330375 | 5.00E-05 | 0.000607 |
| Serpini1 | chr3:75557532-75642523 | 52.7359 | 70.1582 | 1.330369 | 5.00E-05 | 0.000607 |
| Nr2f2 | chr7:70351949-70411146 | 5.88478 | 7.82618 | 1.329902 | 0.0003 | 0.002726 |
| Il6st | chr13:112464069-112506860 | 8.24098 | 10.9592 | 1.329842 | 5.00E-05 | 0.000607 |
| Idi1 | chr13:8885605-8892396 | 13.6768 | 18.1859 | 1.32969 | 5.00E-05 | 0.000607 |
| Lingo2 | chr4:35706647-36951744 | 3.09351 | 4.11314 | 1.329603 | 0.00895 | 0.038901 |
| Pecam1 | chr11:106654217-106715281 | 5.61383 | 7.46283 | 1.329365 | 0.00115 | 0.007885 |
| 2410131K14Rik | chr5:118245226-118263114 | 5.45553 | 7.2505 | 1.329018 | 0.00375 | 0.019852 |
| Qk | chr17:10206470-10319361 | 26.0004 | 34.5464 | 1.328687 | 5.00E-05 | 0.000607 |
| Lrig1 | chr6:94500313-94700145 | 4.86306 | 6.45977 | 1.328334 | 0.0045 | 0.022734 |
| Stam | chr2:14074111-14148330 | 11.5903 | 15.3949 | 1.328257 | 5.00E-05 | 0.000607 |
| Tmem47 | chrX:81070643-81097875 | 15.6056 | 20.7146 | 1.327382 | 5.00E-05 | 0.000607 |
| Myo5a | chr9:75071205-75223687 | 23.6228 | 31.3543 | 1.32729 | 5.00E-05 | 0.000607 |
| Far1 | chr7:113513861-113570458 | 15.1817 | 20.1488 | 1.327177 | 5.00E-05 | 0.000607 |
| Med13l | chr5:118560718-118765437 | 8.75928 | 11.6207 | 1.326673 | 5.00E-05 | 0.000607 |
| Chrm3 | chr13:9876612-10360803 | 6.65795 | 8.83061 | 1.326326 | 0.00075 | 0.005715 |
| Ubxn2b | chr4:6191104-6219788 | 5.7192 | 7.58453 | 1.326152 | 0.0001 | 0.001096 |
| Larp4 | chr15:99970073-100016358 | 4.99708 | 6.62655 | 1.326084 | 5.00E-05 | 0.000607 |
| Tanc2 | chr11:105589985-105929303 | 11.8787 | 15.75 | 1.325903 | 5.00E-05 | 0.000607 |
| Fbln2 | chr6:91212763-91272540 | 4.30165 | 5.70331 | 1.325842 | 0.0011 | 0.007621 |
| Apaf1 | chr10:90989310-91082743 | 3.96745 | 5.25519 | 1.324576 | 0.00015 | 0.001558 |
| Klhl3 | chr13:58004956-58102428 | 6.29747 | 8.341 | 1.3245 | 0.00625 | 0.029392 |
| Chic1 | chrX:103356475-103396118 | 4.59125 | 6.07901 | 1.324042 | 5.00E-05 | 0.000607 |
| Map1b | chr13:99421463-99516602 | 57.7724 | 76.4483 | 1.323267 | 5.00E-05 | 0.000607 |
| Tmx3 | chr18:90510153-90543267 | 9.20577 | 12.1805 | 1.323138 | 5.00E-05 | 0.000607 |
| Sgip1 | chr4:102760362-103011965 | 17.7869 | 23.5163 | 1.322113 | 5.00E-05 | 0.000607 |
| Lypd6 | chr2:50066428-50193569 | 4.09204 | 5.40829 | 1.321661 | 0.0039 | 0.020391 |
| Cmtm4 | chr8:104348192-104395807 | 14.3507 | 18.9641 | 1.321476 | 5.00E-05 | 0.000607 |
| Yap1 | chr9:7932000-8004596 | 4.54863 | 6.00938 | 1.321141 | 0.0008 | 0.006001 |
| Clcn6 | chr4:148006483-148038767 | 10.1411 | 13.3976 | 1.321119 | 0.00015 | 0.001558 |
| Fam115a | chr6:42672546-42693059 | 33.9516 | 44.8391 | 1.320677 | 5.00E-05 | 0.000607 |
| Arhgef11 | chr3:87618750-87748623 | 11.2186 | 14.8121 | 1.320316 | 0.00045 | 0.003815 |
| Zfp955b | chr17:33289543-33304689 | 3.43252 | 4.5304 | 1.319847 | 0.0115 | 0.047055 |
| Hmgcs1 | chr13:119690461-119708077 | 47.5169 | 62.704 | 1.319615 | 5.00E-05 | 0.000607 |
| Chl1 | chr6:103510875-103733035 | 21.2344 | 28.0191 | 1.319515 | 5.00E-05 | 0.000607 |
| Ell2 | chr13:75707483-75772358 | 4.19547 | 5.53169 | 1.318491 | 0.0031 | 0.017146 |
| Manea | chr4:26324505-26346652 | 5.37945 | 7.09242 | 1.318428 | 0.0005 | 0.004159 |
| Car10 | chr11:93099289-93601751 | 13.7982 | 18.1851 | 1.317933 | 5.00E-05 | 0.000607 |
| Phf8 | chrX:151520671-151633857 | 4.4521 | 5.86483 | 1.317318 | 0.0002 | 0.001971 |
| Cbfa2t3 | chr8:122625135-122699109 | 4.44108 | 5.84768 | 1.316725 | 0.0001 | 0.001096 |
| Vcam1 | chr3:116110019-116129688 | 6.00951 | 7.91035 | 1.316305 | 0.0011 | 0.007621 |
| Ptprb | chr10:116301373-116389538 | 3.1774 | 4.18204 | 1.316183 | 5.00E-05 | 0.000607 |
| Wwc1 | chr11:35839177-35980089 | 16.1181 | 21.2114 | 1.315999 | 5.00E-05 | 0.000607 |
| Limk1 | chr5:134656038-134688590 | 10.7626 | 14.1618 | 1.315834 | 0.0001 | 0.001096 |
| Paqr8 | chr1:20890621-20938756 | 19.9005 | 26.1753 | 1.315309 | 5.00E-05 | 0.000607 |
| Dpy19l1 | chr9:24411778-24503140 | 11.6546 | 15.3277 | 1.315163 | 5.00E-05 | 0.000607 |
| Fam13b | chr18:34409422-34506823 | 15.3086 | 20.1255 | 1.314653 | 5.00E-05 | 0.000607 |
| Dpysl5 | chr5:30711894-30799369 | 9.31727 | 12.2478 | 1.314527 | 5.00E-05 | 0.000607 |
| Ttll7 | chr3:146852366-146982750 | 16.1543 | 21.2284 | 1.314102 | 5.00E-05 | 0.000607 |
| Hspa13 | chr16:75755190-75766818 | 8.08395 | 10.6228 | 1.314061 | 0.0001 | 0.001096 |
| Zfp704 | chr3:9427009-9610085 | 4.53634 | 5.96091 | 1.314035 | 5.00E-05 | 0.000607 |
| Lifr | chr15:7129571-7197489 | 5.4261 | 7.12798 | 1.313647 | 5.00E-05 | 0.000607 |
| Slc2a1 | chr4:119108744-119137329 | 42.3728 | 55.6125 | 1.312458 | 5.00E-05 | 0.000607 |
| Pnmal1 | chr7:16959794-16962320 | 31.3872 | 41.1943 | 1.312455 | 5.00E-05 | 0.000607 |
| Shank2 | chr7:143948404-144470935 | 20.0475 | 26.3082 | 1.312293 | 0.0076 | 0.034268 |
| Mapk6 | chr9:75386900-75410014 | 6.51029 | 8.54251 | 1.312155 | 0.0004 | 0.00346 |
| Dnajc28 | chr16:91614256-91618999 | 3.60949 | 4.73481 | 1.311767 | 0.00885 | 0.038577 |
| Kpna1 | chr16:35983362-36036162 | 20.6808 | 27.1255 | 1.311627 | 5.00E-05 | 0.000607 |
| Akap6 | chr12:52699382-53151015 | 11.4516 | 15.009 | 1.310647 | 5.00E-05 | 0.000607 |
| Tbc1d30 | chr10:121263819-121311189 | 8.50219 | 11.1431 | 1.310615 | 5.00E-05 | 0.000607 |
| Glrb | chr3:80843603-80913624 | 39.8213 | 52.1897 | 1.310598 | 5.00E-05 | 0.000607 |
| Samd10 | chr2:181595217-181599147 | 8.46682 | 11.0965 | 1.310587 | 0.003 | 0.016689 |
| Spock3 | chr8:62951231-63357096 | 13.901 | 18.2076 | 1.309805 | 5.00E-05 | 0.000607 |
| Eltd1 | chr3:151437881-151545081 | 4.3712 | 5.72533 | 1.309784 | 0.0027 | 0.01535 |
| 2610005L07Rik | chr8:20385781-20424814 | 16.6997 | 21.8699 | 1.309598 | 5.00E-05 | 0.000607 |
| Caskin2 | chr11:115799351-115813592 | 5.00832 | 6.55793 | 1.309407 | 0.00045 | 0.003815 |
| Sobp | chr10:43002499-43174530 | 13.3342 | 17.4568 | 1.309175 | 5.00E-05 | 0.000607 |
| Nap1l3 | chrX:122394560-122397385 | 10.9064 | 14.2763 | 1.308984 | 0.0001 | 0.001096 |
| Nr3c1 | chr18:39410544-39487245 | 7.60963 | 9.95677 | 1.308443 | 5.00E-05 | 0.000607 |
| Cdh11 | chr8:102632994-102785111 | 11.2423 | 14.708 | 1.308273 | 5.00E-05 | 0.000607 |
| Nxt2 | chrX:142227936-142239692 | 8.15278 | 10.6633 | 1.307934 | 0.00125 | 0.008413 |
| Bdp1 | chr13:100017993-100104070 | 3.037 | 3.97166 | 1.307758 | 0.00025 | 0.002352 |
| Tcf20 | chr15:82808625-82912134 | 10.851 | 14.1888 | 1.307603 | 5.00E-05 | 0.000607 |
| Cntn1 | chr15:92051164-92341967 | 42.704 | 55.8328 | 1.307437 | 5.00E-05 | 0.000607 |
| Dr1 | chr5:108268896-108280521 | 11.0485 | 14.4406 | 1.307019 | 0.0001 | 0.001096 |
| Ppargc1a | chr5:51454248-51553921 | 4.4701 | 5.84178 | 1.306857 | 0.00025 | 0.002352 |
| Gsk3b | chr16:38085063-38246079 | 13.0125 | 17.0041 | 1.306751 | 5.00E-05 | 0.000607 |
| Prrc2c | chr1:162671784-162740556 | 14.8621 | 19.4189 | 1.306605 | 5.00E-05 | 0.000607 |
| Dmxl1 | chr18:49832996-49965473 | 4.01845 | 5.2501 | 1.306499 | 5.00E-05 | 0.000607 |
| Creb1 | chr1:64532803-64604548 | 4.83943 | 6.3227 | 1.306497 | 5.00E-05 | 0.000607 |
| Hcfc2 | chr10:82699006-82741392 | 6.25029 | 8.16448 | 1.306256 | 0.00535 | 0.026156 |
| Zik1 | chr7:10487223-10495381 | 3.12317 | 4.07839 | 1.30585 | 0.00815 | 0.036058 |
| Cdc37l1 | chr19:28990493-29017569 | 15.8233 | 20.6615 | 1.305764 | 5.00E-05 | 0.000607 |
| Rock1 | chr18:10064400-10181792 | 3.79429 | 4.95333 | 1.30547 | 0.0007 | 0.005435 |
| Zfp397 | chr18:23954687-23964670 | 6.62653 | 8.64984 | 1.305335 | 5.00E-05 | 0.000607 |
| Zfp871 | chr17:32765496-32788287 | 10.5273 | 13.7396 | 1.30514 | 5.00E-05 | 0.000607 |
| Usp49 | chr17:47630689-47684067 | 3.0783 | 4.0164 | 1.304746 | 0.0028 | 0.0158 |
| Efnb2 | chr8:8617438-8660773 | 7.8828 | 10.2817 | 1.304321 | 0.0002 | 0.001971 |
| Pid1 | chr1:84036292-84284645 | 10.33 | 13.4627 | 1.303262 | 0.00025 | 0.002352 |
| Klf6 | chr13:5861488-5870393 | 5.6751 | 7.39342 | 1.302782 | 0.00065 | 0.005126 |
| Efr3a | chr15:65787040-65873812 | 25.0162 | 32.5906 | 1.30278 | 5.00E-05 | 0.000607 |
| Ythdf3 | chr3:16183182-16217037 | 11.0596 | 14.4005 | 1.302081 | 5.00E-05 | 0.000607 |
| Ubn2 | chr6:38433924-38512763 | 3.86003 | 5.02518 | 1.30185 | 5.00E-05 | 0.000607 |
| Hspa5 | chr2:34772089-34776529 | 108.77 | 141.596 | 1.301793 | 5.00E-05 | 0.000607 |
| Tmem164 | chrX:142681399-142843494 | 3.68402 | 4.79408 | 1.301318 | 0.00315 | 0.017359 |
| Nrp2 | chr1:62703316-62818692 | 7.78018 | 10.1218 | 1.300972 | 5.00E-05 | 0.000607 |
| Lrch3 | chr16:32914099-33056186 | 5.11481 | 6.6511 | 1.300361 | 0.00435 | 0.022102 |
| Pigg | chr5:108312924-108348779 | 3.57333 | 4.6463 | 1.300272 | 0.0091 | 0.039456 |
| Erc1 | chr6:119570795-119848150 | 6.69378 | 8.70321 | 1.300194 | 5.00E-05 | 0.000607 |
| Sgtb | chr13:104109789-104141441 | 18.8937 | 24.5496 | 1.299354 | 5.00E-05 | 0.000607 |
| Pcgf3 | chr5:108461331-108503099 | 5.88707 | 7.64747 | 1.299028 | 0.0012 | 0.008175 |
| Epm2aip1 | chr9:111271844-111279091 | 18.581 | 24.134 | 1.298854 | 5.00E-05 | 0.000607 |
| Tspan14 | chr14:40906443-40966807 | 13.3229 | 17.3042 | 1.298831 | 5.00E-05 | 0.000607 |
| Klf15 | chr6:90462625-90475209 | 8.55457 | 11.1106 | 1.298791 | 0.00225 | 0.013268 |
| Heatr5b | chr17:78752905-78835381 | 4.55689 | 5.91784 | 1.298658 | 0.0001 | 0.001096 |
| Chm | chrX:113040591-113185515 | 6.80256 | 8.83383 | 1.298604 | 0.0002 | 0.001971 |
| Gm2115 | chr7:84528953-84578339 | 11.3511 | 14.7368 | 1.298271 | 0.0021 | 0.012579 |
| Hif1a | chr12:73907866-73947530 | 9.18774 | 11.9204 | 1.297425 | 5.00E-05 | 0.000607 |
| Kpna4 | chr3:69072220-69127092 | 17.3878 | 22.558 | 1.297346 | 5.00E-05 | 0.000607 |
| Robo1 | chr16:72663148-73046100 | 4.53319 | 5.88064 | 1.297241 | 0.0002 | 0.001971 |
| Dio2 | chr12:90724551-90738438 | 8.86164 | 11.4928 | 1.296916 | 5.00E-05 | 0.000607 |
| Grm7 | chr6:110645597-111567230 | 11.1528 | 14.464 | 1.296894 | 5.00E-05 | 0.000607 |
| Wwp1 | chr4:19608299-19709004 | 4.33614 | 5.62168 | 1.296471 | 0.00035 | 0.003088 |
| Pde10a | chr17:8801744-8986648 | 10.9257 | 14.1634 | 1.296338 | 5.00E-05 | 0.000607 |
| Evi5l | chr8:4166566-4193701 | 13.3658 | 17.316 | 1.295545 | 0.00255 | 0.014638 |
| Zbtb6 | chr2:37425499-37430919 | 2.9415 | 3.81085 | 1.295546 | 0.00935 | 0.04026 |
| Lats1 | chr10:7681208-7716461 | 4.93069 | 6.38785 | 1.295529 | 0.00015 | 0.001558 |
| Man1c1 | chr4:134561689-134704290 | 6.31135 | 8.17042 | 1.29456 | 0.0003 | 0.002726 |
| Dcp2 | chr18:44380499-44424969 | 6.35345 | 8.22289 | 1.29424 | 5.00E-05 | 0.000607 |
| Rnf144b | chr13:47122719-47247991 | 3.28625 | 4.25257 | 1.294049 | 0.006 | 0.028609 |
| Fmnl2 | chr2:52857867-53134202 | 7.35605 | 9.5146 | 1.293439 | 5.00E-05 | 0.000607 |
| Pak3 | chrX:143518590-143797796 | 11.646 | 15.057 | 1.29289 | 5.00E-05 | 0.000607 |
| Cadm1 | chr9:47530351-47853385 | 42.017 | 54.2917 | 1.292137 | 5.00E-05 | 0.000607 |
| L1cam | chrX:73853779-73880834 | 28.9524 | 37.4076 | 1.292038 | 5.00E-05 | 0.000607 |
| Nebl | chr2:17346733-17731068 | 8.225 | 10.6251 | 1.291805 | 0.0009 | 0.006615 |
| Tbc1d9 | chr8:83165351-83272940 | 14.0071 | 18.0878 | 1.291331 | 5.00E-05 | 0.000607 |
| Cep104 | chr4:153975560-154007225 | 7.15455 | 9.23702 | 1.291069 | 0.0017 | 0.010645 |
| Cacng7 | chr7:3336584-3366948 | 41.8479 | 54.0273 | 1.29104 | 0.00055 | 0.004491 |
| Armcx4 | chrX:134686518-134697772 | 8.16122 | 10.5359 | 1.290971 | 5.00E-05 | 0.000607 |
| Flrt3 | chr2:140395429-142390050 | 8.63928 | 11.1514 | 1.290779 | 0.00175 | 0.010881 |
| Zfp770 | chr2:114193460-114201432 | 4.1861 | 5.40249 | 1.290578 | 0.004 | 0.020812 |
| Avl9 | chr6:56714904-56761911 | 6.08221 | 7.84685 | 1.290131 | 0.0001 | 0.001096 |
| Rb1cc1 | chr1:6214661-6276104 | 7.53725 | 9.72354 | 1.290065 | 5.00E-05 | 0.000607 |
| Nipbl | chr15:8291224-8444463 | 4.74578 | 6.12221 | 1.290032 | 0.0001 | 0.001096 |
| Ythdc2 | chr18:44828664-44889720 | 4.2937 | 5.53703 | 1.289571 | 0.0007 | 0.005435 |
| Psd3 | chr8:67689081-67910946 | 40.5509 | 52.289 | 1.289466 | 5.00E-05 | 0.000607 |
| Atxn2 | chr5:121711608-121814950 | 16.3331 | 21.0518 | 1.288904 | 5.00E-05 | 0.000607 |
| Kdm5c | chrX:152233229-152274354 | 8.67161 | 11.1726 | 1.288411 | 5.00E-05 | 0.000607 |
| Pnpla8 | chr12:44269153-44313435 | 16.4787 | 21.2297 | 1.288312 | 5.00E-05 | 0.000607 |
| Lrrc7 | chr3:158084438-158562221 | 6.21818 | 8.00443 | 1.287263 | 5.00E-05 | 0.000607 |
| Lgals3bp | chr11:118392751-118401931 | 6.3248 | 8.13981 | 1.286967 | 0.0113 | 0.046434 |
| Igfbp2 | chr1:72824479-72852471 | 21.0314 | 27.0632 | 1.2868 | 0.0009 | 0.006615 |
| Fam171b | chr2:83812727-83881358 | 30.7264 | 39.5374 | 1.286757 | 5.00E-05 | 0.000607 |
| Ppig | chr2:69723087-69754059 | 8.72607 | 11.228 | 1.286719 | 5.00E-05 | 0.000607 |
| Spred2 | chr11:19924441-20022597 | 13.2837 | 17.089 | 1.286464 | 0.0001 | 0.001096 |
| Hs2st1 | chr3:144431106-144570216 | 8.02213 | 10.3182 | 1.286217 | 5.00E-05 | 0.000607 |
| Zmiz1 | chr14:25459184-25666747 | 14.5478 | 18.7094 | 1.286064 | 5.00E-05 | 0.000607 |
| Gria4 | chr9:4417892-4796234 | 13.2381 | 17.0244 | 1.286015 | 5.00E-05 | 0.000607 |
| Rab8b | chr9:66843663-66919705 | 10.4323 | 13.4107 | 1.285498 | 5.00E-05 | 0.000607 |
| Mll3 | chr5:25271793-25498783 | 5.3358 | 6.85895 | 1.285459 | 5.00E-05 | 0.000607 |
| Msi2 | chr11:88339381-88718267 | 10.4645 | 13.4515 | 1.285441 | 0.0001 | 0.001096 |
| Usp46 | chr5:74000037-74068411 | 21.1546 | 27.1891 | 1.285257 | 5.00E-05 | 0.000607 |
| Tshz1 | chr18:84011626-84086562 | 3.18374 | 4.09121 | 1.285033 | 0.00425 | 0.021761 |
| Ndst1 | chr18:60685975-60713389 | 10.6808 | 13.7202 | 1.284567 | 5.00E-05 | 0.000607 |
| Gnai1 | chr5:18265134-18360413 | 37.852 | 48.6116 | 1.284254 | 5.00E-05 | 0.000607 |
| Cbll1 | chr12:31484828-31499616 | 4.95621 | 6.36172 | 1.283586 | 0.0041 | 0.021197 |
| Heca | chr10:17900465-17948067 | 4.53669 | 5.82311 | 1.283559 | 0.00635 | 0.029796 |
| Pik3r1 | chr13:101680761-101768217 | 10.6274 | 13.6406 | 1.283531 | 5.00E-05 | 0.000607 |
| Rgs7bp | chr13:104947152-105054930 | 25.1107 | 32.2294 | 1.283493 | 5.00E-05 | 0.000607 |
| Abhd3 | chr18:10644410-10706696 | 17.815 | 22.8546 | 1.282885 | 0.00045 | 0.003815 |
| Asap1 | chr15:64086839-64382919 | 11.7028 | 15.0103 | 1.282625 | 5.00E-05 | 0.000607 |
| Gm14436 | chr2:175470024-175483322 | 4.70869 | 6.03922 | 1.282569 | 0.00615 | 0.029076 |
| Ptprf | chr4:118208212-118291397 | 7.61163 | 9.76103 | 1.282384 | 5.00E-05 | 0.000607 |
| Kcnc4 | chr3:107438302-107458898 | 24.8065 | 31.8058 | 1.282156 | 5.00E-05 | 0.000607 |
| Slc5a5 | chr8:70882888-70892757 | 8.21216 | 10.5268 | 1.281855 | 0.00185 | 0.011363 |
| Pgm2l1 | chr7:100227606-100278872 | 33.9734 | 43.5167 | 1.280905 | 5.00E-05 | 0.000607 |
| Shprh | chr10:11149429-11215273 | 4.65598 | 5.96373 | 1.280875 | 0.0002 | 0.001971 |
| Tmod3 | chr9:75497783-75559657 | 6.26115 | 8.01854 | 1.280682 | 0.00235 | 0.013705 |
| Ntrk2 | chr13:58806595-59129967 | 46.7113 | 59.8112 | 1.280444 | 5.00E-05 | 0.000607 |
| Top1 | chr2:160645896-160722763 | 18.6898 | 23.9273 | 1.280233 | 5.00E-05 | 0.000607 |
| Acsl4 | chrX:142317992-142390535 | 11.9391 | 15.2834 | 1.280113 | 5.00E-05 | 0.000607 |
| B230219D22Rik | chr13:55693123-55703500 | 19.1 | 24.4479 | 1.279995 | 5.00E-05 | 0.000607 |
| Tmcc3 | chr10:94514856-94590954 | 7.93093 | 10.1482 | 1.279573 | 5.00E-05 | 0.000607 |
| Zbtb11 | chr16:55973267-56008912 | 7.9365 | 10.154 | 1.279405 | 0.0004 | 0.00346 |
| Rab21 | chr10:115289861-115315591 | 20.842 | 26.6619 | 1.279239 | 0.00025 | 0.002352 |
| Fam179b | chr12:64965741-65022573 | 5.75215 | 7.35738 | 1.279066 | 0.0001 | 0.001096 |
| Itch | chr2:155133480-155226855 | 9.92569 | 12.6918 | 1.278682 | 5.00E-05 | 0.000607 |
| Dlc1 | chr8:36567738-36952442 | 7.97532 | 10.1932 | 1.278093 | 5.00E-05 | 0.000607 |
| Cct6a | chr5:129787384-129794500 | 60.5798 | 77.4254 | 1.278073 | 5.00E-05 | 0.000607 |
| Edem3 | chr1:151755373-151822328 | 6.24477 | 7.98015 | 1.277893 | 0.00015 | 0.001558 |
| Wwc2 | chr8:47827605-47990551 | 4.70465 | 6.01182 | 1.277846 | 0.00195 | 0.011841 |
| Cdkn1b | chr6:134920400-134925525 | 8.62836 | 11.0226 | 1.277485 | 0.0029 | 0.016217 |
| Sestd1 | chr2:77180339-77280592 | 8.09095 | 10.3359 | 1.277464 | 5.00E-05 | 0.000607 |
| Trim23 | chr13:104179097-104202048 | 7.79856 | 9.96128 | 1.277323 | 0.0004 | 0.00346 |
| Zswim6 | chr13:107724616-107890064 | 4.47695 | 5.71834 | 1.277285 | 0.0017 | 0.010645 |
| Tbl2 | chr5:135149710-135162662 | 5.92124 | 7.56226 | 1.277141 | 0.0014 | 0.009144 |
| Trhde | chr10:114398820-114801370 | 3.60222 | 4.6 | 1.27699 | 0.00325 | 0.017763 |
| Tmed7 | chr18:46585927-46597535 | 18.1329 | 23.1544 | 1.276928 | 5.00E-05 | 0.000607 |
| Aasdhppt | chr9:4294792-4309494 | 7.12154 | 9.09314 | 1.27685 | 0.0042 | 0.021546 |
| Ntng2 | chr2:29194820-29252993 | 17.3469 | 22.1462 | 1.276666 | 0.00615 | 0.029076 |
| 2410066E13Rik | chr6:54681771-54700406 | 14.76 | 18.8395 | 1.276389 | 0.00225 | 0.013268 |
| Atf2 | chr2:73816521-73892628 | 17.7498 | 22.6537 | 1.276279 | 5.00E-05 | 0.000607 |
| Elovl6 | chr3:129532385-129638493 | 9.03287 | 11.5272 | 1.276139 | 5.00E-05 | 0.000607 |
| Atp2b3 | chrX:73503085-73571005 | 21.8232 | 27.8478 | 1.276064 | 5.00E-05 | 0.000607 |
| Wdfy3 | chr5:101832952-102069921 | 11.3609 | 14.4961 | 1.275964 | 5.00E-05 | 0.000607 |
| Ddx3x | chrX:13281021-13293983 | 36.9802 | 47.1751 | 1.275685 | 5.00E-05 | 0.000607 |
| Kdm5a | chr6:120324321-120444574 | 3.32801 | 4.24128 | 1.274419 | 0.001 | 0.007133 |
| Ralbp1 | chr17:65848427-65885755 | 15.1614 | 19.315 | 1.273959 | 5.00E-05 | 0.000607 |
| Ankhd1 | chr18:36560602-36658908 | 7.91875 | 10.0876 | 1.273888 | 5.00E-05 | 0.000607 |
| Slc36a1 | chr11:55204339-55236330 | 11.5142 | 14.6676 | 1.273871 | 5.00E-05 | 0.000607 |
| Esyt2 | chr12:116281221-116373098 | 7.2214 | 9.19809 | 1.273727 | 0.0001 | 0.001096 |
| Fcho2 | chr13:98723405-98815449 | 6.58687 | 8.38941 | 1.273657 | 0.0006 | 0.004799 |
| Marcks | chr10:37133242-37138926 | 24.2052 | 30.8213 | 1.273334 | 5.00E-05 | 0.000607 |
| Dnajb1 | chr8:83608174-83611903 | 19.9769 | 25.4351 | 1.273226 | 0.0002 | 0.001971 |
| Lrrtm4 | chr6:80018876-80024932 | 7.50652 | 9.55353 | 1.272698 | 0.00175 | 0.010881 |
| Cachd1 | chr4:100776678-101003748 | 5.04842 | 6.42362 | 1.272402 | 0.00175 | 0.010881 |
| Scaper | chr9:55549882-55938115 | 5.32389 | 6.77235 | 1.272068 | 0.00095 | 0.006869 |
| Arhgef26 | chr3:62338776-62462221 | 8.14706 | 10.3633 | 1.272029 | 0.00015 | 0.001558 |
| Dner | chr1:84369838-84696221 | 36.0807 | 45.8735 | 1.271414 | 5.00E-05 | 0.000607 |
| Txnip | chr3:96555767-96566801 | 10.0901 | 12.828 | 1.271345 | 0.0014 | 0.009144 |
| Bace1 | chr9:45838528-45862484 | 19.9058 | 25.3043 | 1.271202 | 5.00E-05 | 0.000607 |
| Wdr48 | chr9:119894894-119937558 | 11.5679 | 14.7007 | 1.270818 | 0.00505 | 0.024965 |
| Phf2 | chr13:48801749-48870885 | 9.42773 | 11.9799 | 1.270709 | 0.00015 | 0.001558 |
| Nufip2 | chr11:77686138-77717966 | 4.52086 | 5.74302 | 1.270338 | 5.00E-05 | 0.000607 |
| Mecp2 | chrX:74026823-74085636 | 10.7833 | 13.6973 | 1.270233 | 5.00E-05 | 0.000607 |
| Slc4a7 | chr14:14703024-14799943 | 6.69563 | 8.50355 | 1.270015 | 5.00E-05 | 0.000607 |
| Hlf | chr11:90336534-90390917 | 25.2622 | 32.0779 | 1.269798 | 5.00E-05 | 0.000607 |
| Pcdh10 | chr3:45378397-45439309 | 7.0335 | 8.93059 | 1.269722 | 0.0005 | 0.004159 |
| Snx30 | chr4:59805649-59904740 | 6.2216 | 7.89598 | 1.269124 | 5.00E-05 | 0.000607 |
| Pde4b | chr4:102254741-102607262 | 16.9092 | 21.4594 | 1.269096 | 5.00E-05 | 0.000607 |
| Rnf128 | chrX:139563339-139673145 | 8.78918 | 11.1522 | 1.268856 | 0.0035 | 0.018791 |
| Mest | chr6:30733505-30748466 | 10.2661 | 13.0214 | 1.268388 | 0.0024 | 0.013943 |
| Ino80d | chr1:63047800-63114267 | 2.94527 | 3.73534 | 1.26825 | 0.0003 | 0.002726 |
| Pfkfb3 | chr2:11471430-11553929 | 7.44883 | 9.44245 | 1.267642 | 0.00025 | 0.002352 |
| Klhl18 | chr9:110425925-110476694 | 7.52046 | 9.53072 | 1.267305 | 0.00065 | 0.005126 |
| Etv5 | chr16:22381312-22439570 | 19.6921 | 24.9418 | 1.266589 | 5.00E-05 | 0.000607 |
| Magee1 | chrX:105120395-105123911 | 36.2513 | 45.9119 | 1.26649 | 5.00E-05 | 0.000607 |
| Pde7a | chr3:19225917-19311322 | 7.00529 | 8.87104 | 1.266334 | 0.00325 | 0.017763 |
| Ep300 | chr15:81586213-81652077 | 7.39842 | 9.36862 | 1.2663 | 5.00E-05 | 0.000607 |
| Sh3d19 | chr3:86084433-86130521 | 3.91272 | 4.95265 | 1.265782 | 0.00205 | 0.012328 |
| Tmem170b | chr13:41606215-41641357 | 8.71984 | 11.0348 | 1.265482 | 5.00E-05 | 0.000607 |
| Dcaf12l1 | chrX:44786566-44790197 | 12.3372 | 15.6123 | 1.265465 | 0.0001 | 0.001096 |
| Clip4 | chr17:71769690-71864210 | 10.6446 | 13.4696 | 1.265393 | 0.0002 | 0.001971 |
| Mmab | chr5:114431033-114444027 | 10.4484 | 13.2202 | 1.265285 | 0.0007 | 0.005435 |
| Thoc2 | chrX:41794993-41911901 | 5.35203 | 6.77117 | 1.265159 | 0.00035 | 0.003088 |
| Slc35f1 | chr10:52690500-53111622 | 9.74317 | 12.3223 | 1.264712 | 0.0001 | 0.001096 |
| Trpc1 | chr9:95706628-95750358 | 6.80595 | 8.60702 | 1.264632 | 0.00495 | 0.02454 |
| Prkci | chr3:30995770-31052740 | 10.1808 | 12.8741 | 1.264547 | 0.0001 | 0.001096 |
| Ing3 | chr6:21949614-21976037 | 4.50211 | 5.69257 | 1.264423 | 0.00915 | 0.039575 |
| Rab39b | chrX:75572057-75578204 | 10.3096 | 13.0345 | 1.264307 | 0.0006 | 0.004799 |
| Chmp2b | chr16:65539132-65562697 | 25.4648 | 32.1842 | 1.26387 | 5.00E-05 | 0.000607 |
| 2610203C20Rik | chr9:41581338-41592487 | 15.0069 | 18.964 | 1.263685 | 0.0004 | 0.00346 |
| Nrxn1 | chr17:90033643-91092802 | 28.5824 | 36.1094 | 1.263344 | 5.00E-05 | 0.000607 |
| Traf6 | chr2:101678439-101700974 | 3.43945 | 4.34371 | 1.262908 | 0.0059 | 0.028247 |
| Slc39a12 | chr2:14388397-14494977 | 9.58805 | 12.1078 | 1.262801 | 0.0033 | 0.017971 |
| Csmd1 | chr8:15892544-17535385 | 5.68891 | 7.18302 | 1.262636 | 5.00E-05 | 0.000607 |
| C530008M17Rik | chr5:76840598-76873554 | 10.357 | 13.0755 | 1.262479 | 5.00E-05 | 0.000607 |
| Dnajc16 | chr4:141761997-141790644 | 11.4383 | 14.439 | 1.262338 | 0.0001 | 0.001096 |
| Acly | chr11:100476351-100528000 | 28.683 | 36.2068 | 1.262309 | 5.00E-05 | 0.000607 |
| Lmtk2 | chr5:144100435-144188204 | 12.4731 | 15.7447 | 1.262292 | 5.00E-05 | 0.000607 |
| Clip1 | chr5:123579069-123684291 | 20.9573 | 26.4513 | 1.262152 | 0.0001 | 0.001096 |
| Atxn1l | chr8:109726450-109737739 | 5.05306 | 6.37611 | 1.261831 | 0.0004 | 0.00346 |
| Chrna4 | chr2:181022310-181039177 | 5.20437 | 6.56553 | 1.261542 | 0.0031 | 0.017146 |
| Scarb2 | chr5:92443872-92505608 | 14.4439 | 18.2194 | 1.261391 | 0.00145 | 0.009402 |
| Paqr9 | chr9:95559816-95562121 | 7.67822 | 9.68469 | 1.26132 | 0.00875 | 0.038251 |
| AI414108 | chr9:27352684-27357543 | 10.6438 | 13.4223 | 1.261044 | 0.0001 | 0.001096 |
| Gja1 | chr10:56377299-56390419 | 49.9868 | 63.0071 | 1.260475 | 5.00E-05 | 0.000607 |
| Spats2 | chr15:99126844-99212466 | 8.18351 | 10.3145 | 1.2604 | 0.01095 | 0.045258 |
| Lrp4 | chr2:91457530-91513901 | 3.98356 | 5.01865 | 1.25984 | 0.00095 | 0.006869 |
| Usp9x | chrX:13071497-13173327 | 14.7773 | 18.6166 | 1.259811 | 5.00E-05 | 0.000607 |
| Bod1l | chr5:41787539-41844315 | 6.2569 | 7.87823 | 1.259127 | 0.0001 | 0.001096 |
| Shisa9 | chr16:11984112-12270904 | 13.8343 | 17.4113 | 1.25856 | 5.00E-05 | 0.000607 |
| Tmem64 | chr4:15265819-15286753 | 6.4072 | 8.05912 | 1.257822 | 0.00145 | 0.009402 |
| Zfp644 | chr5:106616740-106696830 | 7.21549 | 9.07408 | 1.257583 | 0.0004 | 0.00346 |
| Neto2 | chr8:85636587-85691009 | 5.9116 | 7.43306 | 1.257369 | 0.001 | 0.007133 |
| Tnrc6b | chr15:80711312-80941086 | 5.02319 | 6.31352 | 1.256875 | 5.00E-05 | 0.000607 |
| Rb1 | chr14:73195501-73325791 | 6.86896 | 8.63202 | 1.256671 | 0.0034 | 0.018393 |
| Ntng1 | chr3:109780049-110143472 | 7.21464 | 9.06542 | 1.256531 | 0.0007 | 0.005435 |
| Nfat5 | chr8:107293469-107379517 | 9.0188 | 11.3285 | 1.256098 | 5.00E-05 | 0.000607 |
| Gabra2 | chr5:70961056-71095849 | 20.5526 | 25.8145 | 1.256021 | 5.00E-05 | 0.000607 |
| Vstm2a | chr11:16257723-16284551 | 20.8544 | 26.1909 | 1.255893 | 5.00E-05 | 0.000607 |
| Cadm2 | chr16:66655420-67620908 | 12.2995 | 15.4467 | 1.25588 | 5.00E-05 | 0.000607 |
| Acvr2a | chr2:48814108-48949267 | 10.7836 | 13.537 | 1.255332 | 0.0047 | 0.023553 |
| Rab3c | chr13:110054186-110280206 | 44.9539 | 56.4296 | 1.255277 | 5.00E-05 | 0.000607 |
| Aph1b | chr9:66775486-66795423 | 7.06196 | 8.86434 | 1.255224 | 0.00115 | 0.007885 |
| Tmem30a | chr9:79768940-79793430 | 66.499 | 83.448 | 1.254876 | 5.00E-05 | 0.000607 |
| Psmd5 | chr2:34852088-34870962 | 18.3328 | 22.9934 | 1.254222 | 0.00075 | 0.005715 |
| Nap1l5 | chr6:58833699-58920396 | 129.006 | 161.799 | 1.254197 | 0.00235 | 0.013705 |
| Ankib1 | chr5:3689999-3803109 | 6.52623 | 8.18471 | 1.254125 | 0.0002 | 0.001971 |
| Arhgef18 | chr8:3393007-3456600 | 10.8187 | 13.568 | 1.254125 | 0.0002 | 0.001971 |
| App | chr16:84954435-85173707 | 407.296 | 510.777 | 1.254068 | 0.00365 | 0.01938 |
| Rgmb | chr17:15806252-15826586 | 11.17 | 14.0065 | 1.253939 | 0.00235 | 0.013705 |
| Cdh2 | chr18:16588876-16809049 | 20.5469 | 25.762 | 1.253814 | 5.00E-05 | 0.000607 |
| 4632415L05Rik | chr3:19894870-19898984 | 4.70156 | 5.89201 | 1.253203 | 0.00755 | 0.034086 |
| Mtss1 | chr15:58941233-59082026 | 8.24552 | 10.3332 | 1.25319 | 0.0006 | 0.004799 |
| Slc25a16 | chr10:62920632-62946494 | 10.3626 | 12.9852 | 1.253083 | 0.00125 | 0.008413 |
| Specc1 | chr11:62077096-62223013 | 11.6323 | 14.5734 | 1.252839 | 5.00E-05 | 0.000607 |
| Itga3 | chr11:95044481-95076714 | 3.89226 | 4.87362 | 1.252131 | 0.0081 | 0.035882 |
| Luzp1 | chr4:136469760-136549318 | 12.5646 | 15.7319 | 1.252081 | 5.00E-05 | 0.000607 |
| Flywch1 | chr17:23755422-23771591 | 47.2549 | 59.164 | 1.252018 | 5.00E-05 | 0.000607 |
| Zfyve16 | chr13:92487748-92530810 | 3.33599 | 4.17501 | 1.251506 | 0.01155 | 0.047187 |
| St13 | chr15:81365043-81399694 | 45.757 | 57.2584 | 1.251358 | 0.0001 | 0.001096 |
| Cacnb1 | chr11:98002900-98022627 | 32.3429 | 40.4617 | 1.251023 | 0.00015 | 0.001558 |
| Prkar2b | chr12:31958478-32061279 | 9.05769 | 11.3274 | 1.250584 | 0.00185 | 0.011363 |
| Ypel2 | chr11:86936424-86993762 | 7.2645 | 9.08481 | 1.250576 | 0.0006 | 0.004799 |
| N4bp2l2 | chr5:150635972-150665612 | 3.16587 | 3.95905 | 1.250541 | 0.00145 | 0.009402 |
| Faxc | chr4:21931325-22001461 | 8.57621 | 10.7238 | 1.250412 | 5.00E-05 | 0.000607 |
| Kcnb1 | chr2:167095968-167188818 | 14.6366 | 18.3015 | 1.250393 | 5.00E-05 | 0.000607 |
| Ccdc88a | chr11:29373774-29510811 | 5.76375 | 7.20137 | 1.249424 | 0.0001 | 0.001096 |
| Ccng1 | chr11:40748551-40755286 | 17.0823 | 21.3359 | 1.249006 | 0.0001 | 0.001096 |
| Mpdz | chr4:81278499-81442805 | 11.869 | 14.8206 | 1.248681 | 5.00E-05 | 0.000607 |
| Nova1 | chr12:46694516-46818775 | 5.03217 | 6.28177 | 1.248322 | 0.0006 | 0.004799 |
| Tenm2 | chr11:36006668-36944243 | 14.9307 | 18.6273 | 1.247584 | 0.0001 | 0.001096 |
| Soga2 | chr17:66336981-66449750 | 4.38125 | 5.46587 | 1.247559 | 0.00105 | 0.007395 |
| Fubp1 | chr3:152210457-152236830 | 14.3437 | 17.8946 | 1.247558 | 0.0001 | 0.001096 |
| Dnajc6 | chr4:101496647-101642799 | 43.7701 | 54.6054 | 1.24755 | 5.00E-05 | 0.000607 |
| Ahdc1 | chr4:133011505-133078110 | 5.57518 | 6.95469 | 1.247438 | 0.0007 | 0.005435 |
| Pdzd8 | chr19:59296083-59345780 | 6.67619 | 8.3277 | 1.247373 | 0.0003 | 0.002726 |
| Dvl3 | chr16:20517063-20532187 | 8.69243 | 10.8422 | 1.247315 | 0.00515 | 0.025295 |
| Mxd4 | chr5:34176579-34187710 | 16.2249 | 20.237 | 1.24728 | 0.01165 | 0.047522 |
| Celf4 | chr18:25477619-25753983 | 76.9279 | 95.9417 | 1.247164 | 5.00E-05 | 0.000607 |
| Slc30a1 | chr1:191894071-191913247 | 5.57764 | 6.95555 | 1.247042 | 0.00345 | 0.018588 |
| Zkscan16 | chr4:58943627-58958355 | 9.86895 | 12.3025 | 1.246587 | 0.0029 | 0.016217 |
| Ankrd29 | chr18:12252356-12305720 | 6.62684 | 8.25994 | 1.246437 | 0.00665 | 0.030876 |
| Zdhhc21 | chr4:82798737-82859661 | 5.10037 | 6.35673 | 1.246327 | 0.00025 | 0.002352 |
| D15Ertd621e | chr15:58415467-58457801 | 9.81476 | 12.2284 | 1.245919 | 0.00035 | 0.003088 |
| Cacna2d2 | chr9:107399879-107529343 | 5.28939 | 6.58943 | 1.245783 | 0.00295 | 0.016462 |
| Dcc | chr18:71253631-72351069 | 3.48177 | 4.33709 | 1.245657 | 0.0008 | 0.006001 |
| Lsamp | chr16:41533341-42146213 | 16.6752 | 20.7704 | 1.245586 | 0.0005 | 0.004159 |
| Ube2d3 | chr3:135438758-135467178 | 59.0543 | 73.5518 | 1.245494 | 5.00E-05 | 0.000607 |
| Fam81a | chr9:70089309-70141557 | 31.265 | 38.9352 | 1.245329 | 5.00E-05 | 0.000607 |
| Prepl | chr17:85028346-85090274 | 85.5571 | 106.544 | 1.245297 | 5.00E-05 | 0.000607 |
| Rims1 | chr1:22468190-22805724 | 19.9267 | 24.8117 | 1.245148 | 0.00105 | 0.007395 |
| Cacna1e | chr1:154392518-154725920 | 18.967 | 23.6141 | 1.24501 | 5.00E-05 | 0.000607 |
| Lin7a | chr10:107271830-107425143 | 7.78623 | 9.68942 | 1.24443 | 0.0002 | 0.001971 |
| Lasp1 | chr11:97799671-97838764 | 27.7319 | 34.5088 | 1.244372 | 0.0001 | 0.001096 |
| Ppap2b | chr4:105157346-105232767 | 51.7984 | 64.4476 | 1.244201 | 5.00E-05 | 0.000607 |
| Creld2 | chr15:88819645-88826681 | 15.4938 | 19.2751 | 1.244052 | 0.0104 | 0.043665 |
| Lrrc4c | chr2:97467673-97631664 | 18.6091 | 23.1479 | 1.243902 | 0.00015 | 0.001558 |
| Kcnc2 | chr10:112271122-112466304 | 13.9324 | 17.3267 | 1.243626 | 5.00E-05 | 0.000607 |
| Vezf1 | chr11:88068278-88084729 | 5.20783 | 6.47656 | 1.24362 | 0.00545 | 0.026547 |
| Prdm2 | chr4:143107390-143212709 | 9.04341 | 11.2433 | 1.243259 | 5.00E-05 | 0.000607 |
| Sc5d | chr9:42254176-42264300 | 16.7685 | 20.8459 | 1.243158 | 0.001 | 0.007133 |
| Dcx | chrX:143855841-143933219 | 2.96224 | 3.68219 | 1.243042 | 0.0031 | 0.017146 |
| Pcdh19 | chrX:133582860-133688993 | 6.37666 | 7.9244 | 1.24272 | 0.0001 | 0.001096 |
| Ppp2r5e | chr12:75450880-75596200 | 15.1873 | 18.8728 | 1.24267 | 0.00015 | 0.001558 |
| Ttc39b | chr4:83220300-83324189 | 4.57296 | 5.68039 | 1.242169 | 0.00105 | 0.007395 |
| Csk | chr9:57626645-57645180 | 12.107 | 15.0373 | 1.242034 | 0.00385 | 0.02021 |
| Rpgrip1l | chr8:91217029-91313222 | 3.14652 | 3.9074 | 1.241816 | 0.00765 | 0.034391 |
| Thsd4 | chr9:59966930-60511035 | 6.70907 | 8.33003 | 1.241607 | 0.0004 | 0.00346 |
| Kit | chr5:75574986-75656721 | 18.8773 | 23.4357 | 1.241475 | 5.00E-05 | 0.000607 |
| Rnf144a | chr12:26306797-26415256 | 4.07725 | 5.06176 | 1.241464 | 0.007 | 0.032067 |
| Fam120a | chr13:48879216-48967828 | 30.5617 | 37.9394 | 1.241403 | 5.00E-05 | 0.000607 |
| Gramd4 | chr15:86057694-86137636 | 9.98689 | 12.3951 | 1.241137 | 0.00065 | 0.005126 |
| Rnf165 | chr18:77456109-77565136 | 8.83312 | 10.9603 | 1.240819 | 0.0001 | 0.001096 |
| Ncoa2 | chr1:13139158-13374083 | 8.33516 | 10.3422 | 1.240792 | 5.00E-05 | 0.000607 |
| Map7d2 | chrX:159414577-159498947 | 33.3332 | 41.3578 | 1.240739 | 5.00E-05 | 0.000607 |
| Pde4d | chr13:108654176-109955969 | 5.34679 | 6.63373 | 1.240694 | 0.00075 | 0.005715 |
| Eaf1 | chr14:31495078-31509858 | 4.50344 | 5.58734 | 1.240683 | 0.01225 | 0.049532 |
| Mapk4 | chr18:73928485-74064949 | 10.0079 | 12.4161 | 1.24063 | 0.0004 | 0.00346 |
| Opcml | chr9:27791268-28925048 | 29.8337 | 37.0067 | 1.240433 | 5.00E-05 | 0.000607 |
| Sash1 | chr10:8722218-8886070 | 7.93532 | 9.84085 | 1.240133 | 5.00E-05 | 0.000607 |
| Sgsm1 | chr5:113243219-113310786 | 22.3294 | 27.6906 | 1.240096 | 5.00E-05 | 0.000607 |
| Akap11 | chr14:78492245-78536860 | 20.6879 | 25.6457 | 1.239647 | 5.00E-05 | 0.000607 |
| Nek7 | chr1:138484713-138619696 | 7.67186 | 9.50956 | 1.239538 | 0.00555 | 0.026911 |
| Zc2hc1a | chr3:7503425-7553848 | 11.6846 | 14.4835 | 1.239538 | 0.001 | 0.007133 |
| Nlk | chr11:78567167-78697425 | 11.5821 | 14.3549 | 1.239404 | 0.00035 | 0.003088 |
| Frmpd4 | chrX:167471308-168577233 | 6.69338 | 8.29445 | 1.239202 | 0.00015 | 0.001558 |
| Prex2 | chr1:10993464-11303682 | 4.25504 | 5.27197 | 1.238994 | 0.0049 | 0.02436 |
| B3galt1 | chr2:68104671-68122882 | 5.69312 | 7.05325 | 1.238908 | 0.00225 | 0.013268 |
| Fnip1 | chr11:54438178-54518241 | 6.27523 | 7.77371 | 1.238793 | 0.00125 | 0.008413 |
| Tmpo | chr10:91147570-91171582 | 6.81452 | 8.4401 | 1.238547 | 0.0088 | 0.038422 |
| Phc3 | chr3:30899294-30969415 | 6.80482 | 8.42784 | 1.23851 | 5.00E-05 | 0.000607 |
| Tmem33 | chr5:67260651-67291461 | 12.7609 | 15.8021 | 1.238322 | 5.00E-05 | 0.000607 |
| Unc5c | chr3:141465563-141834922 | 4.55062 | 5.63362 | 1.23799 | 0.0006 | 0.004799 |
| Gda | chr19:21391306-21472661 | 41.1789 | 50.9672 | 1.237702 | 5.00E-05 | 0.000607 |
| Aff4 | chr11:53350766-53421830 | 16.4935 | 20.4124 | 1.237603 | 5.00E-05 | 0.000607 |
| Wapal | chr14:34673927-34746217 | 6.15364 | 7.61404 | 1.237323 | 0.00385 | 0.02021 |
| Lztfl1 | chr9:123697592-123717557 | 8.97809 | 11.1078 | 1.237212 | 0.0028 | 0.0158 |
| Tbr1 | chr2:61804452-61814113 | 8.29878 | 10.266 | 1.237049 | 0.00255 | 0.014638 |
| Scn2a1 | chr2:65670444-65767447 | 21.2714 | 26.3137 | 1.237046 | 5.00E-05 | 0.000607 |
| Ipmk | chr10:71347792-71385885 | 8.5134 | 10.5275 | 1.23658 | 0.0004 | 0.00346 |
| Cdk18 | chr1:132113546-132139685 | 11.5408 | 14.2709 | 1.236561 | 0.00195 | 0.011841 |
| Zranb2 | chr3:157534396-157548339 | 54.8665 | 67.8375 | 1.23641 | 5.00E-05 | 0.000607 |
| Xbp1 | chr11:5520640-5525993 | 41.3069 | 51.0412 | 1.235658 | 5.00E-05 | 0.000607 |
| Zhx1 | chr15:58047002-58076489 | 12.2938 | 15.1869 | 1.23533 | 0.00035 | 0.003088 |
| Rfx7 | chr9:72532239-72622949 | 4.81086 | 5.94259 | 1.235245 | 0.0014 | 0.009144 |
| Nadkd1 | chr15:9071259-9110487 | 9.07397 | 11.2084 | 1.235226 | 0.0021 | 0.012579 |
| Kbtbd2 | chr6:56777524-56797813 | 10.7979 | 13.3354 | 1.234999 | 0.00075 | 0.005715 |
| Cnnm2 | chr19:46761608-46878580 | 6.27517 | 7.74708 | 1.234561 | 0.0115 | 0.047055 |
| Snap91 | chr9:86765935-86880372 | 62.8659 | 77.5844 | 1.234125 | 5.00E-05 | 0.000607 |
| Ankrd13c | chr3:157947465-158006837 | 13.0473 | 16.1007 | 1.234025 | 0.00225 | 0.013268 |
| Golph3 | chr15:12321495-12351267 | 18.9889 | 23.4308 | 1.233921 | 0.00045 | 0.003815 |
| 8-Sep | chr11:53519735-53544096 | 36.7911 | 45.3871 | 1.233643 | 5.00E-05 | 0.000607 |
| Rhoq | chr17:86963110-87025401 | 6.42449 | 7.92405 | 1.233413 | 0.01065 | 0.044364 |
| Mllt4 | chr17:13716435-13905794 | 13.1724 | 16.2456 | 1.233306 | 0.0001 | 0.001096 |
| Igfbp5 | chr1:72858064-72874865 | 28.3158 | 34.9185 | 1.233181 | 5.00E-05 | 0.000607 |
| Man1a2 | chr3:100562204-100685473 | 11.0152 | 13.5777 | 1.232633 | 0.0002 | 0.001971 |
| Pias1 | chr9:62880076-62980879 | 7.88403 | 9.71571 | 1.232328 | 0.00335 | 0.018178 |
| Vwa1 | chr4:155768494-155774561 | 8.15642 | 10.0513 | 1.232318 | 0.00555 | 0.026911 |
| Zfp292 | chr4:34803109-34882948 | 3.70311 | 4.56302 | 1.232213 | 0.0011 | 0.007621 |
| Pacs1 | chr19:5133684-5273119 | 18.0067 | 22.1874 | 1.232175 | 0.00025 | 0.002352 |
| Fgf12 | chr16:28158583-28753243 | 20.3499 | 25.0668 | 1.23179 | 5.00E-05 | 0.000607 |
| Ryk | chr9:102834919-102908307 | 9.12521 | 11.2385 | 1.231588 | 0.00335 | 0.018178 |
| Camsap2 | chr1:136268122-136346104 | 17.5306 | 21.5887 | 1.231487 | 5.00E-05 | 0.000607 |
| Kcnd3 | chr3:105448189-105674002 | 15.9137 | 19.592 | 1.23114 | 5.00E-05 | 0.000607 |
| Mkln1 | chr6:31398827-31509477 | 8.16541 | 10.0519 | 1.231034 | 0.00265 | 0.01513 |
| Map3k2 | chr18:32163088-32236751 | 3.1384 | 3.86181 | 1.230503 | 0.0023 | 0.013502 |
| Kat6a | chr8:22859538-22943262 | 5.60111 | 6.89212 | 1.230492 | 0.00035 | 0.003088 |
| Zfp322a | chr13:23353103-23369202 | 7.15324 | 8.80063 | 1.2303 | 0.0019 | 0.01161 |
| Nlgn1 | chr3:25431840-26331909 | 9.69025 | 11.9205 | 1.230154 | 0.00105 | 0.007395 |
| Nlgn3 | chrX:101299178-101321350 | 14.9481 | 18.3817 | 1.229701 | 0.0006 | 0.004799 |
| Ccdc93 | chr1:121431066-121506460 | 7.26153 | 8.92899 | 1.229629 | 0.00055 | 0.004491 |
| Ash1l | chr3:88965811-89079375 | 9.18849 | 11.2976 | 1.229538 | 0.0001 | 0.001096 |
| Rsf1 | chr7:97579895-97692782 | 4.582 | 5.63272 | 1.229315 | 0.0005 | 0.004159 |
| Smcr8 | chr11:60777524-60788287 | 4.98648 | 6.12964 | 1.229252 | 0.0023 | 0.013502 |
| Sv2a | chr3:96181226-96195180 | 86.9286 | 106.854 | 1.229216 | 5.00E-05 | 0.000607 |
| Hyou1 | chr9:44379489-44392369 | 26.2105 | 32.2175 | 1.229183 | 5.00E-05 | 0.000607 |
| Diap1 | chr18:37844824-37935411 | 8.33334 | 10.2431 | 1.229171 | 0.00255 | 0.014638 |
| Ksr1 | chr11:79014800-79146354 | 19.6777 | 24.1807 | 1.228838 | 0.0002 | 0.001971 |
| Neurl1b | chr17:26414964-26446342 | 7.69649 | 9.45734 | 1.228786 | 0.0007 | 0.005435 |
| Nmt2 | chr2:3284287-3326369 | 14.8835 | 18.2851 | 1.228548 | 0.0075 | 0.033932 |
| Apba2 | chr7:64501705-64753876 | 57.7982 | 71.0017 | 1.228441 | 5.00E-05 | 0.000607 |
| Palm2 | chr4:57568247-57717128 | 4.77559 | 5.86629 | 1.228391 | 0.00205 | 0.012328 |
| Mbnl2 | chr14:120275668-120431698 | 34.4206 | 42.28 | 1.228334 | 5.00E-05 | 0.000607 |
| Xiap | chrX:42067835-42109664 | 9.6283 | 11.8256 | 1.228213 | 0.0002 | 0.001971 |
| Hnrnpr | chr4:136310975-136340678 | 26.8906 | 33.0237 | 1.228076 | 0.0001 | 0.001096 |
| Adam9 | chr8:24949610-25016922 | 10.7687 | 13.2223 | 1.227846 | 0.0009 | 0.006615 |
| Stxbp5l | chr16:37107309-37384958 | 11.1864 | 13.7274 | 1.227151 | 5.00E-05 | 0.000607 |
| Atrn | chr2:130906495-131030326 | 19.6505 | 24.1074 | 1.226808 | 5.00E-05 | 0.000607 |
| Otud6b | chr4:14809504-14826587 | 14.5687 | 17.8713 | 1.226691 | 0.00065 | 0.005126 |
| Mpp5 | chr12:78748946-78840713 | 6.35686 | 7.79781 | 1.226676 | 0.003 | 0.016689 |
| Tmod2 | chr9:75565621-75611325 | 38.4409 | 47.1507 | 1.226576 | 0.00015 | 0.001558 |
| Plxna2 | chr1:194619828-194816868 | 11.3367 | 13.9048 | 1.22653 | 5.00E-05 | 0.000607 |
| 6030458C11Rik | chr15:12808176-12824657 | 11.475 | 14.0719 | 1.226309 | 0.0013 | 0.008656 |
| Robo2 | chr16:73892305-74410912 | 7.48896 | 9.18298 | 1.226202 | 0.00025 | 0.002352 |
| Arid4b | chr13:14063791-14199603 | 7.17782 | 8.80009 | 1.226012 | 0.00125 | 0.008413 |
| Syngr1 | chr15:80091333-80119501 | 104.422 | 128.012 | 1.22591 | 0.00045 | 0.003815 |
| Rab23 | chr1:33719895-33742564 | 6.59049 | 8.07928 | 1.2259 | 0.00655 | 0.030505 |
| Adipor2 | chr6:119353149-119417483 | 14.5367 | 17.8198 | 1.225849 | 0.0003 | 0.002726 |
| Cmip | chr8:117257018-117461505 | 60.7208 | 74.4194 | 1.2256 | 0.0001 | 0.001096 |
| D130043K22Rik | chr13:24845130-24901270 | 7.27395 | 8.91362 | 1.225417 | 0.0027 | 0.01535 |
| Fam65b | chr13:24614609-24733806 | 15.5887 | 19.1025 | 1.225407 | 0.0004 | 0.00346 |
| Atrx | chrX:105797614-105929372 | 15.3338 | 18.786 | 1.225137 | 5.00E-05 | 0.000607 |
| Tulp4 | chr17:6106829-6240637 | 17.3191 | 21.2158 | 1.224994 | 5.00E-05 | 0.000607 |
| Vprbp | chr9:106821975-106880992 | 4.37538 | 5.35938 | 1.224895 | 0.01035 | 0.043473 |
| Dhx9 | chr1:153455757-153487660 | 23.7806 | 29.1194 | 1.224502 | 5.00E-05 | 0.000607 |
| Agpat3 | chr10:78271562-78351700 | 33.3656 | 40.8256 | 1.223584 | 5.00E-05 | 0.000607 |
| Zcchc12 | chrX:36195903-36199158 | 27.2377 | 33.3206 | 1.223326 | 0.0004 | 0.00346 |
| Polr2a | chr11:69734409-69758223 | 14.6965 | 17.9785 | 1.223318 | 0.00025 | 0.002352 |
| Cnep1r1 | chr8:88118758-88135197 | 14.6544 | 17.9227 | 1.223025 | 0.0088 | 0.038422 |
| Chtf8 | chr8:106883862-106893593 | 24.1484 | 29.5305 | 1.222876 | 0.0003 | 0.002726 |
| Derl2 | chr11:71007444-71019263 | 7.91357 | 9.67369 | 1.222418 | 0.00635 | 0.029796 |
| Pdxk | chr10:78436746-78464948 | 25.9955 | 31.7748 | 1.222319 | 0.0001 | 0.001096 |
| Pias2 | chr18:77065207-77155708 | 13.2276 | 16.1629 | 1.221907 | 0.00135 | 0.008911 |
| Slc32a1 | chr2:158610757-158615747 | 14.003 | 17.1102 | 1.221895 | 0.00165 | 0.010418 |
| Rap1gap2 | chr11:74383482-74590158 | 24.6166 | 30.0774 | 1.221834 | 5.00E-05 | 0.000607 |
| Chd2 | chr7:73426651-73558395 | 8.30085 | 10.1363 | 1.221116 | 0.00085 | 0.0063 |
| Camk4 | chr18:32939040-33195767 | 9.4902 | 11.5882 | 1.22107 | 5.00E-05 | 0.000607 |
| Pitpnm3 | chr11:72047527-72135889 | 9.32922 | 11.3912 | 1.221024 | 0.00045 | 0.003815 |
| Pcdh8 | chr14:79766771-79771312 | 11.4324 | 13.9591 | 1.221012 | 0.0016 | 0.010187 |
| Prpf4b | chr13:34875493-34902878 | 21.5574 | 26.3212 | 1.220982 | 0.0002 | 0.001971 |
| Daam1 | chr12:71831077-71992367 | 12.573 | 15.3473 | 1.220655 | 0.0002 | 0.001971 |
| Gfpt1 | chr6:87042845-87092207 | 9.08384 | 11.0876 | 1.220585 | 0.00055 | 0.004491 |
| Frmd5 | chr2:121545528-121807057 | 6.88542 | 8.4041 | 1.220565 | 0.00605 | 0.02877 |
| Nr3c2 | chr8:76902507-77243639 | 13.7705 | 16.8052 | 1.220377 | 0.0006 | 0.004799 |
| Rimbp2 | chr5:128760400-128953362 | 20.1342 | 24.5681 | 1.220217 | 0.0002 | 0.001971 |
| Myrip | chr9:120304072-120474834 | 11.2663 | 13.747 | 1.220188 | 0.0008 | 0.006001 |
| Ubqln2 | chrX:153498231-153501558 | 49.9939 | 61.0009 | 1.220167 | 0.0001 | 0.001096 |
| Rai1 | chr11:60105012-60220604 | 9.96425 | 12.1566 | 1.220022 | 0.0035 | 0.018791 |
| Matr3 | chr18:35562157-35592045 | 73.2521 | 89.3545 | 1.219822 | 5.00E-05 | 0.000607 |
| Elovl4 | chr9:83778691-83806305 | 17.1888 | 20.9638 | 1.21962 | 0.00075 | 0.005715 |
| 2310022B05Rik | chr8:124635755-124663369 | 25.1468 | 30.6633 | 1.219372 | 0.00025 | 0.002352 |
| Cd200 | chr16:45382134-45409053 | 42.6552 | 52.0073 | 1.219249 | 0.00015 | 0.001558 |
| Usp33 | chr3:152346477-152393614 | 25.986 | 31.6824 | 1.21921 | 0.00015 | 0.001558 |
| Azi2 | chr9:118040498-118063907 | 17.1688 | 20.9271 | 1.218903 | 0.0005 | 0.004159 |
| Akap9 | chr5:3928185-4080204 | 4.80071 | 5.85037 | 1.218647 | 0.00025 | 0.002352 |
| Atxn3 | chr12:101918900-101958243 | 5.92352 | 7.21765 | 1.218473 | 0.00705 | 0.032226 |
| Auts2 | chr5:131437681-132542343 | 14.666 | 17.8684 | 1.218355 | 0.0002 | 0.001971 |
| Setd7 | chr3:51515317-51567116 | 19.1889 | 23.3787 | 1.218345 | 0.0002 | 0.001971 |
| Kctd12 | chr14:102976580-102982637 | 16.88 | 20.564 | 1.218246 | 0.0001 | 0.001096 |
| Stxbp1 | chr2:32787606-32847237 | 147.497 | 179.679 | 1.218187 | 0.00075 | 0.005715 |
| Dock4 | chr12:40446052-40846488 | 18.7331 | 22.8202 | 1.218175 | 0.0001 | 0.001096 |
| Cnr1 | chr4:33924631-33948831 | 27.0333 | 32.9301 | 1.218131 | 0.0001 | 0.001096 |
| Sel1l3 | chr5:53107082-53213452 | 13.0148 | 15.851 | 1.217921 | 0.00065 | 0.005126 |
| Braf | chr6:39603236-39725463 | 12.5264 | 15.2559 | 1.2179 | 0.00015 | 0.001558 |
| Itpr1 | chr6:108213095-108551116 | 27.0949 | 32.9966 | 1.217816 | 0.00025 | 0.002352 |
| D630045J12Rik | chr6:38048482-38254009 | 6.50719 | 7.92446 | 1.217801 | 0.0001 | 0.001096 |
| Hipk3 | chr2:104426481-104494489 | 8.1896 | 9.97326 | 1.217796 | 0.0007 | 0.005435 |
| Golga3 | chr5:110176700-110223155 | 10.0212 | 12.203 | 1.217718 | 0.00105 | 0.007395 |
| Vcl | chr14:20929432-21033673 | 11.6254 | 14.1553 | 1.217618 | 0.0007 | 0.005435 |
| Slc4a8 | chr15:100761746-100823971 | 8.98833 | 10.9421 | 1.217367 | 5.00E-05 | 0.000607 |
| Slmap | chr14:26413174-26533740 | 15.1509 | 18.4427 | 1.217268 | 0.0003 | 0.002726 |
| Cep350 | chr1:155844963-155973255 | 4.04533 | 4.92299 | 1.216956 | 0.0005 | 0.004159 |
| Magi3 | chr3:104013264-104220406 | 6.41929 | 7.81183 | 1.216931 | 0.002 | 0.012075 |
| Nhsl2 | chrX:101849384-102092055 | 5.50136 | 6.6945 | 1.216881 | 0.0013 | 0.008656 |
| St8sia1 | chr6:142814230-142964452 | 5.2825 | 6.42663 | 1.216589 | 0.0014 | 0.009144 |
| Trio | chr15:27730648-28025848 | 14.6523 | 17.8221 | 1.216335 | 5.00E-05 | 0.000607 |
| Bmi1 | chr2:18677017-18686629 | 10.924 | 13.2817 | 1.215828 | 0.00275 | 0.015584 |
| Gpr56 | chr8:94977108-95014208 | 19.4487 | 23.6391 | 1.215459 | 0.0003 | 0.002726 |
| Rxra | chr2:27677200-27763319 | 7.05311 | 8.57105 | 1.215216 | 0.0021 | 0.012579 |
| Abca3 | chr17:24352045-24414513 | 12.341 | 14.9965 | 1.215177 | 0.0032 | 0.01758 |
| Apc | chr18:34207774-34322190 | 20.6917 | 25.1435 | 1.215149 | 0.0002 | 0.001971 |
| Fam219a | chr4:41517436-41569527 | 45.3069 | 55.0486 | 1.215016 | 0.00085 | 0.0063 |
| Cltc | chr11:86694652-86757492 | 66.4402 | 80.7238 | 1.214984 | 0.00025 | 0.002352 |
| Fam171a2 | chr11:102436980-102447663 | 12.805 | 15.557 | 1.214916 | 0.0026 | 0.014885 |
| Ak4 | chr4:101419288-101467771 | 18.6082 | 22.6059 | 1.214835 | 0.0004 | 0.00346 |
| Mblac2 | chr13:81711416-81753275 | 7.481 | 9.08749 | 1.214743 | 0.0083 | 0.0366 |
| Nell1 | chr7:49975349-50863289 | 18.9088 | 22.9659 | 1.214561 | 0.00135 | 0.008911 |
| Fam120c | chrX:151344222-151474134 | 9.64297 | 11.7114 | 1.214501 | 0.0003 | 0.002726 |
| Snx6 | chr12:54746356-54795662 | 16.7192 | 20.3 | 1.214173 | 0.0065 | 0.030326 |
| Eif5a2 | chr3:28781310-28798846 | 6.58514 | 7.99509 | 1.214111 | 0.00475 | 0.023714 |
| Sacm1l | chr9:123529881-123592598 | 19.9917 | 24.2664 | 1.213824 | 0.0006 | 0.004799 |
| Whsc1 | chr5:33843111-33897966 | 12.2767 | 14.9011 | 1.213771 | 0.0003 | 0.002726 |
| Zyg11b | chr4:108227754-108301090 | 18.6293 | 22.6075 | 1.213545 | 0.00015 | 0.001558 |
| Ptprg | chr14:11553552-12242039 | 9.52599 | 11.5591 | 1.213428 | 0.00045 | 0.003815 |
| Cadm4 | chr7:24482022-24504533 | 61.4237 | 74.5308 | 1.213388 | 0.0002 | 0.001971 |
| Ccnd2 | chr6:127125708-127212419 | 12.6592 | 15.3595 | 1.213307 | 0.0003 | 0.002726 |
| Pam | chr1:97821093-98095632 | 39.9986 | 48.5299 | 1.21329 | 0.00015 | 0.001558 |
| Slc23a2 | chr2:132052495-132145108 | 21.8317 | 26.4874 | 1.213254 | 0.00025 | 0.002352 |
| Hipk1 | chr3:103739814-103791275 | 12.2816 | 14.8997 | 1.213173 | 0.0001 | 0.001096 |
| Samd8 | chr14:21750530-21798725 | 10.0018 | 12.1337 | 1.213152 | 0.0003 | 0.002726 |
| Snx13 | chr12:35047188-35147477 | 8.76912 | 10.6373 | 1.213041 | 0.00085 | 0.0063 |
| Smad2 | chr18:76241700-76311747 | 3.43009 | 4.15514 | 1.211379 | 0.0069 | 0.031704 |
| Bicd2 | chr13:49341548-49387025 | 18.7512 | 22.7004 | 1.210611 | 0.0002 | 0.001971 |
| Pknox2 | chr9:36890982-37147314 | 10.9348 | 13.2338 | 1.210246 | 0.00365 | 0.01938 |
| Suz12 | chr11:79993105-80034123 | 7.99219 | 9.67059 | 1.210005 | 0.00475 | 0.023714 |
| Samd4b | chr7:28399521-28436191 | 13.0399 | 15.777 | 1.209902 | 0.0006 | 0.004799 |
| Rnf150 | chr8:82863355-83091271 | 7.8439 | 9.48699 | 1.209474 | 0.00025 | 0.002352 |
| Pcm1 | chr8:41239758-41334087 | 9.35395 | 11.3127 | 1.209404 | 0.00025 | 0.002352 |
| Slc12a2 | chr18:57878677-57946821 | 8.22901 | 9.95201 | 1.209381 | 0.0013 | 0.008656 |
| Nyap1 | chr5:137730962-137739998 | 17.1911 | 20.7867 | 1.209155 | 0.0006 | 0.004799 |
| Mtpn | chr6:35508823-35539888 | 56.0625 | 67.7844 | 1.209086 | 0.0001 | 0.001096 |
| Atic | chr1:71557155-71579403 | 10.6543 | 12.8808 | 1.208977 | 0.01 | 0.042388 |
| Gria2 | chr3:80684935-80802791 | 101.222 | 122.365 | 1.208878 | 0.0036 | 0.019163 |
| Atm | chr9:53437121-53536671 | 2.94447 | 3.55927 | 1.208798 | 0.00465 | 0.023325 |
| Pip5k1a | chr3:95059595-95106858 | 18.8449 | 22.7792 | 1.208773 | 0.0013 | 0.008656 |
| Scaf11 | chr15:96411697-96460843 | 7.02016 | 8.48521 | 1.208692 | 0.00105 | 0.007395 |
| Dennd4a | chr9:64811010-64919667 | 5.4471 | 6.58308 | 1.208548 | 0.00195 | 0.011841 |
| Ccdc6 | chr10:70097120-70193200 | 11.3885 | 13.7572 | 1.207991 | 0.00065 | 0.005126 |
| Ireb2 | chr9:54863754-54912534 | 11.477 | 13.8633 | 1.20792 | 0.00045 | 0.003815 |
| Pde1a | chr2:79834452-80129412 | 36.952 | 44.6304 | 1.207794 | 0.00015 | 0.001558 |
| Igdcc4 | chr9:65101494-65137943 | 8.9547 | 10.8144 | 1.207679 | 0.00115 | 0.007885 |
| Tspyl3 | chr2:153222369-153225441 | 9.38013 | 11.3276 | 1.207617 | 0.01085 | 0.044968 |
| Peg3 | chr7:6705959-6730419 | 25.1259 | 30.3408 | 1.207551 | 0.0004 | 0.00346 |
| Klhl9 | chr4:88718291-88722508 | 20.2013 | 24.3938 | 1.207536 | 0.0005 | 0.004159 |
| Grlf1 | chr7:16494472-16614993 | 19.1654 | 23.1424 | 1.207509 | 0.0002 | 0.001971 |
| Zdhhc9 | chrX:48171970-48208702 | 13.065 | 15.7752 | 1.20744 | 0.0043 | 0.021953 |
| N4bp1 | chr8:86841138-86885258 | 8.86834 | 10.7075 | 1.207385 | 0.0008 | 0.006001 |
| Gpc5 | chr14:115092214-116525192 | 12.5152 | 15.1106 | 1.20738 | 0.0049 | 0.02436 |
| Atp2a2 | chr5:122453512-122502225 | 110.334 | 133.175 | 1.207017 | 0.00175 | 0.010881 |
| Rcan3 | chr4:135412308-135433805 | 5.2124 | 6.29029 | 1.206793 | 0.01165 | 0.047522 |
| Rab3b | chr4:108879069-108972077 | 17.4127 | 21.0126 | 1.20674 | 0.0017 | 0.010645 |
| Nckap1 | chr2:80501294-80580965 | 109.938 | 132.645 | 1.206544 | 0.00045 | 0.003815 |
| Pten | chr19:32757576-32826160 | 17.6083 | 21.2415 | 1.206335 | 0.0003 | 0.002726 |
| Htr1a | chr13:105443692-105448133 | 8.21034 | 9.90383 | 1.206263 | 0.00495 | 0.02454 |
| Gpr158 | chr2:21367566-21830542 | 13.3346 | 16.0823 | 1.206058 | 0.0002 | 0.001971 |
| Atn1 | chr6:124742543-124756487 | 30.0741 | 36.2691 | 1.205991 | 0.00025 | 0.002352 |
| Aldh6a1 | chr12:84409067-84450950 | 14.6295 | 17.6314 | 1.205195 | 0.00565 | 0.027333 |
| Mcf2l | chr8:12915892-13020509 | 36.462 | 43.9429 | 1.20517 | 0.00015 | 0.001558 |
| Fam78b | chr1:167001416-167091302 | 6.66791 | 8.03491 | 1.205012 | 0.0081 | 0.035882 |
| Nwd1 | chr8:72646710-72714748 | 7.87931 | 9.49458 | 1.205001 | 0.00085 | 0.0063 |
| Rnf44 | chr13:54679398-54693960 | 27.8802 | 33.5929 | 1.204902 | 0.0003 | 0.002726 |
| Tspyl4 | chr10:34297420-34301320 | 115.037 | 138.6 | 1.20483 | 0.00095 | 0.006869 |
| Cept1 | chr3:106481985-106547761 | 13.2993 | 16.0196 | 1.204545 | 0.00465 | 0.023325 |
| 2810403A07Rik | chr3:88685793-88712933 | 20.9687 | 25.2529 | 1.204314 | 0.0011 | 0.007621 |
| Mgat4a | chr1:37439339-37536259 | 7.22929 | 8.70592 | 1.204257 | 0.00155 | 0.009917 |
| Slc6a11 | chr6:114131240-114249886 | 30.1549 | 36.3106 | 1.204136 | 0.0003 | 0.002726 |
| Smarcad1 | chr6:65042666-65116049 | 6.28469 | 7.56761 | 1.204134 | 0.00995 | 0.042244 |
| Kif21a | chr15:90933275-91049948 | 26.2896 | 31.6557 | 1.204115 | 0.00045 | 0.003815 |
| Nalcn | chr14:123276640-123627144 | 12.2906 | 14.7982 | 1.204026 | 0.0005 | 0.004159 |
| Pnrc2 | chr4:135870925-135873846 | 25.3975 | 30.5786 | 1.204 | 0.0021 | 0.012579 |
| Lrp1 | chr10:127538157-127621148 | 32.4383 | 39.0535 | 1.203932 | 0.0005 | 0.004159 |
| Ccnt2 | chr1:127774163-127804837 | 18.0299 | 21.7046 | 1.203811 | 0.0006 | 0.004799 |
| Prrt3 | chr6:113494094-113501818 | 9.7194 | 11.7 | 1.203778 | 0.009 | 0.039086 |
| Dmd | chrX:82948869-85205050 | 3.38878 | 4.079 | 1.203678 | 0.00155 | 0.009917 |
| Itm2a | chrX:107397194-107403360 | 29.0569 | 34.971 | 1.203535 | 0.00215 | 0.012828 |
| Jam2 | chr16:84774122-84826375 | 7.0354 | 8.46593 | 1.203333 | 0.00725 | 0.032942 |
| Srrm4 | chr5:116438721-116591817 | 9.59973 | 11.5514 | 1.203305 | 0.0005 | 0.004159 |
| Cerk | chr15:86139100-86186141 | 13.4193 | 16.1475 | 1.203304 | 0.00085 | 0.0063 |
| Napb | chr2:148694656-148732420 | 100.287 | 120.654 | 1.203087 | 0.00075 | 0.005715 |
| Sptbn1 | chr11:30099394-30219772 | 94.3372 | 113.448 | 1.20258 | 0.00355 | 0.018973 |
| Lrp6 | chr6:134446477-134566913 | 5.87639 | 7.06681 | 1.202577 | 0.00095 | 0.006869 |
| Zmym1 | chr4:127047093-127061132 | 14.4552 | 17.383 | 1.202543 | 0.0012 | 0.008175 |
| Sv2b | chr7:75114894-75331419 | 75.1875 | 90.4153 | 1.202531 | 0.001 | 0.007133 |
| Pclo | chr5:14514917-14863459 | 13.1019 | 15.7527 | 1.202322 | 0.00015 | 0.001558 |
| Grb10 | chr11:11930498-12037420 | 8.14625 | 9.79194 | 1.202018 | 0.00435 | 0.022102 |
| Chst11 | chr10:82985496-83195891 | 14.216 | 17.0857 | 1.201864 | 0.0005 | 0.004159 |
| Dip2a | chr10:76263048-76345291 | 12.717 | 15.2834 | 1.201809 | 0.00055 | 0.004491 |
| Tmem106b | chr6:13069758-13089269 | 24.0448 | 28.8909 | 1.201545 | 0.00045 | 0.003815 |
| Dhcr7 | chr7:143823166-143848410 | 14.6703 | 17.6251 | 1.201414 | 0.00465 | 0.023325 |
| Manf | chr9:106887414-106891938 | 19.6463 | 23.6012 | 1.201305 | 0.0038 | 0.020026 |
| Tmem65 | chr15:58782268-58823427 | 14.0232 | 16.8391 | 1.200803 | 0.0022 | 0.013067 |
| Myh10 | chr11:68691914-68816624 | 25.7637 | 30.9212 | 1.200185 | 0.00025 | 0.002352 |
| Ankrd33b | chr15:31291478-31367759 | 9.9199 | 11.9038 | 1.199992 | 0.0015 | 0.009667 |
| Sec23a | chr12:58958383-59012017 | 22.4058 | 26.8844 | 1.199886 | 0.00055 | 0.004491 |
| Ccdc50 | chr16:27388976-27452218 | 8.0346 | 9.6398 | 1.199786 | 0.00185 | 0.011363 |
| Cers6 | chr2:68861556-69111290 | 7.66842 | 9.1994 | 1.199647 | 0.01205 | 0.048798 |
| Taok1 | chr11:77529161-77607815 | 9.59285 | 11.5064 | 1.199477 | 0.0006 | 0.004799 |
| Camk1g | chr1:193346345-193370282 | 13.7362 | 16.4762 | 1.199473 | 0.00945 | 0.040592 |
| Fmn2 | chr1:174501824-174822729 | 8.94496 | 10.7241 | 1.198899 | 0.00175 | 0.010881 |
| Spast | chr17:74338986-74391113 | 11.2431 | 13.4757 | 1.198575 | 0.00185 | 0.011363 |
| Prox1 | chr1:190121776-190170680 | 10.4989 | 12.5831 | 1.198516 | 0.00325 | 0.017763 |
| Stag2 | chrX:42149411-42277175 | 7.58282 | 9.08654 | 1.198306 | 0.00385 | 0.02021 |
| Ppm1f | chr16:16896468-16927375 | 19.9233 | 23.8737 | 1.19828 | 0.0006 | 0.004799 |
| Cadps2 | chr6:23262773-23839421 | 16.6713 | 19.9715 | 1.197957 | 0.0008 | 0.006001 |
| 3110047P20Rik | chr5:63649102-63810543 | 4.21212 | 5.04565 | 1.197888 | 0.00655 | 0.030505 |
| Scn1a | chr2:66270781-66440837 | 8.71892 | 10.4439 | 1.197843 | 0.00095 | 0.006869 |
| Rock2 | chr12:16894977-16988274 | 21.061 | 25.2256 | 1.19774 | 0.0003 | 0.002726 |
| Caprin1 | chr2:103762944-103797640 | 41.1341 | 49.2545 | 1.197413 | 0.0011 | 0.007621 |
| Xpr1 | chr1:155275656-155417444 | 11.6839 | 13.99 | 1.197374 | 0.0007 | 0.005435 |
| Wnk2 | chr13:49036301-49148014 | 11.8978 | 14.2449 | 1.197272 | 0.00045 | 0.003815 |
| Pik3ca | chr3:32436150-32468486 | 5.53186 | 6.62267 | 1.197187 | 0.0029 | 0.016217 |
| Bzw1 | chr1:58393135-58406548 | 42.2941 | 50.6219 | 1.196902 | 0.00035 | 0.003088 |
| Rab9b | chrX:136858150-136868540 | 8.20203 | 9.81599 | 1.196776 | 0.0116 | 0.047355 |
| Tars | chr15:11383662-11399658 | 13.8655 | 16.5878 | 1.196336 | 0.00695 | 0.031865 |
| Pdia6 | chr12:17266594-17324730 | 67.4811 | 80.7214 | 1.196208 | 0.00025 | 0.002352 |
| Col5a1 | chr2:27886424-28039510 | 7.509 | 8.98207 | 1.196174 | 0.00185 | 0.011363 |
| Lclat1 | chr17:73107984-73243366 | 9.60809 | 11.4928 | 1.196159 | 0.0036 | 0.019163 |
| Katnal1 | chr5:148871583-148928647 | 12.0272 | 14.3842 | 1.195972 | 0.00115 | 0.007885 |
| Kcnh1 | chr1:192190871-192510158 | 6.82052 | 8.15523 | 1.19569 | 0.00315 | 0.017359 |
| Slit2 | chr5:47983154-48305778 | 4.80141 | 5.73919 | 1.195313 | 0.00605 | 0.02877 |
| Iws1 | chr18:32067733-32104331 | 3.8877 | 4.64642 | 1.195159 | 0.00615 | 0.029076 |
| Kcna2 | chr3:107101566-107115005 | 13.1555 | 15.7219 | 1.195082 | 0.0003 | 0.002726 |
| Rad21 | chr15:51962603-51991760 | 24.8015 | 29.6364 | 1.194944 | 0.00115 | 0.007885 |
| Polr3e | chr7:120917743-120947432 | 10.4393 | 12.473 | 1.194812 | 0.00465 | 0.023325 |
| Sh3bgrl | chrX:109095406-109162467 | 30.0474 | 35.8987 | 1.194736 | 0.00095 | 0.006869 |
| Psmd11 | chr11:80428614-80472133 | 24.714 | 29.5252 | 1.194675 | 0.0051 | 0.025119 |
| Syt17 | chr7:118381855-118443552 | 34.4428 | 41.137 | 1.194357 | 0.0036 | 0.019163 |
| Sh3rf3 | chr10:58813358-59138916 | 6.33398 | 7.56389 | 1.194176 | 0.0087 | 0.038095 |
| Ttbk1 | chr17:46442447-46487675 | 7.47011 | 8.91991 | 1.19408 | 0.0025 | 0.014398 |
| Cpne2 | chr8:94533027-94570529 | 22.654 | 27.0479 | 1.193957 | 0.00355 | 0.018973 |
| Vopp1 | chr6:57752263-57825125 | 25.4362 | 30.3668 | 1.193842 | 0.00135 | 0.008911 |
| Klhl24 | chr16:20097553-20127744 | 8.0348 | 9.58968 | 1.193518 | 0.00215 | 0.012828 |
| Ttc17 | chr2:94300765-94406689 | 8.47916 | 10.1193 | 1.193432 | 0.00605 | 0.02877 |
| Tle3 | chr9:61372365-61418497 | 7.64944 | 9.12789 | 1.193276 | 0.0066 | 0.030711 |
| Tcf4 | chr18:69344491-69687967 | 41.6986 | 49.7555 | 1.193218 | 0.0011 | 0.007621 |
| Ubfd1 | chr7:122067197-122082199 | 11.4953 | 13.7138 | 1.192992 | 0.0024 | 0.013943 |
| Gpd2 | chr2:57237677-57370719 | 26.6128 | 31.7463 | 1.192896 | 0.00055 | 0.004491 |
| Sulf2 | chr2:166073898-166155683 | 34.9983 | 41.7378 | 1.192566 | 0.0007 | 0.005435 |
| Mfsd6 | chr1:52656304-52727318 | 19.9872 | 23.8329 | 1.192408 | 0.0007 | 0.005435 |
| Chordc1 | chr9:18292266-18314000 | 24.2389 | 28.9025 | 1.192401 | 0.0027 | 0.01535 |
| Mllt3 | chr4:87769924-88033407 | 6.35809 | 7.58103 | 1.192344 | 0.01235 | 0.049841 |
| Fam117b | chr1:59913005-59985348 | 12.2727 | 14.633 | 1.192321 | 0.00125 | 0.008413 |
| Asic1 | chr15:99670717-99701128 | 12.0398 | 14.3552 | 1.192312 | 0.00435 | 0.022102 |
| Spag9 | chr11:93996090-94126082 | 34.6796 | 41.3472 | 1.192263 | 0.0003 | 0.002726 |
| Dlgap3 | chr4:127169270-127237022 | 32.9424 | 39.2744 | 1.192214 | 0.00055 | 0.004491 |
| Pcyt1a | chr16:32430920-32475065 | 7.03909 | 8.38863 | 1.191721 | 0.01035 | 0.043473 |
| Clmn | chr12:104763113-104865076 | 11.3344 | 13.5043 | 1.191444 | 0.0002 | 0.001971 |
| Osbpl1a | chr18:12755311-12941841 | 53.7654 | 64.044 | 1.191175 | 0.00075 | 0.005715 |
| Fbxo42 | chr4:141147921-141204062 | 6.81994 | 8.12328 | 1.191107 | 0.0057 | 0.027512 |
| Ntm | chr9:28995963-29963129 | 35.7389 | 42.5679 | 1.19108 | 0.00155 | 0.009917 |
| Numa1 | chr7:101969842-102014959 | 15.8332 | 18.8582 | 1.191054 | 0.0004 | 0.00346 |
| Scd2 | chr19:44293675-44306862 | 172.491 | 205.331 | 1.190387 | 0.0098 | 0.041724 |
| Tns1 | chr1:73910230-74124447 | 4.98788 | 5.93709 | 1.190303 | 0.0041 | 0.021197 |
| Atxn1 | chr13:45549755-45964991 | 7.68961 | 9.15157 | 1.190121 | 0.00105 | 0.007395 |
| Arl4c | chr1:88698225-88702191 | 9.48361 | 11.2828 | 1.189716 | 0.00905 | 0.039271 |
| A830010M20Rik | chr5:107497744-107512556 | 15.257 | 18.1442 | 1.189238 | 0.00105 | 0.007395 |
| Pak2 | chr16:32017862-32079273 | 9.81194 | 11.6687 | 1.189235 | 0.0068 | 0.031381 |
| 1700025G04Rik | chr1:151884523-152090320 | 9.77909 | 11.629 | 1.18917 | 0.0011 | 0.007621 |
| Unc80 | chr1:66468446-66699148 | 19.2201 | 22.8551 | 1.189125 | 0.00075 | 0.005715 |
| Sarm1 | chr11:78472329-78497754 | 6.9878 | 8.30937 | 1.189125 | 0.01005 | 0.042515 |
| Rap2a | chr14:120478460-120507192 | 28.2177 | 33.5532 | 1.189083 | 0.00095 | 0.006869 |
| Ptn | chr6:36715662-36811361 | 65.1294 | 77.4381 | 1.188988 | 0.0005 | 0.004159 |
| Mpped1 | chr15:83780022-83858474 | 77.7929 | 92.4836 | 1.188844 | 0.0006 | 0.004799 |
| Dhcr24 | chr4:106561037-106589113 | 18.4722 | 21.9514 | 1.188348 | 0.00115 | 0.007885 |
| Peg13 | chr15:72589619-73061204 | 44.6872 | 53.095 | 1.188148 | 0.0068 | 0.031381 |
| Lnp | chr2:74514836-74578948 | 6.69921 | 7.95822 | 1.187934 | 0.0023 | 0.013502 |
| Prex1 | chr2:166566344-166713832 | 13.2658 | 15.7578 | 1.187851 | 0.00085 | 0.0063 |
| Ubr3 | chr2:69897245-70024013 | 16.8489 | 20.0136 | 1.187828 | 0.0009 | 0.006615 |
| Hiatl1 | chr13:65065029-65112982 | 10.0495 | 11.9339 | 1.187512 | 0.0107 | 0.044502 |
| Pds5b | chr5:150673826-150810669 | 12.7045 | 15.0846 | 1.187343 | 0.00105 | 0.007395 |
| Naa30 | chr14:49172226-49191031 | 7.70557 | 9.14787 | 1.187176 | 0.01025 | 0.043155 |
| Bcl6 | chr16:23965051-23988612 | 14.8301 | 17.6031 | 1.186985 | 0.00465 | 0.023325 |
| Dscam | chr16:96592078-97170735 | 7.95547 | 9.44073 | 1.186697 | 0.00315 | 0.017359 |
| Epha5 | chr5:84054764-84417382 | 7.07433 | 8.39425 | 1.186579 | 0.0037 | 0.019616 |
| Arap2 | chr5:62602445-62766177 | 6.91815 | 8.20755 | 1.186379 | 0.00315 | 0.017359 |
| Dab2ip | chr2:35549533-35730994 | 14.4439 | 17.1358 | 1.186369 | 0.0018 | 0.011147 |
| Wdtc1 | chr4:133292465-133339315 | 15.115 | 17.9307 | 1.186285 | 0.00185 | 0.011363 |
| Naa50 | chr16:44139808-44163364 | 13.8483 | 16.4235 | 1.185958 | 0.00225 | 0.013268 |
| Fzd3 | chr14:65192440-65262463 | 5.94797 | 7.05361 | 1.185885 | 0.0014 | 0.009144 |
| Csnk1a1 | chr18:61555581-61588299 | 64.1214 | 76.0009 | 1.185266 | 0.00085 | 0.0063 |
| Anks1b | chr10:89873508-90972984 | 44.1414 | 52.3172 | 1.185218 | 0.00205 | 0.012328 |
| Dhx15 | chr5:52150208-52190519 | 27.0292 | 32.0352 | 1.185207 | 0.00235 | 0.013705 |
| Rdx | chr9:52002060-52088738 | 15.9074 | 18.8471 | 1.184801 | 0.0022 | 0.013067 |
| Nhsl1 | chr10:18407674-18533891 | 7.4169 | 8.78727 | 1.184763 | 0.0052 | 0.025505 |
| Clip2 | chr5:134489385-134552434 | 26.7466 | 31.6789 | 1.184408 | 0.001 | 0.007133 |
| Rgl1 | chr1:152517529-152625111 | 21.6342 | 25.6173 | 1.184111 | 0.0011 | 0.007621 |
| Mtus2 | chr5:147957319-148316065 | 5.44895 | 6.44941 | 1.183606 | 0.0065 | 0.030326 |
| Smek1 | chr12:101039408-101088927 | 8.9083 | 10.5428 | 1.183481 | 0.0096 | 0.04107 |
| Akap2 | chr4:57845247-57896984 | 9.69685 | 11.4741 | 1.183281 | 0.00275 | 0.015584 |
| Clasp1 | chr1:118389057-118609462 | 11.6595 | 13.7958 | 1.183224 | 0.0014 | 0.009144 |
| Trp53bp2 | chr1:182409166-182462436 | 8.13624 | 9.62602 | 1.183104 | 0.01195 | 0.048485 |
| Sel1l | chr12:91806042-91849157 | 28.3792 | 33.5715 | 1.182961 | 0.00075 | 0.005715 |
| Camta1 | chr4:151059522-151861768 | 33.6879 | 39.8222 | 1.182092 | 0.0019 | 0.01161 |
| Ncoa1 | chr12:4247361-4477182 | 16.7216 | 19.7647 | 1.181986 | 0.0012 | 0.008175 |
| Csnk1e | chr15:79417851-79442057 | 29.4631 | 34.8224 | 1.181899 | 0.0021 | 0.012579 |
| Xpo1 | chr11:23256040-23297597 | 20.0289 | 23.6697 | 1.181777 | 0.00125 | 0.008413 |
| Fam19a2 | chr10:123264075-123741204 | 12.643 | 14.9382 | 1.181539 | 0.00415 | 0.021362 |
| Eif1a | chr18:46597703-46610225 | 14.1426 | 16.7022 | 1.180985 | 0.00925 | 0.039894 |
| Rmnd5a | chr6:71388633-71440637 | 19.7588 | 23.3299 | 1.180735 | 0.0008 | 0.006001 |
| Kif3b | chr2:153291415-153333389 | 13.6768 | 16.1456 | 1.18051 | 0.00195 | 0.011841 |
| Rapgef2 | chr3:79062528-79145875 | 21.2514 | 25.0822 | 1.180261 | 0.00185 | 0.011363 |
| Stk39 | chr2:68210446-68471981 | 18.078 | 21.3301 | 1.179893 | 0.0036 | 0.019163 |
| Slc2a3 | chr6:122727808-122742745 | 44.3226 | 52.2899 | 1.179757 | 0.0008 | 0.006001 |
| Lrrc8a | chr2:30237768-30263790 | 11.149 | 13.1492 | 1.179406 | 0.00685 | 0.031529 |
| D430041D05Rik | chr2:104143074-104410334 | 17.8326 | 21.0286 | 1.179222 | 0.0013 | 0.008656 |
| Arnt2 | chr7:84246278-84409959 | 45.5124 | 53.6581 | 1.178978 | 0.0013 | 0.008656 |
| Ulk2 | chr11:61775597-61855092 | 14.7395 | 17.3749 | 1.178798 | 0.00185 | 0.011363 |
| Dnalc1 | chr12:84114327-84143510 | 22.9246 | 27.0216 | 1.178716 | 0.0012 | 0.008175 |
| Vps13a | chr19:16615365-16780933 | 4.96831 | 5.85587 | 1.178644 | 0.0046 | 0.023162 |
| Skil | chr3:31095057-31122923 | 7.92687 | 9.34178 | 1.178495 | 0.00515 | 0.025295 |
| Arih1 | chr9:59388553-59486374 | 15.3981 | 18.1451 | 1.178399 | 0.00165 | 0.010418 |
| Ubqln1 | chr13:58176155-58215653 | 43.3473 | 51.0776 | 1.178334 | 0.0014 | 0.009144 |
| Srsf10 | chr4:135856091-135869899 | 23.5982 | 27.803 | 1.178183 | 0.0027 | 0.01535 |
| Smg1 | chr7:118131311-118243637 | 16.0916 | 18.9579 | 1.178124 | 0.00115 | 0.007885 |
| Abat | chr16:8513428-8621567 | 48.9244 | 57.6377 | 1.178097 | 0.0011 | 0.007621 |
| Mlec | chr5:115142980-115158176 | 23.3519 | 27.5082 | 1.177986 | 0.0016 | 0.010187 |
| Kif1b | chr4:149176320-149307698 | 61.3929 | 72.3179 | 1.177952 | 0.0053 | 0.025948 |
| 7-Sep | chr9:25252438-25308571 | 106.909 | 125.88 | 1.17745 | 0.001 | 0.007133 |
| Ctbp2 | chr7:132987010-133123483 | 14.7589 | 17.3775 | 1.177425 | 0.0083 | 0.0366 |
| Ept1 | chr5:30232617-30272430 | 7.46117 | 8.78472 | 1.177392 | 0.00505 | 0.024965 |
| Ranbp2 | chr10:58446851-58494154 | 10.3806 | 12.2189 | 1.17709 | 0.00205 | 0.012328 |
| Dpp8 | chr9:65032457-65082651 | 27.995 | 32.9487 | 1.176949 | 0.0013 | 0.008656 |
| Arhgef12 | chr9:42963841-43105718 | 17.9104 | 21.0782 | 1.176869 | 0.00095 | 0.006869 |
| Pbx1 | chr1:168119363-168432258 | 16.668 | 19.6157 | 1.176848 | 0.00215 | 0.012828 |
| Tmx4 | chr2:134594501-134644121 | 38.824 | 45.6874 | 1.176782 | 0.0013 | 0.008656 |
| Cdk7 | chr13:100669480-100730942 | 6.06002 | 7.13116 | 1.176755 | 0.01025 | 0.043155 |
| Gpr107 | chr2:31152315-31216567 | 12.1997 | 14.3508 | 1.176324 | 0.0068 | 0.031381 |
| Rit2 | chr18:30974313-31317128 | 47.8998 | 56.3419 | 1.176245 | 0.002 | 0.012075 |
| P4ha1 | chr10:59323295-59373304 | 14.3421 | 16.8664 | 1.176006 | 0.00435 | 0.022102 |
| Celsr2 | chr3:108390847-108415494 | 20.695 | 24.3327 | 1.175777 | 0.00155 | 0.009917 |
| Dusp26 | chr8:31089661-31097047 | 33.4698 | 39.3422 | 1.175454 | 0.00415 | 0.021362 |
| Gabrg2 | chr11:41910189-42000714 | 34.6381 | 40.7132 | 1.175388 | 0.0014 | 0.009144 |
| Clcn3 | chr8:60910388-60983311 | 26.6022 | 31.2676 | 1.175376 | 0.0013 | 0.008656 |
| Rnf216 | chr5:142990892-143113020 | 11.7423 | 13.8012 | 1.17534 | 0.00615 | 0.029076 |
| Atp8a1 | chr5:67618140-67847431 | 25.6553 | 30.1518 | 1.175266 | 0.0014 | 0.009144 |
| Sbk1 | chr7:126272618-126294999 | 19.8472 | 23.3252 | 1.175239 | 0.0022 | 0.013067 |
| 6-Mar | chr15:31455898-31531037 | 37.7886 | 44.4005 | 1.174971 | 0.00155 | 0.009917 |
| Akt3 | chr1:177022114-177248767 | 31.2902 | 36.7523 | 1.174563 | 0.0012 | 0.008175 |
| Eif4g1 | chr16:20672748-20692883 | 48.5554 | 57.0257 | 1.174446 | 0.00175 | 0.010881 |
| Pptc7 | chr5:122284397-122324281 | 11.2908 | 13.2554 | 1.174 | 0.00765 | 0.034391 |
| Negr1 | chr3:156561793-157316447 | 28.7199 | 33.7147 | 1.173914 | 0.00115 | 0.007885 |
| Icmt | chr4:152297213-152318625 | 13.5447 | 15.8964 | 1.173625 | 0.00645 | 0.030172 |
| Ints10 | chr8:68793953-68827644 | 23.9091 | 28.0589 | 1.173566 | 0.00475 | 0.023714 |
| Tsc22d3 | chrX:140539528-140600522 | 53.9636 | 63.3158 | 1.173306 | 0.00285 | 0.016005 |
| Ptprn2 | chr12:116485719-117337012 | 71.2836 | 83.6298 | 1.173198 | 0.00225 | 0.013268 |
| 3110035E14Rik | chr1:9548045-9631092 | 86.778 | 101.805 | 1.173166 | 0.0029 | 0.016217 |
| Pbrm1 | chr14:31019137-31121592 | 5.14862 | 6.03997 | 1.173124 | 0.0102 | 0.04303 |
| Zfp365 | chr10:67886104-67912662 | 59.0695 | 69.2784 | 1.172829 | 0.00235 | 0.013705 |
| Usp15 | chr10:123113243-123196923 | 14.8783 | 17.447 | 1.172647 | 0.0083 | 0.0366 |
| Aacs | chr5:125475872-125517403 | 17.5326 | 20.5567 | 1.172484 | 0.0058 | 0.027868 |
| Secisbp2l | chr2:125736985-125782870 | 13.2425 | 15.5237 | 1.172264 | 0.00215 | 0.012828 |
| BC030336 | chr7:120677619-120734854 | 9.377 | 10.9909 | 1.172113 | 0.00915 | 0.039575 |
| Elovl5 | chr9:77917364-77984519 | 20.9409 | 24.5441 | 1.172065 | 0.00605 | 0.02877 |
| Uhrf2 | chr19:30030512-30093724 | 16.6847 | 19.5549 | 1.172026 | 0.00575 | 0.027666 |
| Trim33 | chr3:103279292-103358768 | 6.90108 | 8.0847 | 1.171512 | 0.00475 | 0.023714 |
| Gpd1l | chr9:114899338-114933987 | 24.619 | 28.8364 | 1.171307 | 0.00185 | 0.011363 |
| Bhlhe22 | chr3:18054324-18057514 | 20.3802 | 23.8656 | 1.171019 | 0.0046 | 0.023162 |
| 2610002M06Rik | chrX:107782751-107872911 | 9.3632 | 10.963 | 1.17086 | 0.00695 | 0.031865 |
| Pcnx | chr12:81860029-82000924 | 12.6338 | 14.7893 | 1.170614 | 0.00165 | 0.010418 |
| Dpy19l3 | chr7:35685499-35754454 | 7.96718 | 9.32645 | 1.170609 | 0.00535 | 0.026156 |
| Creg2 | chr1:39618405-39651182 | 25.3627 | 29.6863 | 1.170471 | 0.0023 | 0.013502 |
| Cops2 | chr2:125830303-125859018 | 28.5321 | 33.3934 | 1.17038 | 0.00355 | 0.018973 |
| G3bp2 | chr5:92052145-92083735 | 61.0551 | 71.4453 | 1.170177 | 0.00195 | 0.011841 |
| Tgoln1,Tgoln2 | chr6:72608420-72617000 | 29.674 | 34.723 | 1.170149 | 0.0019 | 0.01161 |
| Cacna1i | chr15:80287237-80398292 | 7.67919 | 8.98455 | 1.169987 | 0.0032 | 0.01758 |
| Fbxl5 | chr5:43744617-43782149 | 27.1281 | 31.7388 | 1.16996 | 0.0038 | 0.020026 |
| Nvl | chr1:181093422-181144157 | 18.1689 | 21.2512 | 1.169647 | 0.0062 | 0.029235 |
| Ckap5 | chr2:91546321-91620665 | 19.1689 | 22.4191 | 1.169556 | 0.00245 | 0.014171 |
| Azin1 | chr15:38487429-38519266 | 26.9646 | 31.536 | 1.169533 | 0.00175 | 0.010881 |
| Gng12 | chr6:66896396-67021361 | 11.4237 | 13.3553 | 1.169087 | 0.0105 | 0.043929 |
| Ddb1 | chr19:10605624-10629821 | 56.2426 | 65.75 | 1.169043 | 0.0021 | 0.012579 |
| Csrnp3 | chr2:65845766-66031546 | 6.50615 | 7.60527 | 1.168936 | 0.0042 | 0.021546 |
| Phlpp1 | chr1:106171868-106394245 | 14.8999 | 17.4105 | 1.168498 | 0.00255 | 0.014638 |
| Fasn | chr11:120805957-120824547 | 22.0608 | 25.7743 | 1.16833 | 0.00155 | 0.009917 |
| Gnaq | chr19:16132830-16387453 | 34.7335 | 40.5736 | 1.16814 | 0.00235 | 0.013705 |
| Phf15 | chr11:51813455-51857481 | 9.70119 | 11.3304 | 1.167939 | 0.00595 | 0.028422 |
| Stk4 | chr2:164074177-164155521 | 10.3353 | 12.0689 | 1.167736 | 0.0072 | 0.032757 |
| Zdhhc17 | chr10:110941779-111010066 | 21.1344 | 24.6753 | 1.167542 | 0.0025 | 0.014398 |
| Egr1 | chr18:34861206-34864956 | 29.7548 | 34.725 | 1.167039 | 0.00355 | 0.018973 |
| 2900026A02Rik | chr5:113086322-113163313 | 16.7438 | 19.5387 | 1.166921 | 0.00285 | 0.016005 |
| Cds2 | chr2:132263256-132312041 | 61.1418 | 71.3451 | 1.166879 | 0.0064 | 0.029991 |
| Wfs1 | chr5:36966103-36988982 | 33.9322 | 39.5828 | 1.166526 | 0.0028 | 0.0158 |
| Ets2 | chr16:95702406-95721049 | 22.7465 | 26.5298 | 1.166324 | 0.00415 | 0.021362 |
| Col25a1 | chr3:130180844-130599883 | 8.98164 | 10.4744 | 1.166201 | 0.0057 | 0.027512 |
| Paqr7 | chr4:134496977-134508810 | 25.7872 | 30.0699 | 1.166079 | 0.00975 | 0.041578 |
| Nrcam | chr12:44328884-44601846 | 39.1686 | 45.6645 | 1.165845 | 0.00285 | 0.016005 |
| Rnf11 | chr4:109452856-109476505 | 78.4479 | 91.4501 | 1.165743 | 0.0022 | 0.013067 |
| A330023F24Rik | chr1:195017398-195037908 | 23.1058 | 26.9325 | 1.165616 | 0.0033 | 0.017971 |
| Kcnj9 | chr1:172321032-172329263 | 31.6106 | 36.8406 | 1.165451 | 0.0038 | 0.020026 |
| Ak3 | chr19:29020831-29048729 | 30.4572 | 35.4897 | 1.165232 | 0.0036 | 0.019163 |
| Slc1a4 | chr11:20302179-20332713 | 16.471 | 19.1901 | 1.165084 | 0.0064 | 0.029991 |
| Ylpm1 | chr12:84996320-85070515 | 13.0588 | 15.2131 | 1.164969 | 0.00335 | 0.018178 |
| Slc6a1 | chr6:114282634-114317525 | 88.0286 | 102.55 | 1.164962 | 0.00345 | 0.018588 |
| Mzt1 | chr14:99034543-99046136 | 22.4619 | 26.1641 | 1.164821 | 0.0105 | 0.043929 |
| Cdk13 | chr13:17715961-17805097 | 7.06457 | 8.22747 | 1.16461 | 0.01115 | 0.045835 |
| Dcun1d4 | chr5:73481054-73560794 | 23.5746 | 27.4542 | 1.164567 | 0.0029 | 0.016217 |
| Astn1 | chr1:158362303-158691786 | 36.1654 | 42.1114 | 1.164411 | 0.00385 | 0.02021 |
| Fbxw11 | chr11:32642554-32746814 | 34.4569 | 40.1204 | 1.164365 | 0.0029 | 0.016217 |
| Srgap2 | chr1:131285250-131527361 | 12.8032 | 14.9052 | 1.164178 | 0.00295 | 0.016462 |
| Ogfod1 | chr8:94037197-94067922 | 24.7548 | 28.816 | 1.164057 | 0.003 | 0.016689 |
| Nav2 | chr7:48959072-49610088 | 7.58936 | 8.83364 | 1.163951 | 0.00345 | 0.018588 |
| Plxna1 | chr6:89316313-89362613 | 20.518 | 23.8811 | 1.16391 | 0.00225 | 0.013268 |
| Sdc3 | chr4:130792536-130826318 | 41.5197 | 48.2978 | 1.16325 | 0.00185 | 0.011363 |
| Eif3a | chr19:60761115-60790693 | 36.275 | 42.1913 | 1.163096 | 0.0027 | 0.01535 |
| Atp2c1 | chr9:105411361-105521257 | 31.0444 | 36.1053 | 1.163021 | 0.0039 | 0.020391 |
| Stim1 | chr7:102267823-102436855 | 17.8466 | 20.754 | 1.162911 | 0.007 | 0.032067 |
| Apba1 | chr19:23758875-23949597 | 22.5073 | 26.1725 | 1.162845 | 0.00295 | 0.016462 |
| Rlim | chrX:103957166-103981284 | 7.37945 | 8.57993 | 1.162679 | 0.0106 | 0.044208 |
| Map3k10 | chr7:27656376-27674598 | 25.1257 | 29.2086 | 1.162499 | 0.0039 | 0.020391 |
| Alcam | chr16:52248995-52452997 | 20.4955 | 23.8226 | 1.162333 | 0.00355 | 0.018973 |
| Phlpp2 | chr8:109868602-109944671 | 7.03002 | 8.16979 | 1.162129 | 0.00945 | 0.040592 |
| Stx7 | chr10:24149316-24188959 | 38.8664 | 45.1664 | 1.162094 | 0.00435 | 0.022102 |
| Ndel1 | chr11:68821445-68853131 | 28.8863 | 33.565 | 1.16197 | 0.00655 | 0.030505 |
| Pitpnc1 | chr11:107207891-107470720 | 10.3149 | 11.9853 | 1.16194 | 0.00675 | 0.031231 |
| Mllt6 | chr11:97663411-97685458 | 23.2345 | 26.9879 | 1.161544 | 0.00285 | 0.016005 |
| Tm9sf2 | chr14:122107081-122159603 | 34.7187 | 40.323 | 1.16142 | 0.003 | 0.016689 |
| Dlg2 | chr7:91090785-92449246 | 44.6182 | 51.817 | 1.161342 | 0.0039 | 0.020391 |
| Reep1 | chr6:71707680-71810705 | 30.7562 | 35.7158 | 1.161255 | 0.0028 | 0.0158 |
| Tnfrsf21 | chr17:43016554-43089188 | 30.6668 | 35.6106 | 1.16121 | 0.0039 | 0.020391 |
| Smad3 | chr9:63646766-63757994 | 19.5251 | 22.6672 | 1.160926 | 0.0044 | 0.022281 |
| Cep170b | chr12:112722173-112746591 | 23.5961 | 27.373 | 1.160065 | 0.00355 | 0.018973 |
| Mxi1 | chr19:53310505-53375810 | 11.7889 | 13.6665 | 1.159268 | 0.00925 | 0.039894 |
| Add3 | chr19:53140444-53247399 | 26.1774 | 30.3461 | 1.159248 | 0.00395 | 0.020612 |
| Ctnnd1 | chr2:84600780-84650740 | 11.0856 | 12.8495 | 1.159116 | 0.0106 | 0.044208 |
| Dtx1 | chr5:120680263-120711669 | 18.5927 | 21.5509 | 1.159105 | 0.00685 | 0.031529 |
| Sik3 | chr9:46012819-46224194 | 20.4354 | 23.6852 | 1.159028 | 0.00325 | 0.017763 |
| Snx27 | chr3:94497543-94582702 | 21.0675 | 24.4117 | 1.158737 | 0.0041 | 0.021197 |
| Hmgcr | chr13:96648961-96670936 | 18.1144 | 20.988 | 1.158636 | 0.0058 | 0.027868 |
| Hk1 | chr10:62268854-62379908 | 87.706 | 101.592 | 1.158324 | 0.0068 | 0.031381 |
| Sphkap | chr1:83255780-83408200 | 28.0683 | 32.5031 | 1.158 | 0.0036 | 0.019163 |
| Vtn | chr11:78499119-78502325 | 48.7004 | 56.3797 | 1.157685 | 0.00605 | 0.02877 |
| Aatk | chr11:120007315-120047145 | 46.3168 | 53.6198 | 1.157675 | 0.00315 | 0.017359 |
| Nbea | chr3:55625197-56183701 | 24.0609 | 27.8513 | 1.157534 | 0.0041 | 0.021197 |
| Socs7 | chr11:97362550-97398542 | 17.4879 | 20.2428 | 1.157532 | 0.00405 | 0.021031 |
| Srcin1 | chr11:97509339-97575126 | 38.4881 | 44.5496 | 1.15749 | 0.00435 | 0.022102 |
| 4932438A13Rik | chr3:36863105-37053033 | 7.39199 | 8.55613 | 1.157487 | 0.00415 | 0.021362 |
| Pdpk1 | chr17:24073679-24141594 | 14.4557 | 16.7316 | 1.15744 | 0.0044 | 0.022281 |
| Bsn | chr9:108096021-108190383 | 24.7971 | 28.6924 | 1.157087 | 0.00615 | 0.029076 |
| Cdk5r1 | chr11:80477045-80481179 | 41.9939 | 48.5746 | 1.156706 | 0.0038 | 0.020026 |
| Clic4 | chr4:135213969-135272760 | 18.8248 | 21.7746 | 1.156698 | 0.0067 | 0.031027 |
| Osbpl6 | chr2:76406537-76600647 | 14.5413 | 16.8197 | 1.156685 | 0.00345 | 0.018588 |
| 9330159F19Rik | chr10:29211642-29230779 | 20.4324 | 23.6292 | 1.156457 | 0.004 | 0.020812 |
| Nceh1 | chr3:27183003-27244911 | 23.6459 | 27.3412 | 1.156277 | 0.0042 | 0.021546 |
| Kpna6 | chr4:129643978-129672767 | 17.3607 | 20.073 | 1.156232 | 0.00495 | 0.02454 |
| Pnma2 | chr14:66911207-66920061 | 14.0888 | 16.2862 | 1.155968 | 0.0096 | 0.04107 |
| Nrep | chr18:33437018-33464029 | 42.2679 | 48.8596 | 1.15595 | 0.0062 | 0.029235 |
| Nr1d2 | chr14:18204055-18239106 | 20.742 | 23.9636 | 1.155318 | 0.0051 | 0.025119 |
| Mll1 | chr9:44803354-44881274 | 13.3521 | 15.4136 | 1.154395 | 0.0035 | 0.018791 |
| Pafah1b1 | chr11:74673948-74724384 | 78.6561 | 90.7886 | 1.154247 | 0.0097 | 0.041448 |
| Xpo7 | chr14:70654245-70766628 | 5.63044 | 6.49493 | 1.153539 | 0.00635 | 0.029796 |
| Herpud1 | chr8:94386499-94395358 | 31.1904 | 35.976 | 1.153432 | 0.0124 | 0.049986 |
| Tmed10 | chr12:85340613-85374717 | 37.0996 | 42.7899 | 1.153379 | 0.00455 | 0.022964 |
| Mll2 | chr15:98831668-98871205 | 8.90059 | 10.2636 | 1.153137 | 0.00405 | 0.021031 |
| Ppm1h | chr10:122678761-122945793 | 11.1816 | 12.8934 | 1.153091 | 0.0098 | 0.041724 |
| Ccp110 | chr7:118712610-118737018 | 12.6547 | 14.587 | 1.152694 | 0.0096 | 0.04107 |
| Chd5 | chr4:152338650-152390194 | 32.5505 | 37.5198 | 1.152664 | 0.0065 | 0.030326 |
| Hnrnph2 | chrX:134601285-134607054 | 53.0978 | 61.1949 | 1.152494 | 0.0051 | 0.025119 |
| Eps15 | chr4:109280274-109387816 | 45.4626 | 52.3776 | 1.152103 | 0.00595 | 0.028422 |
| Rgs4 | chr1:169741476-169747642 | 55.2585 | 63.609 | 1.151117 | 0.0061 | 0.028943 |
| 6820431F20Rik | chr8:20268285-20297432 | 48.9776 | 56.3688 | 1.15091 | 0.0058 | 0.027868 |
| Rasa3 | chr8:13567217-13677587 | 18.7743 | 21.5947 | 1.150227 | 0.0087 | 0.038095 |
| A030009H04Rik | chr11:69340768-69342647 | 51.4767 | 59.2079 | 1.150188 | 0.0075 | 0.033932 |
| Tm9sf3 | chr19:41210841-41264004 | 21 | 24.151 | 1.150048 | 0.0049 | 0.02436 |
| Kcnc1 | chr7:46396467-46438704 | 28.3464 | 32.5991 | 1.150026 | 0.0092 | 0.039743 |
| Atg2b | chr12:105613539-105685241 | 6.44549 | 7.41218 | 1.149979 | 0.0115 | 0.047055 |
| Mapre2 | chr18:23752332-23893861 | 58.6275 | 67.4134 | 1.14986 | 0.0065 | 0.030326 |
| Gdap1 | chr1:17145372-17164270 | 28.776 | 33.0711 | 1.14926 | 0.00615 | 0.029076 |
| Dlg5 | chr14:24133952-24245920 | 9.15259 | 10.5175 | 1.149128 | 0.0107 | 0.044502 |
| Lingo1 | chr9:56618474-56685253 | 55.5325 | 63.8103 | 1.149062 | 0.0061 | 0.028943 |
| Brd4 | chr17:32196273-32284123 | 14.4979 | 16.6581 | 1.149001 | 0.0114 | 0.046754 |
| Dld | chr12:31331561-31351437 | 51.6527 | 59.3438 | 1.1489 | 0.0051 | 0.025119 |
| Foxk1 | chr5:142401496-142462015 | 10.5898 | 12.1635 | 1.148605 | 0.00905 | 0.039271 |
| Vps33a | chr5:123528759-123573015 | 23.497 | 26.9803 | 1.148244 | 0.00805 | 0.035765 |
| Mras | chr9:99385419-99436712 | 50.5943 | 58.075 | 1.147857 | 0.0062 | 0.029235 |
| Nrxn2 | chr19:6418737-6533217 | 27.9084 | 32.0324 | 1.147769 | 0.006 | 0.028609 |
| Slc41a1 | chr1:131828011-131848864 | 17.557 | 20.1505 | 1.147719 | 0.008 | 0.035603 |
| Ptprd | chr4:75941236-78211895 | 21.9059 | 25.1398 | 1.147627 | 0.0068 | 0.031381 |
| Dock3 | chr9:106892824-107231909 | 18.3508 | 21.0445 | 1.146789 | 0.0071 | 0.032371 |
| Adarb1 | chr10:77290726-77418273 | 24.6574 | 28.2641 | 1.146273 | 0.0069 | 0.031704 |
| Tyro3 | chr2:119799513-119818103 | 35.3397 | 40.5028 | 1.146099 | 0.0078 | 0.034859 |
| Pcmtd2 | chr2:181837902-181857450 | 20.0513 | 22.9697 | 1.145547 | 0.01205 | 0.048798 |
| Atp11a | chr8:12757015-12868728 | 11.4755 | 13.1433 | 1.145336 | 0.01025 | 0.043155 |
| Dst | chr1:34011824-34308652 | 19.3663 | 22.1772 | 1.145144 | 0.01055 | 0.044068 |
| Gabra4 | chr5:71569733-71658308 | 21.4142 | 24.5219 | 1.145123 | 0.00995 | 0.042244 |
| Sc4mol | chr8:64718144-64733578 | 47.6237 | 54.5289 | 1.144995 | 0.0117 | 0.047671 |
| Dag1 | chr9:108204860-108263958 | 16.489 | 18.8789 | 1.144939 | 0.0099 | 0.042065 |
| Bri3bp | chr5:125441567-125460885 | 16.7597 | 19.1837 | 1.144633 | 0.0088 | 0.038422 |
| Dmxl2 | chr9:54365157-54501626 | 20.073 | 22.975 | 1.144572 | 0.00765 | 0.034391 |
| Arhgef3 | chr14:27238038-27403904 | 27.6513 | 31.6433 | 1.144369 | 0.0099 | 0.042065 |
| Hcfc1 | chrX:73942794-73966315 | 14.5594 | 16.6566 | 1.144044 | 0.0084 | 0.036995 |
| Vps35 | chr8:85260391-85299497 | 43.3838 | 49.6239 | 1.143835 | 0.0081 | 0.035882 |
| Zbtb4 | chr11:69765911-69784026 | 15.1865 | 17.3638 | 1.143371 | 0.00845 | 0.0372 |
| Tln2 | chr9:67217084-67559703 | 10.3994 | 11.8881 | 1.143152 | 0.00805 | 0.035765 |
| Arpp21 | chr9:112065090-112348925 | 67.1155 | 76.673 | 1.142404 | 0.00925 | 0.039894 |
| Sipa1l1 | chr12:82170015-82451784 | 37.2241 | 42.5221 | 1.142327 | 0.00945 | 0.040592 |
| Snx10 | chr6:51523902-51590670 | 48.1653 | 54.9844 | 1.141577 | 0.00875 | 0.038251 |
| Celf5 | chr10:81459227-81482709 | 73.6641 | 84.0296 | 1.140713 | 0.0115 | 0.047055 |
| Pip4k2b | chr11:97715156-97744704 | 27.0162 | 30.8167 | 1.140675 | 0.0077 | 0.034557 |
| Mapk8ip2 | chr15:89453910-89462447 | 62.0785 | 70.7667 | 1.139955 | 0.0085 | 0.037389 |
| Tomm70a | chr16:57121713-57154530 | 40.9314 | 46.6457 | 1.139607 | 0.01045 | 0.043858 |
| Cdk14 | chr5:4803384-5380251 | 44.6468 | 50.8739 | 1.139475 | 0.00875 | 0.038251 |
| Birc6 | chr17:74528294-74703356 | 8.23019 | 9.37686 | 1.139325 | 0.00985 | 0.041903 |
| Rbfox2 | chr15:77078989-77307053 | 43.0257 | 49.0195 | 1.139307 | 0.0108 | 0.044795 |
| Ralgapa1 | chr12:55602898-55821167 | 17.476 | 19.9082 | 1.139174 | 0.01 | 0.042388 |
| Pygb | chr2:150786795-150831748 | 69.3556 | 78.9654 | 1.138558 | 0.00945 | 0.040592 |
| Rbm33 | chr5:28317188-28419242 | 14.1609 | 16.1193 | 1.138296 | 0.0102 | 0.04303 |
| Ddx6 | chr9:44604891-44640731 | 22.6677 | 25.8016 | 1.138254 | 0.0096 | 0.04107 |
| Gad1 | chr2:70489939-70602012 | 91.4976 | 104.127 | 1.13803 | 0.01145 | 0.046923 |
| Crk | chr11:75679309-75706092 | 35.2123 | 40.0419 | 1.137157 | 0.0096 | 0.04107 |
| Tmem127 | chr2:127247974-127260764 | 27.7687 | 31.5746 | 1.137057 | 0.0105 | 0.043929 |
| Nacc1 | chr8:84670478-84687862 | 29.5076 | 33.5505 | 1.137012 | 0.01 | 0.042388 |
| Cstf2 | chrX:134059348-134086821 | 25.6619 | 29.173 | 1.136822 | 0.01175 | 0.047838 |
| Cdc42bpb | chr12:111292971-111377718 | 21.273 | 24.1693 | 1.136149 | 0.01155 | 0.047187 |
| Abhd8 | chr8:71456699-71463657 | 81.3047 | 92.3094 | 1.135351 | 0.01185 | 0.048135 |
| **Downregulated genes** | | | | | | |
| Gdf1 | chr8:70315774-70353273 | 4.05021 | 0.572271 | 0.141294 | 0.004 | 0.020812 |
| Cstad | chr2:30595043-30608945 | 7.44348 | 2.24213 | 0.301221 | 0.0001 | 0.001096 |
| G530011O06Rik | chrX:169685246-169990797 | 42.5718 | 13.2166 | 0.310454 | 5.00E-05 | 0.000607 |
| Cldn22 | chr8:47824481-47825475 | 4.43019 | 1.93658 | 0.437132 | 0.00235 | 0.013705 |
| Pla2g4e | chr2:120166411-120245335 | 3.72542 | 1.76933 | 0.474934 | 5.00E-05 | 0.000607 |
| Tipin | chr9:64281606-64304792 | 16.1072 | 8.6664 | 0.538045 | 5.00E-05 | 0.000607 |
| Car7 | chr8:104446718-104624396 | 26.1246 | 14.1821 | 0.542864 | 5.00E-05 | 0.000607 |
| Etv4 | chr11:101769741-101785310 | 7.10894 | 3.97351 | 0.558945 | 5.00E-05 | 0.000607 |
| Lefty1 | chr1:180935038-180938401 | 8.53336 | 4.80539 | 0.56313 | 5.00E-05 | 0.000607 |
| 1700047M11Rik | chr1:182300833-182303289 | 12.3183 | 6.97885 | 0.566543 | 0.00135 | 0.008911 |
| Ecscr | chr18:35713088-35721491 | 4.53274 | 2.57138 | 0.56729 | 0.0077 | 0.034557 |
| Xpa | chr4:46175221-46196311 | 11.7139 | 6.66326 | 0.568834 | 0.00035 | 0.003088 |
| Lor | chr3:92080270-92083142 | 3.87569 | 2.22548 | 0.574215 | 0.0015 | 0.009667 |
| Krt9 | chr11:100186780-100193246 | 7.40288 | 4.26984 | 0.576781 | 5.00E-05 | 0.000607 |
| 1110007C09Rik | chr13:49202950-49216026 | 8.53023 | 4.92008 | 0.576782 | 0.0035 | 0.018791 |
| Ccdc37 | chr6:90403735-90428480 | 10.703 | 6.20335 | 0.57959 | 5.00E-05 | 0.000607 |
| Aldob | chr4:49535994-49549483 | 6.24963 | 3.64961 | 0.583972 | 0.0002 | 0.001971 |
| Tnni1 | chr1:135799420-135810989 | 4.5379 | 2.66314 | 0.586866 | 0.01205 | 0.048798 |
| Setd4 | chr16:93583460-93603815 | 7.15216 | 4.22098 | 0.590169 | 5.00E-05 | 0.000607 |
| Il20rb | chr9:100457718-100486473 | 3.96708 | 2.34685 | 0.591581 | 0.00035 | 0.003088 |
| Gm5918 | chr9:72979724-72985376 | 11.8352 | 7.02515 | 0.593581 | 0.00235 | 0.013705 |
| Mapk12 | chr15:89130583-89140703 | 5.51429 | 3.27634 | 0.594154 | 0.00045 | 0.003815 |
| Zfp580 | chr7:5051531-5053723 | 16.4901 | 9.89592 | 0.600113 | 5.00E-05 | 0.000607 |
| Phf7 | chr14:31237695-31251218 | 3.84831 | 2.31499 | 0.60156 | 0.0022 | 0.013067 |
| Hyls1 | chr9:35559465-35570069 | 7.22729 | 4.35732 | 0.602898 | 0.00245 | 0.014171 |
| Snhg8 | chr3:123507551-123508336 | 18.0815 | 10.9184 | 0.603844 | 0.00025 | 0.002352 |
| Itga7 | chr10:128933812-128960988 | 15.4756 | 9.36659 | 0.605249 | 5.00E-05 | 0.000607 |
| Wtip | chr7:34109549-34133268 | 5.72878 | 3.47293 | 0.606225 | 0.0003 | 0.002726 |
| Isoc2b | chr7:4844959-4866179 | 8.7621 | 5.31251 | 0.606306 | 0.0014 | 0.009144 |
| Spink8 | chr9:109816626-109826627 | 47.0781 | 28.6685 | 0.608956 | 5.00E-05 | 0.000607 |
| Dctpp1 | chr7:127256958-127260667 | 27.3236 | 16.6475 | 0.609272 | 5.00E-05 | 0.000607 |
| Bcas1 | chr2:170346990-170427845 | 92.5367 | 56.8482 | 0.614331 | 5.00E-05 | 0.000607 |
| Mbp | chr18:82475122-82585637 | 2357.55 | 1449.8 | 0.61496 | 0.011 | 0.045394 |
| A430105I19Rik | chr2:118754144-118762661 | 5.45799 | 3.36243 | 0.616056 | 5.00E-05 | 0.000607 |
| Ntf3 | chr6:126101411-126166744 | 8.89249 | 5.48298 | 0.616585 | 0.00045 | 0.003815 |
| Snrnp25 | chr11:32205414-32208995 | 28.4988 | 17.6389 | 0.618935 | 5.00E-05 | 0.000607 |
| Sap30 | chr8:57482701-57487860 | 7.3093 | 4.52818 | 0.619509 | 0.00325 | 0.017763 |
| Acta1 | chr8:123891757-123894775 | 6.24444 | 3.87753 | 0.620957 | 0.0013 | 0.008656 |
| Siva1 | chr12:112644827-112649152 | 13.0472 | 8.14816 | 0.624514 | 0.00185 | 0.011363 |
| Snhg6 | chr1:9942024-9944118 | 51.6596 | 32.3896 | 0.626981 | 0.00015 | 0.001558 |
| Rab34 | chr11:78188426-78192193 | 23.9542 | 15.1038 | 0.630528 | 5.00E-05 | 0.000607 |
| Ankrd23 | chr1:36530533-36535729 | 4.49668 | 2.83689 | 0.630885 | 0.0027 | 0.01535 |
| Acot2 | chr12:83987860-83993875 | 5.25196 | 3.35449 | 0.638712 | 0.00105 | 0.007395 |
| Trappc6a | chr7:19508728-19516145 | 12.0649 | 7.7096 | 0.639011 | 0.0043 | 0.021953 |
| Gm5148 | chr3:37714189-37724360 | 10.9539 | 7.01596 | 0.640499 | 0.00035 | 0.003088 |
| Oxld1 | chr11:120456603-120458063 | 10.2344 | 6.57923 | 0.642854 | 0.0067 | 0.031027 |
| Polr3g | chr13:81673836-81711013 | 3.91066 | 2.51449 | 0.642984 | 0.0006 | 0.004799 |
| Gm15706 | chr6:145250551-145251856 | 13.7214 | 8.82921 | 0.643463 | 0.001 | 0.007133 |
| Trmt13 | chr3:116562972-116630984 | 4.13097 | 2.65856 | 0.643568 | 0.002 | 0.012075 |
| Gng11 | chr6:4003986-4008446 | 15.7267 | 10.1237 | 0.643727 | 0.00085 | 0.0063 |
| Hapln2 | chr3:88021749-88027511 | 10.6267 | 6.87006 | 0.64649 | 5.00E-05 | 0.000607 |
| 1810043H04Rik | chr11:120098933-120100424 | 24.4546 | 15.8476 | 0.648042 | 0.00365 | 0.01938 |
| Abracl | chr10:18011259-18023252 | 24.968 | 16.1952 | 0.648638 | 5.00E-05 | 0.000607 |
| 2610524H06Rik | chr5:114821936-114823468 | 21.3443 | 13.8481 | 0.648796 | 0.00015 | 0.001558 |
| Zfp46 | chr4:136286068-136293942 | 15.2418 | 9.89723 | 0.649348 | 5.00E-05 | 0.000607 |
| 2810029C07Rik | chr12:111572316-111574402 | 10.7411 | 6.99155 | 0.650916 | 5.00E-05 | 0.000607 |
| Mettl25 | chr10:105763184-105841380 | 4.73871 | 3.08596 | 0.651224 | 0.00285 | 0.016005 |
| Cep78 | chr19:15955772-15984989 | 8.38438 | 5.46501 | 0.651808 | 5.00E-05 | 0.000607 |
| Rhebl1 | chr15:98877759-98881414 | 6.6128 | 4.31263 | 0.652164 | 0.00755 | 0.034086 |
| Lyrm1 | chr7:119896291-119916750 | 7.39298 | 4.83213 | 0.653611 | 0.0039 | 0.020391 |
| Mrps28 | chr3:8802145-8923857 | 23.0705 | 15.1078 | 0.654854 | 0.0008 | 0.006001 |
| Gpatch3 | chr4:133574744-133584242 | 4.64198 | 3.04317 | 0.655576 | 0.0035 | 0.018791 |
| Lsm7 | chr10:80852824-80855209 | 126.104 | 82.8258 | 0.656805 | 5.00E-05 | 0.000607 |
| Col11a1 | chr3:114030539-114220326 | 6.01976 | 3.96092 | 0.657986 | 5.00E-05 | 0.000607 |
| Fth1 | chr19:9980599-9985111 | 3504.29 | 2310.38 | 0.6593 | 0.0001 | 0.001096 |
| Zcwpw1 | chr5:137787801-137836278 | 4.34913 | 2.87103 | 0.660139 | 0.00955 | 0.040972 |
| 2010204K13Rik | chrX:7411816-7422988 | 60.906 | 40.227 | 0.660477 | 5.00E-05 | 0.000607 |
| Trim68 | chr7:102677579-102687326 | 4.24073 | 2.80102 | 0.660504 | 0.002 | 0.012075 |
| Snhg12 | chr4:132308677-132311024 | 60.5181 | 39.9967 | 0.660905 | 5.00E-05 | 0.000607 |
| Pcbd1 | chr10:61089342-61094329 | 13.0221 | 8.62636 | 0.66244 | 0.00395 | 0.020612 |
| Sec61g | chr11:16501637-16508484 | 84.3349 | 56.0133 | 0.664177 | 5.00E-05 | 0.000607 |
| E130307A14Rik | chr10:39612933-39732007 | 22.2584 | 14.7858 | 0.66428 | 5.00E-05 | 0.000607 |
| Arrdc2 | chr8:70835137-70839720 | 11.9853 | 7.97335 | 0.665261 | 5.00E-05 | 0.000607 |
| Hint2 | chr4:43654226-43656445 | 53.1227 | 35.4823 | 0.667931 | 5.00E-05 | 0.000607 |
| Nipsnap1 | chr11:4874002-4894200 | 78.4859 | 52.4257 | 0.667963 | 5.00E-05 | 0.000607 |
| Thap3 | chr4:151982637-151988986 | 19.4256 | 12.9955 | 0.668988 | 5.00E-05 | 0.000607 |
| Srpk3 | chrX:73774421-73778924 | 4.55592 | 3.04855 | 0.66914 | 0.00475 | 0.023714 |
| Rpp21 | chr17:36255672-36257846 | 44.8686 | 30.0341 | 0.669379 | 0.00025 | 0.002352 |
| Slc26a10 | chr10:127172425-127180645 | 10.7623 | 7.21575 | 0.670465 | 0.0001 | 0.001096 |
| 1500012F01Rik | chr2:167035796-167065862 | 70.6416 | 47.4003 | 0.670997 | 0.0005 | 0.004159 |
| H2-Ke6 | chr17:34026032-34028055 | 44.6467 | 29.96 | 0.671046 | 5.00E-05 | 0.000607 |
| Gm13375 | chr2:20968873-20970348 | 10.723 | 7.21204 | 0.672577 | 0.00095 | 0.006869 |
| Nsun7 | chr5:66260124-66298016 | 10.2865 | 6.92912 | 0.673613 | 5.00E-05 | 0.000607 |
| Asphd1 | chr7:126946007-126949581 | 39.5182 | 26.7471 | 0.67683 | 0.00095 | 0.006869 |
| Smim8 | chr4:34768671-34778337 | 16.9645 | 11.5203 | 0.679083 | 0.0013 | 0.008656 |
| BC030500 | chr8:58911754-58914298 | 9.58429 | 6.53274 | 0.681609 | 0.00085 | 0.0063 |
| Gfm2 | chr13:97137936-97198357 | 18.2735 | 12.4989 | 0.68399 | 0.0001 | 0.001096 |
| Plekhb1 | chr7:100643895-100662394 | 262.008 | 179.346 | 0.684506 | 5.00E-05 | 0.000607 |
| Nmrk1 | chr19:18632015-18652184 | 8.43131 | 5.77421 | 0.684853 | 0.00235 | 0.013705 |
| Tnxb | chr17:34670534-34719815 | 6.05046 | 4.14529 | 0.68512 | 5.00E-05 | 0.000607 |
| Doc2g | chr19:4003384-4007005 | 5.91857 | 4.05771 | 0.68559 | 0.0123 | 0.049696 |
| Pglyrp1 | chr7:18884689-18890438 | 18.4909 | 12.6857 | 0.686051 | 0.005 | 0.024764 |
| 2010107E04Rik | chr12:111961375-111966977 | 541.329 | 371.5 | 0.686274 | 5.00E-05 | 0.000607 |
| Hvcn1 | chr5:122209736-122264460 | 6.65096 | 4.56447 | 0.686287 | 0.01145 | 0.046923 |
| Oaf | chr9:43221277-43239816 | 7.21775 | 4.9594 | 0.687112 | 0.0007 | 0.005435 |
| A930017M01Rik | chr15:44881393-44884743 | 6.33695 | 4.36074 | 0.688145 | 5.00E-05 | 0.000607 |
| 1700113A16Rik | chr3:88171559-88177785 | 47.6332 | 32.8334 | 0.689297 | 5.00E-05 | 0.000607 |
| Rfc5 | chr5:117379144-117389023 | 8.45551 | 5.82871 | 0.689339 | 0.0013 | 0.008656 |
| Clk1 | chr1:58411987-58424088 | 99.3391 | 68.5287 | 0.689846 | 5.00E-05 | 0.000607 |
| Strada | chr11:106162973-106193603 | 23.5437 | 16.2562 | 0.690469 | 5.00E-05 | 0.000607 |
| Tmem194b | chr1:52630704-52651919 | 4.14136 | 2.86046 | 0.690705 | 0.00125 | 0.008413 |
| Polm | chr11:5827859-5838016 | 5.27429 | 3.64645 | 0.691363 | 0.0009 | 0.006615 |
| Fam173a | chr17:25786579-25792284 | 89.362 | 61.8119 | 0.691702 | 5.00E-05 | 0.000607 |
| Bcl10 | chr3:145924377-145934283 | 7.63818 | 5.28379 | 0.69176 | 0.00245 | 0.014171 |
| Ndufa5 | chr6:24518665-24527687 | 309.38 | 214.546 | 0.693471 | 5.00E-05 | 0.000607 |
| Cercam | chr2:29869493-29882840 | 6.05262 | 4.20423 | 0.694613 | 0.0007 | 0.005435 |
| 3000002C10Rik | chr9:109830153-109831431 | 7.88351 | 5.47629 | 0.694651 | 0.008 | 0.035603 |
| Ripk2 | chr4:16123374-16163498 | 5.1096 | 3.55136 | 0.695037 | 0.00755 | 0.034086 |
| Hddc2 | chr10:31313404-31328086 | 23.9559 | 16.6857 | 0.696517 | 0.00215 | 0.012828 |
| Mrto4 | chr4:139347444-139352298 | 44.134 | 30.7629 | 0.697034 | 5.00E-05 | 0.000607 |
| Npy | chr6:49822728-49829505 | 177.732 | 123.937 | 0.697325 | 5.00E-05 | 0.000607 |
| Tmsb4x | chrX:167207093-167209218 | 2936.28 | 2054.92 | 0.699838 | 5.00E-05 | 0.000607 |
| Rgs11 | chr17:26202961-26211324 | 35.5846 | 24.9038 | 0.699848 | 5.00E-05 | 0.000607 |
| Chchd7 | chr4:3938887-3943525 | 30.3259 | 21.2521 | 0.70079 | 0.009 | 0.039086 |
| Bok | chr1:93685693-93695762 | 59.4639 | 41.6745 | 0.700837 | 5.00E-05 | 0.000607 |
| Zdhhc4 | chr5:143316488-143329238 | 24.7805 | 17.3955 | 0.701983 | 5.00E-05 | 0.000607 |
| Bcl7c | chr7:127704977-127708766 | 11.1853 | 7.85255 | 0.702042 | 0.00975 | 0.041578 |
| Rtel1 | chr2:181319723-181356616 | 9.75297 | 6.85291 | 0.702649 | 5.00E-05 | 0.000607 |
| Zfp386 | chr12:116047723-116060594 | 17.6826 | 12.4373 | 0.703364 | 5.00E-05 | 0.000607 |
| Ndufc1 | chr3:51405478-51408955 | 229.853 | 161.731 | 0.703628 | 5.00E-05 | 0.000607 |
| Naglu | chr11:101070093-101077671 | 5.25295 | 3.69987 | 0.704341 | 0.0034 | 0.018393 |
| Mobp | chr9:120149741-120176091 | 155.337 | 109.521 | 0.705054 | 5.00E-05 | 0.000607 |
| S1pr5 | chr9:21242916-21248443 | 6.78005 | 4.78088 | 0.705139 | 0.00145 | 0.009402 |
| Tsr3 | chr17:25240169-25256364 | 29.2742 | 20.6485 | 0.705348 | 0.00075 | 0.005715 |
| Mri1 | chr8:84250575-84257324 | 8.65116 | 6.10415 | 0.705587 | 0.00055 | 0.004491 |
| Zfp963 | chr8:69741638-69749962 | 6.26289 | 4.43067 | 0.707448 | 0.0031 | 0.017146 |
| Fam163b | chr2:27110378-27142477 | 96.8014 | 68.5194 | 0.707835 | 5.00E-05 | 0.000607 |
| Qdpr | chr5:45434031-45450229 | 205.979 | 145.976 | 0.708694 | 5.00E-05 | 0.000607 |
| Smarcd2 | chr11:106263170-106272972 | 19.1074 | 13.5496 | 0.709128 | 5.00E-05 | 0.000607 |
| Sh3bgrl3 | chr4:134127405-134128753 | 298.222 | 211.63 | 0.709639 | 5.00E-05 | 0.000607 |
| Cck | chr9:121489823-121495689 | 528.502 | 375.096 | 0.709734 | 5.00E-05 | 0.000607 |
| Glrx3 | chr7:137437647-137468594 | 50.6055 | 35.9508 | 0.710413 | 5.00E-05 | 0.000607 |
| Rpl23 | chr11:97777525-97782439 | 358.849 | 255.01 | 0.710633 | 5.00E-05 | 0.000607 |
| Dand5 | chr8:84815404-84832265 | 14.6762 | 10.4314 | 0.71077 | 0.0012 | 0.008175 |
| Bbs5 | chr2:69647254-69667569 | 16.626 | 11.8196 | 0.710911 | 0.00035 | 0.003088 |
| Upf3a | chr8:13785614-13798537 | 36.1388 | 25.7004 | 0.711158 | 5.00E-05 | 0.000607 |
| Anxa11 | chr14:25842154-25886804 | 37.3715 | 26.5987 | 0.711738 | 5.00E-05 | 0.000607 |
| Mlip | chr9:77102083-77347870 | 14.8419 | 10.576 | 0.712577 | 0.0015 | 0.009667 |
| Rpl35 | chr2:38998308-39005131 | 812.424 | 579.786 | 0.71365 | 5.00E-05 | 0.000607 |
| 2410006H16Rik | chr11:62602876-62604806 | 58.7635 | 41.94 | 0.713708 | 0.0008 | 0.006001 |
| Cdk9 | chr2:32705781-32712784 | 52.6545 | 37.6173 | 0.714418 | 5.00E-05 | 0.000607 |
| Fhl2 | chr1:43123073-43163961 | 26.2552 | 18.7631 | 0.714643 | 5.00E-05 | 0.000607 |
| 2610002J02Rik | chr4:155249965-155256687 | 27.8704 | 19.9472 | 0.715713 | 0.0002 | 0.001971 |
| Timm10 | chr2:84827020-84830213 | 66.3606 | 47.5108 | 0.715949 | 5.00E-05 | 0.000607 |
| Gm12191 | chr15:34440505-34443276 | 706.348 | 505.809 | 0.71609 | 5.00E-05 | 0.000607 |
| Mrpl54 | chr10:81264721-81266926 | 49.2179 | 35.2482 | 0.716166 | 0.00025 | 0.002352 |
| Rps21 | chr2:180257378-180258444 | 1002.32 | 717.982 | 0.71632 | 5.00E-05 | 0.000607 |
| Lrrc17 | chr5:21483846-21645605 | 522.933 | 374.86 | 0.716841 | 5.00E-05 | 0.000607 |
| Car15 | chr16:17835275-17838186 | 9.85899 | 7.07491 | 0.71761 | 0.01235 | 0.049841 |
| Rps17 | chr7:81342732-81345234 | 805.355 | 578.015 | 0.717715 | 5.00E-05 | 0.000607 |
| Rpl32 | chr6:115774556-115808743 | 700.442 | 502.932 | 0.718021 | 5.00E-05 | 0.000607 |
| Ndufa1 | chrX:37187588-37191238 | 248.434 | 178.421 | 0.718183 | 5.00E-05 | 0.000607 |
| Ddx39 | chr8:83715176-83741311 | 14.6239 | 10.5169 | 0.719158 | 0.0071 | 0.032371 |
| Rpl38 | chr11:114344525-114757886 | 1366.53 | 983.044 | 0.719372 | 5.00E-05 | 0.000607 |
| Prr7 | chr13:55464266-55473155 | 9.39112 | 6.76312 | 0.720161 | 0.0081 | 0.035882 |
| Mus81 | chr19:5482839-5488336 | 14.8641 | 10.7049 | 0.720185 | 5.00E-05 | 0.000607 |
| Psmb5 | chr14:54614119-54617995 | 181.185 | 130.5 | 0.720258 | 5.00E-05 | 0.000607 |
| Ndufa6 | chr15:82350138-82354291 | 249.012 | 179.569 | 0.721126 | 5.00E-05 | 0.000607 |
| Prss36 | chr7:127932637-127946725 | 3.93116 | 2.83567 | 0.721332 | 0.0086 | 0.03775 |
| Siah2 | chr3:58674948-58692388 | 8.27846 | 5.97314 | 0.721528 | 0.00025 | 0.002352 |
| Wdr92 | chr11:17211892-17235200 | 6.329 | 4.56768 | 0.721706 | 0.00055 | 0.004491 |
| Mrpl33 | chr5:31613950-31622644 | 100.097 | 72.2686 | 0.721986 | 5.00E-05 | 0.000607 |
| Bmp1 | chr14:70474554-70520260 | 52.2499 | 37.7304 | 0.722114 | 5.00E-05 | 0.000607 |
| B9d2 | chr7:25681157-25686558 | 14.3444 | 10.3653 | 0.722603 | 0.0053 | 0.025948 |
| Rps27a | chr11:29545841-29578352 | 645.933 | 466.985 | 0.722962 | 5.00E-05 | 0.000607 |
| Pet100 | chr8:3621550-3625545 | 24.5665 | 17.8073 | 0.724861 | 0.0003 | 0.002726 |
| Lmo1 | chr7:109138571-109170308 | 35.3077 | 25.6187 | 0.725584 | 5.00E-05 | 0.000607 |
| Snrpe | chr1:133603870-133610280 | 93.2346 | 67.6633 | 0.725732 | 5.00E-05 | 0.000607 |
| Mif4gd | chr11:115607918-115612969 | 12.9903 | 9.42777 | 0.725755 | 0.00205 | 0.012328 |
| Setd6 | chr8:95715912-95719004 | 12.1568 | 8.82414 | 0.72586 | 0.00095 | 0.006869 |
| Fam162a | chr16:36043843-36071515 | 95.75 | 69.5271 | 0.726132 | 5.00E-05 | 0.000607 |
| Mov10 | chr3:104794833-104818563 | 10.7658 | 7.81853 | 0.726238 | 5.00E-05 | 0.000607 |
| Sdhaf1 | chr7:30321408-30322375 | 21.9251 | 15.9388 | 0.726966 | 0.0014 | 0.009144 |
| Zfp112 | chr7:24112319-24127952 | 5.61009 | 4.08365 | 0.727912 | 0.00125 | 0.008413 |
| Naa38 | chr6:18848634-18854052 | 44.7833 | 32.6133 | 0.728247 | 0.00015 | 0.001558 |
| Rpl37a | chr1:72711259-72713813 | 596.83 | 434.694 | 0.728338 | 5.00E-05 | 0.000607 |
| Ccdc134 | chr15:82127921-82142202 | 9.52034 | 6.93511 | 0.728452 | 0.0016 | 0.010187 |
| Ap1g2 | chr14:55094783-55106593 | 9.50612 | 6.9256 | 0.728541 | 0.0107 | 0.044502 |
| Ppdpf | chr2:181187342-181188504 | 75.0847 | 54.716 | 0.728724 | 5.00E-05 | 0.000607 |
| Hpca | chr4:129105529-129121740 | 1375.22 | 1002.32 | 0.728843 | 0.0002 | 0.001971 |
| Lbr | chr1:181815314-181842401 | 5.99151 | 4.36755 | 0.728956 | 0.0011 | 0.007621 |
| Miip | chr4:147860777-147868719 | 19.4672 | 14.1935 | 0.729098 | 5.00E-05 | 0.000607 |
| Atp5e | chr2:174461074-174464101 | 481.548 | 351.471 | 0.729877 | 5.00E-05 | 0.000607 |
| H2-DMa | chr17:34122831-34139101 | 14.2556 | 10.4106 | 0.730281 | 0.0012 | 0.008175 |
| Med12 | chrX:101274090-101298934 | 14.0655 | 10.2732 | 0.730383 | 5.00E-05 | 0.000607 |
| Pfn1 | chr11:70651846-70654650 | 224.746 | 164.197 | 0.730589 | 5.00E-05 | 0.000607 |
| Idua | chr5:108669105-108684558 | 10.2117 | 7.46081 | 0.730614 | 0.0001 | 0.001096 |
| Tsta3 | chr15:75924682-75929730 | 23.7881 | 17.3841 | 0.73079 | 0.0001 | 0.001096 |
| Anks6 | chr4:47015688-47057306 | 7.3197 | 5.34969 | 0.730862 | 0.00035 | 0.003088 |
| S100a13 | chr3:90514434-90524581 | 44.8643 | 32.8002 | 0.731098 | 0.00015 | 0.001558 |
| A230077H06Rik | chr7:40849152-40898324 | 8.30907 | 6.07603 | 0.731253 | 0.00055 | 0.004491 |
| Ndufb9 | chr15:58933809-58939489 | 439.106 | 321.173 | 0.731425 | 5.00E-05 | 0.000607 |
| Bola2 | chr7:126695999-126696693 | 136.145 | 99.589 | 0.731492 | 0.00025 | 0.002352 |
| Cnpy2 | chr10:128322458-128327187 | 52.7067 | 38.5668 | 0.731725 | 5.00E-05 | 0.000607 |
| Bola3 | chr6:83349483-83358392 | 39.4874 | 28.8994 | 0.731864 | 0.0044 | 0.022281 |
| Lage3 | chrX:74352161-74353618 | 25.9622 | 19.0062 | 0.732072 | 0.003 | 0.016689 |
| 2700029M09Rik | chr8:60890450-60907572 | 39.7674 | 29.1198 | 0.732253 | 5.00E-05 | 0.000607 |
| Fam109a | chr5:121849027-121854599 | 6.73656 | 4.93428 | 0.732463 | 0.00425 | 0.021761 |
| Vps25 | chr11:101253742-101259546 | 90.1998 | 66.0834 | 0.732634 | 5.00E-05 | 0.000607 |
| E130311K13Rik | chr3:63914695-63929385 | 8.78902 | 6.44039 | 0.732777 | 0.00775 | 0.034738 |
| Sh3gl3 | chr7:82260128-82307417 | 38.434 | 28.1676 | 0.732882 | 5.00E-05 | 0.000607 |
| Park2 | chr17:10840383-12063360 | 10.5473 | 7.73116 | 0.732999 | 5.00E-05 | 0.000607 |
| Rpl35a | chr16:33056452-33060188 | 775.935 | 568.801 | 0.733052 | 5.00E-05 | 0.000607 |
| Tor2a | chr2:32757233-32775383 | 23.2833 | 17.0701 | 0.733148 | 0.0001 | 0.001096 |
| Lrrc10b | chr19:10455370-10457447 | 26.465 | 19.4108 | 0.733452 | 5.00E-05 | 0.000607 |
| Caprin2 | chr6:148842511-148896126 | 6.10753 | 4.48133 | 0.733739 | 0.0012 | 0.008175 |
| Use1 | chr8:71366847-71369732 | 100.928 | 74.0559 | 0.73375 | 5.00E-05 | 0.000607 |
| Gtf3c6 | chr10:40249202-40257665 | 22.3098 | 16.3824 | 0.734314 | 0.0015 | 0.009667 |
| Gemin7 | chr7:19564948-19573343 | 34.3878 | 25.2593 | 0.734542 | 0.0011 | 0.007621 |
| Rad52 | chr6:119902697-119922823 | 15.332 | 11.262 | 0.734542 | 0.00065 | 0.005126 |
| Ccdc12 | chr9:110656502-110711593 | 69.2926 | 50.9183 | 0.73483 | 5.00E-05 | 0.000607 |
| Rps19 | chr7:24884713-24889802 | 493.87 | 362.981 | 0.734973 | 5.00E-05 | 0.000607 |
| Rpl13a | chr7:45125562-45128745 | 599.224 | 440.678 | 0.735414 | 5.00E-05 | 0.000607 |
| Bloc1s5 | chr13:38602705-38635109 | 11.0324 | 8.11626 | 0.735675 | 0.0022 | 0.013067 |
| Prkab1 | chr5:116013589-116024428 | 9.68501 | 7.12696 | 0.735875 | 0.002 | 0.012075 |
| Sytl2 | chr7:90348698-90410439 | 16.4762 | 12.1266 | 0.736007 | 5.00E-05 | 0.000607 |
| 4933439C10Rik | chr11:59485520-59511067 | 23.9902 | 17.6741 | 0.736722 | 5.00E-05 | 0.000607 |
| Amy1 | chr3:113555951-113577750 | 34.2314 | 25.2219 | 0.736806 | 5.00E-05 | 0.000607 |
| Capn3 | chr2:120355308-120504919 | 9.08629 | 6.70055 | 0.737435 | 0.0102 | 0.04303 |
| Mrps24 | chr11:5703982-5707699 | 29.7545 | 21.9487 | 0.73766 | 0.0003 | 0.002726 |
| Mrps18b | chr17:35910384-35916369 | 34.295 | 25.3032 | 0.73781 | 0.00015 | 0.001558 |
| Aldh1a1 | chr19:20601981-20643462 | 27.5535 | 20.3407 | 0.738226 | 5.00E-05 | 0.000607 |
| Snrpd2 | chr7:19149837-19152726 | 207.873 | 153.471 | 0.738292 | 5.00E-05 | 0.000607 |
| Smoc2 | chr17:14279505-14404790 | 20.9752 | 15.4865 | 0.738324 | 5.00E-05 | 0.000607 |
| Amdhd2 | chr17:24155832-24163733 | 14.626 | 10.8012 | 0.738493 | 0.0013 | 0.008656 |
| Trp53 | chr11:69580358-69591873 | 20.9877 | 15.4994 | 0.738499 | 5.00E-05 | 0.000607 |
| 1110065P20Rik | chr4:124802548-124850730 | 78.4785 | 57.9875 | 0.738897 | 0.001 | 0.007133 |
| Alg12 | chr15:88805242-88819318 | 8.95406 | 6.61722 | 0.739019 | 0.0044 | 0.022281 |
| Hmgn2 | chr4:133964738-133967991 | 123.441 | 91.231 | 0.739066 | 5.00E-05 | 0.000607 |
| Mrpl53 | chr6:83101515-83109932 | 68.6237 | 50.7364 | 0.739342 | 0.0006 | 0.004799 |
| Pts | chr9:50521616-50528641 | 39.4544 | 29.1717 | 0.739378 | 5.00E-05 | 0.000607 |
| Rps12 | chr10:23785182-23787209 | 759.817 | 561.812 | 0.739404 | 5.00E-05 | 0.000607 |
| Atp5k | chr5:108433252-108434378 | 1140.67 | 843.956 | 0.739877 | 5.00E-05 | 0.000607 |
| Rps20 | chr4:3834472-3835600 | 529.866 | 392.1 | 0.739998 | 5.00E-05 | 0.000607 |
| Amigo2 | chr15:97244073-97385691 | 14.1786 | 10.4934 | 0.740087 | 5.00E-05 | 0.000607 |
| Rpl27a | chr7:109519194-109522369 | 365.303 | 270.368 | 0.74012 | 5.00E-05 | 0.000607 |
| Rps4x | chrX:102184942-102188371 | 558.024 | 413.073 | 0.740242 | 5.00E-05 | 0.000607 |
| Zcchc7 | chr4:44756558-44932214 | 32.9936 | 24.4259 | 0.740322 | 5.00E-05 | 0.000607 |
| Zcrb1 | chr15:93386112-93398290 | 85.4925 | 63.2994 | 0.740409 | 5.00E-05 | 0.000607 |
| 1500017E21Rik | chr19:36554638-36689479 | 7.8752 | 5.83157 | 0.740498 | 0.00745 | 0.033764 |
| Zfp27 | chr7:29893336-29906104 | 7.33461 | 5.43434 | 0.740917 | 0.00025 | 0.002352 |
| Fuom | chr7:140099469-140102406 | 34.7496 | 25.7527 | 0.741093 | 0.002 | 0.012075 |
| Mrps9 | chr1:42851232-42905683 | 32.8281 | 24.3319 | 0.741191 | 5.00E-05 | 0.000607 |
| Gm3893 | chr4:41889794-42462993 | 31.906 | 23.6538 | 0.741359 | 5.00E-05 | 0.000607 |
| Ndufb2 | chr6:39592582-39599471 | 152.316 | 112.942 | 0.741498 | 5.00E-05 | 0.000607 |
| 4833420G17Rik | chr13:119462758-119486117 | 28.6225 | 21.2241 | 0.741518 | 5.00E-05 | 0.000607 |
| Tmem256 | chr11:69838524-69839558 | 123.851 | 91.8399 | 0.741535 | 0.00015 | 0.001558 |
| Sepw1 | chr7:15917207-15922371 | 1618.49 | 1200.19 | 0.741549 | 5.00E-05 | 0.000607 |
| Ctxn2 | chr2:125136691-125147841 | 14.7464 | 10.9373 | 0.741693 | 0.0089 | 0.038747 |
| Rps26 | chr10:128624528-128626506 | 494.676 | 367.034 | 0.741968 | 5.00E-05 | 0.000607 |
| Acot13 | chr13:24817954-24831489 | 168.729 | 125.194 | 0.741983 | 5.00E-05 | 0.000607 |
| Snrpf | chr10:93540631-93605245 | 38.2919 | 28.4252 | 0.742329 | 0.00095 | 0.006869 |
| Rps7 | chr12:28630846-28635953 | 470.548 | 349.355 | 0.742443 | 5.00E-05 | 0.000607 |
| Fcf1 | chr12:84970929-84983303 | 35.2081 | 26.1534 | 0.742823 | 0.00185 | 0.011363 |
| 3010026O09Rik | chr11:50174850-50200115 | 15.4244 | 11.4578 | 0.742836 | 0.0067 | 0.031027 |
| Mrfap1 | chr5:36794866-36796754 | 322.172 | 239.842 | 0.744453 | 5.00E-05 | 0.000607 |
| Ppp1r1a | chr15:103530278-103537992 | 102.929 | 76.6317 | 0.74451 | 5.00E-05 | 0.000607 |
| Klk8 | chr7:43797576-43803822 | 11.6854 | 8.71386 | 0.745705 | 0.0076 | 0.034268 |
| Tmem158 | chr9:123259056-123260789 | 13.5117 | 10.077 | 0.745798 | 0.0013 | 0.008656 |
| Zfand2b | chr1:75168645-75171626 | 30.1677 | 22.5038 | 0.745957 | 0.0001 | 0.001096 |
| Cenpv | chr11:62524943-62539261 | 33.7614 | 25.1872 | 0.746035 | 0.0001 | 0.001096 |
| Wipf3 | chr6:54452882-54503768 | 167.95 | 125.341 | 0.746299 | 5.00E-05 | 0.000607 |
| Hypk | chr2:121457087-121458440 | 133.183 | 99.4172 | 0.746471 | 5.00E-05 | 0.000607 |
| Znrd1 | chr17:36954357-36958428 | 31.3149 | 23.3787 | 0.746568 | 0.00385 | 0.02021 |
| Deb1 | chr9:121710388-121712921 | 82.7118 | 61.7523 | 0.746596 | 0.0003 | 0.002726 |
| Aimp1 | chr3:132660497-132683879 | 62.0188 | 46.3061 | 0.746646 | 5.00E-05 | 0.000607 |
| Mrpl24 | chr3:87919543-87930195 | 36.9662 | 27.6214 | 0.747207 | 0.00665 | 0.030876 |
| Tnip2 | chr5:34496095-34513979 | 19.7079 | 14.7289 | 0.74736 | 5.00E-05 | 0.000607 |
| Anapc13 | chr9:102626295-102634244 | 111.926 | 83.6566 | 0.747428 | 0.00035 | 0.003088 |
| Stk38 | chr17:28970884-29007937 | 22.1957 | 16.6003 | 0.747906 | 5.00E-05 | 0.000607 |
| Rps3a1 | chr3:86137939-86142668 | 633.986 | 474.164 | 0.747909 | 5.00E-05 | 0.000607 |
| D4Wsu53e | chr4:134923624-134927370 | 188.226 | 140.886 | 0.748494 | 5.00E-05 | 0.000607 |
| Snrnp35 | chr5:124483154-124491122 | 15.0483 | 11.2649 | 0.748583 | 0.0086 | 0.03775 |
| Slco1a4 | chr6:141805439-141856171 | 20.5656 | 15.4051 | 0.749071 | 5.00E-05 | 0.000607 |
| Ccdc107 | chr4:43493364-43495921 | 41.5496 | 31.1563 | 0.749858 | 0.00025 | 0.002352 |
| Rbak | chr5:143172188-143180743 | 8.07361 | 6.05445 | 0.749906 | 0.00055 | 0.004491 |
| Ldlr | chr9:21723575-21749918 | 4.91292 | 3.68456 | 0.749974 | 0.0017 | 0.010645 |
| 3230401D17Rik | chr2:163405821-163419470 | 15.7255 | 11.7958 | 0.750107 | 0.00135 | 0.008911 |
| Rps8 | chr4:117153835-117156132 | 661.873 | 496.801 | 0.750599 | 5.00E-05 | 0.000607 |
| Zfp787 | chr7:6131488-6155971 | 7.59013 | 5.70063 | 0.751058 | 0.0101 | 0.04271 |
| 0610011F06Rik | chr17:25875499-25877163 | 28.6031 | 21.4854 | 0.751156 | 0.00305 | 0.01694 |
| Ndufb5 | chr3:32737062-32751559 | 162.04 | 121.751 | 0.751364 | 5.00E-05 | 0.000607 |
| Ryr1 | chr7:29003339-29125151 | 6.21385 | 4.67373 | 0.752147 | 5.00E-05 | 0.000607 |
| Rnasek | chr11:70238122-70239852 | 260.74 | 196.179 | 0.752393 | 5.00E-05 | 0.000607 |
| Pin4 | chrX:102119464-102127673 | 84.4659 | 63.552 | 0.752398 | 0.00075 | 0.005715 |
| Rpl14 | chr9:120571516-120574653 | 434.626 | 327.076 | 0.752546 | 5.00E-05 | 0.000607 |
| Arhgef25 | chr10:127182520-127190054 | 241.736 | 181.96 | 0.752722 | 5.00E-05 | 0.000607 |
| Rtn4ip1 | chr10:43901806-43947862 | 7.36361 | 5.54335 | 0.752803 | 0.002 | 0.012075 |
| Rpusd3 | chr6:113415318-113419340 | 13.5995 | 10.243 | 0.753189 | 0.0073 | 0.03314 |
| Fam58b | chr11:78750505-78751729 | 15.0931 | 11.3715 | 0.753424 | 0.00575 | 0.027666 |
| Timmdc1 | chr16:38497842-38522663 | 12.7977 | 9.64774 | 0.753865 | 0.00055 | 0.004491 |
| Ndufaf3 | chr9:108565864-108567342 | 39.8544 | 30.0607 | 0.754263 | 0.00015 | 0.001558 |
| Thoc7 | chr14:13949013-13961225 | 72.0955 | 54.3815 | 0.754298 | 5.00E-05 | 0.000607 |
| Slmo1 | chr18:67464848-67480581 | 25.5507 | 19.2832 | 0.754703 | 5.00E-05 | 0.000607 |
| Rft1 | chr14:30654374-30691313 | 7.47133 | 5.64043 | 0.754943 | 0.0046 | 0.023162 |
| N6amt1 | chr16:87354184-87368649 | 15.7146 | 11.8653 | 0.755049 | 0.00065 | 0.005126 |
| Traip | chr9:107950962-107972268 | 33.0938 | 25.0066 | 0.755628 | 5.00E-05 | 0.000607 |
| Rps24 | chr14:24490680-24496146 | 679.495 | 513.455 | 0.755642 | 5.00E-05 | 0.000607 |
| 2810468N07Rik | chr17:25570810-25575043 | 25.4355 | 19.2259 | 0.755869 | 0.00055 | 0.004491 |
| Usp2 | chr9:44067020-44095627 | 24.203 | 18.3011 | 0.75615 | 5.00E-05 | 0.000607 |
| Myeov2 | chr1:92637144-92641985 | 330.054 | 249.586 | 0.756197 | 5.00E-05 | 0.000607 |
| Krt2 | chr15:101810688-101818169 | 6.57195 | 4.96993 | 0.756234 | 0.0055 | 0.026705 |
| Cln3 | chr7:126571399-126584280 | 9.1469 | 6.92011 | 0.756552 | 0.00225 | 0.013268 |
| Nfkbiz | chr16:55811376-55838641 | 4.83118 | 3.65515 | 0.756575 | 0.00585 | 0.028071 |
| Mrpl27 | chr11:94653790-94660087 | 85.5437 | 64.7257 | 0.756639 | 5.00E-05 | 0.000607 |
| Slc7a6 | chr8:106168874-106198704 | 17.1989 | 13.0164 | 0.756816 | 5.00E-05 | 0.000607 |
| Rps25 | chr9:44407713-44418007 | 551.763 | 417.588 | 0.756825 | 5.00E-05 | 0.000607 |
| Spata7 | chr12:98628183-98669814 | 13.692 | 10.3684 | 0.75726 | 0.0011 | 0.007621 |
| Cox17 | chr16:38346998-38352763 | 161.887 | 122.595 | 0.757287 | 0.0001 | 0.001096 |
| Flt3 | chr5:147330741-147400489 | 5.50933 | 4.17229 | 0.757314 | 0.00315 | 0.017359 |
| Zfp639 | chr3:32510549-32520833 | 22.3372 | 16.9208 | 0.757517 | 5.00E-05 | 0.000607 |
| Psmb6 | chr11:70525356-70527858 | 140.63 | 106.565 | 0.757769 | 5.00E-05 | 0.000607 |
| Ercc6l2 | chr13:63815319-63900301 | 6.44284 | 4.88392 | 0.758038 | 0.0002 | 0.001971 |
| Zbtb8os | chr4:129336025-129347029 | 29.4115 | 22.3 | 0.758207 | 0.0096 | 0.04107 |
| Ndufs4 | chr13:114287794-114388094 | 71.1512 | 54.0216 | 0.759251 | 5.00E-05 | 0.000607 |
| Slc50a1 | chr3:89268245-89270570 | 56.6111 | 42.984 | 0.759286 | 5.00E-05 | 0.000607 |
| 1500032L24Rik | chr15:82346045-82349062 | 223.503 | 169.72 | 0.759363 | 5.00E-05 | 0.000607 |
| Ggct | chr6:54985094-54992867 | 15.9235 | 12.095 | 0.759569 | 0.00405 | 0.021031 |
| Phpt1 | chr2:25573430-25574871 | 110.88 | 84.2254 | 0.759609 | 5.00E-05 | 0.000607 |
| Wibg | chr10:128747878-128766568 | 23.807 | 18.0842 | 0.759617 | 0.00175 | 0.010881 |
| Pfdn5 | chr15:102326115-102331489 | 383.608 | 291.504 | 0.759901 | 5.00E-05 | 0.000607 |
| Crtc2 | chr3:90254280-90264125 | 19.6213 | 14.9128 | 0.760031 | 5.00E-05 | 0.000607 |
| Mettl8 | chr2:70964561-71055606 | 10.5968 | 8.05508 | 0.760143 | 0.0043 | 0.021953 |
| Ssbp1 | chr6:40471414-40481823 | 22.2661 | 16.9267 | 0.7602 | 0.0016 | 0.010187 |
| Hipk2 | chr6:38697839-38876190 | 4.64907 | 3.53509 | 0.760386 | 0.00465 | 0.023325 |
| Spag5 | chr11:78301590-78322454 | 6.06158 | 4.60934 | 0.760419 | 0.00195 | 0.011841 |
| Rn45s | chr17:39842996-39848829 | 57.044 | 43.3832 | 0.760522 | 5.00E-05 | 0.000607 |
| Egfl7 | chr2:26581055-26592682 | 34.8309 | 26.4921 | 0.760592 | 0.00015 | 0.001558 |
| Rpl36al | chr12:69182733-69184067 | 268.089 | 203.937 | 0.760706 | 5.00E-05 | 0.000607 |
| Mrpl11 | chr19:4962305-4966995 | 21.9423 | 16.6923 | 0.760736 | 5.00E-05 | 0.000607 |
| Prmt3 | chr7:49778357-49858265 | 12.0724 | 9.18432 | 0.76077 | 0.0006 | 0.004799 |
| BC027231 | chr16:44724300-44737284 | 5.58948 | 4.25358 | 0.760997 | 0.00885 | 0.038577 |
| 1700021F05Rik | chr10:43525120-43540994 | 36.7033 | 27.9354 | 0.761114 | 0.00075 | 0.005715 |
| Crlf1 | chr8:70493155-70504081 | 25.8409 | 19.68 | 0.761583 | 0.0001 | 0.001096 |
| Rpl41 | chr10:128548109-128549168 | 1689.01 | 1286.93 | 0.761943 | 5.00E-05 | 0.000607 |
| Arl6ip4 | chr5:124116107-124118195 | 48.6483 | 37.0699 | 0.761998 | 5.00E-05 | 0.000607 |
| Drap1 | chr19:5406873-5424916 | 268.163 | 204.447 | 0.762398 | 5.00E-05 | 0.000607 |
| Mrpl42 | chr10:95480805-95501927 | 99.8802 | 76.1518 | 0.762431 | 5.00E-05 | 0.000607 |
| Tbca | chr13:94788942-94842899 | 162.985 | 124.29 | 0.762586 | 5.00E-05 | 0.000607 |
| Rpl9 | chr5:65388363-65391431 | 871.387 | 664.507 | 0.762585 | 5.00E-05 | 0.000607 |
| Lars2 | chr9:123366939-123462664 | 20.9797 | 16.0053 | 0.762895 | 5.00E-05 | 0.000607 |
| Plekhj1 | chr10:80796098-80798626 | 26.0803 | 19.905 | 0.76322 | 0.00085 | 0.0063 |
| Ofd1 | chrX:166390032-166440704 | 4.31647 | 3.29501 | 0.763358 | 0.00435 | 0.022102 |
| Zfp940 | chr7:29843935-29853648 | 9.33692 | 7.12742 | 0.763359 | 0.00125 | 0.008413 |
| Tsc22d4 | chr5:137745968-137768453 | 34.3231 | 26.2076 | 0.763556 | 5.00E-05 | 0.000607 |
| Higd2a | chr13:54590230-54591147 | 218.29 | 166.707 | 0.763695 | 5.00E-05 | 0.000607 |
| Ndufs3 | chr2:90894635-90904721 | 174.609 | 133.351 | 0.763712 | 5.00E-05 | 0.000607 |
| Mrpl23 | chr7:142533116-142540742 | 88.7154 | 67.7531 | 0.763713 | 0.0001 | 0.001096 |
| Slc22a5 | chr11:53864541-53891703 | 9.4508 | 7.2193 | 0.763882 | 0.00095 | 0.006869 |
| Pdlim2 | chr14:70164217-70177672 | 15.9501 | 12.1882 | 0.764146 | 0.00255 | 0.014638 |
| Cecr5 | chr6:120509493-120531299 | 26.5658 | 20.3008 | 0.76417 | 5.00E-05 | 0.000607 |
| Ndufv3 | chr17:31520114-31531325 | 241.512 | 184.581 | 0.764273 | 5.00E-05 | 0.000607 |
| Gfra4 | chr2:131039631-131043088 | 56.9162 | 43.5056 | 0.76438 | 5.00E-05 | 0.000607 |
| Rpl7 | chr1:16101295-16104433 | 499.876 | 382.148 | 0.764486 | 5.00E-05 | 0.000607 |
| Cstb | chr10:78425669-78427622 | 49.751 | 38.0345 | 0.764497 | 0.0026 | 0.014885 |
| Uba52 | chr8:70508265-70510367 | 1574.71 | 1203.87 | 0.764503 | 5.00E-05 | 0.000607 |
| Fis1 | chr5:136953274-136966234 | 226.434 | 173.141 | 0.764642 | 5.00E-05 | 0.000607 |
| Cc2d2a | chr5:43662378-43740970 | 7.35659 | 5.62656 | 0.764833 | 0.00015 | 0.001558 |
| Olfm1 | chr2:28193092-28230736 | 1126.15 | 896.101 | 0.795721 | 0.003 | 0.016689 |
| Rpl24 | chr16:55966274-55971437 | 472.785 | 361.662 | 0.764961 | 5.00E-05 | 0.000607 |
| Rpl39 | chrX:37082519-37085184 | 567.221 | 433.969 | 0.765079 | 5.00E-05 | 0.000607 |
| Rpl28 | chr7:4792964-4794547 | 667.435 | 510.675 | 0.765131 | 5.00E-05 | 0.000607 |
| Tssc4 | chr7:143069367-143071087 | 33.9813 | 26.0003 | 0.765136 | 0.00015 | 0.001558 |
| Cox5a | chr9:57521231-57532426 | 310.268 | 237.463 | 0.765348 | 5.00E-05 | 0.000607 |
| Chchd2 | chr5:129881160-129887470 | 276.358 | 211.541 | 0.76546 | 5.00E-05 | 0.000607 |
| 9130401M01Rik | chr15:58022270-58034294 | 30.3706 | 23.2477 | 0.765467 | 0.0001 | 0.001096 |
| Zfp61 | chr7:24291045-24299549 | 11.7046 | 8.96018 | 0.765526 | 0.00125 | 0.008413 |
| Grb14 | chr2:64912481-65022766 | 28.069 | 21.4963 | 0.765838 | 5.00E-05 | 0.000607 |
| Ccdc57 | chr11:120826541-120932872 | 6.61661 | 5.0684 | 0.766012 | 0.0033 | 0.017971 |
| Nrgn | chr9:37544492-37552745 | 1141.91 | 874.974 | 0.766237 | 0.0001 | 0.001096 |
| Ryr3 | chr2:112631381-113030331 | 15.5724 | 11.9337 | 0.766337 | 5.00E-05 | 0.000607 |
| Gpatch4 | chr3:88043107-88055992 | 22.7356 | 17.4265 | 0.766485 | 0.00025 | 0.002352 |
| Rprml | chr11:103649508-103650580 | 44.6745 | 34.2481 | 0.766614 | 0.0002 | 0.001971 |
| Eif3f | chr7:108934414-108941942 | 108.999 | 83.5623 | 0.766634 | 5.00E-05 | 0.000607 |
| Minos1 | chr4:139101813-139131113 | 45.6724 | 35.0148 | 0.766651 | 5.00E-05 | 0.000607 |
| Slc25a20 | chr9:108662097-108684641 | 9.6275 | 7.38189 | 0.76675 | 0.0102 | 0.04303 |
| Zfp661 | chr2:127575532-127584677 | 6.55675 | 5.02788 | 0.766825 | 0.0069 | 0.031704 |
| Pcp4 | chr16:96467605-96525793 | 394.381 | 302.468 | 0.766944 | 5.00E-05 | 0.000607 |
| Imp3 | chr9:56937499-56938398 | 43.0022 | 32.9858 | 0.767072 | 0.00055 | 0.004491 |
| Tbce | chr13:13954673-14039600 | 49.3087 | 37.8259 | 0.767124 | 5.00E-05 | 0.000607 |
| Plgrkt | chr19:29348676-29361871 | 24.6686 | 18.9253 | 0.767182 | 0.0066 | 0.030711 |
| Rpl34-ps1 | chr3:130726830-130730329 | 617.425 | 473.7 | 0.767219 | 5.00E-05 | 0.000607 |
| Znhit2 | chr19:6061206-6062468 | 22.3018 | 17.1137 | 0.767369 | 0.0017 | 0.010645 |
| Rpl36 | chr17:56613394-56614246 | 349.93 | 268.542 | 0.767416 | 5.00E-05 | 0.000607 |
| Mcm7 | chr5:138164588-138171862 | 8.27708 | 6.35316 | 0.767561 | 0.00485 | 0.024168 |
| Timm8b | chr9:50603900-50625000 | 296.013 | 227.223 | 0.767612 | 5.00E-05 | 0.000607 |
| Rpl12 | chr2:32961711-32964045 | 585.122 | 449.146 | 0.767611 | 5.00E-05 | 0.000607 |
| Pvrl3 | chr16:46394857-46496967 | 8.07535 | 6.19934 | 0.767687 | 0.004 | 0.020812 |
| Tmem181c-ps | chr17:6610102-6620925 | 24.6182 | 18.9021 | 0.76781 | 5.00E-05 | 0.000607 |
| Sec11a | chr7:80915378-80947550 | 31.7365 | 24.3681 | 0.767826 | 0.00055 | 0.004491 |
| Uqcr10 | chr11:4701967-4704344 | 490.036 | 376.291 | 0.767884 | 5.00E-05 | 0.000607 |
| Nphp1 | chr2:127740731-127788854 | 12.3586 | 9.49123 | 0.767986 | 0.0011 | 0.007621 |
| Atox1 | chr11:55446642-55461138 | 147.167 | 113.024 | 0.767998 | 0.0002 | 0.001971 |
| Tomm22 | chr15:79670867-79672862 | 110.824 | 85.1231 | 0.768093 | 5.00E-05 | 0.000607 |
| Sec61b | chr4:47474660-47483233 | 48.6127 | 37.3404 | 0.76812 | 0.006 | 0.028609 |
| Cyp4f15 | chr17:32685658-32703349 | 8.49675 | 6.5289 | 0.7684 | 0.00705 | 0.032226 |
| Opalin | chr19:41063419-41077113 | 30.1216 | 23.1474 | 0.768465 | 0.00265 | 0.01513 |
| Trim39 | chr17:36258872-36272004 | 19.8041 | 15.2249 | 0.768775 | 5.00E-05 | 0.000607 |
| Arf5 | chr6:28423639-28426499 | 143.257 | 110.149 | 0.768891 | 5.00E-05 | 0.000607 |
| Phyhd1 | chr2:30266528-30282149 | 23.0395 | 17.7168 | 0.768975 | 0.00155 | 0.009917 |
| Slc9a2 | chr1:40681711-40768885 | 17.9533 | 13.8098 | 0.769207 | 0.0001 | 0.001096 |
| Tppp3 | chr8:105467491-105471422 | 36.0021 | 27.6952 | 0.769266 | 0.00075 | 0.005715 |
| Dusp7 | chr9:106368631-106375723 | 23.1644 | 17.8201 | 0.769288 | 5.00E-05 | 0.000607 |
| Gins4 | chr8:23226609-23237668 | 13.2903 | 10.2247 | 0.769336 | 0.01035 | 0.043473 |
| Gng13 | chr17:25717171-25727415 | 115.085 | 88.5532 | 0.769459 | 0.00125 | 0.008413 |
| Focad | chr4:88094629-88411011 | 20.2861 | 15.6107 | 0.769527 | 5.00E-05 | 0.000607 |
| Cdc42ep1 | chr15:78842646-78855529 | 7.74455 | 5.95979 | 0.769546 | 0.00605 | 0.02877 |
| Ndufb11 | chrX:20615325-20650905 | 197.995 | 152.375 | 0.76959 | 0.0003 | 0.002726 |
| Tor1a | chr2:30960560-30967918 | 37.1185 | 28.5687 | 0.769662 | 0.0001 | 0.001096 |
| Zfp286 | chr11:62778386-62789417 | 9.35675 | 7.20401 | 0.769927 | 0.00095 | 0.006869 |
| Rpl36a | chrX:134585653-134588062 | 429.549 | 330.764 | 0.770026 | 5.00E-05 | 0.000607 |
| Cryl1 | chr14:57275033-57398483 | 16.8022 | 12.9385 | 0.770048 | 0.0048 | 0.023953 |
| Cox8a | chr19:7215157-7217616 | 1000.73 | 770.72 | 0.770158 | 5.00E-05 | 0.000607 |
| Rgs14 | chr13:55369731-55384687 | 51.6139 | 39.7577 | 0.770291 | 5.00E-05 | 0.000607 |
| Pisd-ps1 | chr11:3124020-3193463 | 274.907 | 211.783 | 0.770381 | 5.00E-05 | 0.000607 |
| Eif1 | chr11:100319995-100322096 | 467.464 | 360.158 | 0.770451 | 5.00E-05 | 0.000607 |
| Gpc4 | chrX:52053017-52164923 | 18.057 | 13.9125 | 0.770477 | 0.0003 | 0.002726 |
| Robo3 | chr9:37416044-37433175 | 10.5509 | 8.13196 | 0.770736 | 5.00E-05 | 0.000607 |
| Znhit1 | chr5:136982200-136996646 | 43.6047 | 33.6095 | 0.770777 | 0.00775 | 0.034738 |
| Mtfmt | chr9:65435781-65453054 | 8.51543 | 6.56436 | 0.770878 | 0.0105 | 0.043929 |
| Bckdha | chr7:25629851-25658761 | 31.7521 | 24.4773 | 0.770888 | 5.00E-05 | 0.000607 |
| Ndufs6 | chr13:73319875-73328482 | 186.746 | 144.16 | 0.771958 | 5.00E-05 | 0.000607 |
| Slc25a29 | chr12:108825877-108835876 | 11.3108 | 8.73255 | 0.772054 | 0.00445 | 0.022502 |
| Rpl21 | chr5:146832889-146837032 | 338.485 | 261.357 | 0.772138 | 5.00E-05 | 0.000607 |
| Rpl37 | chr15:5116612-5119140 | 369.055 | 284.976 | 0.772178 | 5.00E-05 | 0.000607 |
| Prr18 | chr17:8340405-8344113 | 20.2904 | 15.6717 | 0.77237 | 5.00E-05 | 0.000607 |
| Neurod2 | chr11:98325416-98329645 | 53.4691 | 41.2993 | 0.772396 | 5.00E-05 | 0.000607 |
| Mpc2 | chr1:165461207-165481214 | 115.52 | 89.231 | 0.772429 | 5.00E-05 | 0.000607 |
| Tomm5 | chr4:45105209-45108113 | 79.3149 | 61.2718 | 0.772513 | 5.00E-05 | 0.000607 |
| Med10 | chr13:69809881-69816094 | 49.974 | 38.6057 | 0.772516 | 0.00045 | 0.003815 |
| Cox6c | chr15:35931975-35938246 | 914.844 | 706.763 | 0.77255 | 5.00E-05 | 0.000607 |
| Metap1d | chr2:71453337-71525191 | 31.0266 | 23.9715 | 0.772611 | 0.0004 | 0.00346 |
| Atp5h | chr11:115415696-115419919 | 677.518 | 523.566 | 0.772771 | 5.00E-05 | 0.000607 |
| Zfp772 | chr7:7202121-7209998 | 11.3528 | 8.77524 | 0.772958 | 0.00085 | 0.0063 |
| Exosc8 | chr3:54728678-54735364 | 15.7549 | 12.1782 | 0.772979 | 0.00755 | 0.034086 |
| Mrpl13 | chr15:55534094-55557312 | 64.4831 | 49.8443 | 0.772982 | 0.00025 | 0.002352 |
| Tmem63a | chr1:180942517-180975104 | 12.9647 | 10.0219 | 0.773014 | 0.00035 | 0.003088 |
| Pvalb | chr15:78191117-78206351 | 55.1001 | 42.5993 | 0.773126 | 0.00035 | 0.003088 |
| Rps6 | chr4:86854098-86857367 | 316.932 | 245.11 | 0.773384 | 5.00E-05 | 0.000607 |
| Mnf1 | chr17:27122664-27133891 | 253.867 | 196.345 | 0.773417 | 5.00E-05 | 0.000607 |
| Ddt | chr10:75771232-75773374 | 65.1918 | 50.4248 | 0.773484 | 0.001 | 0.007133 |
| Klhdc4 | chr8:121796307-121829569 | 19.5443 | 15.121 | 0.773678 | 5.00E-05 | 0.000607 |
| Ndufa4 | chr6:11900372-11907446 | 946.869 | 732.696 | 0.773809 | 5.00E-05 | 0.000607 |
| Rcor2 | chr19:7269763-7275225 | 9.38066 | 7.26333 | 0.774288 | 0.00915 | 0.039575 |
| Usmg5 | chr19:47067747-47090625 | 1015.72 | 786.684 | 0.774509 | 5.00E-05 | 0.000607 |
| Gmpr2 | chr14:55672234-55678751 | 19.6863 | 15.2519 | 0.774747 | 0.00095 | 0.006869 |
| Uqcr11 | chr10:80402996-80406821 | 538.983 | 417.651 | 0.774887 | 5.00E-05 | 0.000607 |
| Yipf1 | chr4:107314362-107359823 | 36.0751 | 27.9548 | 0.774906 | 0.00015 | 0.001558 |
| Mcat | chr15:83546796-83555711 | 14.7928 | 11.4634 | 0.774931 | 0.0018 | 0.011147 |
| Rpl19 | chr11:98026709-98030493 | 776.64 | 602.022 | 0.775162 | 5.00E-05 | 0.000607 |
| Dguok | chr6:83480213-83506969 | 17.0419 | 13.211 | 0.775207 | 0.01105 | 0.04553 |
| Rogdi | chr16:5008728-5013553 | 249.086 | 193.108 | 0.775266 | 5.00E-05 | 0.000607 |
| Rps23 | chr13:90923121-90924732 | 736.391 | 571.475 | 0.776048 | 5.00E-05 | 0.000607 |
| Glt8d1 | chr14:30999825-31019089 | 30.1712 | 23.4196 | 0.776224 | 0.00965 | 0.041251 |
| Kcnip2 | chr19:45792345-45816063 | 181.028 | 140.61 | 0.776731 | 5.00E-05 | 0.000607 |
| Mt1 | chr8:94179088-94180327 | 745.19 | 579.064 | 0.777069 | 5.00E-05 | 0.000607 |
| 1110038F14Rik | chr15:76948543-76950731 | 21.4265 | 16.6501 | 0.77708 | 0.0055 | 0.026705 |
| Klhdc9 | chr1:171358448-171360798 | 14.0953 | 10.9565 | 0.777316 | 0.00765 | 0.034391 |
| Mrpl55 | chr11:59202516-59206002 | 42.2955 | 32.8787 | 0.777357 | 0.00235 | 0.013705 |
| Pagr1a | chr7:127015050-127017352 | 75.0048 | 58.3067 | 0.777373 | 5.00E-05 | 0.000607 |
| Matn2 | chr15:34306680-34436240 | 18.4049 | 14.3091 | 0.777461 | 5.00E-05 | 0.000607 |
| Rps14 | chr18:60774595-60778546 | 790.67 | 614.73 | 0.77748 | 5.00E-05 | 0.000607 |
| Pycr2 | chr1:180904273-180908088 | 26.5707 | 20.6595 | 0.777529 | 0.00045 | 0.003815 |
| Eef1b2 | chr1:63176830-63180486 | 124.033 | 96.4625 | 0.777716 | 5.00E-05 | 0.000607 |
| Cd276 | chr9:58524299-58540940 | 5.59716 | 4.35464 | 0.778009 | 0.0123 | 0.049696 |
| Msh3 | chr13:92211880-92389053 | 8.37484 | 6.51572 | 0.778011 | 0.0017 | 0.010645 |
| Rbm5 | chr9:107740494-107771002 | 89.0861 | 69.3196 | 0.778119 | 5.00E-05 | 0.000607 |
| Commd4 | chr9:57155040-57158299 | 77.2826 | 60.1611 | 0.778456 | 5.00E-05 | 0.000607 |
| Thrsp | chr7:97412956-97417510 | 58.7693 | 45.7543 | 0.778541 | 0.00015 | 0.001558 |
| Rgs10 | chr7:128373624-128418172 | 40.487 | 31.5239 | 0.778618 | 0.00175 | 0.010881 |
| Cnih2 | chr19:5088537-5098418 | 366.451 | 285.361 | 0.778715 | 5.00E-05 | 0.000607 |
| Btf3 | chr13:98309896-98317006 | 212.995 | 165.88 | 0.778798 | 5.00E-05 | 0.000607 |
| Phka2 | chrX:160502165-160598878 | 12.0289 | 9.37013 | 0.778968 | 5.00E-05 | 0.000607 |
| Arpc5l | chr2:39008138-39015872 | 83.8984 | 65.3544 | 0.778971 | 5.00E-05 | 0.000607 |
| Polr2i | chr7:30232073-30233387 | 31.8417 | 24.8038 | 0.778972 | 0.01195 | 0.048485 |
| Rnf112 | chr11:61448441-61453886 | 340.877 | 265.603 | 0.779175 | 5.00E-05 | 0.000607 |
| Mrps21 | chr3:95862651-95870619 | 156.136 | 121.674 | 0.779282 | 0.00055 | 0.004491 |
| Cox6a1 | chr5:115345653-115348955 | 821.594 | 640.438 | 0.779507 | 5.00E-05 | 0.000607 |
| Rps10 | chr17:27630428-27635242 | 493.355 | 384.594 | 0.779548 | 5.00E-05 | 0.000607 |
| Wdr73 | chr7:80890722-80901269 | 21.2874 | 16.5962 | 0.779626 | 0.00035 | 0.003088 |
| Rps18 | chr17:33951998-33955641 | 612.053 | 477.234 | 0.779727 | 5.00E-05 | 0.000607 |
| 1810013D10Rik | chr5:53267105-53278540 | 26.2545 | 20.4715 | 0.779733 | 0.0059 | 0.028247 |
| Efha1 | chr14:57916279-57999262 | 14.0377 | 10.9469 | 0.779821 | 0.00145 | 0.009402 |
| Rpl27 | chr11:101442244-101445596 | 278.504 | 217.288 | 0.780197 | 5.00E-05 | 0.000607 |
| Cacna1h | chr17:25370552-25433783 | 34.6837 | 27.0685 | 0.780439 | 5.00E-05 | 0.000607 |
| Mrpl36 | chr13:73331008-73332178 | 33.5286 | 26.1682 | 0.780474 | 0.00325 | 0.017763 |
| Cox7a2 | chr9:79755240-79759853 | 352.517 | 275.152 | 0.780535 | 5.00E-05 | 0.000607 |
| 2010300C02Rik | chr1:37611675-37719811 | 122.313 | 95.4733 | 0.780565 | 5.00E-05 | 0.000607 |
| Msra | chr14:64122620-64455903 | 69.3175 | 54.132 | 0.780928 | 5.00E-05 | 0.000607 |
| Shfm1 | chr6:6558274-6578658 | 190.076 | 148.458 | 0.781045 | 5.00E-05 | 0.000607 |
| Rpl31 | chr1:39367850-39478747 | 394.82 | 308.456 | 0.781257 | 5.00E-05 | 0.000607 |
| Dgkz | chr2:91932821-91963563 | 208.151 | 162.62 | 0.78126 | 5.00E-05 | 0.000607 |
| Rad50 | chr11:53649518-53707319 | 4.95668 | 3.87251 | 0.781271 | 0.0032 | 0.01758 |
| Mog | chr17:37010739-37023398 | 46.1331 | 36.0458 | 0.781344 | 5.00E-05 | 0.000607 |
| Mrpl52 | chr14:54426908-54429750 | 70.4655 | 55.0593 | 0.781365 | 0.00975 | 0.041578 |
| Cox5b | chr1:36691486-36693388 | 590.389 | 461.338 | 0.781414 | 5.00E-05 | 0.000607 |
| 0610010K14Rik | chr11:70235203-70237914 | 40.2002 | 31.4215 | 0.781625 | 0.0031 | 0.017146 |
| Sertad4 | chr1:192844487-192855752 | 6.70716 | 5.24282 | 0.781675 | 0.00795 | 0.03541 |
| Rpl5 | chr5:107900527-107987077 | 202.731 | 158.5 | 0.781824 | 5.00E-05 | 0.000607 |
| Ovgp1 | chr3:105973801-105987423 | 8.63067 | 6.75044 | 0.782146 | 0.00565 | 0.027333 |
| Adam8 | chr7:139978940-139992488 | 6.76476 | 5.29118 | 0.782168 | 0.00795 | 0.03541 |
| Tmem8 | chr17:26113315-26123253 | 7.94407 | 6.21547 | 0.782404 | 0.0029 | 0.016217 |
| Avpi1 | chr19:42123274-42128993 | 21.8984 | 17.1338 | 0.782422 | 0.0109 | 0.045122 |
| Matk | chr10:81257544-81262981 | 191.132 | 149.548 | 0.782433 | 5.00E-05 | 0.000607 |
| Itpa | chr2:130667840-130681614 | 67.1571 | 52.5513 | 0.782513 | 5.00E-05 | 0.000607 |
| Fbxw4 | chr19:45560614-45660193 | 20.714 | 16.2163 | 0.782867 | 0.0089 | 0.038747 |
| Rps9 | chr7:3704040-3706897 | 541.263 | 423.741 | 0.782874 | 5.00E-05 | 0.000607 |
| Hint1 | chr11:54866437-54870496 | 457.894 | 358.546 | 0.783033 | 5.00E-05 | 0.000607 |
| Dbndd2 | chr2:164486139-164493323 | 55.2874 | 43.3065 | 0.783298 | 5.00E-05 | 0.000607 |
| Cisd1 | chr10:71330493-71344849 | 137.144 | 107.557 | 0.784263 | 5.00E-05 | 0.000607 |
| 2310036O22Rik | chr8:85026832-85030286 | 74.3144 | 58.2828 | 0.784273 | 5.00E-05 | 0.000607 |
| Echdc2 | chr4:108165436-108179308 | 26.6163 | 20.8752 | 0.784301 | 0.00245 | 0.014171 |
| Plekha2 | chr8:25039143-25101811 | 11.1718 | 8.76426 | 0.784498 | 5.00E-05 | 0.000607 |
| Glt25d1 | chr8:71611023-71624911 | 39.4352 | 30.9377 | 0.78452 | 5.00E-05 | 0.000607 |
| Pomp | chr5:147860627-147875778 | 204.445 | 160.464 | 0.784876 | 5.00E-05 | 0.000607 |
| Adam1a | chr5:121518603-121521695 | 9.45147 | 7.41997 | 0.78506 | 0.0027 | 0.01535 |
| Rpl22l1 | chr3:28805510-28807415 | 190.535 | 149.597 | 0.785142 | 0.0001 | 0.001096 |
| Cdc40 | chr10:40831621-40883143 | 24.5892 | 19.3156 | 0.785532 | 5.00E-05 | 0.000607 |
| Ndufa13 | chr8:69894181-69903518 | 177.942 | 139.895 | 0.786183 | 5.00E-05 | 0.000607 |
| Trmt1 | chr8:84689246-84699808 | 51.6386 | 40.6163 | 0.786549 | 5.00E-05 | 0.000607 |
| Tbcc | chr17:46890620-46892463 | 11.6041 | 9.12738 | 0.786565 | 0.0092 | 0.039743 |
| Elovl1 | chr4:118428092-118437343 | 18.1204 | 14.2539 | 0.786622 | 0.002 | 0.012075 |
| Fam96b | chr8:104639838-104641728 | 36.0553 | 28.3631 | 0.786655 | 0.0118 | 0.047968 |
| Fopnl | chr16:14299243-14317332 | 36.1093 | 28.4142 | 0.786894 | 0.0002 | 0.001971 |
| Ndufa3 | chr7:3617372-3620161 | 630.751 | 496.617 | 0.787342 | 5.00E-05 | 0.000607 |
| Gm14827 | chrX:94442731-94447727 | 10.8414 | 8.53712 | 0.787455 | 0.0017 | 0.010645 |
| Rps16 | chr7:28350688-28352698 | 477.597 | 376.095 | 0.787474 | 5.00E-05 | 0.000607 |
| Fbxo6 | chr4:148145715-148152135 | 21.5895 | 17.003 | 0.787559 | 0.00575 | 0.027666 |
| Rpsa | chr9:120127765-120132369 | 368.781 | 290.443 | 0.787576 | 5.00E-05 | 0.000607 |
| Elmod3 | chr6:72565921-72598413 | 19.5288 | 15.3848 | 0.787801 | 0.00055 | 0.004491 |
| Urb1 | chr16:90751526-90810413 | 3.82707 | 3.01504 | 0.787819 | 0.0038 | 0.020026 |
| Aarsd1 | chr11:101406839-101417433 | 57.8903 | 45.6076 | 0.787828 | 5.00E-05 | 0.000607 |
| Atp5j2 | chr5:145183705-145191592 | 528.848 | 416.724 | 0.787984 | 5.00E-05 | 0.000607 |
| Bnip1 | chr17:26781078-26792521 | 27.3547 | 21.5564 | 0.788033 | 0.0041 | 0.021197 |
| Atraid | chr5:31036035-31054623 | 87.0113 | 68.5717 | 0.788078 | 0.00015 | 0.001558 |
| Csf1 | chr3:107741047-107760469 | 10.9463 | 8.62663 | 0.788086 | 0.00095 | 0.006869 |
| Nbl1 | chr4:139082291-139092970 | 49.9911 | 39.3991 | 0.788122 | 5.00E-05 | 0.000607 |
| Mrps26 | chr2:130563756-130565394 | 71.5737 | 56.4212 | 0.788295 | 0.00015 | 0.001558 |
| Pdgfrb | chr18:61045149-61085067 | 7.11836 | 5.61242 | 0.788443 | 0.0008 | 0.006001 |
| Cgref1 | chr5:30933142-30945480 | 18.2395 | 14.3838 | 0.788607 | 0.01055 | 0.044068 |
| Vrk3 | chr7:44748628-44777514 | 19.5794 | 15.4406 | 0.788615 | 0.0019 | 0.01161 |
| Tpt1 | chr14:75845255-75848303 | 925.814 | 730.131 | 0.788637 | 5.00E-05 | 0.000607 |
| Olfr856-ps1 | chr9:19657038-19658680 | 26.4559 | 20.8689 | 0.788818 | 0.00075 | 0.005715 |
| Fdx1l | chr9:21067513-21092008 | 76.1704 | 60.1184 | 0.789262 | 0.0067 | 0.031027 |
| Srp14 | chr2:118475842-118479696 | 203.768 | 160.826 | 0.78926 | 5.00E-05 | 0.000607 |
| Mrpl15 | chr1:4773199-4785726 | 8.23776 | 6.50244 | 0.789346 | 0.00565 | 0.027333 |
| Nedd8 | chr14:55662266-55671906 | 258.647 | 204.164 | 0.789354 | 5.00E-05 | 0.000607 |
| Acbd6 | chr1:155558119-155687233 | 31.1491 | 24.5967 | 0.789644 | 0.00135 | 0.008911 |
| Ubxn1 | chr19:8871558-8875656 | 104.831 | 82.7844 | 0.789694 | 5.00E-05 | 0.000607 |
| Mtmr14 | chr6:113237842-113281392 | 8.51601 | 6.72514 | 0.789706 | 0.00715 | 0.032557 |
| Zmat2 | chr18:36793922-36799660 | 130.374 | 102.961 | 0.789736 | 5.00E-05 | 0.000607 |
| Tab1 | chr15:80133153-80161702 | 15.3656 | 12.1369 | 0.789875 | 0.00035 | 0.003088 |
| Mt3 | chr8:94152606-94154148 | 1072.48 | 847.179 | 0.789925 | 5.00E-05 | 0.000607 |
| Snx22 | chr9:66060168-66069731 | 38.4324 | 30.3614 | 0.789995 | 0.00055 | 0.004491 |
| Rgl2 | chr17:33929893-33937687 | 41.479 | 32.7687 | 0.790007 | 5.00E-05 | 0.000607 |
| 1810022K09Rik | chr3:14578670-14611256 | 110.878 | 87.6048 | 0.790101 | 0.00825 | 0.036455 |
| Ccz1 | chr5:143987908-144014853 | 23.6678 | 18.7032 | 0.790238 | 0.00075 | 0.005715 |
| Mrpl46 | chr7:78775340-78783089 | 29.0402 | 22.9488 | 0.790242 | 0.00595 | 0.028422 |
| Cox6b1 | chr7:30616973-30626151 | 822.111 | 649.668 | 0.790244 | 5.00E-05 | 0.000607 |
| Pop5 | chr5:115235850-115240970 | 75.3138 | 59.519 | 0.79028 | 5.00E-05 | 0.000607 |
| Ndufaf1 | chr2:119655450-119662798 | 17.9028 | 14.1527 | 0.79053 | 0.0054 | 0.026352 |
| Mas1 | chr17:12682405-12868143 | 28.4882 | 22.5281 | 0.790787 | 0.0033 | 0.017971 |
| Tle1 | chr4:72117141-72200919 | 13.4675 | 10.6507 | 0.790845 | 0.0001 | 0.001096 |
| Pcgf1 | chr6:83078389-83080855 | 26.493 | 20.9559 | 0.790998 | 0.0109 | 0.045122 |
| Limd2 | chr11:106156255-106160142 | 50.415 | 39.8863 | 0.791159 | 5.00E-05 | 0.000607 |
| Gstm7 | chr3:107926333-107931745 | 32.4292 | 25.6584 | 0.791213 | 0.00105 | 0.007395 |
| Msrb1 | chr17:24736641-24742778 | 31.4089 | 24.8535 | 0.791288 | 0.0072 | 0.032757 |
| Ssr4 | chrX:73787027-73790828 | 70.6206 | 55.8852 | 0.791344 | 0.00095 | 0.006869 |
| Cyth4 | chr15:78597046-78622019 | 10.9741 | 8.6862 | 0.791518 | 0.0028 | 0.0158 |
| Mcm6 | chr1:128331590-128359656 | 7.42875 | 5.88156 | 0.791729 | 0.01065 | 0.044364 |
| Fau | chr19:6057887-6059524 | 561.03 | 444.234 | 0.791819 | 5.00E-05 | 0.000607 |
| Myl6 | chr10:128490860-128493825 | 759.971 | 601.937 | 0.792053 | 5.00E-05 | 0.000607 |
| Fkbp1b | chr12:4833173-4841595 | 43.1416 | 34.177 | 0.792205 | 0.00115 | 0.007885 |
| Cep63 | chr9:102586577-102626124 | 13.5077 | 10.7023 | 0.792311 | 0.00125 | 0.008413 |
| Fam57b | chr7:126816884-126830219 | 52.8509 | 41.8767 | 0.792355 | 5.00E-05 | 0.000607 |
| Aamdc | chr7:97550330-97579497 | 52.5875 | 41.6706 | 0.792405 | 0.00115 | 0.007885 |
| Cbs | chr17:31612622-31637205 | 20.5485 | 16.2868 | 0.792603 | 0.0004 | 0.00346 |
| Cc2d1b | chr4:108619955-108634122 | 16.958 | 13.4432 | 0.792735 | 0.00015 | 0.001558 |
| Ccnl1 | chr3:65946150-65958225 | 31.3601 | 24.862 | 0.792791 | 0.0001 | 0.001096 |
| Nsmce4a | chr7:130532525-130547381 | 33.2354 | 26.3542 | 0.792956 | 0.0004 | 0.00346 |
| Atp5l | chr9:44913247-44920742 | 676.808 | 536.707 | 0.792997 | 5.00E-05 | 0.000607 |
| Bax | chr7:45461694-45466898 | 70.5902 | 55.9824 | 0.793062 | 0.0003 | 0.002726 |
| Tarbp2 | chr15:102518191-102523676 | 18.4013 | 14.5956 | 0.793183 | 0.00265 | 0.01513 |
| Tdrd3 | chr14:87416582-87545508 | 8.73676 | 6.92998 | 0.793198 | 0.01005 | 0.042515 |
| Pisd-ps2 | chr17:3064317-3084183 | 41.5069 | 32.9381 | 0.793557 | 0.00025 | 0.002352 |
| Cpsf4 | chr5:145167223-145182040 | 13.9811 | 11.0988 | 0.793843 | 0.01135 | 0.046603 |
| Slc30a3 | chr5:31086105-31093527 | 64.3722 | 51.1039 | 0.793882 | 5.00E-05 | 0.000607 |
| Dvl2 | chr11:70000625-70010109 | 10.0086 | 7.94771 | 0.794088 | 0.00385 | 0.02021 |
| Mrpl40 | chr16:18872017-18876637 | 31.8282 | 25.28 | 0.794264 | 0.00375 | 0.019852 |
| Them6 | chr15:74721233-74724373 | 28.0311 | 22.2736 | 0.794603 | 0.00155 | 0.009917 |
| Rpl17 | chr18:75000476-75003381 | 668.916 | 531.553 | 0.794648 | 5.00E-05 | 0.000607 |
| Zfp740 | chr15:102208266-102215601 | 33.0834 | 26.2918 | 0.794713 | 5.00E-05 | 0.000607 |
| Rps29 | chr12:69157721-69159186 | 1746.98 | 1388.4 | 0.794743 | 5.00E-05 | 0.000607 |
| Hsd17b10 | chrX:152001895-152004442 | 45.1688 | 35.9033 | 0.794869 | 0.0018 | 0.011147 |
| Zfp692 | chr11:58307068-58314613 | 36.3048 | 28.8642 | 0.795052 | 5.00E-05 | 0.000607 |
| Rps13 | chr7:116331506-116334190 | 383.358 | 304.846 | 0.795199 | 5.00E-05 | 0.000607 |
| Rps11 | chr7:45122387-45124389 | 509.097 | 404.979 | 0.795485 | 5.00E-05 | 0.000607 |
| Itpka | chr2:119742336-119751253 | 165.45 | 131.623 | 0.795545 | 5.00E-05 | 0.000607 |
| Psph | chr5:129765557-129787253 | 16.4482 | 13.0858 | 0.795576 | 0.00805 | 0.035765 |
| Rpl8 | chr15:76904070-76906318 | 509.794 | 405.708 | 0.795827 | 5.00E-05 | 0.000607 |
| Cdk5rap3 | chr11:96907785-96916481 | 24.1651 | 19.2336 | 0.795925 | 0.00095 | 0.006869 |
| Slc9a8 | chr2:167421720-167476998 | 14.5735 | 11.6012 | 0.796048 | 5.00E-05 | 0.000607 |
| Pebp1 | chr5:117282650-117287564 | 608.943 | 484.884 | 0.796272 | 5.00E-05 | 0.000607 |
| Znf512b | chr2:181569152-181592461 | 38.5271 | 30.682 | 0.796375 | 0.00015 | 0.001558 |
| Tor1b | chr2:30953000-30959015 | 26.3366 | 20.9752 | 0.796428 | 5.00E-05 | 0.000607 |
| Smpd2 | chr10:41487171-41490340 | 15.6015 | 12.4417 | 0.797468 | 0.0078 | 0.034859 |
| Rplp2 | chr7:141447649-141451342 | 440.649 | 351.476 | 0.797633 | 5.00E-05 | 0.000607 |
| Adprh | chr16:38445398-38452689 | 71.5054 | 57.0376 | 0.797668 | 5.00E-05 | 0.000607 |
| Nme2 | chr11:93949814-93956007 | 212.36 | 169.403 | 0.797716 | 5.00E-05 | 0.000607 |
| Penk | chr4:4133535-4138445 | 26.1717 | 20.8861 | 0.798041 | 0.00275 | 0.015584 |
| Evc | chr5:37299170-37336881 | 5.14877 | 4.10896 | 0.798047 | 0.01 | 0.042388 |
| Uqcrh | chr4:116066964-116075070 | 653.84 | 521.841 | 0.798117 | 5.00E-05 | 0.000607 |
| 2610017I09Rik | chr1:42648199-42694825 | 106.629 | 85.1051 | 0.798142 | 0.0008 | 0.006001 |
| BC029214 | chr2:25459487-25461094 | 35.2733 | 28.1592 | 0.798315 | 0.00425 | 0.021761 |
| Stoml2 | chr4:43027689-43031384 | 42.0066 | 33.5346 | 0.798317 | 0.0003 | 0.002726 |
| Cwc27 | chr13:104631326-104816953 | 13.3886 | 10.69 | 0.79844 | 0.0092 | 0.039743 |
| Rpl11 | chr4:136049947-136053371 | 650.908 | 519.825 | 0.798615 | 5.00E-05 | 0.000607 |
| Orai2 | chr5:136147460-136170656 | 64.5935 | 51.5864 | 0.798631 | 5.00E-05 | 0.000607 |
| Dnajc30 | chr5:135052956-135065365 | 34.4562 | 27.5252 | 0.798846 | 0.00935 | 0.04026 |
| Psmd4 | chr3:95032704-95042545 | 120.389 | 96.191 | 0.799002 | 5.00E-05 | 0.000607 |
| BC056474 | chr8:85080962-85082339 | 84.9396 | 67.8773 | 0.799124 | 0.001 | 0.007133 |
| Rps6kb2 | chr19:4156976-4163245 | 40.2369 | 32.1547 | 0.799135 | 0.0003 | 0.002726 |
| Fbxl6 | chr15:76535727-76538746 | 29.1535 | 23.2976 | 0.799136 | 0.00065 | 0.005126 |
| Psmb4 | chr3:94884323-94886958 | 211.828 | 169.298 | 0.799224 | 5.00E-05 | 0.000607 |
| Rpl23a | chr11:78180935-78183584 | 882.071 | 704.99 | 0.799244 | 5.00E-05 | 0.000607 |
| Rpl13 | chr8:123102349-123105242 | 520.896 | 416.386 | 0.799365 | 5.00E-05 | 0.000607 |
| Sod1 | chr16:90220741-90226324 | 486.103 | 388.576 | 0.79937 | 5.00E-05 | 0.000607 |
| Abhd11 | chr5:135009151-135013157 | 31.2756 | 25.0035 | 0.799457 | 0.0067 | 0.031027 |
| Mnat1 | chr12:73123716-73273988 | 8.90665 | 7.12114 | 0.799531 | 0.01155 | 0.047187 |
| Smarcd3 | chr5:24592621-24602002 | 52.1024 | 41.6648 | 0.799671 | 0.0001 | 0.001096 |
| Uqcrb | chr13:66900620-66905350 | 325.557 | 260.369 | 0.799765 | 5.00E-05 | 0.000607 |
| Serf2 | chr2:121449227-121456764 | 54.3067 | 43.4344 | 0.799798 | 5.00E-05 | 0.000607 |
| Rab24 | chr13:55319222-55321980 | 44.2977 | 35.4295 | 0.799805 | 5.00E-05 | 0.000607 |
| Ost4 | chr5:30888851-30907788 | 98.3025 | 78.6366 | 0.799945 | 0.01195 | 0.048485 |
| Adck4 | chr7:27233012-27257949 | 18.3958 | 14.7167 | 0.800003 | 0.002 | 0.012075 |
| Mrps18c | chr5:100798758-100804467 | 75.7345 | 60.5985 | 0.800144 | 0.0078 | 0.034859 |
| Rbm3 | chrX:8138974-8147963 | 100.757 | 80.6608 | 0.800548 | 5.00E-05 | 0.000607 |
| Bloc1s1 | chr10:128919913-128923524 | 136.812 | 109.537 | 0.800639 | 0.00045 | 0.003815 |
| Bag1 | chr4:40936397-40948294 | 109.094 | 87.3509 | 0.800694 | 5.00E-05 | 0.000607 |
| Atp6v0b | chr4:117884329-117887329 | 287.939 | 230.556 | 0.800711 | 5.00E-05 | 0.000607 |
| Hagh | chr17:24850489-24864450 | 104.077 | 83.3495 | 0.800845 | 5.00E-05 | 0.000607 |
| Slc7a6os | chr8:106200437-106210933 | 17.4772 | 13.9975 | 0.800901 | 0.00855 | 0.037593 |
| Ssna1 | chr2:25271038-25272418 | 59.6447 | 47.7702 | 0.800913 | 0.00105 | 0.007395 |
| Psmb3 | chr11:97703433-97713500 | 159.8 | 128.021 | 0.801133 | 0.00015 | 0.001558 |
| Selm | chr11:3514701-3517351 | 127.198 | 101.933 | 0.801373 | 5.00E-05 | 0.000607 |
| Aurkaip1 | chr4:155831268-155833098 | 64.6482 | 51.8118 | 0.801442 | 0.0002 | 0.001971 |
| Oxsr1 | chr9:119238432-119322427 | 19.8365 | 15.8997 | 0.801538 | 0.0001 | 0.001096 |
| Mecr | chr4:131843470-131867787 | 23.1093 | 18.5296 | 0.801824 | 0.00695 | 0.031865 |
| Smyd5 | chr6:85431975-85446429 | 29.5526 | 23.6962 | 0.801831 | 0.0001 | 0.001096 |
| Anapc16 | chr10:59987908-60099990 | 56.8905 | 45.637 | 0.80219 | 0.0007 | 0.005435 |
| Invs | chr4:48279801-48473422 | 6.59231 | 5.28891 | 0.802285 | 0.01115 | 0.045835 |
| Cep70 | chr9:99243467-99300403 | 10.6179 | 8.5196 | 0.802381 | 0.0075 | 0.033932 |
| Praf2 | chrX:7728570-7731063 | 99.151 | 79.5889 | 0.802704 | 5.00E-05 | 0.000607 |
| Dgka | chr10:128706257-128744056 | 11.3215 | 9.08911 | 0.802819 | 0.00415 | 0.021362 |
| Ufc1 | chr1:171288563-171294982 | 99.3544 | 79.7713 | 0.802896 | 5.00E-05 | 0.000607 |
| Tmbim4 | chr10:120208825-120224897 | 47.0686 | 37.7944 | 0.802964 | 0.00575 | 0.027666 |
| Ybx1 | chr4:119277326-119294513 | 85 | 68.2609 | 0.803069 | 5.00E-05 | 0.000607 |
| Mcm3ap | chr10:76449080-76515857 | 16.6384 | 13.3627 | 0.803124 | 0.0001 | 0.001096 |
| Ddrgk1 | chr2:130654082-130664645 | 70.9246 | 56.9653 | 0.803181 | 0.0001 | 0.001096 |
| Lingo3 | chr10:80832800-80844039 | 12.2203 | 9.81762 | 0.803386 | 0.00085 | 0.0063 |
| Polr2h | chr16:20717825-20722265 | 41.3428 | 33.2154 | 0.803414 | 0.00665 | 0.030876 |
| Zkscan6 | chr11:65807243-65829239 | 14.9323 | 11.9977 | 0.803473 | 0.004 | 0.020812 |
| Snapc4 | chr2:26362764-26380653 | 29.0982 | 23.3984 | 0.804118 | 5.00E-05 | 0.000607 |
| 9430023L20Rik | chr15:101284300-101290934 | 41.4819 | 33.357 | 0.804134 | 0.00125 | 0.008413 |
| Tmem242 | chr17:5410863-5440260 | 59.2719 | 47.6627 | 0.804137 | 0.00055 | 0.004491 |
| Dbi | chr1:120113279-120121096 | 489.954 | 394.005 | 0.804167 | 5.00E-05 | 0.000607 |
| Rpl10 | chrX:74270815-74273135 | 621.933 | 500.277 | 0.804391 | 0.0001 | 0.001096 |
| Anp32b | chr4:46451116-46472523 | 75.281 | 60.5575 | 0.804419 | 0.0001 | 0.001096 |
| Mrpl20 | chr4:155803617-155808829 | 131.753 | 105.986 | 0.80443 | 0.0001 | 0.001096 |
| Trnp1 | chr4:133491099-133498550 | 106.714 | 85.8446 | 0.804436 | 5.00E-05 | 0.000607 |
| Pan2 | chr10:128303334-128321358 | 13.1659 | 10.5922 | 0.804518 | 0.0006 | 0.004799 |
| Helq | chr5:100762147-100798600 | 6.26432 | 5.04011 | 0.804574 | 0.0116 | 0.047355 |
| Lamtor2 | chr3:88549818-88552927 | 100.8 | 81.1228 | 0.80479 | 0.00155 | 0.009917 |
| Stub1 | chr17:25829042-25833361 | 115.271 | 92.8014 | 0.805072 | 0.0001 | 0.001096 |
| Clpp | chr17:56990263-56996371 | 58.2446 | 46.8916 | 0.805081 | 0.00025 | 0.002352 |
| Tmem50a | chr4:134897848-134914916 | 103.481 | 83.3374 | 0.80534 | 5.00E-05 | 0.000607 |
| Srrt | chr5:137295703-137307674 | 75.9112 | 61.139 | 0.805402 | 5.00E-05 | 0.000607 |
| D8Ertd738e | chr8:84246234-84249761 | 141.528 | 113.989 | 0.805417 | 0.0001 | 0.001096 |
| Rab26 | chr17:24529053-24533747 | 49.961 | 40.2412 | 0.805452 | 0.00015 | 0.001558 |
| Snrpg | chr6:86371539-86378902 | 124.066 | 99.939 | 0.805531 | 0.0055 | 0.026705 |
| Dgat2 | chr7:99153662-99182713 | 61.8822 | 49.8493 | 0.805552 | 5.00E-05 | 0.000607 |
| Ndufv2 | chr17:66078794-66101491 | 121.218 | 97.6525 | 0.805594 | 5.00E-05 | 0.000607 |
| Parp2 | chr14:50807945-50821300 | 17.0418 | 13.7294 | 0.805631 | 0.00665 | 0.030876 |
| Ppan | chr9:20888174-20892179 | 16.5625 | 13.3469 | 0.805851 | 0.00885 | 0.038577 |
| Txndc17 | chr11:72207553-72210487 | 27.5656 | 22.2156 | 0.805918 | 0.00245 | 0.014171 |
| Hook2 | chr8:84990594-85003364 | 10.1097 | 8.14993 | 0.80615 | 0.00895 | 0.038901 |
| Clasrp | chr7:19581042-19604468 | 43.6704 | 35.2099 | 0.806265 | 5.00E-05 | 0.000607 |
| Mrpl17 | chr7:105803781-105811087 | 6.3766 | 5.14134 | 0.806282 | 0.00155 | 0.009917 |
| Slc35b1 | chr11:95384921-95391652 | 52.1476 | 42.0463 | 0.806294 | 0.00075 | 0.005715 |
| Cox14 | chr15:99725617-99728136 | 95.6849 | 77.1884 | 0.806694 | 0.00035 | 0.003088 |
| 2700060E02Rik | chr14:19751256-19823823 | 70.8451 | 57.1729 | 0.807013 | 0.00015 | 0.001558 |
| Gstz1 | chr12:87106865-87164723 | 36.1206 | 29.1568 | 0.807207 | 0.00495 | 0.02454 |
| Mpnd | chr17:56009200-56036637 | 67.013 | 54.0947 | 0.807227 | 0.00365 | 0.01938 |
| Atp5j | chr16:84827870-84863778 | 310.03 | 250.277 | 0.807267 | 5.00E-05 | 0.000607 |
| Fra10ac1 | chr19:38188478-38224132 | 19.3437 | 15.6165 | 0.807317 | 0.01225 | 0.049532 |
| Bzrap1 | chr11:87760540-87785928 | 85.3153 | 68.8828 | 0.807391 | 0.0002 | 0.001971 |
| Dgcr6 | chr16:18052859-18071342 | 55.4846 | 44.8017 | 0.807462 | 0.0005 | 0.004159 |
| Ppia | chr11:6415869-6419810 | 2366.19 | 1910.64 | 0.807475 | 0.0033 | 0.017971 |
| Jtb | chr3:90231596-90235840 | 39.9689 | 32.2781 | 0.80758 | 0.0031 | 0.017146 |
| Arpc3 | chr5:122391927-122406178 | 303.959 | 245.644 | 0.808148 | 5.00E-05 | 0.000607 |
| Itga8 | chr2:12106659-12312315 | 7.79242 | 6.29786 | 0.808203 | 0.00225 | 0.013268 |
| Tmem14c | chr13:41016249-41022582 | 58.9205 | 47.6312 | 0.808398 | 0.00075 | 0.005715 |
| Ndufb10 | chr17:24722066-24724388 | 303.669 | 245.534 | 0.808558 | 5.00E-05 | 0.000607 |
| Smim12 | chr4:127243783-127247809 | 49.6507 | 40.1532 | 0.808714 | 0.00175 | 0.010881 |
| Nubp2 | chr17:24882610-24886350 | 23.6921 | 19.1611 | 0.808755 | 0.00415 | 0.021362 |
| Eef1d | chr15:75894799-75909340 | 53.2257 | 43.0477 | 0.808777 | 0.0003 | 0.002726 |
| Ephx1 | chr1:180989555-181017495 | 22.3748 | 18.1079 | 0.809299 | 0.0035 | 0.018791 |
| Denr | chr5:123907274-123928832 | 37.3449 | 30.2241 | 0.809323 | 0.0001 | 0.001096 |
| Rpl4 | chr9:64173386-64178562 | 558.645 | 452.139 | 0.809349 | 0.0001 | 0.001096 |
| Trappc2l | chr8:122611625-122615591 | 99.218 | 80.307 | 0.8094 | 0.0014 | 0.009144 |
| Arl3 | chr19:46531108-46573085 | 152.955 | 123.82 | 0.809519 | 5.00E-05 | 0.000607 |
| Krba1 | chr6:48395585-48419855 | 14.488 | 11.7295 | 0.809601 | 5.00E-05 | 0.000607 |
| Ndufa2 | chr18:36735069-36744548 | 213.924 | 173.197 | 0.809619 | 0.0005 | 0.004159 |
| Coq2 | chr5:100654725-100674256 | 63.4986 | 51.4229 | 0.809827 | 5.00E-05 | 0.000607 |
| Rps5 | chr7:12922310-12926686 | 407.89 | 330.379 | 0.809971 | 5.00E-05 | 0.000607 |
| Ptpmt1 | chr2:90910712-90918050 | 27.4387 | 22.2252 | 0.809995 | 0.0049 | 0.02436 |
| Cetn3 | chr13:81783291-81797157 | 56.2211 | 45.5409 | 0.810032 | 0.0011 | 0.007621 |
| Ddx55 | chr5:124552863-124569660 | 16.1198 | 13.0581 | 0.810066 | 0.00165 | 0.010418 |
| Rnf5 | chr17:34601098-34603561 | 76.5104 | 61.9831 | 0.810126 | 0.00025 | 0.002352 |
| Nol9 | chr4:152039326-152061494 | 11.0926 | 8.98742 | 0.810218 | 0.0024 | 0.013943 |
| Tbcb | chr7:30224130-30232029 | 81.8839 | 66.3507 | 0.810302 | 0.00015 | 0.001558 |
| Lrrc45 | chr11:120713952-120721127 | 48.4397 | 39.2598 | 0.810488 | 5.00E-05 | 0.000607 |
| Cox7a2l | chr17:83501916-83514333 | 237.439 | 192.442 | 0.81049 | 5.00E-05 | 0.000607 |
| Orc6 | chr8:85299631-85308279 | 20.388 | 16.5324 | 0.810889 | 0.00765 | 0.034391 |
| 4930506M07Rik | chr19:58973357-59076069 | 16.407 | 13.3042 | 0.810886 | 0.00035 | 0.003088 |
| Nt5m | chr11:59848072-59876533 | 48.9312 | 39.6787 | 0.810908 | 0.00055 | 0.004491 |
| Polr2g | chr19:8793128-8798557 | 122.484 | 99.3413 | 0.811055 | 5.00E-05 | 0.000607 |
| Trappc4 | chr9:44403758-44407548 | 49.4989 | 40.1642 | 0.811416 | 0.0008 | 0.006001 |
| Aprt | chr8:122574636-122576907 | 51.2941 | 41.6308 | 0.81161 | 0.00335 | 0.018178 |
| Nenf | chr1:191306796-191318118 | 116.716 | 94.7294 | 0.811623 | 0.0003 | 0.002726 |
| Cryab | chr9:50752757-50756633 | 102.123 | 82.8936 | 0.811704 | 5.00E-05 | 0.000607 |
| Kptn | chr7:16119875-16127516 | 16.445 | 13.3504 | 0.811821 | 0.0117 | 0.047671 |
| Fam131a | chr16:20695056-20716636 | 315.201 | 255.979 | 0.812114 | 0.0018 | 0.011147 |
| Fam89b | chr19:5728086-5729666 | 24.3201 | 19.7524 | 0.812184 | 0.01055 | 0.044068 |
| Nrn1 | chr13:36725624-36734454 | 174.778 | 141.972 | 0.812299 | 0.00015 | 0.001558 |
| Vimp | chr7:66079648-66089405 | 47.3371 | 38.457 | 0.812407 | 0.00135 | 0.008911 |
| Hmgn3 | chr9:83109941-83146607 | 81.0397 | 65.8458 | 0.812513 | 0.0003 | 0.002726 |
| Mrps33 | chr6:39801806-39810936 | 96.9106 | 78.749 | 0.812594 | 0.00025 | 0.002352 |
| Rras2 | chr7:114046781-114117781 | 13.6344 | 11.0847 | 0.812995 | 0.0085 | 0.037389 |
| Prr24 | chr7:16272012-16273692 | 22.1198 | 17.9849 | 0.813068 | 0.0054 | 0.026352 |
| Grik4 | chr9:42520411-42944371 | 18.2233 | 14.8179 | 0.813129 | 0.00055 | 0.004491 |
| Spcs1 | chr14:30999825-31019089 | 107.139 | 87.1376 | 0.813314 | 0.0042 | 0.021546 |
| Lamtor5 | chr3:107278857-107284081 | 76.4627 | 62.189 | 0.813325 | 0.0005 | 0.004159 |
| Faf1 | chr4:109676626-109963960 | 11.3342 | 9.2189 | 0.81337 | 0.00105 | 0.007395 |
| Lsm4 | chr8:70673230-70678752 | 35.0356 | 28.4991 | 0.813433 | 0.0093 | 0.040094 |
| Ubc | chr5:125385964-125390017 | 108.419 | 88.2219 | 0.813713 | 0.0001 | 0.001096 |
| Timm44 | chr8:4259730-4275905 | 46.939 | 38.1957 | 0.813731 | 0.0003 | 0.002726 |
| Dclre1c | chr2:3424130-3474986 | 10.4554 | 8.50936 | 0.813872 | 0.0042 | 0.021546 |
| Nsa2 | chr13:97067286-97137926 | 34.0558 | 27.7221 | 0.81402 | 0.00325 | 0.017763 |
| E130012A19Rik | chr11:97627386-97629716 | 12.3348 | 10.0432 | 0.814217 | 0.011 | 0.045394 |
| Ngfrap1 | chrX:136270252-136271978 | 313.644 | 255.376 | 0.814222 | 5.00E-05 | 0.000607 |
| Npdc1 | chr2:25403082-25409494 | 204.433 | 166.473 | 0.814316 | 5.00E-05 | 0.000607 |
| Mt2 | chr8:94172617-94173567 | 501.79 | 408.625 | 0.814335 | 0.0001 | 0.001096 |
| Tesc | chr5:118027823-118061870 | 63.6869 | 51.8692 | 0.814441 | 0.00135 | 0.008911 |
| Gabarap | chr11:69991369-69994949 | 192.091 | 156.477 | 0.814598 | 5.00E-05 | 0.000607 |
| Lamp5 | chr2:136057926-136069917 | 30.9038 | 25.1782 | 0.814728 | 0.00075 | 0.005715 |
| Atp5o | chr16:91925222-91931630 | 438.567 | 357.42 | 0.814972 | 5.00E-05 | 0.000607 |
| Col4a2 | chr8:11312828-11449287 | 34.0673 | 27.7666 | 0.815051 | 0.0001 | 0.001096 |
| Tmem199 | chr11:78507054-78512168 | 42.1602 | 34.3686 | 0.815191 | 0.0011 | 0.007621 |
| Swi5 | chr2:32278815-32288068 | 238.729 | 194.618 | 0.815226 | 0.0002 | 0.001971 |
| Chkb | chr15:89416404-89429927 | 26.6162 | 21.7002 | 0.8153 | 0.00375 | 0.019852 |
| Gnl2 | chr4:125030013-125065657 | 40.3411 | 32.8923 | 0.815355 | 0.00035 | 0.003088 |
| Apoo | chrX:94367109-94417092 | 59.2255 | 48.2937 | 0.815421 | 0.00165 | 0.010418 |
| Dnajc8 | chr4:132535558-132553742 | 90.1929 | 73.5612 | 0.815599 | 5.00E-05 | 0.000607 |
| Dynlrb1 | chr2:155236532-155250277 | 326.189 | 266.051 | 0.815634 | 5.00E-05 | 0.000607 |
| Ptms | chr6:124913674-124917946 | 671.554 | 547.767 | 0.815671 | 0.00035 | 0.003088 |
| Edc4 | chr8:105880950-105893207 | 28.2112 | 23.0177 | 0.815906 | 0.00015 | 0.001558 |
| Ict1 | chr11:115403765-115410913 | 52.4606 | 42.8078 | 0.815999 | 0.00825 | 0.036455 |
| Ahi1 | chr10:20952546-21080429 | 126.772 | 103.453 | 0.816056 | 0.001 | 0.007133 |
| Dut | chr2:125247247-125259049 | 14.299 | 11.6724 | 0.816309 | 0.00785 | 0.035053 |
| Cnih3 | chr1:181352627-181460641 | 44.3779 | 36.2389 | 0.816598 | 5.00E-05 | 0.000607 |
| Stx5a | chr19:8741423-8755642 | 50.6116 | 41.3338 | 0.816686 | 0.00025 | 0.002352 |
| Tomm6 | chr17:47686644-47691092 | 82.9835 | 67.7738 | 0.816714 | 0.0015 | 0.009667 |
| Etfb | chr7:43444071-43457800 | 65.1952 | 53.2487 | 0.816758 | 0.00185 | 0.011363 |
| Timm9 | chr12:71111427-71136675 | 59.2434 | 48.3889 | 0.816781 | 0.00335 | 0.018178 |
| Pld2 | chr11:70540163-70558110 | 9.15291 | 7.47987 | 0.817212 | 0.0071 | 0.032371 |
| Kif1c | chr11:70700547-70731970 | 29.646 | 24.2307 | 0.817335 | 0.0001 | 0.001096 |
| A730017C20Rik | chr18:59062380-59076960 | 35.7001 | 29.1792 | 0.817342 | 0.00065 | 0.005126 |
| Zfp410 | chr12:84316858-84344439 | 11.8334 | 9.67197 | 0.817345 | 0.00735 | 0.033325 |
| Rps3 | chr7:99477896-99483709 | 216.819 | 177.215 | 0.817341 | 0.0002 | 0.001971 |
| Rab40b | chr11:121356120-121388251 | 27.8214 | 22.742 | 0.817428 | 0.00215 | 0.012828 |
| Atp5g3 | chr2:73908449-73911294 | 559.483 | 457.357 | 0.817464 | 0.0001 | 0.001096 |
| Cops5 | chr1:10024830-10037898 | 72.5181 | 59.2987 | 0.817709 | 0.00015 | 0.001558 |
| C1ql2 | chr1:120340581-120343174 | 39.7534 | 32.5143 | 0.8179 | 0.0005 | 0.004159 |
| Dennd6b | chr15:89182212-89196474 | 34.3433 | 28.0906 | 0.817935 | 5.00E-05 | 0.000607 |
| Cotl1 | chr8:119809213-119840579 | 68.7444 | 56.232 | 0.817987 | 5.00E-05 | 0.000607 |
| Arl2 | chr19:6134388-6141137 | 72.1522 | 59.0203 | 0.817997 | 0.0014 | 0.009144 |
| Mrps34 | chr17:24895119-24896273 | 55.5431 | 45.4383 | 0.818073 | 0.0031 | 0.017146 |
| Banf1 | chr19:5364639-5366645 | 94.0083 | 76.9101 | 0.81812 | 0.00105 | 0.007395 |
| Stoml1 | chr9:58253163-58262524 | 48.5783 | 39.7441 | 0.818145 | 0.0002 | 0.001971 |
| Bex2 | chrX:136066564-136068236 | 452.912 | 370.636 | 0.81834 | 0.00025 | 0.002352 |
| Lrrfip2 | chr9:111118110-111225668 | 14.3947 | 11.7799 | 0.81835 | 0.00175 | 0.010881 |
| Parl | chr16:20279820-20302362 | 26.2061 | 21.4487 | 0.818462 | 0.00795 | 0.03541 |
| Zfp956 | chr6:47943174-47965299 | 18.5827 | 15.211 | 0.818557 | 0.0041 | 0.021197 |
| Atpif1 | chr4:132530554-132533659 | 512.616 | 419.607 | 0.81856 | 0.0001 | 0.001096 |
| Suclg1 | chr6:73248504-73276907 | 109.998 | 90.0462 | 0.818617 | 0.0002 | 0.001971 |
| Rpl7a | chr2:26910806-26913311 | 532.223 | 435.706 | 0.818653 | 0.00015 | 0.001558 |
| Snrpb2 | chr2:143063068-143072052 | 26.4431 | 21.6509 | 0.818773 | 0.01175 | 0.047838 |
| Commd3 | chr2:18672461-18676216 | 89.3594 | 73.1689 | 0.818816 | 0.001 | 0.007133 |
| Sri | chr5:8046079-8069245 | 31.0073 | 25.3926 | 0.818923 | 0.0006 | 0.004799 |
| Bckdhb | chr9:83948780-84124240 | 31.9954 | 26.2057 | 0.819046 | 0.0034 | 0.018393 |
| Prpf40b | chr15:99295408-99317007 | 47.5104 | 38.9273 | 0.819343 | 0.0001 | 0.001096 |
| Glrx5 | chr12:105032688-105040910 | 49.5947 | 40.6379 | 0.8194 | 0.00475 | 0.023714 |
| Lamtor1 | chr7:101899807-101911903 | 86.4405 | 70.842 | 0.819546 | 0.0003 | 0.002726 |
| Rps2 | chr17:24720062-24721927 | 540.678 | 443.132 | 0.819586 | 0.0002 | 0.001971 |
| Psma7 | chr2:180036375-180042402 | 141.518 | 115.993 | 0.819634 | 5.00E-05 | 0.000607 |
| Map7 | chr10:20148919-20281590 | 38.2217 | 31.329 | 0.819665 | 5.00E-05 | 0.000607 |
| Gm996 | chr2:25575415-25580099 | 46.3865 | 38.0243 | 0.819728 | 5.00E-05 | 0.000607 |
| Rpl6 | chr5:121204500-121209241 | 414.817 | 340.062 | 0.819788 | 0.00025 | 0.002352 |
| Vash1 | chr12:86678699-86695681 | 16.1115 | 13.2087 | 0.819831 | 0.00025 | 0.002352 |
| Rpl22 | chr4:152325877-152334071 | 75.029 | 61.5401 | 0.820218 | 5.00E-05 | 0.000607 |
| Sst | chr16:23889580-23890844 | 590.9 | 484.673 | 0.820228 | 5.00E-05 | 0.000607 |
| Grina | chr15:76246806-76249904 | 449.338 | 368.629 | 0.820382 | 0.0004 | 0.00346 |
| Golgb1 | chr16:36880518-36933085 | 22.6609 | 18.5968 | 0.820656 | 5.00E-05 | 0.000607 |
| Rnf167 | chr11:70647588-70651414 | 67.1289 | 55.0913 | 0.820679 | 0.0003 | 0.002726 |
| Snf8 | chr11:96034916-96047405 | 87.1223 | 71.5204 | 0.82092 | 0.0005 | 0.004159 |
| Rpl3 | chr15:80077780-80083406 | 673.743 | 553.14 | 0.820996 | 0.0015 | 0.009667 |
| Ddx26b | chrX:56454838-56507843 | 33.0593 | 27.1543 | 0.821382 | 0.00015 | 0.001558 |
| Dpysl2 | chr14:66802863-66868600 | 56.6299 | 46.519 | 0.821457 | 0.0001 | 0.001096 |
| 2410002F23Rik | chr7:44246721-44252319 | 50.8348 | 41.7699 | 0.821679 | 5.00E-05 | 0.000607 |
| Serp2 | chr14:76532811-76556889 | 115.336 | 94.7784 | 0.821759 | 0.00035 | 0.003088 |
| Cox4i1 | chr8:120668289-120674209 | 1176.39 | 966.807 | 0.821842 | 0.00035 | 0.003088 |
| Sart1 | chr19:5377522-5390069 | 21.2948 | 17.5017 | 0.821877 | 0.0001 | 0.001096 |
| Grin2c | chr11:115249168-115267243 | 14.8514 | 12.2061 | 0.821882 | 0.0006 | 0.004799 |
| Rpl18a | chr8:70894721-70897443 | 262.256 | 215.569 | 0.821979 | 0.0001 | 0.001096 |
| Wdr74 | chr19:8735838-8740624 | 32.7278 | 26.9087 | 0.822197 | 0.00735 | 0.033325 |
| Ppif | chr14:25694169-25701282 | 29.7326 | 24.4468 | 0.822222 | 0.0057 | 0.027512 |
| Dgcr14 | chr16:17900708-17911348 | 14.5194 | 11.9422 | 0.8225 | 0.0043 | 0.021953 |
| Trim11 | chr11:58978115-58991458 | 18.8835 | 15.5344 | 0.822644 | 0.00445 | 0.022502 |
| Mthfr | chr4:148039076-148059562 | 12.5892 | 10.3609 | 0.822999 | 0.0005 | 0.004159 |
| Pop4 | chr7:38262819-38271348 | 28.4788 | 23.4388 | 0.823026 | 0.0114 | 0.046754 |
| Zkscan2 | chr7:123478638-123500449 | 7.05125 | 5.80368 | 0.823071 | 0.01055 | 0.044068 |
| Rnpepl1 | chr1:92910824-92920585 | 18.3914 | 15.1379 | 0.823097 | 0.00165 | 0.010418 |
| Dpm2 | chr2:32570857-32573571 | 79.6024 | 65.5254 | 0.823159 | 0.00145 | 0.009402 |
| Map2k5 | chr9:63163769-63377852 | 20.2549 | 16.6794 | 0.823475 | 0.0035 | 0.018791 |
| Anapc11 | chr11:120598420-120608198 | 15.5374 | 12.7952 | 0.82351 | 0.0018 | 0.011147 |
| Sigmar1 | chr4:41738495-41741313 | 23.404 | 19.2738 | 0.823526 | 0.00815 | 0.036058 |
| Apoa1bp | chr3:88056522-88058495 | 72.4215 | 59.6412 | 0.823529 | 0.00145 | 0.009402 |
| Hras1 | chr7:141189933-141194004 | 75.0499 | 61.8115 | 0.823605 | 0.0001 | 0.001096 |
| Serpina3n | chr12:104406707-104414329 | 34.165 | 28.1391 | 0.823624 | 0.0008 | 0.006001 |
| Vps37b | chr5:124004640-124032260 | 15.2079 | 12.5265 | 0.823684 | 0.00615 | 0.029076 |
| Nme1 | chr11:93958924-93968521 | 186.961 | 154.007 | 0.823739 | 5.00E-05 | 0.000607 |
| Mrpl30 | chr1:37890552-37898333 | 105.354 | 86.7917 | 0.82381 | 0.0014 | 0.009144 |
| Zfp426 | chr9:20468548-20492746 | 11.6129 | 9.56953 | 0.824043 | 0.0052 | 0.025505 |
| 2-Mar | chr1:184813067-184845847 | 29.6386 | 24.4246 | 0.824081 | 0.00225 | 0.013268 |
| Arpc5 | chr1:152766541-152775580 | 96.3023 | 79.3831 | 0.824312 | 0.0001 | 0.001096 |
| Ndufb7 | chr8:83566757-83571623 | 229.749 | 189.407 | 0.824408 | 0.0006 | 0.004799 |
| 4930455F23Rik | chr1:164275597-164287847 | 18.1206 | 14.9421 | 0.824592 | 0.0108 | 0.044795 |
| Gcdh | chr8:84872110-84893921 | 24.745 | 20.4058 | 0.824643 | 0.0041 | 0.021197 |
| Ift20 | chr11:78536435-78541473 | 70.7984 | 58.3862 | 0.824682 | 0.00365 | 0.01938 |
| Faah | chr4:115996655-116017902 | 63.2834 | 52.1898 | 0.8247 | 5.00E-05 | 0.000607 |
| Cep19 | chr16:32099801-32108054 | 47.6522 | 39.3383 | 0.82553 | 0.00035 | 0.003088 |
| Vps16 | chr2:130417683-130444269 | 33.1463 | 27.3652 | 0.825588 | 0.00135 | 0.008911 |
| Atg16l1 | chr1:87756010-87792428 | 28.8747 | 23.8426 | 0.825726 | 0.0003 | 0.002726 |
| Tstd2 | chr4:46039221-46138475 | 13.9507 | 11.5198 | 0.825751 | 0.0078 | 0.034859 |
| Ndufb3 | chr1:58586598-58595948 | 201.663 | 166.567 | 0.825967 | 0.0007 | 0.005435 |
| U2af1l4 | chr7:30563339-30565364 | 54.5716 | 45.0756 | 0.82599 | 0.00645 | 0.030172 |
| Car4 | chr11:84957753-84966054 | 56.6236 | 46.7887 | 0.826311 | 0.0011 | 0.007621 |
| Slc9a5 | chr8:105348257-105369881 | 7.40506 | 6.11951 | 0.826396 | 0.00615 | 0.029076 |
| Ppt2 | chr17:34616661-34627097 | 32.2921 | 26.6895 | 0.826502 | 0.0023 | 0.013502 |
| Atp5g2 | chr15:102662867-102671047 | 249.735 | 206.427 | 0.826584 | 0.0003 | 0.002726 |
| Lime1 | chr2:181381234-181383628 | 64.3405 | 53.1866 | 0.826643 | 0.0002 | 0.001971 |
| Hcfc1r1 | chr17:23660522-23677449 | 84.4739 | 69.8307 | 0.826654 | 0.00095 | 0.006869 |
| 1810043G02Rik | chr10:77978649-77985438 | 25.8822 | 21.3962 | 0.826676 | 0.00595 | 0.028422 |
| Fn3krp | chr11:121421372-121430768 | 21.4947 | 17.7705 | 0.826739 | 0.0055 | 0.026705 |
| Ly6a | chr15:74994876-74998031 | 48.5778 | 40.1652 | 0.826822 | 0.0053 | 0.025948 |
| Tmem191c | chr16:17276299-17278661 | 44.2309 | 36.5808 | 0.827042 | 0.00205 | 0.012328 |
| Arhgef1 | chr7:24902985-24926591 | 37.1934 | 30.7632 | 0.827114 | 0.0002 | 0.001971 |
| Cwc15 | chr9:14500618-14510620 | 74.3062 | 61.4757 | 0.827329 | 0.00095 | 0.006869 |
| Cabp1 | chr5:115168690-115186121 | 69.932 | 57.8575 | 0.827339 | 0.00085 | 0.0063 |
| Fndc5 | chr4:129137059-129144593 | 38.6351 | 31.9656 | 0.827372 | 0.00045 | 0.003815 |
| Sharpin | chr15:76347039-76351110 | 30.7474 | 25.4403 | 0.827397 | 0.0033 | 0.017971 |
| Atxn7l2 | chr3:108202254-108210934 | 14.9064 | 12.3353 | 0.827517 | 0.00445 | 0.022502 |
| Ubl5 | chr9:20637786-20646789 | 63.3167 | 52.4029 | 0.827632 | 0.0009 | 0.006615 |
| Mmadhc | chr2:50279880-50296677 | 39.8113 | 32.9504 | 0.827665 | 0.00325 | 0.017763 |
| Cops6 | chr5:138161101-138163984 | 117.025 | 96.8938 | 0.827975 | 0.00025 | 0.002352 |
| Recql5 | chr11:115892594-115933492 | 14.0326 | 11.6203 | 0.828093 | 0.0023 | 0.013502 |
| Mrps7 | chr11:115604150-115607624 | 51.538 | 42.6798 | 0.828123 | 0.00165 | 0.010418 |
| Msrb2 | chr2:19371635-19394971 | 38.1029 | 31.5649 | 0.828412 | 0.0063 | 0.029601 |
| Cacfd1 | chr2:27009925-27021089 | 30.8099 | 25.5245 | 0.828451 | 0.0044 | 0.022281 |
| Stag1 | chr9:100643622-100958544 | 14.8372 | 12.2928 | 0.828512 | 0.0005 | 0.004159 |
| Nudt19 | chr7:35547184-35555928 | 37.8534 | 31.3643 | 0.828573 | 0.0008 | 0.006001 |
| Cuedc2 | chr19:46328183-46338660 | 92.0549 | 76.2973 | 0.828824 | 0.00015 | 0.001558 |
| Cpne7 | chr8:123117373-123135185 | 312.725 | 259.195 | 0.828827 | 0.00155 | 0.009917 |
| Coa3 | chr11:101277969-101278948 | 154.525 | 128.105 | 0.829024 | 0.0004 | 0.00346 |
| Hdac3 | chr18:37936970-37954988 | 75.7266 | 62.7836 | 0.829083 | 0.00025 | 0.002352 |
| Arpc2 | chr1:74236549-74268213 | 210.153 | 174.242 | 0.82912 | 0.00035 | 0.003088 |
| Col11a2 | chr17:34039436-34066242 | 7.81927 | 6.4832 | 0.829131 | 0.00515 | 0.025295 |
| Ube2m | chr7:13035119-13038275 | 161.724 | 134.093 | 0.829147 | 0.0004 | 0.00346 |
| AI854517 | chr7:79500025-79534403 | 12.7771 | 10.5951 | 0.829226 | 0.00465 | 0.023325 |
| Cdk11b | chr4:155624868-155649932 | 48.1661 | 39.9487 | 0.829395 | 0.00025 | 0.002352 |
| Prr3 | chr17:35972538-35979825 | 29.6652 | 24.607 | 0.82949 | 0.00395 | 0.020612 |
| Tcea2 | chr2:181680309-181688051 | 45.862 | 38.0439 | 0.82953 | 0.00335 | 0.018178 |
| Mrpl45 | chr11:97315715-97329920 | 21.2888 | 17.6601 | 0.829549 | 0.0031 | 0.017146 |
| Hs3st4 | chr7:123983180-124398989 | 38.3461 | 31.8104 | 0.82956 | 0.0004 | 0.00346 |
| Hspe1 | chr1:55088147-55091317 | 148.156 | 122.906 | 0.829572 | 0.00055 | 0.004491 |
| Bms1 | chr6:118383380-118419417 | 19.0951 | 15.8429 | 0.829684 | 0.0008 | 0.006001 |
| Syt7 | chr19:10389089-10453181 | 92.2496 | 76.5435 | 0.829743 | 0.00065 | 0.005126 |
| Fkbp1a | chr2:151542498-151561691 | 516.428 | 428.509 | 0.829756 | 0.00145 | 0.009402 |
| Zfp637 | chr6:117841241-117845956 | 34.345 | 28.5036 | 0.82992 | 0.0105 | 0.043929 |
| Clk2 | chr3:89164804-89177087 | 28.2145 | 23.4165 | 0.829946 | 0.00165 | 0.010418 |
| Zfp346 | chr13:55105308-55135071 | 17.6091 | 14.6189 | 0.83019 | 0.0019 | 0.01161 |
| Nr4a1 | chr15:101266845-101274794 | 29.3401 | 24.358 | 0.830195 | 0.001 | 0.007133 |
| Rps28 | chr17:33823036-33824498 | 699.573 | 580.799 | 0.830219 | 0.00025 | 0.002352 |
| Pak1ip1 | chr13:41001009-41013033 | 37.0802 | 30.7873 | 0.830289 | 0.00145 | 0.009402 |
| Eif3g | chr9:20894348-20898590 | 88.0309 | 73.0912 | 0.83029 | 0.00085 | 0.0063 |
| Fam213b | chr4:154896429-154899043 | 96.584 | 80.2048 | 0.830415 | 0.002 | 0.012075 |
| Trappc1 | chr11:69323985-69325793 | 71.0914 | 59.0364 | 0.83043 | 0.0051 | 0.025119 |
| Psmb2 | chr4:126677642-126709715 | 131.818 | 109.491 | 0.830623 | 0.00045 | 0.003815 |
| Gmppa | chr1:75435942-75443176 | 36.8422 | 30.6035 | 0.830664 | 0.0025 | 0.014398 |
| Myl12b | chr17:70973962-70990516 | 434.866 | 361.238 | 0.830688 | 0.00035 | 0.003088 |
| Rplp1 | chr9:61913282-61914510 | 1218.89 | 1012.67 | 0.830813 | 0.0001 | 0.001096 |
| 1110051M20Rik | chr2:91277827-91444642 | 68.3547 | 56.8023 | 0.830993 | 0.0002 | 0.001971 |
| Rwdd1 | chr10:33996554-34019616 | 60.2565 | 50.0791 | 0.831099 | 0.00195 | 0.011841 |
| Srp9 | chr1:182124736-182132415 | 72.5653 | 60.3241 | 0.831308 | 0.00055 | 0.004491 |
| Smim7 | chr8:72565197-72571048 | 40.5392 | 33.7059 | 0.83144 | 0.00035 | 0.003088 |
| Snrpd3 | chr10:75518041-75535440 | 116.225 | 96.6378 | 0.831472 | 0.0028 | 0.0158 |
| Ssbp4 | chr8:70597489-70608314 | 54.3503 | 45.1953 | 0.831556 | 0.00105 | 0.007395 |
| Me3 | chr7:89632817-89854359 | 14.5385 | 12.0914 | 0.831681 | 0.00195 | 0.011841 |
| Fah | chr7:84585158-84605942 | 23.2733 | 19.3632 | 0.831992 | 0.01185 | 0.048135 |
| Slc29a4 | chr5:142702100-142722490 | 24.4566 | 20.3496 | 0.83207 | 0.0012 | 0.008175 |
| Fbxo2 | chr4:148160667-148166417 | 72.5629 | 60.3803 | 0.83211 | 0.0009 | 0.006615 |
| Ercc4 | chr16:13109735-13152009 | 6.58138 | 5.47687 | 0.832177 | 0.00625 | 0.029392 |
| Atp6v1g2 | chr17:35236595-35238768 | 541.093 | 450.409 | 0.832406 | 0.0017 | 0.010645 |
| Kctd17 | chr15:78428627-78439303 | 101.556 | 84.5408 | 0.832455 | 0.0002 | 0.001971 |
| Grasp | chr15:101221190-101232756 | 31.2476 | 26.0161 | 0.832579 | 0.0037 | 0.019616 |
| Gng3 | chr19:8836928-8848683 | 136.93 | 114.005 | 0.832579 | 0.0034 | 0.018393 |
| Pes1 | chr11:3963974-3980004 | 18.7652 | 15.6236 | 0.832584 | 0.0032 | 0.01758 |
| Thbs3 | chr3:89215186-89226837 | 20.7881 | 17.3104 | 0.832707 | 0.00165 | 0.010418 |
| Prkag1 | chr15:98812796-98831508 | 43.2638 | 36.0403 | 0.833036 | 0.00125 | 0.008413 |
| Cope | chr8:70302784-70312990 | 109.106 | 90.8921 | 0.833062 | 0.0005 | 0.004159 |
| Tbkbp1 | chr11:97136170-97149712 | 36.7247 | 30.595 | 0.833091 | 0.00035 | 0.003088 |
| H2-Ke2 | chr17:33938908-33940343 | 109.719 | 91.4207 | 0.833226 | 0.00435 | 0.022102 |
| Ndufa8 | chr2:36036333-36049292 | 204.187 | 170.162 | 0.833364 | 0.00075 | 0.005715 |
| Dus1l | chr11:120789201-120796395 | 21.5852 | 17.9886 | 0.833377 | 0.00885 | 0.038577 |
| Cox7b | chrX:106015699-106022450 | 258.201 | 215.218 | 0.833529 | 0.0002 | 0.001971 |
| Oscp1 | chr4:126058564-126089334 | 18.7272 | 15.6119 | 0.833648 | 0.0087 | 0.038095 |
| Lcmt1 | chr7:123377981-123430358 | 72.6408 | 60.565 | 0.83376 | 0.001 | 0.007133 |
| Slc2a6 | chr2:27021364-27027998 | 23.0612 | 19.2276 | 0.833764 | 0.00385 | 0.02021 |
| Edf1 | chr2:25557899-25562082 | 222.747 | 185.743 | 0.833874 | 0.0005 | 0.004159 |
| Thoc3 | chr13:54458836-54468840 | 16.7439 | 13.9633 | 0.833934 | 0.0105 | 0.043929 |
| Igfbp4 | chr11:99041259-99052643 | 50.4117 | 42.0412 | 0.833957 | 0.0005 | 0.004159 |
| Crym | chr7:120186383-120201988 | 339.122 | 282.835 | 0.834021 | 0.00035 | 0.003088 |
| Sarnp | chr10:128821770-128877638 | 56.3489 | 46.9993 | 0.834077 | 0.00345 | 0.018588 |
| Wbscr17 | chr5:130874950-131307522 | 41.5989 | 34.7047 | 0.83427 | 0.00025 | 0.002352 |
| Park7 | chr4:150897132-150909921 | 195.741 | 163.318 | 0.834358 | 0.00035 | 0.003088 |
| Evc2 | chr5:37338477-37425054 | 11.1801 | 9.32855 | 0.834389 | 0.0057 | 0.027512 |
| Usp48 | chr4:137594188-137658537 | 36.1988 | 30.2102 | 0.834564 | 0.00045 | 0.003815 |
| Inf2 | chr12:112588783-112615557 | 33.4384 | 27.9192 | 0.834944 | 0.00035 | 0.003088 |
| Clk4 | chr11:51263170-51281764 | 43.7457 | 36.5275 | 0.834996 | 0.0011 | 0.007621 |
| Supt4a | chr11:87737564-87743617 | 86.319 | 72.0832 | 0.835079 | 0.00545 | 0.026547 |
| Setd1a | chr7:127777388-127800119 | 21.7748 | 18.188 | 0.835277 | 0.00025 | 0.002352 |
| Trp53inp2 | chr2:155381855-155389847 | 58.4098 | 48.7895 | 0.835296 | 0.00035 | 0.003088 |
| Ttc4 | chr4:106661807-106678944 | 37.2512 | 31.1183 | 0.835364 | 0.0009 | 0.006615 |
| Rasl11b | chr5:74195325-74199477 | 43.1628 | 36.0775 | 0.835847 | 0.0018 | 0.011147 |
| C1qc | chr4:136889801-136892914 | 50.5054 | 42.2263 | 0.836075 | 0.00645 | 0.030172 |
| Nsmaf | chr4:6396207-6454271 | 17.3856 | 14.5373 | 0.836169 | 0.0026 | 0.014885 |
| Lgi1 | chr19:38264781-38308939 | 71.4782 | 59.7841 | 0.836396 | 0.00045 | 0.003815 |
| Psma3 | chr12:70974622-71015823 | 94.239 | 78.8228 | 0.836414 | 0.001 | 0.007133 |
| Ndufb4 | chr16:37647601-37654368 | 315.455 | 263.852 | 0.836417 | 0.0007 | 0.005435 |
| Zfp488 | chr14:33967069-33978764 | 17.6525 | 14.7658 | 0.836471 | 0.0015 | 0.009667 |
| Gtl3 | chr8:95420249-95434869 | 38.6287 | 32.3172 | 0.836611 | 0.00675 | 0.031231 |
| C1qbp | chr11:70970199-70983026 | 71.4688 | 59.8011 | 0.836744 | 0.0026 | 0.014885 |
| Pddc1 | chr7:141408183-141414125 | 27.2252 | 22.7814 | 0.836776 | 0.0109 | 0.045122 |
| Ntrk3 | chr7:78192113-78577838 | 50.204 | 42.0096 | 0.836778 | 0.00025 | 0.002352 |
| Ssu72 | chr4:155704814-155733873 | 57.4818 | 48.1016 | 0.836814 | 0.0018 | 0.011147 |
| Asphd2 | chr5:112385444-112392213 | 84.5559 | 70.7601 | 0.836844 | 0.0006 | 0.004799 |
| Clcn7 | chr17:25133393-25162099 | 25.8761 | 21.6596 | 0.83705 | 0.00065 | 0.005126 |
| Maf1 | chr15:76351293-76354378 | 51.0074 | 42.7026 | 0.837184 | 0.0015 | 0.009667 |
| Trmt6 | chr2:132804214-132816054 | 17.3474 | 14.523 | 0.837186 | 0.0051 | 0.025119 |
| Iscu | chr5:113772811-113778282 | 117.198 | 98.1173 | 0.837193 | 0.00145 | 0.009402 |
| Mybbp1a | chr11:72441377-72451550 | 25.1103 | 21.031 | 0.837545 | 0.00065 | 0.005126 |
| Mki67ip | chr1:118321842-118333831 | 22.2572 | 18.6518 | 0.838012 | 0.01075 | 0.04464 |
| 2610301B20Rik | chr4:10874497-10899423 | 26.5618 | 22.2627 | 0.838147 | 0.004 | 0.020812 |
| Hdac1 | chr4:129516103-129542646 | 29.8189 | 25.005 | 0.838562 | 0.0038 | 0.020026 |
| Pxn | chr5:115506701-115555986 | 23.6095 | 19.7988 | 0.838595 | 0.00125 | 0.008413 |
| Cxxc1 | chr18:74216211-74221491 | 30.8542 | 25.8846 | 0.838933 | 0.0017 | 0.010645 |
| Ndufa7 | chr17:33824613-33838313 | 247.581 | 207.705 | 0.838938 | 0.00165 | 0.010418 |
| Dlg3 | chrX:100767722-100818410 | 81.1834 | 68.1086 | 0.838947 | 0.0016 | 0.010187 |
| Leprel2 | chr6:124841094-124857687 | 22.1299 | 18.568 | 0.839046 | 0.0033 | 0.017971 |
| Fam216a | chr5:122364583-122371963 | 83.4127 | 70.0034 | 0.839242 | 0.0019 | 0.01161 |
| Orai3 | chr7:127769814-127775150 | 21.7521 | 18.256 | 0.839275 | 0.0098 | 0.041724 |
| Rpl29 | chr9:106429538-106431567 | 374.203 | 314.078 | 0.839325 | 0.00045 | 0.003815 |
| 2210016L21Rik | chr5:114942201-114948540 | 124.298 | 104.373 | 0.8397 | 0.00065 | 0.005126 |
| Comt | chr16:18348181-18479073 | 49.2857 | 41.3886 | 0.839769 | 0.00895 | 0.038901 |
| Polr2j | chr5:136116690-136122947 | 95.8277 | 80.5023 | 0.840073 | 0.00865 | 0.037923 |
| Ube2s | chr7:4808013-4812340 | 87.9105 | 73.8578 | 0.840148 | 0.00165 | 0.010418 |
| Brd9 | chr13:73937837-73960895 | 80.0839 | 67.2886 | 0.840226 | 0.00045 | 0.003815 |
| Pmvk | chr3:89459117-89469009 | 55.6869 | 46.8005 | 0.840422 | 0.0039 | 0.020391 |
| Tle2 | chr10:81575286-81590845 | 18.3232 | 15.4029 | 0.840623 | 0.00625 | 0.029392 |
| Babam1 | chr8:71396854-71404772 | 52.0917 | 43.8039 | 0.8409 | 0.0015 | 0.009667 |
| Cacnb2 | chr2:14604305-14987908 | 21.1798 | 17.8126 | 0.841018 | 0.0014 | 0.009144 |
| Eif1b | chr9:120492605-120495327 | 173.734 | 146.156 | 0.841263 | 0.0008 | 0.006001 |
| Tmem59l | chr8:70483866-70487358 | 333.349 | 280.458 | 0.841334 | 0.001 | 0.007133 |
| Guk1 | chr11:59183854-59191952 | 150.021 | 126.22 | 0.841349 | 0.00075 | 0.005715 |
| Apmap | chr2:150583080-150608523 | 32.6121 | 27.452 | 0.841773 | 0.0027 | 0.01535 |
| Rrp1 | chr10:78400361-78413043 | 242.248 | 203.996 | 0.842096 | 0.00165 | 0.010418 |
| Chst1 | chr2:92599706-92615252 | 165.372 | 139.286 | 0.842259 | 0.00115 | 0.007885 |
| Nudc | chr4:133532541-133546027 | 117.714 | 99.1485 | 0.842283 | 0.00075 | 0.005715 |
| Dync1i2 | chr2:71211705-71263302 | 106.625 | 89.8102 | 0.8423 | 0.0011 | 0.007621 |
| Psma4 | chr9:54950256-54958030 | 132.645 | 111.736 | 0.842369 | 0.0009 | 0.006615 |
| Cyc1 | chr15:76343522-76345934 | 204.958 | 172.657 | 0.842402 | 0.0007 | 0.005435 |
| Epha10 | chr4:124881784-124917800 | 17.7457 | 14.9501 | 0.842463 | 0.0023 | 0.013502 |
| Tia1 | chr6:86404218-86433405 | 33.5792 | 28.2897 | 0.842477 | 0.0011 | 0.007621 |
| Cog2 | chr8:124520766-124552007 | 13.8291 | 11.6513 | 0.84252 | 0.0124 | 0.049986 |
| Fam195b | chr11:120542887-120549727 | 75.6056 | 63.7011 | 0.842545 | 0.0019 | 0.01161 |
| Cxx1b | chrX:53669176-53670408 | 62.5007 | 52.6676 | 0.842672 | 0.0023 | 0.013502 |
| B3gat1 | chr9:26751561-26761338 | 125.025 | 105.358 | 0.842695 | 0.00115 | 0.007885 |
| Hax1 | chr3:89995456-89998686 | 63.9732 | 53.9122 | 0.842731 | 0.00275 | 0.015584 |
| Ppp1r11 | chr17:36948354-36951792 | 61.445 | 51.7972 | 0.842985 | 0.0013 | 0.008656 |
| Eif3k | chr7:28971372-28981814 | 211.456 | 178.256 | 0.842993 | 0.00065 | 0.005126 |
| Asic2 | chr11:80880164-81968396 | 28.8684 | 24.3387 | 0.843091 | 0.00245 | 0.014171 |
| Sirt7 | chr11:120618371-120625002 | 34.4011 | 29.008 | 0.843229 | 0.00385 | 0.02021 |
| Naa20 | chr2:145903240-145916425 | 59.6638 | 50.3123 | 0.843263 | 0.0038 | 0.020026 |
| Higd1a | chr9:121848559-121858000 | 59.5059 | 50.1803 | 0.843283 | 0.00145 | 0.009402 |
| Ly6e | chr15:74955050-74959905 | 149.491 | 126.068 | 0.843315 | 0.0007 | 0.005435 |
| Plekha5 | chr6:140424098-140594906 | 20.8836 | 17.613 | 0.843389 | 0.00125 | 0.008413 |
| Enho | chr4:41569793-41640302 | 44.6393 | 37.6504 | 0.843436 | 0.0079 | 0.035261 |
| Uqcrfs1 | chr13:30540311-30545316 | 119.555 | 100.839 | 0.843453 | 0.00085 | 0.0063 |
| Stk32c | chr7:139103637-139242973 | 58.3174 | 49.1895 | 0.843479 | 0.00255 | 0.014638 |
| Hsbp1 | chr8:119344537-119348929 | 261.811 | 220.854 | 0.843563 | 0.00085 | 0.0063 |
| Ptcd3 | chr6:71880637-71908762 | 28.0968 | 23.7027 | 0.843609 | 0.00235 | 0.013705 |
| Sstr3 | chr15:78537014-78544345 | 13.0664 | 11.0245 | 0.843729 | 0.00595 | 0.028422 |
| Cyth1 | chr11:118164165-118248592 | 51.4215 | 43.3861 | 0.843735 | 0.001 | 0.007133 |
| Rnf25 | chr1:74593751-74601397 | 53.4199 | 45.0778 | 0.843839 | 0.00235 | 0.013705 |
| Smyd2 | chr1:189880491-189922288 | 52.0362 | 43.9175 | 0.84398 | 0.0024 | 0.013943 |
| Telo2 | chr17:25099568-25115967 | 13.2043 | 11.1444 | 0.843998 | 0.011 | 0.045394 |
| Psmb1 | chr17:15475720-15498276 | 172.766 | 145.823 | 0.844049 | 0.00115 | 0.007885 |
| Ccndbp1 | chr2:121008407-121016912 | 37.3434 | 31.5287 | 0.844291 | 0.0054 | 0.026352 |
| Ccnl2 | chr4:155812488-155824543 | 67.3991 | 56.9057 | 0.844309 | 0.0008 | 0.006001 |
| Rnf181 | chr6:72355482-72362381 | 62.1071 | 52.4376 | 0.844309 | 0.0068 | 0.031381 |
| Mdm2 | chr10:117688904-117710716 | 22.4588 | 18.9624 | 0.844319 | 0.00355 | 0.018973 |
| Rae1 | chr2:173000116-173015739 | 36.2967 | 30.6479 | 0.844372 | 0.0044 | 0.022281 |
| Tmem147 | chr7:30727700-30729534 | 90.523 | 76.438 | 0.844404 | 0.0036 | 0.019163 |
| Ndor1 | chr2:25244812-25255414 | 17.0412 | 14.393 | 0.8446 | 0.0025 | 0.014398 |
| Sltm | chr9:70542777-70592232 | 38.7864 | 32.7623 | 0.844685 | 0.00105 | 0.007395 |
| Kctd13 | chr7:126928878-126945609 | 115.63 | 97.6799 | 0.844763 | 0.00065 | 0.005126 |
| Actr3b | chr5:25760025-25850341 | 75.0342 | 63.391 | 0.844828 | 0.00155 | 0.009917 |
| Ubp1 | chr9:113930933-114026751 | 55.1988 | 46.638 | 0.84491 | 0.00495 | 0.02454 |
| Arrb2 | chr11:70432579-70440828 | 52.6811 | 44.5118 | 0.844929 | 0.00245 | 0.014171 |
| Cttnbp2 | chr6:18366476-18514825 | 48.5548 | 41.0332 | 0.84509 | 0.0011 | 0.007621 |
| Snx11 | chr11:96767548-96777555 | 20.7871 | 17.5678 | 0.84513 | 0.0078 | 0.034859 |
| Dcaf11 | chr14:55560028-55570065 | 50.4007 | 42.5984 | 0.845195 | 0.00145 | 0.009402 |
| Rpl10a | chr17:28328470-28331033 | 290.07 | 245.169 | 0.845206 | 0.0007 | 0.005435 |
| Polr1d | chr5:147077345-147111361 | 66.8992 | 56.5554 | 0.845382 | 0.0055 | 0.026705 |
| Eif2b5 | chr16:20498816-20509325 | 31.7602 | 26.8504 | 0.84541 | 0.00185 | 0.011363 |
| Trank1 | chr9:111311738-111395775 | 91.1485 | 77.0717 | 0.845562 | 0.0086 | 0.03775 |
| Cox7c | chr13:86044797-86046795 | 515.17 | 435.631 | 0.845606 | 0.00075 | 0.005715 |
| Eif5a | chr11:69916711-69921958 | 306.905 | 259.535 | 0.845653 | 0.0013 | 0.008656 |
| Kcnip4 | chr5:48389502-49285659 | 32.53 | 27.5102 | 0.845687 | 0.00515 | 0.025295 |
| Prodh | chr16:18071725-18089190 | 19.3811 | 16.3965 | 0.846005 | 0.01095 | 0.045258 |
| Safb2 | chr17:56562941-56584583 | 43.8433 | 37.1087 | 0.846394 | 0.00085 | 0.0063 |
| Idh2 | chr7:80094846-80115350 | 34.2025 | 28.95 | 0.846429 | 0.00685 | 0.031529 |
| Thoc5 | chr11:4895342-4928865 | 25.5794 | 21.6519 | 0.846458 | 0.00685 | 0.031529 |
| Mif | chr10:75859352-75860250 | 311.102 | 263.453 | 0.846838 | 0.0014 | 0.009144 |
| Napa | chr7:16098642-16117975 | 146.278 | 123.884 | 0.846908 | 0.0011 | 0.007621 |
| Ndufb8 | chr19:44550253-44555415 | 347.818 | 294.662 | 0.847173 | 0.00095 | 0.006869 |
| Psme4 | chr11:30771774-30880361 | 15.4023 | 13.0517 | 0.847386 | 0.0019 | 0.01161 |
| Ramp1 | chr1:91179821-91225196 | 38.4922 | 32.6211 | 0.847473 | 0.0039 | 0.020391 |
| Lmo7 | chr14:101729927-101934693 | 22.531 | 19.0957 | 0.84753 | 0.00135 | 0.008911 |
| Epha7 | chr4:28813144-28967499 | 26.5219 | 22.4818 | 0.847669 | 0.00505 | 0.024965 |
| Brk1 | chr6:113604771-113616951 | 220.957 | 187.306 | 0.847703 | 0.00115 | 0.007885 |
| Sh3glb2 | chr2:30344808-30359231 | 143.289 | 121.49 | 0.847867 | 0.00095 | 0.006869 |
| Btg1 | chr10:96617000-96622811 | 9.13814 | 7.74822 | 0.847899 | 0.0118 | 0.047968 |
| Cpne6 | chr14:55510447-55517431 | 476.502 | 404.106 | 0.848068 | 0.01015 | 0.042887 |
| Rplp0 | chr5:115559466-115563729 | 237.426 | 201.369 | 0.848134 | 0.00105 | 0.007395 |
| Srsf9 | chr5:115327176-115333080 | 53.1562 | 45.088 | 0.848217 | 0.00575 | 0.027666 |
| Fyco1 | chr9:123789509-123851899 | 27.1034 | 22.9949 | 0.848414 | 0.00095 | 0.006869 |
| Tyk2 | chr9:21104067-21131275 | 10.0768 | 8.54944 | 0.848428 | 0.00875 | 0.038251 |
| Ring1 | chr17:34020791-34024680 | 23.2369 | 19.7165 | 0.8485 | 0.01215 | 0.049165 |
| Ivd | chr2:118861999-118881357 | 56.0444 | 47.5639 | 0.848682 | 0.0017 | 0.010645 |
| Arpc1a | chr5:145083868-145108756 | 175.762 | 149.167 | 0.848687 | 0.0011 | 0.007621 |
| Ica1 | chr6:8630526-8778484 | 41.9144 | 35.5795 | 0.848861 | 0.00705 | 0.032226 |
| Sec11c | chr18:65800577-65817657 | 123.794 | 105.094 | 0.848943 | 0.00285 | 0.016005 |
| Bod1 | chr11:31665149-31671862 | 76.7574 | 65.1871 | 0.849261 | 0.00195 | 0.011841 |
| Nfyc | chr4:120757434-120831575 | 29.5272 | 25.0772 | 0.849292 | 0.00715 | 0.032557 |
| Unc13b | chr4:43058983-43264887 | 17.9554 | 15.2509 | 0.849377 | 0.0015 | 0.009667 |
| Get4 | chr5:139252323-139270050 | 30.3253 | 25.7587 | 0.849413 | 0.00625 | 0.029392 |
| Chmp3 | chr6:71543853-71581574 | 50.3959 | 42.8171 | 0.849615 | 0.0016 | 0.010187 |
| Fam114a2 | chr11:57482989-57518644 | 25.2031 | 21.4203 | 0.849907 | 0.00545 | 0.026547 |
| 2410015M20Rik | chr17:56607451-56609771 | 99.5806 | 84.667 | 0.850236 | 0.00625 | 0.029392 |
| Rabggta | chr14:55715876-55722176 | 23.3159 | 19.8375 | 0.850814 | 0.0067 | 0.031027 |
| Aup1 | chr6:83054652-83057682 | 54.6863 | 46.5323 | 0.850895 | 0.00345 | 0.018588 |
| Ctxn1 | chr8:4257645-4259274 | 412.064 | 350.641 | 0.850938 | 0.0024 | 0.013943 |
| Rpl15 | chr14:18267822-18270986 | 165.2 | 140.665 | 0.851483 | 0.0014 | 0.009144 |
| Hnrnpab | chr11:51584756-51606881 | 67.9276 | 57.8403 | 0.851499 | 0.00175 | 0.010881 |
| Smpd4 | chr16:17619353-17644830 | 33.28 | 28.3387 | 0.851523 | 0.0017 | 0.010645 |
| Tmem66 | chr8:34154562-34170847 | 180.833 | 153.985 | 0.851532 | 0.0022 | 0.013067 |
| Atp5g1 | chr11:96072792-96075694 | 287.858 | 245.176 | 0.851726 | 0.00115 | 0.007885 |
| Zfp259 | chr9:46273063-46282642 | 18.1623 | 15.4713 | 0.851836 | 0.00915 | 0.039575 |
| Rbx1 | chr15:81466315-81476369 | 91.5181 | 77.9844 | 0.85212 | 0.00195 | 0.011841 |
| Vti1b | chr12:79130787-79172458 | 113.049 | 96.3451 | 0.852242 | 0.0024 | 0.013943 |
| 1810037I17Rik | chr3:122924396-122926194 | 58.145 | 49.5587 | 0.85233 | 0.01235 | 0.049841 |
| Bpgm | chr6:34476355-34505610 | 39.9556 | 34.0565 | 0.852359 | 0.00345 | 0.018588 |
| Farsa | chr8:84856985-84869257 | 29.5372 | 25.1796 | 0.852471 | 0.01105 | 0.04553 |
| Ndufab1 | chr7:122088043-122101848 | 81.7264 | 69.6712 | 0.852493 | 0.0086 | 0.03775 |
| Yeats2 | chr16:20141062-20232573 | 16.0412 | 13.6756 | 0.85253 | 0.00415 | 0.021362 |
| Ppp1ca | chr19:4192173-4201603 | 159.418 | 135.916 | 0.852576 | 0.0028 | 0.0158 |
| Per3 | chr4:151003654-151044622 | 16.0419 | 13.6799 | 0.852761 | 0.00275 | 0.015584 |
| Mapk1ip1 | chr7:138835817-138846267 | 57.2982 | 48.8766 | 0.853022 | 0.0046 | 0.023162 |
| Htatsf1 | chrX:57053569-57067182 | 51.1209 | 43.6308 | 0.853483 | 0.00135 | 0.008911 |
| Ppa1 | chr10:61648620-61674165 | 77.7784 | 66.4022 | 0.853736 | 0.0036 | 0.019163 |
| Gtf2h5 | chr17:6079827-6085485 | 77.8403 | 66.4625 | 0.853831 | 0.00455 | 0.022964 |
| Preb | chr5:30950065-30960361 | 20.4814 | 17.4895 | 0.853921 | 0.00265 | 0.01513 |
| Pllp | chr8:94674894-94696242 | 28.7978 | 24.5917 | 0.853944 | 0.0107 | 0.044502 |
| Chid1 | chr7:141475235-141539857 | 20.4381 | 17.4563 | 0.854106 | 0.00735 | 0.033325 |
| Tubgcp6 | chr15:89089106-89123150 | 12.7624 | 10.9019 | 0.85422 | 0.00645 | 0.030172 |
| Otub1 | chr19:7198205-7206284 | 177.728 | 151.826 | 0.85426 | 0.0017 | 0.010645 |
| Hebp1 | chr6:135137518-135168215 | 58.6848 | 50.1376 | 0.854354 | 0.01075 | 0.04464 |
| Prkdc | chr16:15637865-15842239 | 5.16163 | 4.4103 | 0.854439 | 0.0061 | 0.028943 |
| Abhd16a | chr17:35089290-35102987 | 49.5552 | 42.3468 | 0.854538 | 0.0029 | 0.016217 |
| Med28 | chr5:45520228-45529284 | 15.7225 | 13.4401 | 0.854832 | 0.0055 | 0.026705 |
| Naca | chr10:128035345-128048637 | 252.143 | 215.589 | 0.855027 | 0.00225 | 0.013268 |
| AI593442 | chr9:52673041-52679429 | 25.402 | 21.7269 | 0.855322 | 0.0019 | 0.01161 |
| Sugp1 | chr8:70042812-70071953 | 31.7857 | 27.1871 | 0.855325 | 0.00515 | 0.025295 |
| Gmpr | chr13:45507468-45546381 | 46.5446 | 39.8198 | 0.855519 | 0.00585 | 0.028071 |
| Chmp2a | chr7:13032005-13034777 | 144.302 | 123.47 | 0.855636 | 0.0025 | 0.014398 |
| Zfp821 | chr8:109705548-109724932 | 33.4206 | 28.5974 | 0.855682 | 0.0071 | 0.032371 |
| Pithd1 | chr4:135975601-135987244 | 73.3163 | 62.7367 | 0.855699 | 0.00305 | 0.01694 |
| Nrbp2 | chr15:76085593-76090013 | 149.607 | 128.069 | 0.856036 | 0.0026 | 0.014885 |
| Sdccag3 | chr2:26382799-26389316 | 69.806 | 59.7643 | 0.856148 | 0.0021 | 0.012579 |
| Gps1 | chr11:120784271-120789102 | 95.431 | 81.716 | 0.856284 | 0.00165 | 0.010418 |
| Zfp932 | chr5:109996526-110010411 | 53.5936 | 45.9136 | 0.856699 | 0.0038 | 0.020026 |
| Scd1 | chr19:44394449-44407709 | 38.2643 | 32.7896 | 0.856924 | 0.00285 | 0.016005 |
| Tango2 | chr16:18300824-18343932 | 61.3578 | 52.5795 | 0.856933 | 0.00495 | 0.02454 |
| Abhd12 | chr2:150832514-150904731 | 195.479 | 167.523 | 0.856987 | 0.00355 | 0.018973 |
| Lpl | chr8:68880554-68906932 | 14.7057 | 12.6031 | 0.857021 | 0.00925 | 0.039894 |
| Ube2r2 | chr4:41136020-41193370 | 32.5678 | 27.9124 | 0.857055 | 0.0037 | 0.019616 |
| Gpc1 | chr1:92831685-92860196 | 41.742 | 35.7828 | 0.857237 | 0.00305 | 0.01694 |
| Pfdn2 | chr1:171345698-171358170 | 141.984 | 121.721 | 0.857287 | 0.0081 | 0.035882 |
| Psmc1 | chr12:100112330-100123364 | 123.455 | 105.842 | 0.857333 | 0.0017 | 0.010645 |
| Gde1 | chr7:118688557-118705738 | 169.577 | 145.413 | 0.857504 | 0.00165 | 0.010418 |
| Arhgdig | chr17:26199182-26201350 | 61.9102 | 53.1054 | 0.857781 | 0.01085 | 0.044968 |
| Auh | chr13:52835109-52929677 | 47.829 | 41.0325 | 0.8579 | 0.01065 | 0.044364 |
| Stx3 | chr19:11775117-11819403 | 27.2453 | 23.3745 | 0.857928 | 0.00865 | 0.037923 |
| Fkbp2 | chr19:6977738-6980461 | 156.303 | 134.151 | 0.858275 | 0.0062 | 0.029235 |
| Ergic3 | chr2:156008124-156018279 | 107.76 | 92.5008 | 0.858396 | 0.00225 | 0.013268 |
| Dtnbp1 | chr13:44922079-45002096 | 61.3302 | 52.6626 | 0.858673 | 0.0059 | 0.028247 |
| Snx17 | chr5:31193303-31198900 | 49.7075 | 42.6849 | 0.858722 | 0.0051 | 0.025119 |
| Psat1 | chr19:15905122-15925059 | 58.0649 | 49.8639 | 0.858761 | 0.00345 | 0.018588 |
| Becn1 | chr11:101279202-101302267 | 80.9027 | 69.4812 | 0.858824 | 0.00315 | 0.017359 |
| BC018242 | chr9:21938273-21948907 | 133.053 | 114.317 | 0.859184 | 0.00275 | 0.015584 |
| Atp13a1 | chr8:69791162-69807748 | 23.0535 | 19.8087 | 0.859249 | 0.0045 | 0.022734 |
| Ran | chr5:129020155-129024321 | 123.142 | 105.818 | 0.859317 | 0.0032 | 0.01758 |
| Mlf2 | chr6:124931387-124936149 | 359.17 | 308.848 | 0.859894 | 0.00575 | 0.027666 |
| Kdm1a | chr4:136550532-136602723 | 31.4439 | 27.0455 | 0.860119 | 0.00485 | 0.024168 |
| Zfp512 | chr5:31452435-31481753 | 24.1045 | 20.7343 | 0.860184 | 0.00525 | 0.025738 |
| Gsta4 | chr9:78191965-78209349 | 71.8952 | 61.8528 | 0.860319 | 0.0105 | 0.043929 |
| Pkig | chr2:163658385-163726158 | 63.7331 | 54.8328 | 0.86035 | 0.0111 | 0.045683 |
| Iffo1 | chr6:125145240-125161782 | 32.1842 | 27.6902 | 0.860366 | 0.0052 | 0.025505 |
| Esd | chr14:74732344-74750443 | 48.5019 | 41.7377 | 0.860537 | 0.00695 | 0.031865 |
| Pmm1 | chr15:81951105-81960867 | 170.871 | 147.056 | 0.860626 | 0.00325 | 0.017763 |
| Rab28 | chr5:41624975-41708155 | 39.3545 | 33.8711 | 0.860667 | 0.01105 | 0.04553 |
| Slc25a3 | chr10:91116577-91123963 | 314.016 | 270.334 | 0.860892 | 0.00475 | 0.023714 |
| Atp6v1f | chr6:29467782-29470509 | 232.456 | 200.294 | 0.861643 | 0.0044 | 0.022281 |
| Pdap1 | chr5:145128769-145140089 | 58.4094 | 50.3309 | 0.861692 | 0.00355 | 0.018973 |
| BC018507 | chr13:70588688-70637634 | 39.0628 | 33.6641 | 0.861794 | 0.0041 | 0.021197 |
| Smarcb1 | chr10:75896768-75921614 | 57.8986 | 49.9016 | 0.861879 | 0.00565 | 0.027333 |
| 4-Sep | chr11:87581130-87590539 | 107.003 | 92.3151 | 0.862734 | 0.0032 | 0.01758 |
| D10Wsu52e | chr10:85938636-85957793 | 73.6353 | 63.5604 | 0.863178 | 0.00335 | 0.018178 |
| Sdhb | chr4:140961270-140979192 | 167.875 | 144.966 | 0.863535 | 0.00285 | 0.016005 |
| Begain | chr12:109032181-109068217 | 52.4231 | 45.2713 | 0.863575 | 0.00415 | 0.021362 |
| Dagla | chr19:10245264-10304877 | 36.2096 | 31.2808 | 0.863881 | 0.00295 | 0.016462 |
| Abcc8 | chr7:46104522-46180033 | 13.7076 | 11.8436 | 0.864017 | 0.01035 | 0.043473 |
| Atg2a | chr19:6241667-6262304 | 14.6942 | 12.6975 | 0.864116 | 0.0061 | 0.028943 |
| Ndufa12 | chr10:94199008-94220948 | 293.567 | 253.692 | 0.864171 | 0.00475 | 0.023714 |
| Arfgap1 | chr2:180967224-180982524 | 57.6548 | 49.8259 | 0.864211 | 0.00435 | 0.022102 |
| Tma7 | chr9:109077987-109082381 | 210.812 | 182.189 | 0.864225 | 0.00565 | 0.027333 |
| Hmgb1 | chr5:149047226-149053037 | 124.252 | 107.412 | 0.864469 | 0.00405 | 0.021031 |
| Mpp3 | chr11:101999652-102026955 | 32.5638 | 28.1548 | 0.864604 | 0.0064 | 0.029991 |
| Akr1a1 | chr4:116636509-116651674 | 295.042 | 255.126 | 0.864711 | 0.0055 | 0.026705 |
| Slc44a2 | chr9:21320718-21355028 | 20.2849 | 17.5533 | 0.865338 | 0.0098 | 0.041724 |
| Ist1 | chr8:109614516-109693294 | 63.4471 | 54.904 | 0.865351 | 0.00545 | 0.026547 |
| Cpt1c | chr7:44959371-44974851 | 71.7425 | 62.1263 | 0.865962 | 0.00425 | 0.021761 |
| Cpsf7 | chr19:10525243-10547735 | 42.8507 | 37.1129 | 0.866098 | 0.0042 | 0.021546 |
| Grcc10 | chr6:124739183-124741079 | 214.698 | 185.972 | 0.866203 | 0.0077 | 0.034557 |
| Hnrnpl | chr7:28810889-28822266 | 91.6766 | 79.4231 | 0.86634 | 0.00465 | 0.023325 |
| Cdk10 | chr8:123224840-123232256 | 62.4635 | 54.1186 | 0.866404 | 0.0076 | 0.034268 |
| Mbd3 | chr10:80392540-80399479 | 84.7074 | 73.4171 | 0.866714 | 0.0059 | 0.028247 |
| Appl2 | chr10:83600033-83648664 | 58.3321 | 50.5837 | 0.867167 | 0.0049 | 0.02436 |
| Arfgap2 | chr2:91265114-91277371 | 30.4065 | 26.3741 | 0.867384 | 0.0071 | 0.032371 |
| Taf15 | chr11:83473107-83506740 | 54.9875 | 47.702 | 0.867506 | 0.0063 | 0.029601 |
| Rin1 | chr19:5050807-5057071 | 31.8911 | 27.6669 | 0.867543 | 0.00505 | 0.024965 |
| Arfip2 | chr7:105634200-105640416 | 42.7484 | 37.0911 | 0.867661 | 0.005 | 0.024764 |
| Mdh2 | chr5:135778648-135790386 | 346.133 | 300.41 | 0.867903 | 0.0069 | 0.031704 |
| B3gat3 | chr19:8920392-8927236 | 110.191 | 95.667 | 0.868193 | 0.0054 | 0.026352 |
| Nabp2 | chr10:128401394-128409796 | 83.3129 | 72.3324 | 0.868202 | 0.00965 | 0.041251 |
| Ppp1cc | chr5:122158278-122175269 | 105.917 | 91.9848 | 0.868461 | 0.0047 | 0.023553 |
| Chchd10 | chr10:75935572-75937734 | 117.679 | 102.203 | 0.86849 | 0.0091 | 0.039456 |
| Otud7b | chr3:96080595-96161129 | 9.62372 | 8.35956 | 0.868641 | 0.0099 | 0.042065 |
| Fam193b | chr13:55539315-55571120 | 58.635 | 50.9342 | 0.868665 | 0.0046 | 0.023162 |
| Fnbp4 | chr2:90745369-90781020 | 20.9501 | 18.2068 | 0.869056 | 0.00795 | 0.03541 |
| S100b | chr10:76253835-76261319 | 82.4286 | 71.6369 | 0.869078 | 0.00555 | 0.026911 |
| Psmd14 | chr2:61711693-61800376 | 72.67 | 63.1604 | 0.86914 | 0.00725 | 0.032942 |
| Akap8l | chr17:32321423-32350577 | 109.733 | 95.4431 | 0.869776 | 0.00515 | 0.025295 |
| Pnn | chr12:59066918-59074017 | 55.5737 | 48.3394 | 0.869825 | 0.00585 | 0.028071 |
| Erp29 | chr5:121444752-121452474 | 101.38 | 88.1905 | 0.8699 | 0.00665 | 0.030876 |
| Coro1b | chr19:4148662-4154035 | 49.0014 | 42.6277 | 0.869928 | 0.0095 | 0.04079 |
| Ociad2 | chr5:73322198-73338639 | 63.4809 | 55.268 | 0.870624 | 0.0073 | 0.03314 |
| Car11 | chr7:45699966-45704661 | 100.002 | 87.0784 | 0.870767 | 0.00695 | 0.031865 |
| 15-Sep | chr3:144570426-144597676 | 110.14 | 95.9127 | 0.870825 | 0.00655 | 0.030505 |
| Wscd1 | chr11:71750702-71789646 | 57.0958 | 49.7212 | 0.870838 | 0.006 | 0.028609 |
| Tagln3 | chr16:45711229-45724531 | 246.399 | 214.591 | 0.870909 | 0.00535 | 0.026156 |
| Psmd8 | chr7:29174186-29180673 | 80.4305 | 70.0794 | 0.871304 | 0.00815 | 0.036058 |
| Phldb1 | chr9:44686307-44735198 | 28.7026 | 25.0087 | 0.871304 | 0.00575 | 0.027666 |
| Psmc4 | chr7:28041701-28050092 | 97.6849 | 85.1195 | 0.871368 | 0.0057 | 0.027512 |
| Fbxw5 | chr2:25498649-25505470 | 57.8988 | 50.4683 | 0.871664 | 0.0077 | 0.034557 |
| Agrn | chr4:156165289-156197488 | 61.3078 | 53.4407 | 0.871679 | 0.00975 | 0.041578 |
| Lgi3 | chr14:70530820-70538324 | 47.3884 | 41.3209 | 0.871962 | 0.0061 | 0.028943 |
| Sf3b1 | chr1:54985169-55027478 | 82.4199 | 71.8894 | 0.872234 | 0.0124 | 0.049986 |
| Vdac3 | chr8:22577074-22593813 | 142.372 | 124.198 | 0.872348 | 0.0065 | 0.030326 |
| Rpa1 | chr11:75300258-75348383 | 26.7525 | 23.3379 | 0.872363 | 0.01215 | 0.049165 |
| Psmb7 | chr2:38588045-38643906 | 184.131 | 160.63 | 0.872368 | 0.0062 | 0.029235 |
| Sfswap | chr5:129501230-129571384 | 47.2008 | 41.18 | 0.872443 | 0.00705 | 0.032226 |
| C2cd5 | chr6:143010919-143100107 | 30.4383 | 26.5568 | 0.87248 | 0.0075 | 0.033932 |
| 9530082P21Rik | chr17:23749235-23754065 | 58.3374 | 50.9133 | 0.872739 | 0.00725 | 0.032942 |
| Gnb2l1 | chr11:48800359-48806241 | 212.143 | 185.178 | 0.872892 | 0.00755 | 0.034086 |
| Tmem222 | chr4:133266044-133277790 | 76.5476 | 66.8203 | 0.872925 | 0.01005 | 0.042515 |
| Clta | chr4:44012642-44032846 | 204.108 | 178.172 | 0.87293 | 0.00775 | 0.034738 |
| 5730409E04Rik | chr4:126609853-126614371 | 43.6236 | 38.081 | 0.872945 | 0.00805 | 0.035765 |
| Chmp4b | chr2:154657025-154694783 | 137.309 | 119.874 | 0.873024 | 0.00765 | 0.034391 |
| Ndufs2 | chr1:171234859-171247112 | 170.782 | 149.118 | 0.873148 | 0.0065 | 0.030326 |
| Psd | chr19:46304736-46327156 | 119.985 | 104.792 | 0.873376 | 0.01205 | 0.048798 |
| Prpf38b | chr3:108902806-108911704 | 32.4491 | 28.3536 | 0.873787 | 0.0111 | 0.045683 |
| Tmem9b | chr7:109735835-109752263 | 80.349 | 70.2524 | 0.874341 | 0.00865 | 0.037923 |
| Srsf11 | chr3:158010492-158036639 | 76.1901 | 66.6216 | 0.874413 | 0.0082 | 0.036265 |
| Pink1 | chr4:138313409-138326296 | 174.297 | 152.424 | 0.874507 | 0.0103 | 0.043331 |
| 2410089E03Rik | chr15:8169105-8271158 | 13.0131 | 11.3804 | 0.874534 | 0.0081 | 0.035882 |
| Col6a1 | chr10:76708791-76726044 | 43.9252 | 38.4178 | 0.874619 | 0.00765 | 0.034391 |
| Chka | chr19:3851772-3894369 | 45.8729 | 40.1254 | 0.874708 | 0.0096 | 0.04107 |
| Daam2 | chr17:49456021-49564337 | 26.3583 | 23.0659 | 0.875091 | 0.0078 | 0.034859 |
| Arglu1 | chr8:8666575-8690537 | 72.1372 | 63.1371 | 0.875236 | 0.01115 | 0.045835 |
| Nhp2l1 | chr15:82041344-82047598 | 127.257 | 111.383 | 0.87526 | 0.0078 | 0.034859 |
| Skp1a | chr11:52231994-52246858 | 300.941 | 263.649 | 0.876082 | 0.0118 | 0.047968 |
| Eif3e | chr15:43250039-43282736 | 78.3629 | 68.6542 | 0.876106 | 0.01095 | 0.045258 |
| Pfkl | chr10:77986948-78009796 | 95.9875 | 84.0958 | 0.876112 | 0.0106 | 0.044208 |
| Pdxp | chr15:78913918-78919517 | 113.413 | 99.3982 | 0.876427 | 0.0084 | 0.036995 |
| Scg5 | chr2:113776312-113829091 | 185.566 | 162.646 | 0.876486 | 0.00915 | 0.039575 |
| Rapgefl1 | chr11:98836784-98853005 | 71.0551 | 62.2865 | 0.876594 | 0.00975 | 0.041578 |
| Lancl1 | chr1:67000516-67038872 | 52.6188 | 46.1391 | 0.876856 | 0.0084 | 0.036995 |
| B3gnt1 | chr19:5038825-5041134 | 67.8717 | 59.5162 | 0.876893 | 0.01025 | 0.043155 |
| Dctn5 | chr7:122133040-122149044 | 81.0638 | 71.0912 | 0.876978 | 0.01075 | 0.04464 |
| Atp5c1 | chr2:10056030-10080510 | 250.343 | 219.588 | 0.877149 | 0.00945 | 0.040592 |
| Hnrpdl | chr5:100033578-100039222 | 77.6472 | 68.1142 | 0.877227 | 0.00945 | 0.040592 |
| Actl6b | chr5:137553554-137569573 | 98.8111 | 86.7013 | 0.877445 | 0.00895 | 0.038901 |
| Elp2 | chr18:24603960-24638830 | 51.296 | 45.019 | 0.877632 | 0.01015 | 0.042887 |
| Ryr2 | chr13:11553102-12106945 | 23.7855 | 20.8773 | 0.877732 | 0.0117 | 0.047671 |
| Eif3h | chr15:51786562-51865461 | 131.436 | 115.37 | 0.877766 | 0.0103 | 0.043331 |
| Psmc3 | chr2:91054015-91059438 | 128.823 | 113.094 | 0.877902 | 0.01005 | 0.042515 |
| Ntsr2 | chr12:16653469-16660236 | 101.762 | 89.3894 | 0.878416 | 0.00935 | 0.04026 |
| Fam212b | chr3:105704598-105720842 | 27.378 | 24.0505 | 0.878461 | 0.0105 | 0.043929 |
| Cltb | chr13:54592938-54611272 | 94.8073 | 83.2899 | 0.878518 | 0.01155 | 0.047187 |
| Asna1 | chr8:85017930-85025278 | 98.0291 | 86.1235 | 0.87855 | 0.0118 | 0.047968 |
| Cnbp | chr6:87842614-87851106 | 145.123 | 127.518 | 0.878689 | 0.01135 | 0.046603 |
| Atf4 | chr15:80255183-80257541 | 119.185 | 104.767 | 0.879028 | 0.01075 | 0.04464 |
| Sgta | chr10:81044072-81060154 | 83.602 | 73.5601 | 0.879884 | 0.0108 | 0.044795 |
| Htra1 | chr7:130936202-130985658 | 99.1912 | 87.2772 | 0.879889 | 0.01005 | 0.042515 |
| Ipo9 | chr1:135382314-135430491 | 39.6242 | 34.88 | 0.88027 | 0.011 | 0.045394 |
| Eri3 | chr4:117550493-117674281 | 107.916 | 95.0844 | 0.881096 | 0.01105 | 0.04553 |
| Ddx1 | chr12:13219306-13249174 | 65.2019 | 57.4799 | 0.881568 | 0.01205 | 0.048798 |

HAL, haloperidol; NS, normal saline

**Table S3. The results of gene ontology for significantly upregulated genes (>1.3-fold change)**

| **GOID** | **GO Term** | **Benjamini-Hochberg**  **P value** | **Number of genes** |
| --- | --- | --- | --- |
| **Biological process** | | | |
| GO:0010613 | positive regulation of cardiac muscle hypertrophy | 0.000236 | 7 |
| GO:0007193 | adenylate cyclase-inhibiting G protein-coupled receptor signaling pathway | 0.000344 | 10 |
| GO:0018107 | peptidyl-threonine phosphorylation | 0.000526 | 11 |
| GO:0070884 | regulation of calcineurin-NFAT signaling cascade | 0.000678 | 6 |
| GO:0090101 | negative regulation of transmembrane receptor protein serine/threonine kinase signaling pathway | 0.000256 | 12 |
| GO:0061014 | positive regulation of mRNA catabolic process | 0.000323 | 7 |
| GO:1900151 | regulation of nuclear-transcribed mRNA catabolic process, deadenylation-dependent decay | 0.000323 | 5 |
| GO:0030371 | translation repressor activity | 0.000438 | 5 |
| GO:2000766 | negative regulation of cytoplasmic translation | 0.000438 | 4 |
| GO:0032869 | cellular response to insulin stimulus | 0.000302 | 18 |
| GO:0008286 | insulin receptor signaling pathway | 0.000302 | 13 |
| GO:0006865 | amino acid transport | 0.000075 | 16 |
| GO:0015807 | L-amino acid transport | 0.000075 | 10 |
| GO:1902023 | L-arginine transport | 0.000075 | 4 |
| GO:0018198 | peptidyl-cysteine modification | 0.000253 | 8 |
| GO:0018119 | peptidyl-cysteine S-nitrosylation | 0.000253 | 5 |
| GO:2000169 | regulation of peptidyl-cysteine S-nitrosylation | 0.000253 | 4 |
| GO:0019933 | cAMP-mediated signaling | 0.000319 | 16 |
| GO:0007189 | adenylate cyclase-activating G protein-coupled receptor signaling pathway | 0.000319 | 14 |
| GO:0046058 | cAMP metabolic process | 0.000319 | 5 |
| GO:0007269 | neurotransmitter secretion | < 0.000001 | 15 |
| GO:0050804 | modulation of chemical synaptic transmission | < 0.000001 | 52 |
| GO:0050806 | positive regulation of synaptic transmission | < 0.000001 | 27 |
| GO:0048167 | regulation of synaptic plasticity | < 0.000001 | 28 |
| GO:0046928 | regulation of neurotransmitter secretion | < 0.000001 | 15 |
| GO:0060079 | excitatory postsynaptic potential | < 0.000001 | 13 |
| GO:0060291 | long-term synaptic potentiation | < 0.000001 | 15 |
| GO:0098815 | modulation of excitatory postsynaptic potential | < 0.000001 | 10 |
| GO:1900271 | regulation of long-term synaptic potentiation | < 0.000001 | 9 |
| GO:2000300 | regulation of synaptic vesicle exocytosis | < 0.000001 | 12 |
| GO:2000311 | regulation of AMPA receptor activity | < 0.000001 | 6 |
| GO:0004971 | AMPA glutamate receptor activity | < 0.000001 | 6 |
| GO:0098815 | modulation of excitatory postsynaptic potential | < 0.000001 | 10 |
| GO:0016358 | dendrite development | < 0.000001 | 28 |
| GO:0060996 | dendritic spine development | < 0.000001 | 13 |
| GO:0097107 | postsynaptic density assembly | < 0.000001 | 6 |
| GO:1900006 | positive regulation of dendrite development | < 0.000001 | 12 |
| GO:0050773 | regulation of dendrite development | < 0.000001 | 23 |
| GO:0060998 | regulation of dendritic spine development | < 0.000001 | 13 |
| GO:0060999 | positive regulation of dendritic spine development | < 0.000001 | 8 |
| GO:0005237 | inhibitory extracellular ligand-gated ion channel activity | < 0.000001 | 5 |
| GO:0022824 | transmitter-gated ion channel activity | < 0.000001 | 17 |
| GO:2000311 | regulation of AMPA receptor activity | < 0.000001 | 6 |
| GO:0004971 | AMPA glutamate receptor activity | < 0.000001 | 6 |
| GO:0050806 | positive regulation of synaptic transmission | < 0.000001 | 27 |
| GO:0099565 | chemical synaptic transmission, postsynaptic | < 0.000001 | 23 |
| GO:0005216 | ion channel activity | < 0.000001 | 43 |
| GO:0022836 | gated channel activity | < 0.000001 | 36 |
| GO:0051966 | regulation of synaptic transmission, glutamatergic | < 0.000001 | 10 |
| GO:0060079 | excitatory postsynaptic potential | < 0.000001 | 13 |
| GO:0060291 | long-term synaptic potentiation | < 0.000001 | 15 |
| GO:0098815 | modulation of excitatory postsynaptic potential | < 0.000001 | 10 |
| GO:0098960 | postsynaptic neurotransmitter receptor activity | < 0.000001 | 14 |
| GO:0005244 | voltage-gated ion channel activity | < 0.000001 | 22 |
| GO:0005261 | cation channel activity | < 0.000001 | 34 |
| GO:0015276 | ligand-gated ion channel activity | < 0.000001 | 22 |
| GO:0097107 | postsynaptic density assembly | < 0.000001 | 6 |
| GO:2001257 | regulation of cation channel activity | < 0.000001 | 19 |
| GO:0060999 | positive regulation of dendritic spine development | < 0.000001 | 8 |
| GO:0099094 | ligand-gated cation channel activity | < 0.000001 | 16 |
| GO:0099095 | ligand-gated anion channel activity | < 0.000001 | 5 |
| GO:0004970 | ionotropic glutamate receptor activity | < 0.000001 | 11 |
| GO:1904315 | transmitter-gated ion channel activity involved in regulation of postsynaptic membrane potential | < 0.000001 | 9 |
| GO:0048666 | neuron development | < 0.000001 | 80 |
| GO:0050767 | regulation of neurogenesis | < 0.000001 | 73 |
| GO:0050768 | negative regulation of neurogenesis | < 0.000001 | 27 |
| GO:0050769 | positive regulation of neurogenesis | < 0.000001 | 42 |
| GO:0045664 | regulation of neuron differentiation | < 0.000001 | 60 |
| GO:0031175 | neuron projection development | < 0.000001 | 72 |
| GO:0048667 | cell morphogenesis involved in neuron differentiation | < 0.000001 | 45 |
| GO:0045665 | negative regulation of neuron differentiation | < 0.000001 | 22 |
| GO:0045666 | positive regulation of neuron differentiation | < 0.000001 | 36 |
| GO:0016358 | dendrite development | < 0.000001 | 28 |
| GO:0060996 | dendritic spine development | < 0.000001 | 13 |
| GO:0048813 | dendrite morphogenesis | < 0.000001 | 18 |
| GO:0007411 | axon guidance | < 0.000001 | 18 |
| GO:0010975 | regulation of neuron projection development | < 0.000001 | 48 |
| GO:0010976 | positive regulation of neuron projection development | < 0.000001 | 30 |
| GO:0010977 | negative regulation of neuron projection development | < 0.000001 | 16 |
| GO:0048812 | neuron projection morphogenesis | < 0.000001 | 47 |
| GO:1900006 | positive regulation of dendrite development | < 0.000001 | 12 |
| GO:0061564 | axon development | < 0.000001 | 32 |
| GO:0050773 | regulation of dendrite development | < 0.000001 | 23 |
| GO:0007409 | axonogenesis | < 0.000001 | 30 |
| GO:0048814 | regulation of dendrite morphogenesis | < 0.000001 | 13 |
| GO:0060998 | regulation of dendritic spine development | < 0.000001 | 13 |
| GO:0060999 | positive regulation of dendritic spine development | < 0.000001 | 8 |
| **Cellular components** | | | |
| GO:0031674 | I band | 0.001546 | 13 |
| GO:0034705 | potassium channel complex | 0.002188 | 9 |
| GO:0099026 | anchored component of presynaptic membrane | 0.003172 | 3 |
| GO:0099056 | integral component of presynaptic membrane | 0.000119 | 13 |
| GO:0000118 | histone deacetylase complex | 0.005115 | 7 |
| GO:0016581 | NuRD complex | 0.005115 | 4 |
| GO:0097444 | spine apparatus | 0.001295 | 3 |
| GO:0099065 | integral component of spine apparatus membrane | 0.001295 | 2 |
| GO:0098839 | postsynaptic density membrane | < 0.000001 | 16 |
| GO:0099055 | integral component of postsynaptic membrane | < 0.000001 | 22 |
| GO:0099060 | integral component of postsynaptic specialization membrane | < 0.000001 | 16 |
| GO:0099061 | integral component of postsynaptic density membrane | < 0.000001 | 12 |
| **Molecular function** | | | |
| GO:0099635 | voltage-gated calcium channel activity involved in positive regulation of presynaptic cytosolic calcium levels | 0.001303 | 3 |
| GO:0022843 | voltage-gated cation channel activity | 0.000001 | 21 |
| GO:0000064 | L-ornithine transmembrane transporter activity | 0.000035 | 3 |
| GO:0015179 | L-amino acid transmembrane transporter activity | 0.000035 | 9 |
| GO:0015181 | arginine transmembrane transporter activity | 0.000035 | 3 |
| GO:0015189 | L-lysine transmembrane transporter activity | 0.000035 | 3 |
| GO:0099094 | ligand-gated cation channel activity | < 0.000001 | 16 |
| GO:0099095 | ligand-gated anion channel activity | < 0.000001 | 5 |
| GO:0022824 | transmitter-gated ion channel activity | < 0.000001 | 17 |
| GO:0004970 | ionotropic glutamate receptor activity | < 0.000001 | 11 |
| GO:0005237 | inhibitory extracellular ligand-gated ion channel activity | < 0.000001 | 5 |
| GO:2000311 | regulation of AMPA receptor activity | < 0.000001 | 6 |
| GO:1904315 | transmitter-gated ion channel activity involved in regulation of postsynaptic membrane potential | < 0.000001 | 9 |
| GO:0004971 | AMPA glutamate receptor activity | < 0.000001 | 6 |
| GO:0004972 | NMDA glutamate receptor activity | < 0.000001 | 5 |

HAL, haloperidol; Number, the number of genes that belonged to each GO term, NS, normal saline

**Table S4. The results of gene ontology for significantly downregulated genes (>1.3-fold change)**

| **GOID** | **GO Term** | **Benjamini-Hochberg**  **P value** | **Number of genes** |
| --- | --- | --- | --- |
| **Biological process** | | | |
| GO:0006364 | rRNA processing | 0.000036 | 15 |
| GO:0015252 | proton channel activity | 0.002164 | 4 |
| GO:0030490 | maturation of SSU-rRNA | 0.001742 | 5 |
| GO:1990948 | ubiquitin ligase inhibitor activity | 0.000653 | 3 |
| GO:1901798 | positive regulation of signal transduction by p53 class mediator | 0.000653 | 5 |
| GO:1903321 | negative regulation of protein modification by small protein conjugation or removal | 0.000653 | 7 |
| GO:1990948 | ubiquitin ligase inhibitor activity | 0.000815 | 3 |
| GO:1901798 | positive regulation of signal transduction by p53 class mediator | 0.000815 | 5 |
| GO:2001244 | positive regulation of intrinsic apoptotic signaling pathway | 0.000815 | 6 |
| GO:1902253 | regulation of intrinsic apoptotic signaling pathway by p53 class mediator | 0.000815 | 5 |
| GO:1902255 | positive regulation of intrinsic apoptotic signaling pathway by p53 class mediator | 0.000815 | 3 |
| **Cellular components** | | | |
| GO:0005753 | mitochondrial proton-transporting ATP synthase complex | 0.009765 | 3 |
| GO:0005762 | mitochondrial large ribosomal subunit | 0.000037 | 8 |
| GO:0005763 | mitochondrial small ribosomal subunit | 0.000640 | 5 |
| GO:0012510 | trans-Golgi network transport vesicle membrane | 0.021044 | 2 |
| GO:0046540 | U4/U6 x U5 tri-snRNP complex | 0.006034 | 4 |
| GO:0098800 | inner mitochondrial membrane protein complex | < 0.000001 | 17 |
| GO:0005746 | mitochondrial respirasome | < 0.000001 | 12 |
| GO:0005743 | mitochondrial inner membrane | < 0.000001 | 27 |
| GO:0005747 | mitochondrial respiratory chain complex I | < 0.000001 | 10 |
| **Molecular function** | | | |
| GO:0015252 | proton channel activity | 0.001002 | 4 |

HAL, haloperidol; Number, the number of genes that belonged to each GO term, NS, normal saline

**Table S5. Predicted miRNAs relevant to upregulated genes in RNA-seq**

| **Upstream Regulator** | **Molecule Type** | **P value** |
| --- | --- | --- |
| mir-8 | microRNA | 1.26E-11 |
| miR-17-5p (and other miRNAs w/seed AAAGUGC) | mature microRNA | 1.46E-09 |
| miR-26a-5p (and other miRNAs w/seed UCAAGUA) | mature microRNA | 2.59E-07 |
| miR-1-3p (and other miRNAs w/seed GGAAUGU) | mature microRNA | 1.45E-06 |
| miR-486-5p (and other miRNAs w/seed CCUGUAC) | mature microRNA | 4.62E-06 |
| mir-486 | microRNA | 7.31E-06 |
| miR-30c-5p (and other miRNAs w/seed GUAAACA) | mature microRNA | 1.38E-05 |
| mir-19 | microRNA | 2.06E-05 |
| miR-291a-3p (and other miRNAs w/seed AAGUGCU) | mature microRNA | 3.07E-05 |
| mir-181 | microRNA | 4.48E-05 |
| mir-26 | microRNA | 4.86E-05 |
| mir-17 | microRNA | 5.31E-05 |
| miR-132-3p (and other miRNAs w/seed AACAGUC) | mature microRNA | 6.77E-05 |
| mir-148 | microRNA | 9.01E-05 |
| mir-132 | microRNA | 0.000204 |
| mir-192 | microRNA | 0.000651 |
| miR-204-5p (and other miRNAs w/seed UCCCUUU) | mature microRNA | 0.000675 |
| mir-1 | microRNA | 0.000727 |
| mir-122 | microRNA | 0.000757 |
| miR-135a-5p (and other miRNAs w/seed AUGGCUU) | mature microRNA | 0.000768 |
| mir-25 | microRNA | 0.000778 |
| miR-126a-3p (and other miRNAs w/seed CGUACCG) | mature microRNA | 0.00104 |
| miR-129-5p (and other miRNAs w/seed UUUUUGC) | mature microRNA | 0.0011 |
| mir-10 | microRNA | 0.00129 |
| miR-29b-3p (and other miRNAs w/seed AGCACCA) | mature microRNA | 0.00138 |
| mir-204 | microRNA | 0.00147 |
| mir-185 | microRNA | 0.00166 |
| mir-133 | microRNA | 0.00168 |
| miR-92a-3p (and other miRNAs w/seed AUUGCAC) | mature microRNA | 0.00182 |
| mir-103 | microRNA | 0.00186 |
| mir-214 | microRNA | 0.00233 |
| miR-221-3p (and other miRNAs w/seed GCUACAU) | mature microRNA | 0.00263 |
| mir-126 | microRNA | 0.00263 |
| mir-210 | microRNA | 0.00278 |
| mir-485 | microRNA | 0.00323 |
| miR-375-3p (miRNAs w/seed UUGUUCG) | mature microRNA | 0.00357 |
| miR-34a-5p (and other miRNAs w/seed GGCAGUG) | mature microRNA | 0.00369 |
| miR-122-5p (miRNAs w/seed GGAGUGU) | mature microRNA | 0.00394 |
| miR-293-5p (and other miRNAs w/seed CUCAAAC) | mature microRNA | 0.0056 |
| miR-21-5p (and other miRNAs w/seed AGCUUAU) | mature microRNA | 0.00605 |
| miR-140-5p (and other miRNAs w/seed AGUGGUU) | mature microRNA | 0.00829 |
| miR-30a-3p (and other miRNAs w/seed UUUCAGU) | mature microRNA | 0.00842 |
| mir-221 | microRNA | 0.00849 |
| miR-96-5p (and other miRNAs w/seed UUGGCAC) | mature microRNA | 0.00859 |
| mir-183 | microRNA | 0.00866 |
| miR-222-5p (miRNAs w/seed UCAGUAG) | mature microRNA | 0.00934 |
| miR-199a-5p (and other miRNAs w/seed CCAGUGU) | mature microRNA | 0.00979 |
| mir-375 | microRNA | 0.0119 |
| mir-9 | microRNA | 0.0121 |
| mir-137 | microRNA | 0.0137 |
| miR-16-5p (and other miRNAs w/seed AGCAGCA) | mature microRNA | 0.0139 |
| miR-450a-5p (and other miRNAs w/seed UUUGCGA) | mature microRNA | 0.015 |
| miR-127-3p (miRNAs w/seed CGGAUCC) | mature microRNA | 0.018 |
| miR-339-5p (and other miRNAs w/seed CCCUGUC) | mature microRNA | 0.018 |
| mir-138 | microRNA | 0.018 |
| miR-574-5p (miRNAs w/seed GAGUGUG) | mature microRNA | 0.018 |
| miR-19b-3p (and other miRNAs w/seed GUGCAAA) | mature microRNA | 0.0203 |
| miR-124-3p (and other miRNAs w/seed AAGGCAC) | mature microRNA | 0.0233 |
| miR-27a-3p (and other miRNAs w/seed UCACAGU) | mature microRNA | 0.0245 |
| miR-141-3p (and other miRNAs w/seed AACACUG) | mature microRNA | 0.0261 |
| miR-155-5p (miRNAs w/seed UAAUGCU) | mature microRNA | 0.0261 |
| miR-10a-5p (and other miRNAs w/seed ACCCUGU) | mature microRNA | 0.0275 |
| miR-126a-5p (and other miRNAs w/seed AUUAUUA) | mature microRNA | 0.0275 |
| mir-665 | microRNA | 0.0288 |
| mir-135 | microRNA | 0.0293 |
| miR-103-3p (and other miRNAs w/seed GCAGCAU) | mature microRNA | 0.0336 |
| miR-199a-3p (and other miRNAs w/seed CAGUAGU) | mature microRNA | 0.0336 |
| mir-379 | microRNA | 0.0342 |
| mir-223 | microRNA | 0.0352 |
| miR-18a-5p (and other miRNAs w/seed AAGGUGC) | mature microRNA | 0.0389 |
| miR-483-3p (miRNAs w/seed CACUCCU) | mature microRNA | 0.041 |
| miR-9-3p (and other miRNAs w/seed UAAAGCU) | mature microRNA | 0.0416 |
| miR-137-3p (miRNAs w/seed UAUUGCU) | mature microRNA | 0.0416 |
| miR-3118 (and other miRNAs w/seed GUGACUG) | mature microRNA | 0.0416 |
| miR-148a-3p (and other miRNAs w/seed CAGUGCA) | mature microRNA | 0.0417 |

**Table S6. Predicted target genes of mmu-miR-137-3p**

| **Gene** | **EntrezID** | **RefseqID** | **miRWalk** | **Microt4** | **miRanda** | **miRDB** | **Pictar2** | **PITA** | **RNA22** | **Targetscan** | **SUM** |
| --- | --- | --- | --- | --- | --- | --- | --- | --- | --- | --- | --- |
| Hmgcll1 | 208982 | NM_173731 | 1 | 1 | 1 | 1 | 0 | 1 | 1 | 1 | 7 |
| Kcnab3 | 16499 | NM_010599 | 1 | 1 | 1 | 1 | 0 | 1 | 1 | 1 | 7 |
| Dock7 | 67299 | NM_026082 | 1 | 1 | 1 | 1 | 0 | 1 | 1 | 1 | 7 |
| Snx25 | 102141 | NM_207213 | 1 | 1 | 1 | 1 | 0 | 1 | 1 | 1 | 7 |
| Pdcd6 | 18570 | NM_011051 | 1 | 1 | 1 | 1 | 0 | 1 | 1 | 1 | 7 |
| Tmem56 | 99887 | NM_178936 | 1 | 1 | 1 | 1 | 0 | 1 | 1 | 1 | 7 |
| Tmprss11f | 243083 | NM_178730 | 1 | 1 | 1 | 1 | 0 | 1 | 1 | 1 | 7 |
| Ric3 | 320360 | NM_001038624 | 1 | 1 | 1 | 1 | 0 | 1 | 1 | 1 | 7 |
| Shroom2 | 110380 | NM_172441 | 1 | 1 | 1 | 1 | 0 | 1 | 1 | 1 | 7 |
| Cdh12 | 215654 | NM_001008420 | 1 | 1 | 1 | 1 | 0 | 1 | 1 | 1 | 7 |
| Ccbe1 | 320924 | NM_178793 | 1 | 1 | 1 | 1 | 0 | 1 | 1 | 1 | 7 |
| Iqgap1 | 29875 | NM_016721 | 1 | 1 | 1 | 1 | 0 | 1 | 1 | 1 | 7 |
| Tmem229b | 268567 | NM_178745 | 1 | 1 | 1 | 1 | 0 | 1 | 1 | 1 | 7 |
| Cped1 | 214642 | NM_001081351 | 1 | 1 | 1 | 1 | 0 | 1 | 1 | 1 | 7 |
| Itln1 | 16429 | NM_010584 | 1 | 1 | 1 | 1 | 0 | 1 | 1 | 1 | 7 |
| Rassf4 | 213391 | NM_178045 | 1 | 1 | 1 | 1 | 0 | 1 | 1 | 1 | 7 |
| Prkab1 | 19079 | NM_031869 | 1 | 1 | 1 | 1 | 0 | 1 | 1 | 1 | 7 |
| Ugt2b34 | 100727 | NM_153598 | 1 | 1 | 1 | 1 | 0 | 1 | 1 | 1 | 7 |
| Cr2 | 12902 | NM_007758 | 1 | 1 | 1 | 1 | 0 | 1 | 1 | 1 | 7 |
| Fam216b | 219170 | NM_177629 | 1 | 1 | 1 | 1 | 0 | 1 | 1 | 1 | 7 |
| Ccni | 12453 | NM_017367 | 1 | 1 | 1 | 1 | 0 | 1 | 1 | 1 | 7 |
| Suv39h2 | 64707 | NM_022724 | 1 | 1 | 1 | 1 | 0 | 1 | 1 | 1 | 7 |
| Syncrip | 56403 | NM_019796 | 1 | 1 | 1 | 1 | 0 | 1 | 1 | 1 | 7 |
| Prdm1 | 12142 | NM_007548 | 1 | 1 | 1 | 1 | 0 | 1 | 1 | 1 | 7 |
| Hsd17b13 | 243168 | NM_198030 | 1 | 1 | 1 | 1 | 0 | 1 | 1 | 1 | 7 |
| Gxylt1 | 223827 | NM_001033275 | 1 | 1 | 1 | 1 | 0 | 1 | 1 | 1 | 7 |
| Col10a1 | 12813 | NM_009925 | 1 | 1 | 1 | 1 | 0 | 1 | 1 | 1 | 7 |
| Dcdc2a | 195208 | NM_177577 | 1 | 1 | 1 | 1 | 0 | 1 | 1 | 1 | 7 |
| Birc5 | 11799 | NM_001012273 | 1 | 1 | 1 | 1 | 0 | 1 | 1 | 1 | 7 |
| Ybx1 | 22608 | NM_011732 | 1 | 1 | 1 | 1 | 0 | 1 | 1 | 1 | 7 |
| Slc12a2 | 20496 | NM_009194 | 1 | 1 | 1 | 1 | 0 | 1 | 1 | 1 | 7 |
| Sptlc1 | 268656 | NM_009269 | 1 | 1 | 1 | 1 | 0 | 1 | 1 | 1 | 7 |
| Cdc37l1 | 67072 | NM_025950 | 1 | 1 | 1 | 1 | 0 | 1 | 1 | 1 | 7 |
| Neurod4 | 11923 | NM_007501 | 1 | 1 | 1 | 1 | 0 | 1 | 1 | 1 | 7 |
| Esrra | 26379 | NM_007953 | 1 | 1 | 1 | 1 | 0 | 1 | 1 | 1 | 7 |
| Tdrd7 | 100121 | NM_146142 | 1 | 1 | 1 | 1 | 0 | 1 | 1 | 1 | 7 |
| Tssk1 | 22114 | NM_009435 | 1 | 1 | 1 | 1 | 0 | 1 | 1 | 1 | 7 |
| Ulk2 | 29869 | NM_013881 | 1 | 1 | 1 | 1 | 0 | 1 | 1 | 1 | 7 |
| Chd9 | 109151 | NM_177224 | 1 | 1 | 1 | 1 | 0 | 1 | 1 | 1 | 7 |
| Raver2 | 242570 | NM_183024 | 1 | 1 | 1 | 1 | 0 | 1 | 1 | 1 | 7 |
| 1700001C19Rik | 75462 | NM_029296 | 1 | 1 | 1 | 1 | 0 | 1 | 1 | 1 | 7 |
| Rhox6 | 19202 | NM_008955 | 1 | 1 | 1 | 1 | 0 | 1 | 1 | 1 | 7 |
| Fat3 | 270120 | NM_001080814 | 1 | 1 | 1 | 1 | 0 | 1 | 1 | 1 | 7 |
| T | 20997 | NM_009309 | 1 | 1 | 1 | 1 | 0 | 1 | 1 | 1 | 7 |
| Rell1 | 100532 | NM_145923 | 1 | 1 | 1 | 1 | 0 | 1 | 1 | 1 | 7 |
| Otc | 18416 | NM_008769 | 1 | 1 | 1 | 1 | 0 | 1 | 1 | 1 | 7 |
| 1190002N15Rik | 68861 | NM_001033145 | 1 | 1 | 1 | 1 | 0 | 1 | 1 | 1 | 7 |
| Nck1 | 17973 | NM_010878 | 1 | 1 | 1 | 1 | 0 | 1 | 1 | 1 | 7 |
| Paip2b | 232164 | NM_146169 | 1 | 1 | 1 | 1 | 0 | 1 | 1 | 1 | 7 |
| Vash2 | 226841 | NM_144879 | 1 | 1 | 1 | 1 | 0 | 1 | 1 | 1 | 7 |
| Eml5 | 319670 | NM_001081191 | 1 | 1 | 1 | 1 | 0 | 1 | 1 | 1 | 7 |
| Nmnat3 | 74080 | NM_144533 | 1 | 1 | 1 | 1 | 0 | 1 | 1 | 1 | 7 |
| Ahcyl2 | 74340 | NM_021414 | 1 | 1 | 1 | 1 | 0 | 1 | 1 | 1 | 7 |
| Cpsf6 | 432508 | NM_001013391 | 1 | 1 | 1 | 1 | 0 | 1 | 1 | 1 | 7 |
| Zfp36l2 | 12193 | NM_001001806 | 1 | 1 | 1 | 1 | 0 | 1 | 1 | 1 | 7 |
| 2810403A07Rik | 74200 | NM_028814 | 1 | 1 | 1 | 1 | 0 | 1 | 1 | 1 | 7 |
| 2210010C04Rik | 67373 | NM_023333 | 1 | 1 | 1 | 1 | 0 | 1 | 1 | 1 | 7 |
| Atpaf1 | 230649 | NM_181040 | 1 | 1 | 1 | 1 | 0 | 1 | 1 | 1 | 7 |
| Tspan2 | 70747 | NM_027533 | 1 | 1 | 1 | 1 | 0 | 1 | 1 | 1 | 7 |
| Nat8l | 269642 | NM_001001985 | 1 | 1 | 1 | 1 | 0 | 1 | 1 | 1 | 7 |
| Mid2 | 23947 | NM_011845 | 1 | 1 | 1 | 1 | 0 | 1 | 1 | 1 | 7 |
| Dlgap1 | 224997 | NM_177639 | 1 | 1 | 1 | 1 | 0 | 1 | 1 | 1 | 7 |
| Bdp1 | 544971 | NM_001081061 | 1 | 1 | 1 | 1 | 0 | 1 | 1 | 1 | 7 |
| Zfp385a | 29813 | NM_013866 | 1 | 1 | 1 | 1 | 0 | 1 | 1 | 1 | 7 |
| Scrt1 | 170729 | NM_130893 | 1 | 1 | 1 | 1 | 0 | 1 | 1 | 1 | 7 |
| Ube3c | 100763 | NM_133907 | 1 | 1 | 1 | 1 | 0 | 1 | 1 | 1 | 7 |
| Ubn2 | 320538 | NM_177185 | 1 | 1 | 1 | 1 | 0 | 1 | 1 | 1 | 7 |
| Cttnbp2nl | 80281 | NM_030249 | 1 | 1 | 1 | 1 | 0 | 1 | 1 | 1 | 7 |
| Cxxc4 | 319478 | NM_001004367 | 1 | 1 | 1 | 1 | 0 | 1 | 1 | 1 | 7 |
| Prr16 | 71373 | NM_001081224 | 1 | 1 | 1 | 1 | 0 | 1 | 1 | 1 | 7 |
| Snrk | 20623 | NM_133741 | 1 | 1 | 1 | 1 | 0 | 1 | 1 | 1 | 7 |
| Cep97 | 74201 | NM_028815 | 1 | 1 | 1 | 1 | 0 | 1 | 1 | 1 | 7 |
| Fgl2 | 14190 | NM_008013 | 1 | 1 | 1 | 1 | 0 | 1 | 1 | 1 | 7 |
| Ddx21 | 56200 | NM_019553 | 1 | 1 | 1 | 1 | 0 | 1 | 1 | 1 | 7 |
| Ctdspl | 69274 | NM_133710 | 1 | 1 | 1 | 1 | 0 | 1 | 1 | 1 | 7 |
| Chchd5 | 66170 | NM_025395 | 1 | 1 | 1 | 1 | 0 | 1 | 1 | 1 | 7 |
| Ppp4r2 | 232314 | NM_182939 | 1 | 1 | 1 | 1 | 0 | 1 | 1 | 1 | 7 |
| Clic5 | 224796 | NM_172621 | 1 | 1 | 1 | 1 | 0 | 1 | 1 | 1 | 7 |
| Ap3s1 | 11777 | NM_009681 | 1 | 1 | 1 | 1 | 0 | 1 | 1 | 1 | 7 |
| Erg | 13876 | NM_133659 | 1 | 1 | 1 | 1 | 0 | 1 | 1 | 1 | 7 |
| Sh3bp5 | 24056 | NM_011894 | 1 | 1 | 1 | 1 | 0 | 1 | 1 | 1 | 7 |
| Mef2a | 17258 | NM_001033713 | 1 | 1 | 1 | 1 | 0 | 1 | 1 | 1 | 7 |
| Hlf | 217082 | NM_172563 | 1 | 1 | 1 | 1 | 0 | 1 | 1 | 1 | 7 |
| Gpr17 | 574402 | NM_001025381 | 1 | 1 | 1 | 0 | 0 | 1 | 1 | 1 | 6 |
| Stx8 | 55943 | NM_018768 | 1 | 1 | 1 | 0 | 0 | 1 | 1 | 1 | 6 |
| Slc17a6 | 140919 | NM_080853 | 1 | 1 | 1 | 0 | 0 | 1 | 1 | 1 | 6 |
| Kif3b | 16569 | NM_008444 | 1 | 1 | 1 | 0 | 0 | 1 | 1 | 1 | 6 |
| Slc30a4 | 22785 | NM_011774 | 1 | 1 | 1 | 1 | 0 | 1 | 0 | 1 | 6 |
| Sulf1 | 240725 | NM_001198565 | 1 | 1 | 1 | 1 | 0 | 0 | 1 | 1 | 6 |
| Neurod1 | 18012 | NM_010894 | 1 | 1 | 1 | 1 | 0 | 1 | 0 | 1 | 6 |
| Sv2a | 64051 | NM_022030 | 1 | 1 | 1 | 0 | 0 | 1 | 1 | 1 | 6 |
| Kirrel | 170643 | NM_001170985 | 1 | 1 | 1 | 1 | 0 | 0 | 1 | 1 | 6 |
| Zfp277 | 246196 | NM_172575 | 1 | 1 | 1 | 1 | 0 | 1 | 0 | 1 | 6 |
| Sfrs18 | 66625 | NM_025669 | 1 | 1 | 1 | 1 | 0 | 0 | 1 | 1 | 6 |
| Urb2 | 382038 | NM_001029876 | 1 | 1 | 1 | 1 | 0 | 1 | 0 | 1 | 6 |
| BC051019 | 57355 | NM_001040700 | 1 | 1 | 1 | 1 | 0 | 1 | 0 | 1 | 6 |
| Kctd7 | 212919 | NM_172509 | 1 | 1 | 1 | 0 | 0 | 1 | 1 | 1 | 6 |
| Slc16a9 | 66859 | NM_025807 | 1 | 1 | 1 | 0 | 0 | 1 | 1 | 1 | 6 |
| Rbm41 | 237073 | NM_001172147 | 1 | 1 | 1 | 1 | 0 | 0 | 1 | 1 | 6 |
| Mgat5b | 268510 | NM_172948 | 1 | 1 | 1 | 1 | 0 | 1 | 0 | 1 | 6 |
| Hmgn3 | 94353 | NM_026122 | 1 | 1 | 1 | 1 | 0 | 0 | 1 | 1 | 6 |
| Zfp259 | 22687 | NM_011752 | 1 | 1 | 1 | 0 | 0 | 1 | 1 | 1 | 6 |
| Pgm1 | 66681 | NM_025700 | 1 | 1 | 1 | 1 | 0 | 1 | 0 | 1 | 6 |
| Slc7a6 | 330836 | NM_178798 | 1 | 1 | 1 | 0 | 0 | 1 | 1 | 1 | 6 |
| Cblb | 208650 | NM_001033238 | 1 | 1 | 1 | 1 | 0 | 1 | 0 | 1 | 6 |
| Trps1 | 83925 | NM_032000 | 1 | 1 | 1 | 0 | 0 | 1 | 1 | 1 | 6 |
| Diras2 | 68203 | NM_001024474 | 1 | 1 | 1 | 0 | 0 | 1 | 1 | 1 | 6 |
| Jdp2 | 81703 | NM_030887 | 1 | 1 | 1 | 1 | 0 | 1 | 0 | 1 | 6 |
| Ndst1 | 15531 | NM_008306 | 1 | 1 | 1 | 0 | 0 | 1 | 1 | 1 | 6 |
| Aak1 | 269774 | NM_001040106 | 1 | 1 | 1 | 1 | 0 | 0 | 1 | 1 | 6 |
| Acad9 | 229211 | NM_172678 | 1 | 1 | 1 | 1 | 0 | 1 | 0 | 1 | 6 |
| 9130023H24Rik | 100043133 | NM_177001 | 1 | 1 | 1 | 0 | 0 | 1 | 1 | 1 | 6 |
| Nfasc | 269116 | NM_182716 | 1 | 1 | 1 | 1 | 0 | 0 | 1 | 1 | 6 |
| Cwf19l1 | 72502 | NM_001081077 | 1 | 1 | 1 | 0 | 0 | 1 | 1 | 1 | 6 |
| Mpp1 | 17524 | NM_008621 | 1 | 1 | 1 | 1 | 0 | 1 | 0 | 1 | 6 |
| Gosr2 | 56494 | NM_019650 | 1 | 1 | 1 | 0 | 0 | 1 | 1 | 1 | 6 |
| Hpse | 15442 | NM_152803 | 1 | 1 | 1 | 0 | 0 | 1 | 1 | 1 | 6 |
| Phf15 | 76901 | NM_199299 | 1 | 1 | 1 | 0 | 0 | 1 | 1 | 1 | 6 |
| Cse1l | 110750 | NM_023565 | 1 | 1 | 1 | 1 | 0 | 1 | 0 | 1 | 6 |
| Phf20 | 228829 | NM_172674 | 1 | 1 | 1 | 0 | 0 | 1 | 1 | 1 | 6 |
| Dnajb1 | 81489 | NM_018808 | 1 | 1 | 1 | 0 | 0 | 1 | 1 | 1 | 6 |
| Arhgef38 | 77669 | NM_029953 | 1 | 0 | 1 | 1 | 0 | 1 | 1 | 1 | 6 |
| Plekhh1 | 211945 | NM_181073 | 1 | 1 | 1 | 1 | 0 | 0 | 1 | 1 | 6 |
| Bach1 | 12013 | NM_007520 | 1 | 1 | 1 | 0 | 0 | 1 | 1 | 1 | 6 |
| Runx2 | 12393 | NM_001145920 | 1 | 1 | 1 | 1 | 0 | 0 | 1 | 1 | 6 |
| Tardbp | 230908 | NM_145556 | 1 | 1 | 1 | 0 | 0 | 1 | 1 | 1 | 6 |
| Smc6 | 67241 | NM_025695 | 1 | 1 | 1 | 0 | 0 | 1 | 1 | 1 | 6 |
| Lipm | 78753 | NM_023903 | 1 | 1 | 1 | 1 | 0 | 1 | 0 | 1 | 6 |
| Fam19a3 | 329731 | NM_183224 | 1 | 1 | 1 | 0 | 0 | 1 | 1 | 1 | 6 |
| Dhfr | 13361 | NM_010049 | 1 | 1 | 1 | 0 | 0 | 1 | 1 | 1 | 6 |
| Wnt7a | 22421 | NM_009527 | 1 | 1 | 1 | 1 | 0 | 1 | 0 | 1 | 6 |
| Srgap3 | 259302 | NM_080448 | 1 | 1 | 1 | 0 | 0 | 1 | 1 | 1 | 6 |
| Slc19a2 | 116914 | NM_054087 | 1 | 1 | 1 | 0 | 0 | 1 | 1 | 1 | 6 |
| Neurod2 | 18013 | NM_010895 | 1 | 1 | 1 | 1 | 0 | 1 | 0 | 1 | 6 |
| Smim7 | 66818 | NM_172396 | 1 | 1 | 1 | 1 | 0 | 1 | 0 | 1 | 6 |
| Ubqln4 | 94232 | NM_033526 | 1 | 1 | 1 | 0 | 0 | 1 | 1 | 1 | 6 |
| Ccng2 | 12452 | NM_007635 | 1 | 1 | 1 | 1 | 0 | 1 | 0 | 1 | 6 |
| Rcor2 | 104383 | NM_054048 | 1 | 1 | 1 | 1 | 0 | 1 | 0 | 1 | 6 |
| Zfp346 | 26919 | NM_012017 | 1 | 1 | 1 | 0 | 0 | 1 | 1 | 1 | 6 |
| Ubxn8 | 108159 | NM_178648 | 1 | 1 | 1 | 1 | 0 | 1 | 0 | 1 | 6 |
| Rab11b | 19326 | NM_008997 | 1 | 1 | 1 | 1 | 0 | 1 | 0 | 1 | 6 |
| Ankrd44 | 329154 | NM_001081433 | 1 | 1 | 1 | 0 | 0 | 1 | 1 | 1 | 6 |
| Ap1s3 | 252903 | NM_183027 | 1 | 1 | 1 | 1 | 0 | 1 | 0 | 1 | 6 |
| Psg29 | 114872 | NM_054064 | 1 | 1 | 1 | 0 | 0 | 1 | 1 | 1 | 6 |
| Gphb5 | 217674 | NM_175644 | 1 | 1 | 1 | 0 | 0 | 1 | 1 | 1 | 6 |
| Gadd45a | 13197 | NM_007836 | 1 | 1 | 1 | 1 | 0 | 1 | 0 | 1 | 6 |
| Tmtc4 | 70551 | NM_028651 | 1 | 1 | 1 | 0 | 0 | 1 | 1 | 1 | 6 |
| Zmat4 | 320158 | NM_177086 | 1 | 1 | 1 | 0 | 0 | 1 | 1 | 1 | 6 |
| Mab21l2 | 23937 | NM_011839 | 1 | 1 | 1 | 1 | 0 | 1 | 0 | 1 | 6 |
| E130309D14Rik | 432582 | NM_001013784 | 1 | 1 | 1 | 0 | 0 | 1 | 1 | 1 | 6 |
| Mlc1 | 170790 | NM_133241 | 1 | 1 | 1 | 1 | 0 | 1 | 0 | 1 | 6 |
| Ubxn10 | 212190 | NM_178671 | 1 | 1 | 1 | 0 | 0 | 1 | 1 | 1 | 6 |
| Herpud2 | 80517 | NM_020586 | 1 | 1 | 1 | 1 | 0 | 1 | 0 | 1 | 6 |
| Rpgrip1l | 244585 | NM_173431 | 1 | 1 | 1 | 1 | 0 | 1 | 0 | 1 | 6 |
| Unc5c | 22253 | NM_009472 | 1 | 1 | 1 | 0 | 0 | 1 | 1 | 1 | 6 |
| Prdx6b | 320769 | NM_177256 | 1 | 1 | 1 | 1 | 0 | 1 | 0 | 1 | 6 |
| Fnip1 | 216742 | NM_173753 | 1 | 1 | 1 | 1 | 0 | 1 | 0 | 1 | 6 |
| Zbtb4 | 75580 | NM_029348 | 1 | 1 | 1 | 0 | 0 | 1 | 1 | 1 | 6 |
| 8-Mar | 71779 | NM_027920 | 1 | 1 | 1 | 0 | 0 | 1 | 1 | 1 | 6 |
| Stk38l | 232533 | NM_172734 | 1 | 1 | 1 | 1 | 0 | 1 | 0 | 1 | 6 |
| Scamp2 | 24044 | NM_022813 | 1 | 1 | 1 | 0 | 0 | 1 | 1 | 1 | 6 |
| Erbb2ip | 59079 | NM_001005868 | 1 | 1 | 1 | 1 | 0 | 0 | 1 | 1 | 6 |
| Rwdd4a | 192174 | NM_203507 | 1 | 1 | 1 | 1 | 0 | 1 | 0 | 1 | 6 |
| Igfbp5 | 16011 | NM_010518 | 1 | 1 | 1 | 0 | 0 | 1 | 1 | 1 | 6 |
| Sart1 | 20227 | NM_016882 | 1 | 1 | 1 | 0 | 0 | 1 | 1 | 1 | 6 |
| Csnk1g3 | 70425 | NM_152809 | 1 | 1 | 1 | 1 | 0 | 1 | 0 | 1 | 6 |
| Lrit1 | 239037 | NM_146245 | 1 | 1 | 1 | 1 | 0 | 1 | 0 | 1 | 6 |
| Ppp3cb | 19056 | NM_008914 | 1 | 1 | 1 | 0 | 0 | 1 | 1 | 1 | 6 |
| Ptpn2 | 19255 | NM_001127177 | 1 | 1 | 1 | 1 | 0 | 0 | 1 | 1 | 6 |
| Klhl10 | 66720 | NM_025727 | 1 | 1 | 1 | 0 | 0 | 1 | 1 | 1 | 6 |
| Slc38a4 | 69354 | NM_027052 | 1 | 1 | 1 | 0 | 0 | 1 | 1 | 1 | 6 |
| Kcna2 | 16490 | NM_008417 | 1 | 1 | 1 | 1 | 0 | 1 | 0 | 1 | 6 |
| Zkscan8 | 93681 | NM_139141 | 1 | 1 | 1 | 0 | 0 | 1 | 1 | 1 | 6 |
| Otos | 260301 | NM_153114 | 1 | 1 | 1 | 0 | 0 | 1 | 1 | 1 | 6 |
| Spata16 | 70862 | NM_029150 | 1 | 1 | 1 | 1 | 0 | 1 | 0 | 1 | 6 |
| Stt3b | 68292 | NM_024222 | 1 | 1 | 1 | 0 | 0 | 1 | 1 | 1 | 6 |
| Cyp2c38 | 13097 | NM_010002 | 1 | 1 | 1 | 1 | 0 | 1 | 0 | 1 | 6 |
| Zdhhc5 | 228136 | NM_144887 | 1 | 1 | 1 | 1 | 0 | 1 | 0 | 1 | 6 |
| Exo1 | 26909 | NM_012012 | 1 | 1 | 1 | 1 | 0 | 1 | 0 | 1 | 6 |
| Mpzl3 | 319742 | NM_176993 | 1 | 1 | 1 | 1 | 0 | 1 | 0 | 1 | 6 |
| Ltn1 | 78913 | NM_001081068 | 1 | 1 | 1 | 1 | 0 | 1 | 0 | 1 | 6 |
| Nfatc2 | 18019 | NM_010899 | 1 | 1 | 1 | 0 | 0 | 1 | 1 | 1 | 6 |
| Dsc2 | 13506 | NM_013505 | 1 | 1 | 1 | 0 | 0 | 1 | 1 | 1 | 6 |
| Fam132a | 67389 | NM_026125 | 1 | 1 | 1 | 1 | 0 | 1 | 0 | 1 | 6 |
| Gcsh | 68133 | NM_026572 | 1 | 1 | 1 | 1 | 0 | 1 | 0 | 1 | 6 |
| Zmym2 | 76007 | NM_029498 | 1 | 1 | 1 | 1 | 0 | 1 | 0 | 1 | 6 |
| Zfp148 | 22661 | NM_011749 | 1 | 1 | 1 | 0 | 0 | 1 | 1 | 1 | 6 |
| G2e3 | 217558 | NM_001167963 | 1 | 1 | 1 | 1 | 0 | 0 | 1 | 1 | 6 |
| Kank4 | 242553 | NM_172872 | 1 | 1 | 1 | 1 | 0 | 1 | 0 | 1 | 6 |
| Pcdhb16 | 93887 | NM_053141 | 1 | 1 | 1 | 1 | 0 | 1 | 0 | 1 | 6 |
| Gm5938 | 546335 | NM_001085534 | 1 | 1 | 1 | 0 | 0 | 1 | 1 | 1 | 6 |
| Srsf6 | 67996 | NM_026499 | 1 | 1 | 1 | 1 | 0 | 1 | 0 | 1 | 6 |
| Rrm2b | 382985 | NM_199476 | 1 | 1 | 1 | 0 | 0 | 1 | 1 | 1 | 6 |
| Slc25a46 | 67453 | NM_026165 | 1 | 1 | 1 | 0 | 0 | 1 | 1 | 1 | 6 |
| Lphn2 | 99633 | NM_001081298 | 1 | 1 | 1 | 0 | 0 | 1 | 1 | 1 | 6 |
| Fam98a | 72722 | NM_133747 | 1 | 1 | 1 | 0 | 0 | 1 | 1 | 1 | 6 |
| Ugcg | 22234 | NM_011673 | 1 | 1 | 1 | 0 | 0 | 1 | 1 | 1 | 6 |
| Tubgcp5 | 233276 | NM_146190 | 1 | 1 | 1 | 1 | 0 | 1 | 0 | 1 | 6 |
| Bmpr1a | 12166 | NM_009758 | 1 | 1 | 1 | 0 | 0 | 1 | 1 | 1 | 6 |
| Rtp1 | 239766 | NM_001004151 | 1 | 1 | 1 | 1 | 0 | 1 | 0 | 1 | 6 |
| Tsn | 22099 | NM_011650 | 1 | 1 | 1 | 1 | 0 | 1 | 0 | 1 | 6 |
| Ssr1 | 107513 | NM_025965 | 1 | 1 | 1 | 1 | 0 | 1 | 0 | 1 | 6 |
| B4galt5 | 56336 | NM_019835 | 1 | 1 | 1 | 0 | 0 | 1 | 1 | 1 | 6 |
| Fam210b | 67017 | NM_025912 | 1 | 1 | 1 | 1 | 0 | 1 | 0 | 1 | 6 |
| Limch1 | 77569 | NM_001001980 | 1 | 1 | 1 | 0 | 0 | 1 | 1 | 1 | 6 |
| Col8a1 | 12837 | NM_007739 | 1 | 1 | 1 | 0 | 0 | 1 | 1 | 1 | 6 |
| Tars2 | 71807 | NM_001163619 | 1 | 1 | 1 | 1 | 0 | 0 | 1 | 1 | 6 |
| Med1 | 19014 | NM_001080118 | 1 | 1 | 1 | 1 | 0 | 0 | 1 | 1 | 6 |
| Rgs7bp | 52882 | NM_029879 | 1 | 1 | 1 | 0 | 0 | 1 | 1 | 1 | 6 |
| Zfp704 | 170753 | NM_133218 | 1 | 1 | 1 | 0 | 0 | 1 | 1 | 1 | 6 |
| Appl2 | 216190 | NM_145220 | 1 | 1 | 1 | 1 | 0 | 1 | 0 | 1 | 6 |
| Serpinb6b | 20708 | NM_011454 | 1 | 1 | 1 | 0 | 0 | 1 | 1 | 1 | 6 |
| Lcp2 | 16822 | NM_010696 | 1 | 1 | 1 | 0 | 0 | 1 | 1 | 1 | 6 |
| Baz2a | 116848 | NM_054078 | 1 | 1 | 1 | 1 | 0 | 1 | 0 | 1 | 6 |
| Alcam | 11658 | NM_009655 | 1 | 1 | 1 | 0 | 0 | 1 | 1 | 1 | 6 |
| Naa50 | 72117 | NM_028108 | 1 | 1 | 1 | 0 | 0 | 1 | 1 | 1 | 6 |
| Stk40 | 74178 | NM_001145827 | 1 | 1 | 1 | 1 | 0 | 0 | 1 | 1 | 6 |
| Glce | 93683 | NM_033320 | 1 | 1 | 1 | 0 | 0 | 1 | 1 | 1 | 6 |
| Tfap2c | 21420 | NM_009335 | 1 | 1 | 1 | 0 | 0 | 1 | 1 | 1 | 6 |
| Tjp1 | 21872 | NM_009386 | 1 | 1 | 1 | 1 | 0 | 1 | 0 | 1 | 6 |
| Cntnap2 | 66797 | NM_001004357 | 1 | 1 | 1 | 1 | 0 | 0 | 1 | 1 | 6 |
| Pls1 | 102502 | NM_001033210 | 1 | 1 | 1 | 0 | 0 | 1 | 1 | 1 | 6 |
| Fgf7 | 14178 | NM_008008 | 1 | 1 | 1 | 1 | 0 | 1 | 0 | 1 | 6 |
| Sos1 | 20662 | NM_009231 | 1 | 1 | 1 | 0 | 0 | 1 | 1 | 1 | 6 |
| Slc35a5 | 74102 | NM_028756 | 1 | 1 | 1 | 1 | 0 | 1 | 0 | 1 | 6 |
| Mapk10 | 26414 | NM_009158 | 1 | 1 | 1 | 1 | 0 | 0 | 1 | 1 | 6 |
| E2f6 | 50496 | NM_033270 | 1 | 1 | 1 | 0 | 0 | 1 | 1 | 1 | 6 |
| Spred1 | 114715 | NM_033524 | 1 | 1 | 1 | 0 | 0 | 1 | 1 | 1 | 6 |
| Cpxcr1 | 382239 | NM_001033471 | 1 | 1 | 1 | 1 | 0 | 1 | 0 | 1 | 6 |
| Cpne8 | 66871 | NM_025815 | 1 | 1 | 1 | 0 | 0 | 1 | 1 | 1 | 6 |
| Cst6 | 73720 | NM_028623 | 1 | 1 | 1 | 0 | 0 | 1 | 1 | 1 | 6 |
| Baz1a | 217578 | NM_013815 | 1 | 1 | 1 | 1 | 0 | 0 | 1 | 1 | 6 |
| Itgav | 16410 | NM_008402 | 1 | 1 | 1 | 0 | 0 | 1 | 1 | 1 | 6 |
| Tmem260 | 218989 | NM_172600 | 1 | 1 | 1 | 0 | 0 | 1 | 1 | 1 | 6 |
| Pcdhb18 | 93889 | NM_053143 | 1 | 1 | 1 | 1 | 0 | 1 | 0 | 1 | 6 |
| Sash3 | 74131 | NM_028773 | 1 | 1 | 1 | 0 | 0 | 1 | 1 | 1 | 6 |
| Mtap | 66902 | NM_024433 | 1 | 1 | 1 | 1 | 0 | 1 | 0 | 1 | 6 |
| Lemd3 | 380664 | NM_001081193 | 1 | 1 | 1 | 0 | 0 | 1 | 1 | 1 | 6 |
| Mab21l1 | 17116 | NM_010750 | 1 | 1 | 1 | 1 | 0 | 1 | 0 | 1 | 6 |
| Zfp523 | 224656 | NM_172617 | 1 | 1 | 1 | 0 | 0 | 1 | 1 | 1 | 6 |
| Inpp5a | 212111 | NM_001127363 | 1 | 1 | 1 | 1 | 0 | 0 | 1 | 1 | 6 |
| Htr2c | 15560 | NM_008312 | 1 | 1 | 1 | 0 | 0 | 1 | 1 | 1 | 6 |
| Tns4 | 217169 | NM_172564 | 1 | 1 | 1 | 0 | 0 | 1 | 1 | 1 | 6 |
| Il1rap | 16180 | NM_008364 | 1 | 1 | 1 | 0 | 0 | 1 | 1 | 1 | 6 |
| Rnf185 | 193670 | NM_145355 | 1 | 1 | 1 | 0 | 0 | 1 | 1 | 1 | 6 |
| Mysm1 | 320713 | NM_177239 | 1 | 1 | 1 | 1 | 0 | 1 | 0 | 1 | 6 |
| Btaf1 | 107182 | NM_001080706 | 1 | 1 | 1 | 0 | 0 | 1 | 1 | 1 | 6 |
| Fam76b | 72826 | NM_176836 | 1 | 1 | 1 | 1 | 0 | 1 | 0 | 1 | 6 |
| Scarb1 | 20778 | NM_016741 | 1 | 1 | 1 | 1 | 0 | 1 | 0 | 1 | 6 |
| Col11a1 | 12814 | NM_007729 | 1 | 1 | 1 | 0 | 0 | 1 | 1 | 1 | 6 |
| Capn2 | 12334 | NM_009794 | 1 | 1 | 1 | 0 | 0 | 1 | 1 | 1 | 6 |
| Gjb3 | 14620 | NM_001160012 | 1 | 1 | 1 | 1 | 0 | 0 | 1 | 1 | 6 |
| Dock4 | 238130 | NM_172803 | 1 | 1 | 1 | 0 | 0 | 1 | 1 | 1 | 6 |
| Gmnc | 239789 | NM_001013761 | 1 | 1 | 1 | 1 | 0 | 1 | 0 | 1 | 6 |
| Rreb1 | 68750 | NM_001177869 | 1 | 1 | 1 | 1 | 0 | 0 | 1 | 1 | 6 |
| Lgr4 | 107515 | NM_172671 | 1 | 1 | 1 | 1 | 0 | 1 | 0 | 1 | 6 |
| Ppm1e | 320472 | NM_177167 | 1 | 1 | 1 | 0 | 0 | 1 | 1 | 1 | 6 |
| 3-Sep | 24050 | NM_011889 | 1 | 1 | 1 | 1 | 0 | 1 | 0 | 1 | 6 |
| Osbpl6 | 99031 | NM_145525 | 1 | 1 | 1 | 0 | 0 | 1 | 1 | 1 | 6 |
| Zfp568 | 243905 | NM_001167872 | 1 | 1 | 1 | 1 | 0 | 0 | 1 | 1 | 6 |
| Gm5615 | 434396 | NM_001033783 | 1 | 1 | 1 | 0 | 0 | 1 | 1 | 1 | 6 |
| Cyyr1 | 224405 | NM_144853 | 1 | 1 | 1 | 0 | 0 | 1 | 1 | 1 | 6 |
| Pdlim3 | 53318 | NM_016798 | 1 | 1 | 1 | 1 | 0 | 1 | 0 | 1 | 6 |
| Pafah1b2 | 18475 | NM_008775 | 1 | 1 | 1 | 0 | 0 | 1 | 1 | 1 | 6 |
| Dnal1 | 105000 | NM_028821 | 1 | 1 | 1 | 0 | 0 | 1 | 1 | 1 | 6 |
| Olfr613 | 259104 | NM_147100 | 1 | 1 | 1 | 0 | 0 | 1 | 1 | 1 | 6 |
| Thpo | 21832 | NM_009379 | 1 | 1 | 1 | 0 | 0 | 1 | 1 | 1 | 6 |
| Kcnmb2 | 72413 | NM_028231 | 1 | 1 | 1 | 1 | 0 | 1 | 0 | 1 | 6 |
| Yipf6 | 77929 | NM_207633 | 1 | 1 | 1 | 1 | 0 | 1 | 0 | 1 | 6 |
| N4bp2l2 | 381695 | NM_201369 | 1 | 1 | 1 | 1 | 0 | 1 | 0 | 1 | 6 |
| Arhgef18 | 102098 | NM_133962 | 1 | 1 | 1 | 0 | 0 | 1 | 1 | 1 | 6 |
| Itga9 | 104099 | NM_133721 | 1 | 1 | 1 | 1 | 0 | 0 | 1 | 1 | 6 |
| Seh1l | 72124 | NM_001039088 | 1 | 1 | 1 | 1 | 0 | 1 | 0 | 1 | 6 |
| Fmnl2 | 71409 | NM_172409 | 1 | 1 | 1 | 1 | 0 | 1 | 0 | 1 | 6 |
| Kcnc3 | 16504 | NM_008422 | 1 | 1 | 1 | 0 | 0 | 1 | 1 | 1 | 6 |
| Dmrt2 | 226049 | NM_145831 | 1 | 1 | 1 | 1 | 0 | 1 | 0 | 1 | 6 |
| Megf9 | 230316 | NM_172694 | 1 | 1 | 1 | 1 | 0 | 1 | 0 | 1 | 6 |
| Arid4b | 94246 | NM_194262 | 1 | 1 | 1 | 1 | 0 | 0 | 1 | 1 | 6 |
| Slc25a5 | 11740 | NM_007451 | 1 | 1 | 1 | 1 | 0 | 1 | 0 | 1 | 6 |
| Slc17a4 | 319848 | NM_177016 | 1 | 1 | 1 | 1 | 0 | 1 | 0 | 1 | 6 |
| Itga4 | 16401 | NM_010576 | 1 | 1 | 1 | 0 | 0 | 1 | 1 | 1 | 6 |
| Msantd2 | 235184 | NM_146222 | 1 | 1 | 1 | 1 | 0 | 1 | 0 | 1 | 6 |
| Padi2 | 18600 | NM_008812 | 1 | 1 | 1 | 0 | 0 | 1 | 1 | 1 | 6 |
| Pcdhb19 | 93890 | NM_053144 | 1 | 1 | 1 | 1 | 0 | 1 | 0 | 1 | 6 |
| Ddx3x | 13205 | NM_010028 | 1 | 1 | 1 | 1 | 0 | 1 | 0 | 1 | 6 |
| Zfp217 | 228913 | NM_001033299 | 1 | 1 | 1 | 1 | 0 | 1 | 0 | 1 | 6 |
| Setd7 | 73251 | NM_080793 | 1 | 1 | 1 | 0 | 0 | 1 | 1 | 1 | 6 |
| Rlim | 19820 | NM_011276 | 1 | 1 | 1 | 0 | 0 | 1 | 1 | 1 | 6 |
| Fam3c | 27999 | NM_138587 | 1 | 1 | 1 | 0 | 0 | 1 | 1 | 1 | 6 |
| Strbp | 20744 | NM_009261 | 1 | 1 | 1 | 1 | 0 | 0 | 1 | 1 | 6 |
| Glis2 | 83396 | NM_031184 | 1 | 1 | 1 | 1 | 0 | 1 | 0 | 1 | 6 |
| Anapc13 | 69010 | NM_181394 | 1 | 1 | 1 | 0 | 0 | 1 | 1 | 1 | 6 |
| Atp1b1 | 11931 | NM_009721 | 1 | 1 | 1 | 1 | 0 | 1 | 0 | 1 | 6 |
| Zc3h11a | 70579 | NM_144530 | 1 | 1 | 1 | 0 | 0 | 1 | 1 | 1 | 6 |
| Reck | 53614 | NM_016678 | 1 | 1 | 1 | 1 | 0 | 1 | 0 | 1 | 6 |
| Trhr | 22045 | NM_013696 | 1 | 1 | 1 | 0 | 0 | 1 | 1 | 1 | 6 |
| Serp1 | 28146 | NM_030685 | 1 | 1 | 1 | 1 | 0 | 1 | 0 | 1 | 6 |
| Pptc7 | 320717 | NM_177242 | 1 | 1 | 1 | 0 | 0 | 1 | 1 | 1 | 6 |
| Ccdc90b | 66365 | NM_025515 | 1 | 1 | 1 | 1 | 0 | 1 | 0 | 1 | 6 |
| Foxk1 | 17425 | NM_199068 | 1 | 1 | 1 | 0 | 0 | 1 | 1 | 1 | 6 |
| Papss2 | 23972 | NM_011864 | 1 | 1 | 1 | 0 | 0 | 1 | 1 | 1 | 6 |
| Ttc14 | 67120 | NM_025978 | 1 | 1 | 1 | 0 | 0 | 1 | 1 | 1 | 6 |
| Mettl9 | 59052 | NM_021554 | 1 | 1 | 1 | 1 | 0 | 1 | 0 | 1 | 6 |
| Phtf2 | 68770 | NM_172992 | 1 | 1 | 1 | 1 | 0 | 1 | 0 | 1 | 6 |
| Clec5a | 23845 | NM_001038604 | 1 | 1 | 1 | 1 | 0 | 0 | 1 | 1 | 6 |
| Oxr1 | 170719 | NM_001130166 | 1 | 1 | 1 | 1 | 0 | 0 | 1 | 1 | 6 |
| Mbtps2 | 270669 | NM_172307 | 1 | 0 | 1 | 1 | 0 | 1 | 1 | 1 | 6 |
| Papd7 | 210106 | NM_198600 | 1 | 1 | 1 | 1 | 0 | 1 | 0 | 1 | 6 |
| Arf4 | 11843 | NM_007479 | 1 | 1 | 1 | 1 | 0 | 1 | 0 | 1 | 6 |
| Ahcyl1 | 229709 | NM_145542 | 1 | 1 | 1 | 1 | 0 | 1 | 0 | 1 | 6 |
| Tbc1d19 | 67249 | NM_144517 | 1 | 1 | 1 | 1 | 0 | 1 | 0 | 1 | 6 |
| Spty2d1 | 101685 | NM_175318 | 1 | 1 | 1 | 0 | 0 | 1 | 1 | 1 | 6 |
| Rictor | 78757 | NM_030168 | 1 | 1 | 1 | 0 | 0 | 1 | 1 | 1 | 6 |
| Ikzf3 | 22780 | NM_011771 | 1 | 1 | 1 | 0 | 0 | 1 | 1 | 1 | 6 |
| Dhx40 | 67487 | NM_026191 | 1 | 1 | 1 | 0 | 0 | 1 | 1 | 1 | 6 |
| Nova1 | 664883 | NM_021361 | 1 | 1 | 1 | 1 | 0 | 0 | 1 | 1 | 6 |
| Med17 | 234959 | NM_144933 | 1 | 1 | 1 | 0 | 0 | 1 | 1 | 1 | 6 |
| Gabra1 | 14394 | NM_010250 | 1 | 1 | 1 | 0 | 0 | 1 | 1 | 1 | 6 |
| Chm | 12662 | NM_018818 | 1 | 1 | 1 | 0 | 0 | 1 | 1 | 1 | 6 |
| Hey2 | 15214 | NM_013904 | 1 | 1 | 1 | 0 | 0 | 1 | 1 | 1 | 6 |
| Vegfc | 22341 | NM_009506 | 1 | 1 | 1 | 0 | 0 | 1 | 1 | 1 | 6 |
| Zbtb34 | 241311 | NM_001085507 | 1 | 1 | 1 | 0 | 0 | 1 | 1 | 1 | 6 |
| Lmtk2 | 231876 | NM_001081109 | 1 | 1 | 1 | 1 | 0 | 1 | 0 | 1 | 6 |
| Slc1a5 | 20514 | NM_009201 | 1 | 1 | 1 | 1 | 0 | 1 | 0 | 1 | 6 |
| Fam135a | 68187 | NM_026604 | 1 | 1 | 1 | 0 | 0 | 1 | 1 | 1 | 6 |
| Necab3 | 56846 | NM_021546 | 1 | 1 | 1 | 1 | 0 | 1 | 0 | 1 | 6 |
| Ccdc38 | 237465 | NM_175488 | 1 | 1 | 1 | 1 | 0 | 1 | 0 | 1 | 6 |
| Msrb3 | 320183 | NM_177092 | 1 | 1 | 1 | 0 | 0 | 1 | 1 | 1 | 6 |
| Desi1 | 28075 | NM_134095 | 1 | 1 | 1 | 0 | 0 | 1 | 1 | 1 | 6 |
| Ugp2 | 216558 | NM_139297 | 1 | 1 | 1 | 1 | 0 | 1 | 0 | 1 | 6 |
| Ephx3 | 71932 | NM_001033163 | 1 | 1 | 1 | 0 | 0 | 1 | 1 | 1 | 6 |
| Ss18 | 268996 | NM_009280 | 1 | 1 | 1 | 1 | 0 | 1 | 0 | 1 | 6 |
| Cacna1i | 239556 | NM_001044308 | 1 | 1 | 1 | 0 | 0 | 1 | 1 | 1 | 6 |
| Agpat3 | 28169 | NM_053014 | 1 | 1 | 1 | 0 | 0 | 1 | 1 | 1 | 6 |
| Mapkapk2 | 17164 | NM_008551 | 1 | 1 | 1 | 1 | 0 | 1 | 0 | 1 | 6 |
| Map3k14 | 53859 | NM_016896 | 1 | 1 | 1 | 0 | 0 | 1 | 1 | 1 | 6 |
| Gulp1 | 70676 | NM_028450 | 1 | 1 | 1 | 1 | 0 | 1 | 0 | 1 | 6 |
| Tmem55a | 72519 | NM_028264 | 1 | 1 | 1 | 1 | 0 | 1 | 0 | 1 | 6 |
| Col4a3 | 12828 | NM_007734 | 1 | 1 | 1 | 0 | 0 | 1 | 1 | 1 | 6 |
| Gltp | 56356 | NM_019821 | 1 | 1 | 1 | 0 | 0 | 1 | 1 | 1 | 6 |
| Sgcg | 24053 | NM_011892 | 1 | 1 | 1 | 1 | 0 | 1 | 0 | 1 | 6 |
| Prickle1 | 106042 | NM_001033217 | 1 | 1 | 1 | 0 | 0 | 1 | 1 | 1 | 6 |
| Rell2 | 225392 | NM_153793 | 1 | 1 | 1 | 1 | 0 | 1 | 0 | 1 | 6 |
| Rsbn1 | 229675 | NM_172684 | 1 | 1 | 1 | 1 | 0 | 1 | 0 | 1 | 6 |
| Nt5dc2 | 70021 | NM_027289 | 1 | 1 | 1 | 1 | 0 | 1 | 0 | 1 | 6 |
| Axin1 | 12005 | NM_009733 | 1 | 1 | 1 | 0 | 0 | 1 | 1 | 1 | 6 |
| Rhag | 19743 | NM_011269 | 1 | 1 | 1 | 1 | 0 | 1 | 0 | 1 | 6 |
| Nsg1 | 18196 | NM_010942 | 1 | 1 | 1 | 0 | 0 | 1 | 1 | 1 | 6 |
| Zscan20 | 269585 | NM_177758 | 1 | 1 | 1 | 1 | 0 | 1 | 0 | 1 | 6 |
| Dmxl1 | 240283 | NM_001081371 | 1 | 1 | 1 | 0 | 0 | 1 | 1 | 1 | 6 |
| Wif1 | 24117 | NM_011915 | 1 | 1 | 1 | 1 | 0 | 1 | 0 | 1 | 6 |
| Aldh1a3 | 56847 | NM_053080 | 1 | 1 | 1 | 0 | 0 | 1 | 1 | 1 | 6 |
| Maml1 | 103806 | NM_175334 | 1 | 1 | 1 | 0 | 0 | 1 | 1 | 1 | 6 |
| Unc79 | 217843 | NM_001081017 | 1 | 1 | 1 | 0 | 0 | 1 | 1 | 1 | 6 |
| Nck2 | 17974 | NM_010879 | 1 | 1 | 1 | 0 | 0 | 1 | 1 | 1 | 6 |
| 2900011O08Rik | 67254 | NM_144518 | 1 | 1 | 1 | 0 | 0 | 1 | 1 | 1 | 6 |
| Zfp872 | 619310 | NM_001033813 | 1 | 1 | 1 | 1 | 0 | 1 | 0 | 1 | 6 |
| Zfp459 | 328274 | NM_177811 | 1 | 1 | 1 | 0 | 0 | 1 | 1 | 1 | 6 |
| Shisa3 | 330096 | NM_001033415 | 1 | 1 | 1 | 1 | 0 | 1 | 0 | 1 | 6 |
| Kcnd2 | 16508 | NM_019697 | 1 | 1 | 1 | 0 | 0 | 1 | 1 | 1 | 6 |
| Brd1 | 223770 | NM_001033274 | 1 | 1 | 1 | 0 | 0 | 1 | 1 | 1 | 6 |
| Tbx18 | 76365 | NM_023814 | 1 | 1 | 1 | 1 | 0 | 1 | 0 | 1 | 6 |
| Tvp23b | 67510 | NM_026210 | 1 | 1 | 1 | 1 | 0 | 1 | 0 | 1 | 6 |
| Prkacb | 18749 | NM_011100 | 1 | 1 | 1 | 0 | 0 | 1 | 1 | 1 | 6 |
| Kcnc2 | 268345 | NM_001025581 | 1 | 1 | 1 | 0 | 0 | 1 | 1 | 1 | 6 |
| Kcnj6 | 16522 | NM_001025590 | 1 | 1 | 1 | 1 | 0 | 1 | 0 | 1 | 6 |
| Nfe2l2 | 18024 | NM_010902 | 1 | 1 | 1 | 1 | 0 | 1 | 0 | 1 | 6 |
| Hnrnpdl | 50926 | NM_016690 | 1 | 1 | 1 | 1 | 0 | 1 | 0 | 1 | 6 |
| Obox3 | 246791 | NM_145707 | 1 | 1 | 1 | 1 | 0 | 1 | 0 | 1 | 6 |
| Rnf150 | 330812 | NM_177378 | 1 | 1 | 1 | 0 | 0 | 1 | 1 | 1 | 6 |
| Pah | 18478 | NM_008777 | 1 | 1 | 1 | 1 | 0 | 1 | 0 | 1 | 6 |
| Fam167a | 219148 | NM_177628 | 1 | 1 | 1 | 0 | 0 | 1 | 1 | 1 | 6 |
| Zfp280d | 235469 | NM_146224 | 1 | 1 | 1 | 1 | 0 | 1 | 0 | 1 | 6 |
| Btf3l4 | 70533 | NM_027453 | 1 | 1 | 1 | 1 | 0 | 1 | 0 | 1 | 6 |
| Gramd3 | 107022 | NM_026240 | 1 | 1 | 1 | 1 | 0 | 1 | 0 | 1 | 6 |
| Enpp4 | 224794 | NM_199016 | 1 | 1 | 1 | 0 | 0 | 1 | 1 | 1 | 6 |
| Sipa1l2 | 244668 | NM_001081337 | 1 | 1 | 1 | 0 | 0 | 1 | 1 | 1 | 6 |
| Limd1 | 29806 | NM_013860 | 1 | 1 | 1 | 1 | 0 | 1 | 0 | 1 | 6 |
| Apc | 11789 | NM_007462 | 1 | 1 | 1 | 0 | 0 | 1 | 1 | 1 | 6 |
| Pno1 | 66249 | NM_025443 | 1 | 1 | 1 | 0 | 0 | 1 | 1 | 1 | 6 |
| Kdm5b | 75605 | NM_152895 | 1 | 1 | 1 | 1 | 0 | 1 | 0 | 1 | 6 |
| Gm13119 | 433779 | NM_001034101 | 1 | 1 | 1 | 0 | 0 | 1 | 1 | 1 | 6 |
| Swt1 | 66875 | NM_025819 | 1 | 1 | 1 | 1 | 0 | 1 | 0 | 1 | 6 |
| Tmem218 | 66279 | NM_025464 | 1 | 1 | 1 | 1 | 0 | 1 | 0 | 1 | 6 |
| Rhox2c | 100039948 | NM_001099318 | 1 | 1 | 1 | 0 | 0 | 1 | 1 | 1 | 6 |
| Plcb1 | 18795 | NM_019677 | 1 | 1 | 1 | 1 | 0 | 0 | 1 | 1 | 6 |
| Onecut2 | 225631 | NM_194268 | 1 | 1 | 1 | 0 | 0 | 1 | 1 | 1 | 6 |
| Tcf12 | 21406 | NM_011544 | 1 | 1 | 1 | 0 | 0 | 1 | 1 | 1 | 6 |
| Zc3h6 | 78751 | NM_178404 | 1 | 1 | 1 | 1 | 0 | 1 | 0 | 1 | 6 |
| Zbtb7b | 22724 | NM_009565 | 1 | 1 | 1 | 0 | 0 | 1 | 1 | 1 | 6 |
| Akap2 | 11641 | NM_001035532 | 1 | 1 | 1 | 1 | 0 | 0 | 1 | 1 | 6 |
| Cxadr | 13052 | NM_001025192 | 1 | 1 | 1 | 1 | 0 | 1 | 0 | 1 | 6 |
| Synj1 | 104015 | NM_001164483 | 1 | 1 | 1 | 1 | 0 | 0 | 1 | 1 | 6 |
| Kat2b | 18519 | NM_020005 | 1 | 1 | 1 | 1 | 0 | 1 | 0 | 1 | 6 |
| Fam168a | 319604 | NM_178764 | 1 | 1 | 1 | 1 | 0 | 1 | 0 | 1 | 6 |
| Desi2 | 78825 | NM_024282 | 1 | 1 | 1 | 0 | 0 | 1 | 1 | 1 | 6 |
| Zfp326 | 54367 | NM_018759 | 1 | 0 | 1 | 1 | 0 | 1 | 1 | 1 | 6 |
| Eaf1 | 74427 | NM_028932 | 1 | 1 | 1 | 0 | 0 | 1 | 1 | 1 | 6 |
| Med14 | 26896 | NM_001048208 | 1 | 1 | 1 | 1 | 0 | 1 | 0 | 1 | 6 |
| Hspa12a | 73442 | NM_175199 | 1 | 1 | 1 | 0 | 0 | 1 | 1 | 1 | 6 |
| Dr1 | 13486 | NM_026106 | 1 | 1 | 1 | 0 | 0 | 1 | 1 | 1 | 6 |
| Pitpnm2 | 19679 | NM_011256 | 1 | 1 | 1 | 0 | 0 | 1 | 1 | 1 | 6 |
| Asph | 65973 | NM_023066 | 1 | 1 | 1 | 0 | 0 | 1 | 1 | 1 | 6 |
| Ubxn2a | 217379 | NM_145441 | 1 | 1 | 1 | 1 | 0 | 1 | 0 | 1 | 6 |
| Grb7 | 14786 | NM_010346 | 1 | 1 | 1 | 1 | 0 | 1 | 0 | 1 | 6 |
| Qk | 19317 | NM_021881 | 1 | 1 | 1 | 0 | 0 | 1 | 1 | 1 | 6 |
| Pkd2l1 | 329064 | NM_181422 | 1 | 1 | 1 | 1 | 0 | 1 | 0 | 1 | 6 |
| Lix1 | 66643 | NM_025681 | 1 | 1 | 1 | 1 | 0 | 1 | 0 | 1 | 6 |
| Fam210a | 108654 | NM_153794 | 1 | 1 | 1 | 0 | 0 | 1 | 1 | 1 | 6 |
| Myo1c | 17913 | NM_001080774 | 1 | 1 | 1 | 1 | 0 | 0 | 1 | 1 | 6 |
| Ncapg2 | 76044 | NM_133762 | 1 | 1 | 1 | 0 | 0 | 1 | 1 | 1 | 6 |
| Gad2 | 14417 | NM_008078 | 1 | 1 | 1 | 0 | 0 | 1 | 1 | 1 | 6 |
| Ncln | 103425 | NM_134009 | 1 | 1 | 1 | 0 | 0 | 1 | 1 | 1 | 6 |
| Ercc6 | 319955 | NM_001081221 | 1 | 1 | 1 | 0 | 0 | 1 | 1 | 1 | 6 |
| Fam126b | 213056 | NM_172513 | 1 | 1 | 1 | 1 | 0 | 1 | 0 | 1 | 6 |
| Gpr137b | 83924 | NM_031999 | 1 | 1 | 1 | 1 | 0 | 1 | 0 | 1 | 6 |
| Prtg | 235472 | NM_175485 | 1 | 1 | 1 | 0 | 0 | 1 | 1 | 1 | 6 |
| Ankrd12 | 106585 | NM_001025572 | 1 | 1 | 1 | 1 | 0 | 1 | 0 | 1 | 6 |
| Chordc1 | 66917 | NM_025844 | 1 | 1 | 1 | 1 | 0 | 1 | 0 | 1 | 6 |
| Dmrt3 | 240590 | NM_177360 | 1 | 1 | 1 | 1 | 0 | 1 | 0 | 1 | 6 |
| Dram1 | 71712 | NM_027878 | 1 | 1 | 1 | 1 | 0 | 1 | 0 | 1 | 6 |
| 8030462N17Rik | 212163 | NM_178670 | 1 | 1 | 1 | 1 | 0 | 1 | 0 | 1 | 6 |
| Epha7 | 13841 | NM_010141 | 1 | 1 | 1 | 1 | 0 | 1 | 0 | 1 | 6 |
| Tbc1d1 | 57915 | NM_019636 | 1 | 1 | 1 | 1 | 0 | 1 | 0 | 1 | 6 |
| Gm5464 | 432870 | NM_001034881 | 1 | 1 | 1 | 0 | 0 | 1 | 1 | 1 | 6 |
| Psmc6 | 67089 | NM_025959 | 1 | 1 | 1 | 1 | 0 | 1 | 0 | 1 | 6 |
| Emc8 | 18117 | NM_010926 | 1 | 1 | 1 | 0 | 0 | 1 | 1 | 1 | 6 |
| Ssbp2 | 66970 | NM_024272 | 1 | 1 | 1 | 0 | 0 | 1 | 1 | 1 | 6 |
| Gjc1 | 14615 | NM_008122 | 1 | 1 | 1 | 0 | 0 | 1 | 1 | 1 | 6 |
| Map3k1 | 26401 | NM_011945 | 1 | 1 | 1 | 0 | 0 | 1 | 1 | 1 | 6 |
| Lurap1l | 52829 | NM_026821 | 1 | 1 | 1 | 1 | 0 | 1 | 0 | 1 | 6 |
| Ryr2 | 20191 | NM_023868 | 1 | 1 | 1 | 0 | 0 | 1 | 1 | 1 | 6 |
| Dnajb5 | 56323 | NM_019874 | 1 | 1 | 1 | 0 | 0 | 1 | 1 | 1 | 6 |
| Bhlhe22 | 59058 | NM_021560 | 1 | 1 | 1 | 1 | 0 | 1 | 0 | 1 | 6 |
| Casp9 | 12371 | NM_015733 | 1 | 1 | 1 | 1 | 0 | 1 | 0 | 1 | 6 |
| Ptgfrn | 19221 | NM_011197 | 1 | 1 | 1 | 1 | 0 | 1 | 0 | 1 | 6 |
| Whamm | 434204 | NM_001004185 | 1 | 1 | 1 | 1 | 0 | 1 | 0 | 1 | 6 |
| Ints2 | 70422 | NM_027421 | 1 | 1 | 1 | 0 | 0 | 1 | 1 | 1 | 6 |
| Sik1 | 17691 | NM_010831 | 1 | 1 | 1 | 1 | 0 | 1 | 0 | 1 | 6 |
| Fancm | 104806 | NM_178912 | 1 | 1 | 1 | 0 | 0 | 1 | 1 | 1 | 6 |
| Slc35e2 | 320541 | NM_177186 | 1 | 1 | 1 | 1 | 0 | 1 | 0 | 1 | 6 |
| Zrsr2 | 22184 | NM_178794 | 1 | 1 | 1 | 1 | 0 | 1 | 0 | 1 | 6 |
| Gm16515 | 24083 | NM_025294 | 1 | 1 | 1 | 0 | 0 | 1 | 1 | 1 | 6 |
| Vma21 | 67048 | NM_001081356 | 1 | 1 | 1 | 1 | 0 | 1 | 0 | 1 | 6 |
| AI593442 | 330941 | NM_178906 | 1 | 1 | 1 | 0 | 0 | 1 | 1 | 1 | 6 |


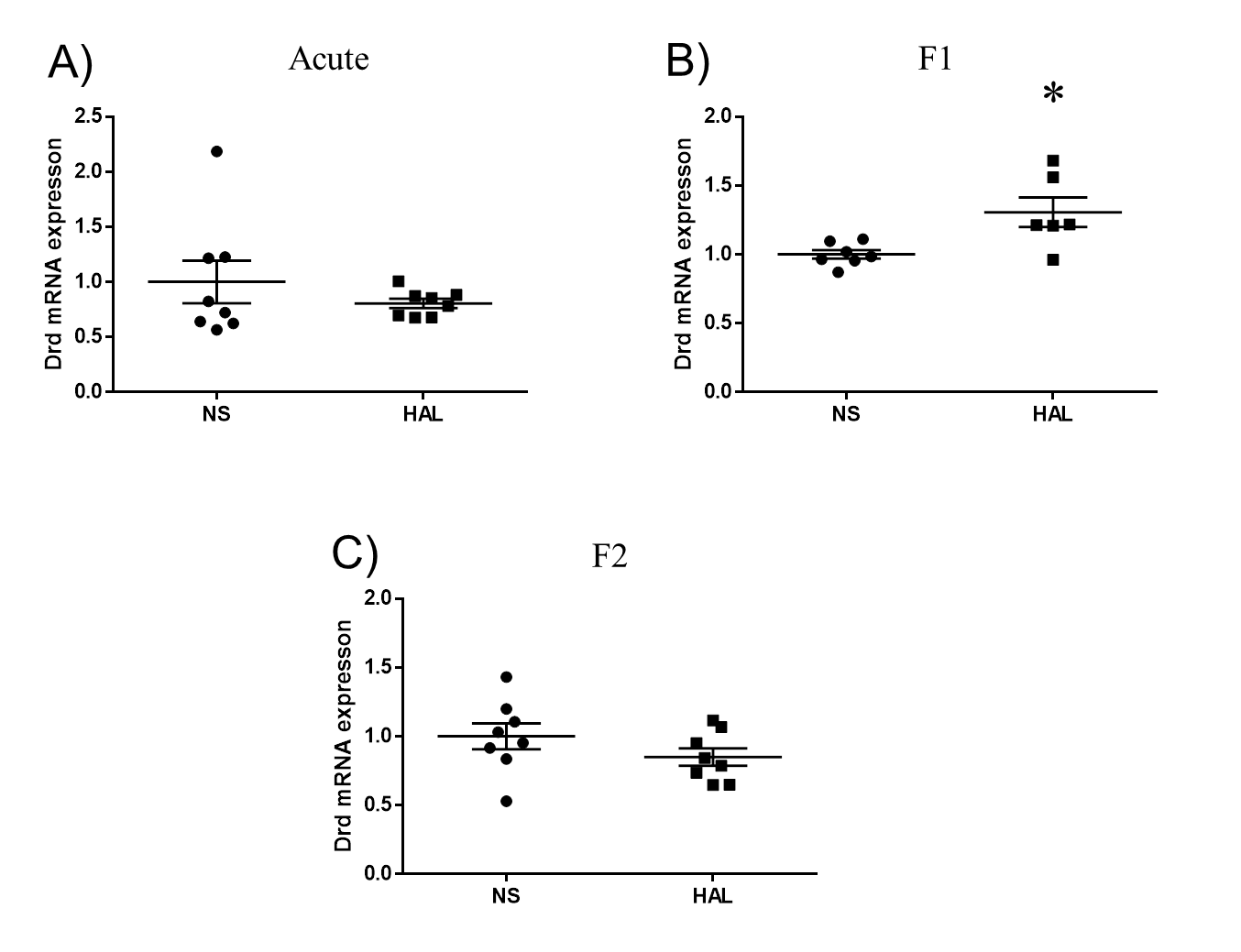


**Supplementary Figure 1. Drd2 mRNA expressions in HAL models.**

The y-axis represents the ratio of relative expression values of the NS and HAL groups. Drd2 mRNA expression was measured with qPCR in samples from A) SC, B) F1, and C) F2 offspring. * p < 0.05. SC, subchronic model; HAL, haloperidol; NS, normal saline.


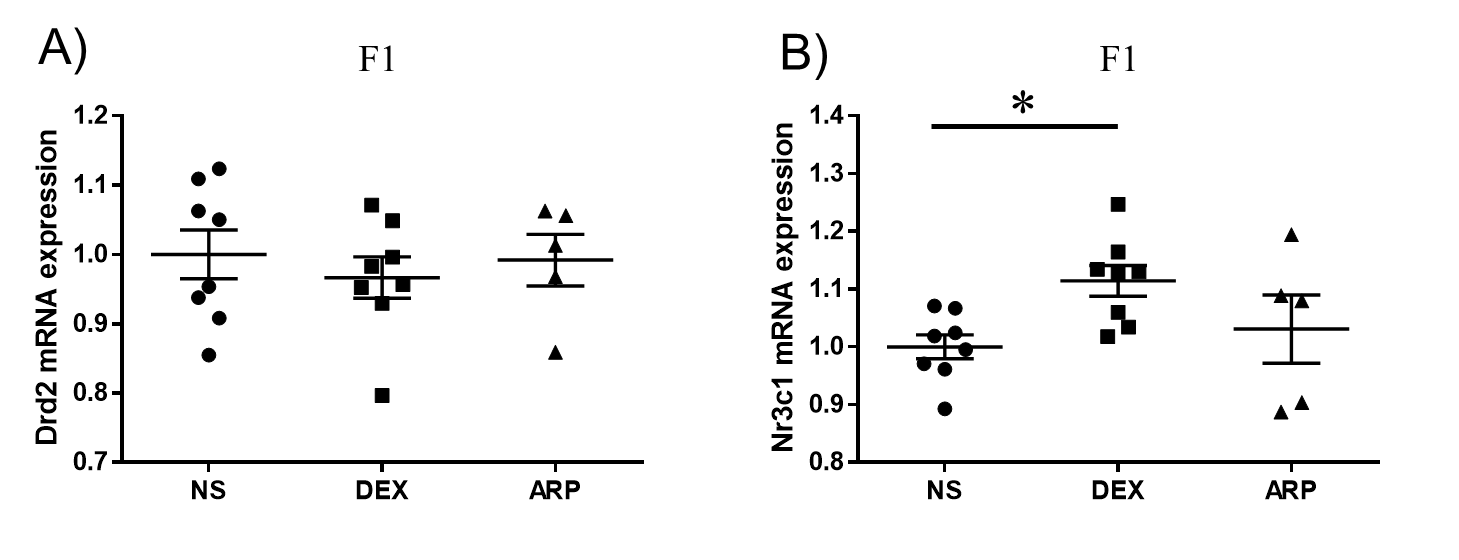


**Supplementary Figure 2. Drd2 and Nr3c1 mRNA expressions in ARP models.**

The y-axis represents the ratio of relative expression values of the NS, DEX, ARP groups. Drd2 (A) and Nr3c1 (B) mRNA expression was measured with qPCR in samples from F1 offspring. * p < 0.05. ARP, aripiprazole, DEX, 10% of β-cyclodextrin; haloperidol; NS, normal saline.


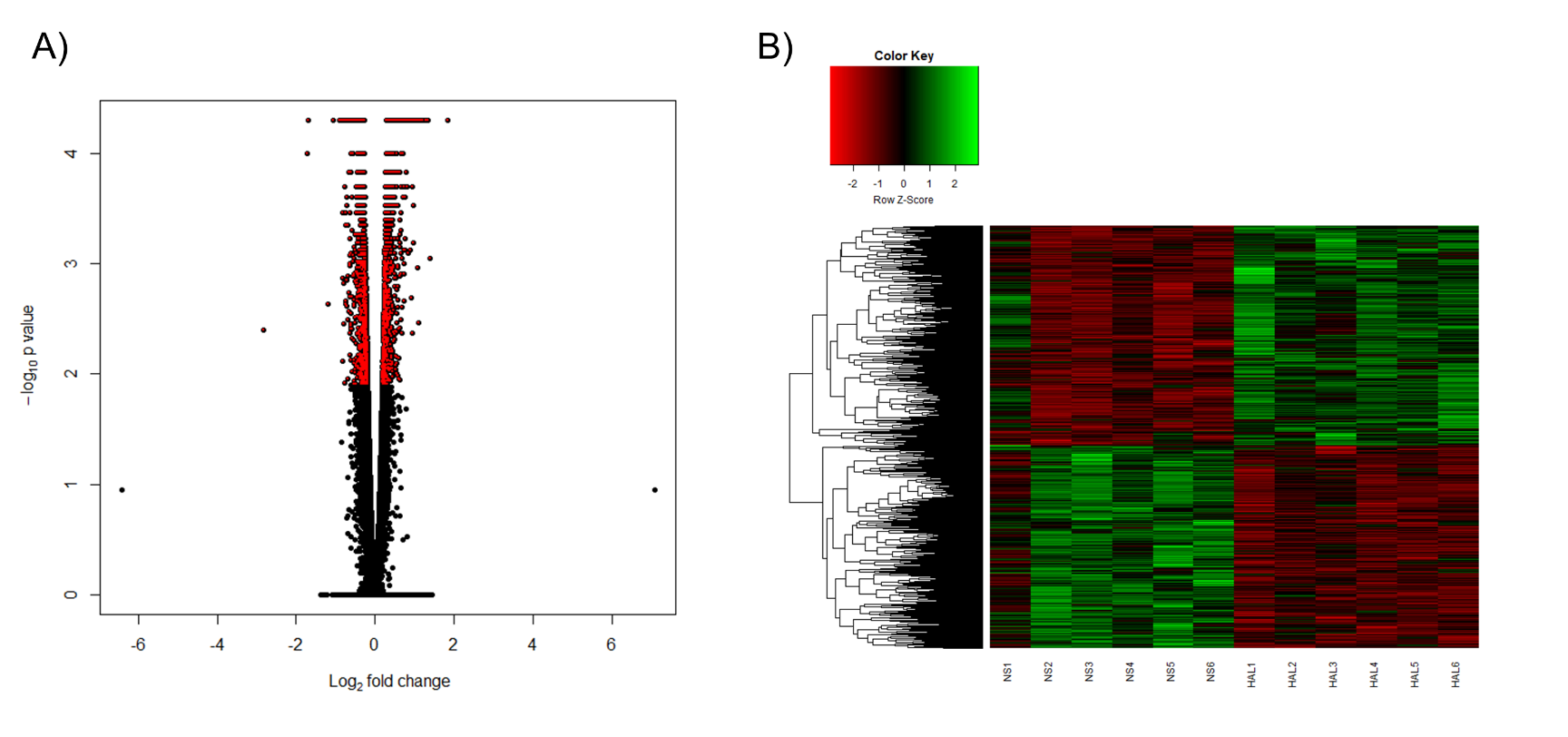


**Supplementary Figure 3. RNA sequencing data in a volcano plot and heatmap.**

(A) FPKM values of 12,941 detected genes were plotted in a volcano plot. The y-axis corresponds to the significance level represented with the log_10_P value, and the x-axis displays the log_2_ fold change value. The red dots represent significantly (p < 0.05 and q < 0.05) upregulated genes in the HAL group; the blue dots represent significantly (p < 0.05 and q < 0.05) downregulated genes in the HAL group. The dotted horizontal line indicates p = 0.05 [–log_10_ (1.30)]. (B) High expression genes are shown in red on the map; low expression genes are shown in green. The 1,370 upregulated and 1,260 downregulated DEGs were used to create the heatmap. DEGs, differentially expressed genes, HAL, haloperidol; NS, normal saline.

**
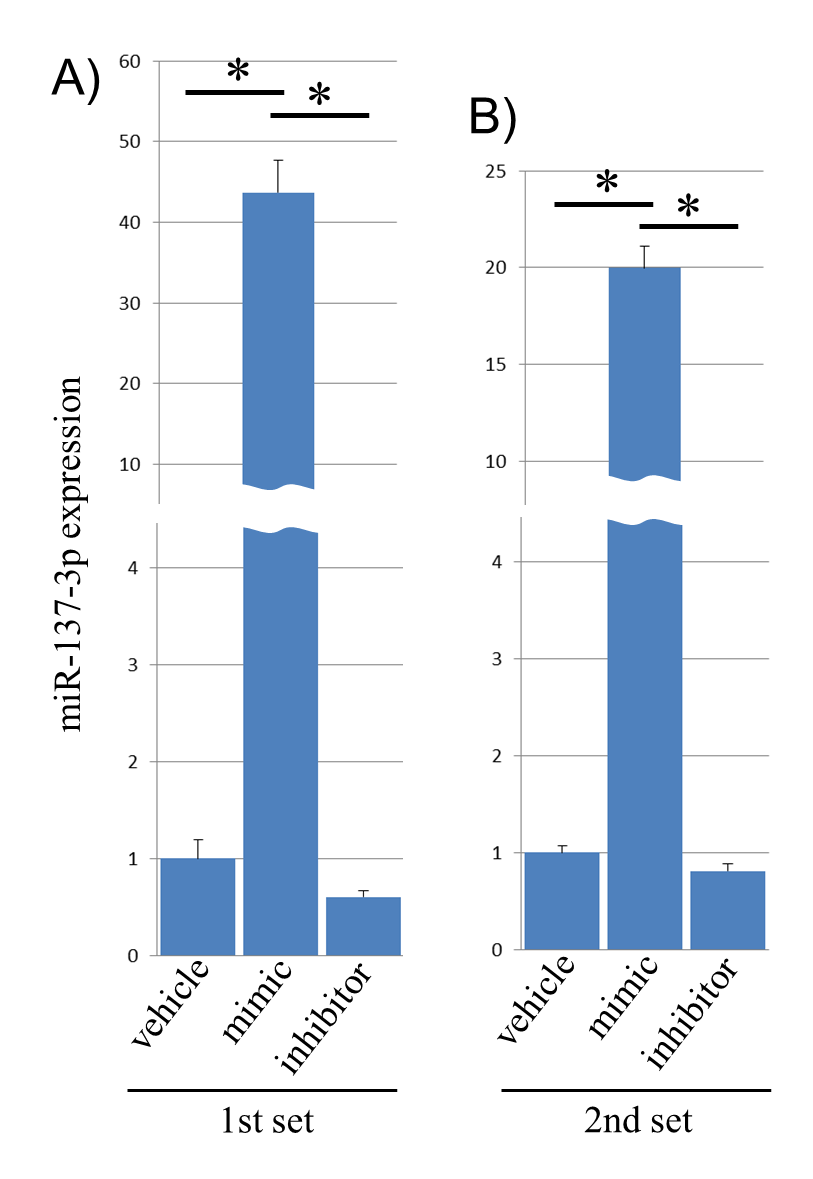
**

**Supplementary Figure 4. miR-137-3p expression in Neuro2a cells transfected miR-137-3p mimic and inhibitor.**

The y-axis represents the ratio of relative expression values of the vehicle, miR-137-3p mimic, and miR-137-3p inhibitor. miR-137-3p expression was measured with qPCR in the A) 1st and B) 2nd sets. * p < 0.05.

**Supplementary Figure 5
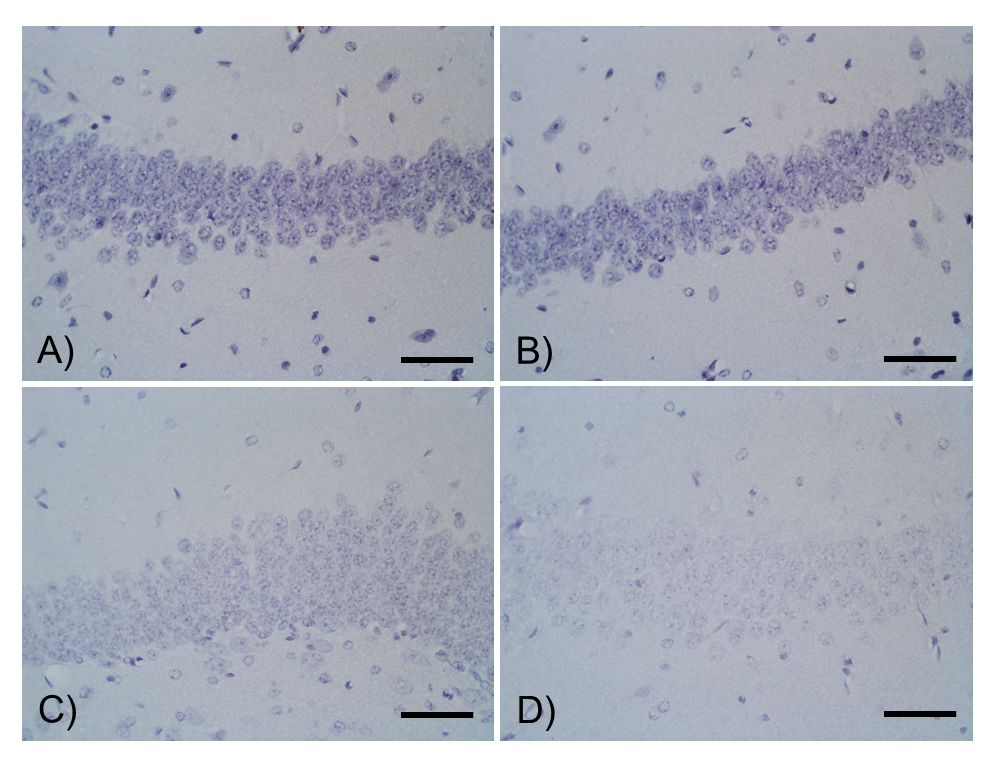
.**

**TUNEL staining and immunohistochemistry for assessing apoptosis in the hippocampus**

No TUNEL-positive cells were observed in either haloperidol-treated (A) or control mice (B). Light microscopic observations of the hippocampus stained with the caspase 3 antibody. No caspase 3 immunoreactivity was observed in either haloperidol-treated (C) or control mice (D). Scale bars: 10 µm.
